# Supplementary material for: Durvalumab and cediranib with and without olaparib in recurrent ovarian cancer: a phase II proof-of-concept study
Source: Nat Commun. 2026 Mar 16;17:4160. doi: 10.1038/s41467-026-70785-6 (PMC13153414; doi:10.1038/s41467-026-70785-6)
Supplement: Supplementary file 1 — Supplementary Information [file 41467_2026_70785_MOESM1_ESM.pdf]

Supplementary Information

Durvalumab and cediranib with and without olaparib in recurrent ovarian cancer: a phase II proof-of-concept study

Junya Tabata[1+], Tzu-Ting Huang[1+*], Elena Giudice[1,2], Kristen R. Ibanez[1,3], Jayakumar R. Nair[1], Aanika Balaji Warner[1], Britanny B. Solarz[1], Valentina Bolanos[1], Bernadette Redd[4], Nahoko Sato[5], Shraddha Rastogi[5], Sunmin Lee[5], Roshan L. Shrestha[5], Alexander Y. Mitrophanov[6], Stanley Lipkowitz[1], Kevin Conlon[1], Chien Chu Huang[1], and Jung-Min Lee[1,7*]

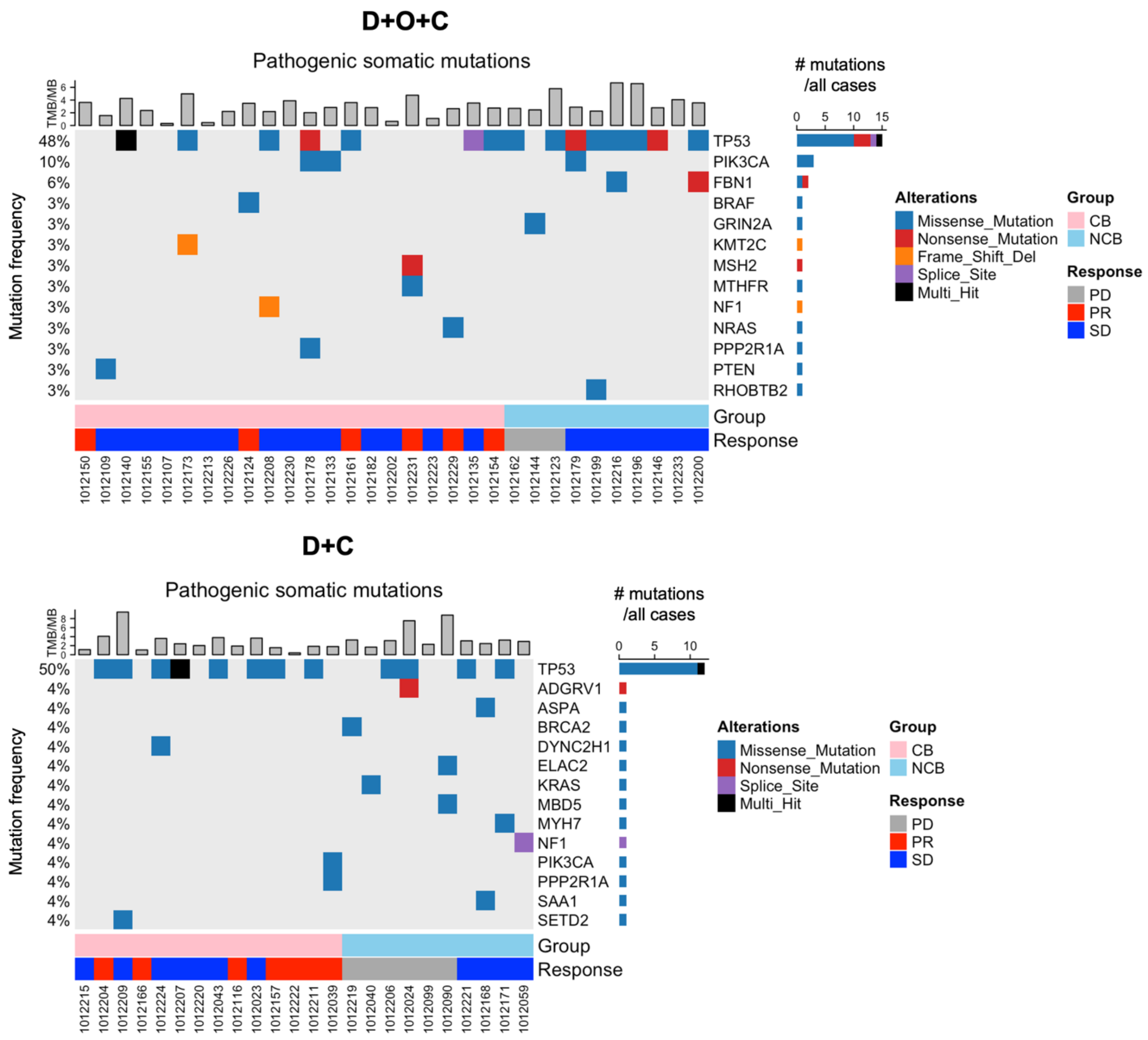

Supplementary Fig. 1: Pathogenic somatic mutations unlikely predict the response to D+O+C or D+C.

Whole-exome sequencing data analysis revealed the genetic alterations in D+O+C (*n* = 31) and D+C (*n* = 24) arms. Source data are provided as a Source Data file. Abbreviations: CB, clinical benefit; D+O+C, durvalumab, cediranib, and olaparib; D+C, durvalumab plus cediranib; NCB, no clinical benefit; PD, progression disease; PR, partial response; SD, stable disease; TMB/MB, tumor mutation burden per megabase.

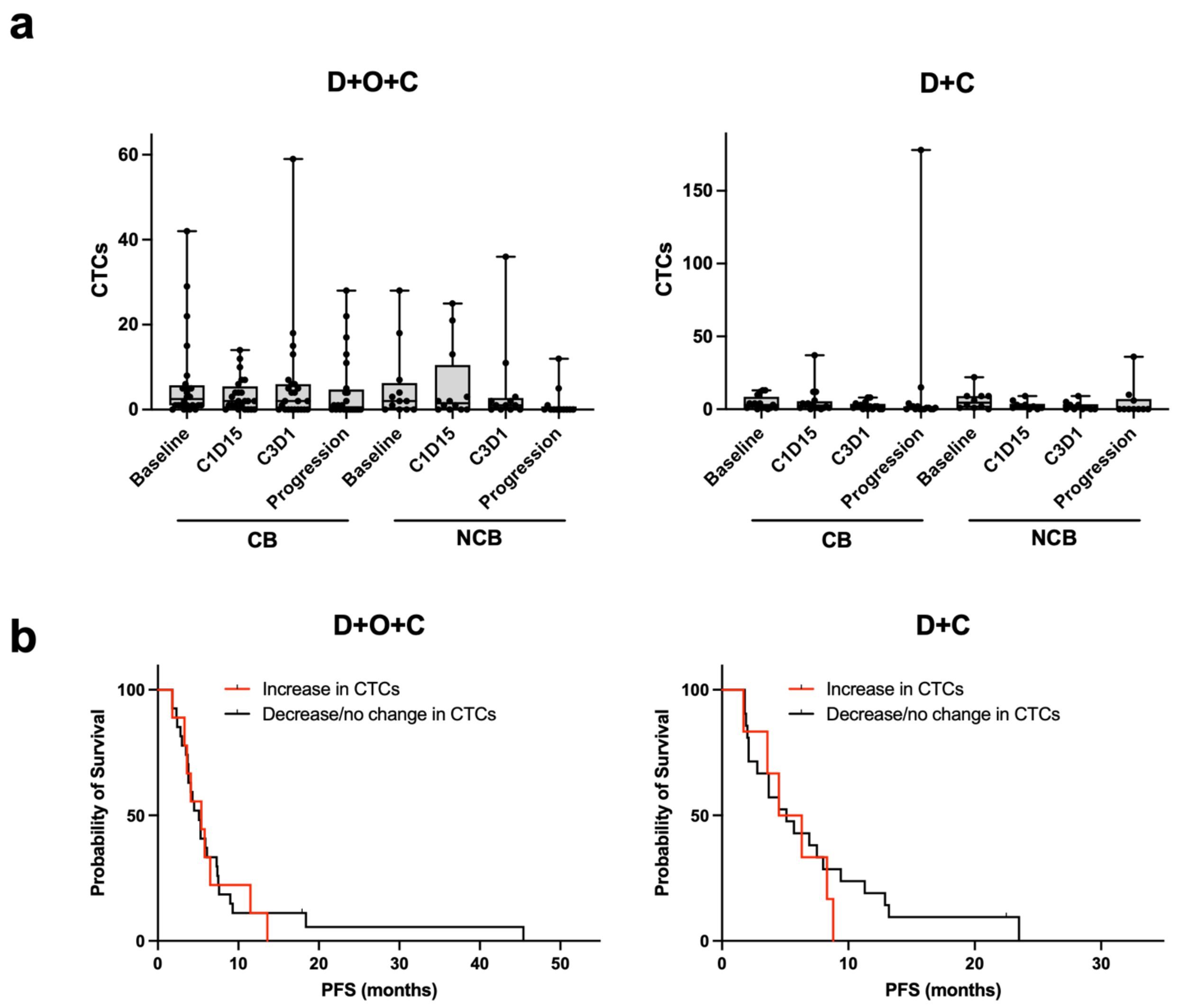

D+O+C	Events	Median PFS (Months)	Hazard ratio (95% CI)	log-rank p
Increase	27	5.4 (1.8-11.5)	1.08 (0.499-2.34)	0.84
Decrease/ no change	9	5.1 (3.7-7.3)		

D+C	Events	Median PFS (Months)	Hazard ratio (95% CI)	log-rank p
Increase	21	5.4 (1.7-∞)	1.38 (0.506-3.77)	0.47
Decrease/ no change	6	5.1 (2.1-8.0)		

Supplementary Figure 2: CTCs analyses.

a Dynamic changes of CTCs between baseline, C1D15, C3D1 and at progression in D+O+C (CB, *n* = 24; NCB, *n* = 12) and D+C (CB, *n* = 16; NCB, *n* = 10). No changes were observed in the count per tube of EpCAM+ CTCs in patients with CB or NCB. Non-parametric Wilcoxon rank sum test (two-sided) was used for unpaired samples, while Wilcoxon matched-pairs test (two-sided) was used to analyze paired samples. The boxes extend from min to max values, with the median depicted by a horizontal line. **b** Kaplan–Meier estimates of PFS in patients with EpCAM+ MUC1+ CTCs versus no change or increase in EpCAM+ MUC1+ CTCs. The PFS was assessed by Kaplan-

Meier survival plots, with the hazard ratio with 95% CI and logrank p values. Source data are provided as a Source Data file. Abbreviations: C1D15, cycle 1 day 15; C3D1, cycle 3 day 1; CB, clinical benefit; CTCs, circulating tumor cells; CI, confidence interval; D+O+C, durvalumab, cediranib, and olaparib; D+C, durvalumab plus cediranib; EpCAM, epithelial cell adhesion molecules; NCB, no clinical benefit; PFS, progression-free survival.

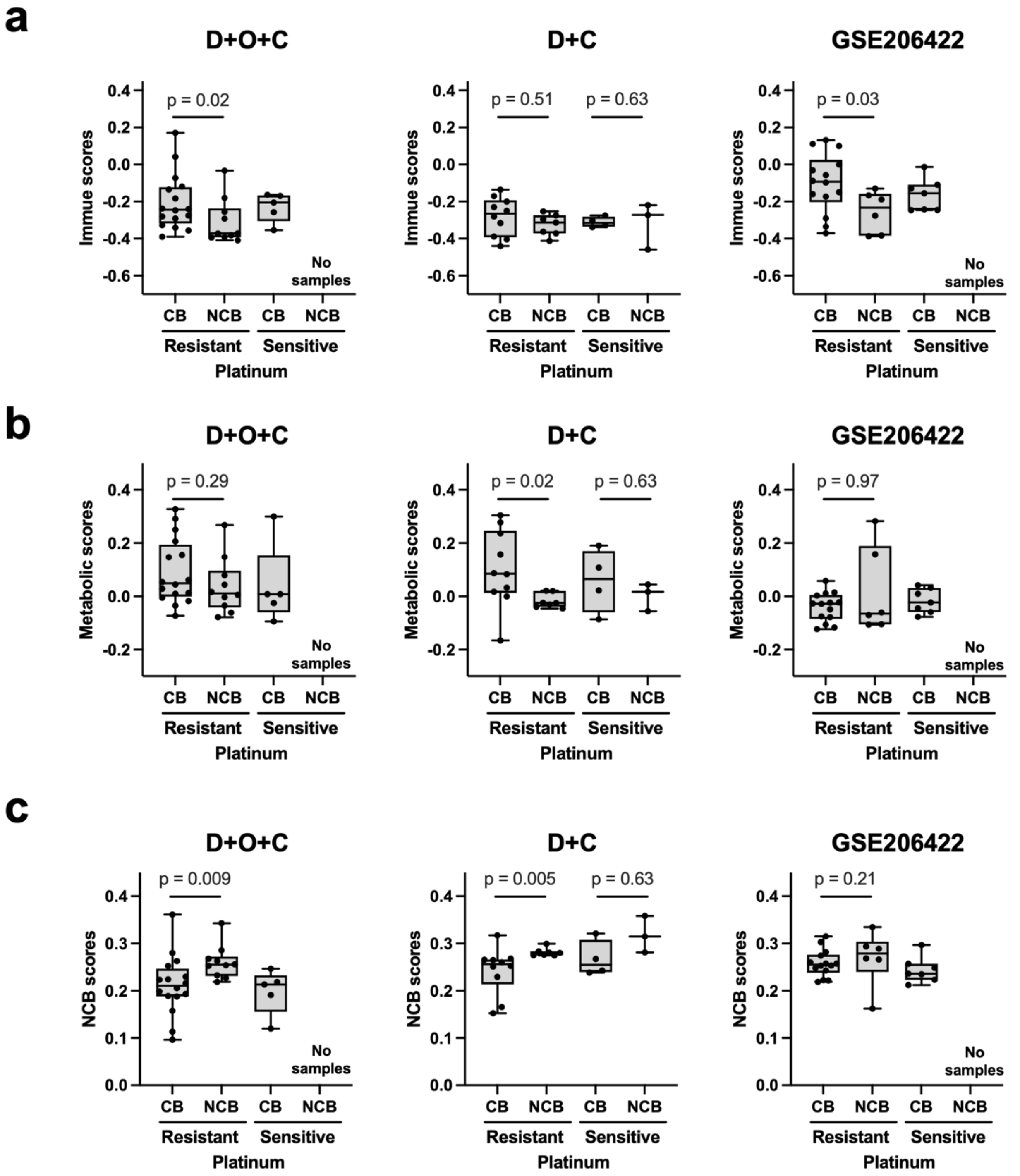

Supplementary Figure 3: Predictive performance of signatures stratified by clinical platinum status.

a-c Boxplots illustrate immune (a), metabolic (b) and NCB (c) signature scores in patients with CB versus NCB, grouped by prior platinum sensitivity across the D+O+C (platinum-resistant: 16

CB and 10 NCB, platinum-sensitive: 5 CB), D+C (platinum-resistant: 10 CB and 7 NCB, platinum-sensitive: 4 CB and 3 NCB), and GSE206422 (platinum-resistant: 14 CB and 6 NCB, platinum-sensitive: 7 CB) datasets. The boxes extend from min to max values, with the median depicted by a horizontal line. Data from were analyzed using two-sided Wilcoxon rank-sum test. Source data are provided as a Source Data file. Abbreviations: C1D15, cycle 1 day 15; C3D1, cycle 3 day 1; CB, clinical benefit; CTCs, circulating tumor cells; CI, confidence interval; D+O+C, durvalumab, cediranib, and olaparib; D+C, durvalumab plus cediranib; EpCAM, epithelial cell adhesion molecules; NCB, no clinical benefit; PFS, progression-free survival.

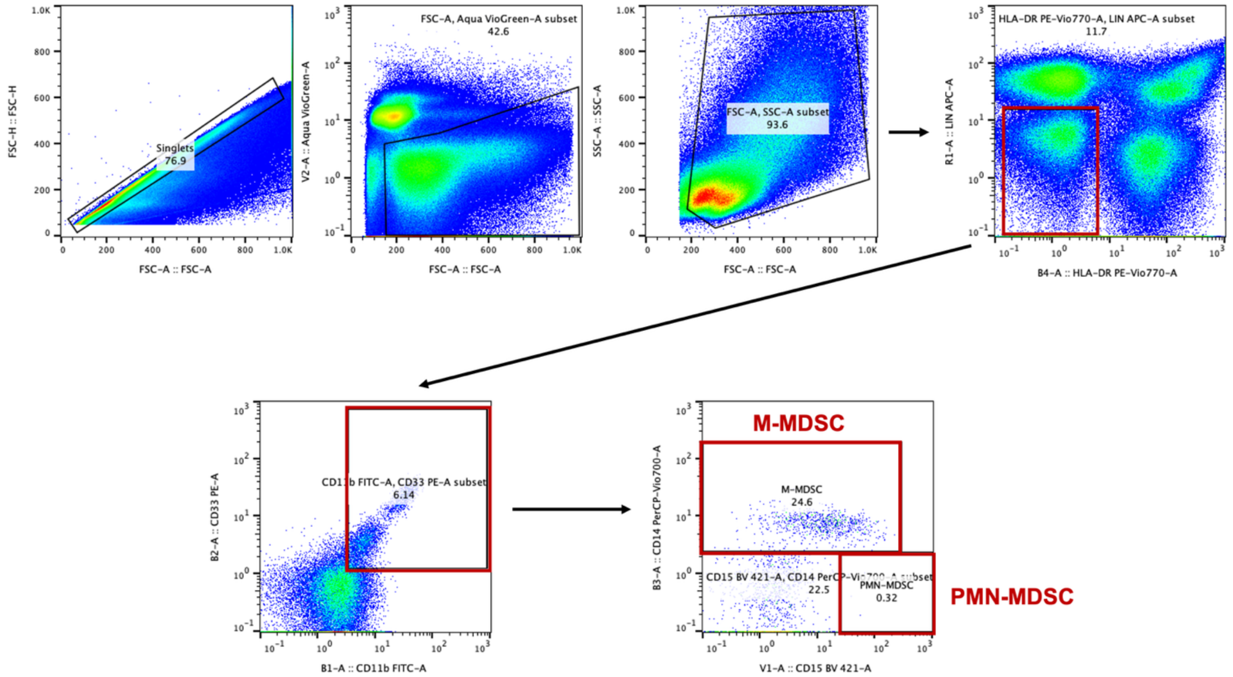

Supplementary Fig. 4: Gating strategy for identification of M-MDSC and PMN-MDSC subsets.

Viable singlet cells were first gated, followed by exclusion of lineage-negative HLA-DR$^-$ cells. CD33$^+$CD11b$^+$ myeloid cells were subsequently identified. M-MDSCs were defined as CD11b$^+$CD14$^+$CD15$^-$HLA-DR$^-$ cells, and PMN-MDSCs as CD11b$^+$CD14$^-$CD15$^+$HLA-DR$^-$ cells. Representative plots are shown. Abbreviations: FSC/SSC, forward and side scatter; HLA-DR, human leukocyte antigen-DR isotype; MDSC, myeloid-derived suppressor cell; PBMCs, peripheral blood mononuclear cells; M-MDSCs, monocytic MDSCs; PMN-MDSCs, polymorphonuclear MDSCs.

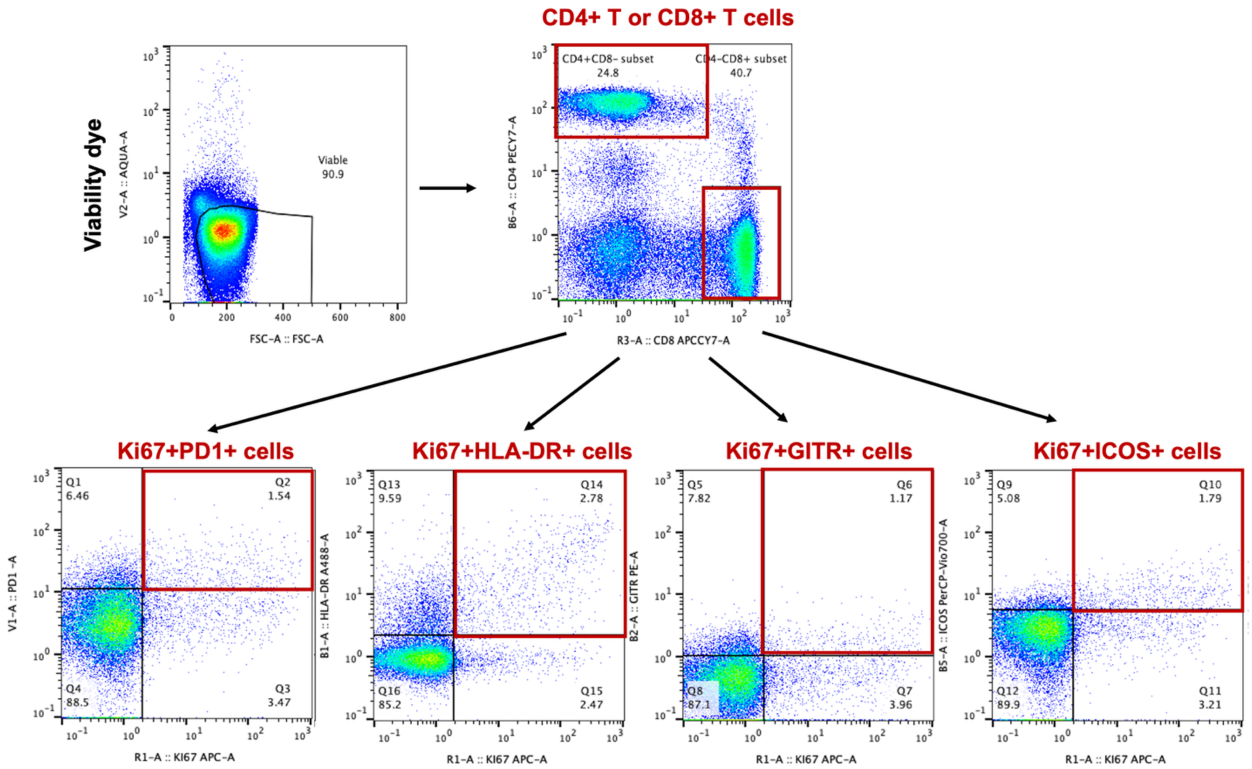

Supplementary Fig. 5: Gating strategy for functional analysis of CD4⁺ and CD8⁺ T cells. Viable singlet CD4⁺ and CD8⁺ T cells were gated and subsequently analyzed for expression of the proliferation marker Ki-67 and the activation markers PD-1, HLA-DR, GITR, and ICOS. Representative plots are shown. Abbreviations: GITR, glucocorticoid-induced tumor necrosis factor receptor; HLA-DR, human leukocyte antigen-DR isotype; ICOS, inducible T-cell costimulatory; Ki-67, marker of proliferation Ki-67; PBMCs, peripheral blood mononuclear cells; PD-1, programmed cell death protein 1.

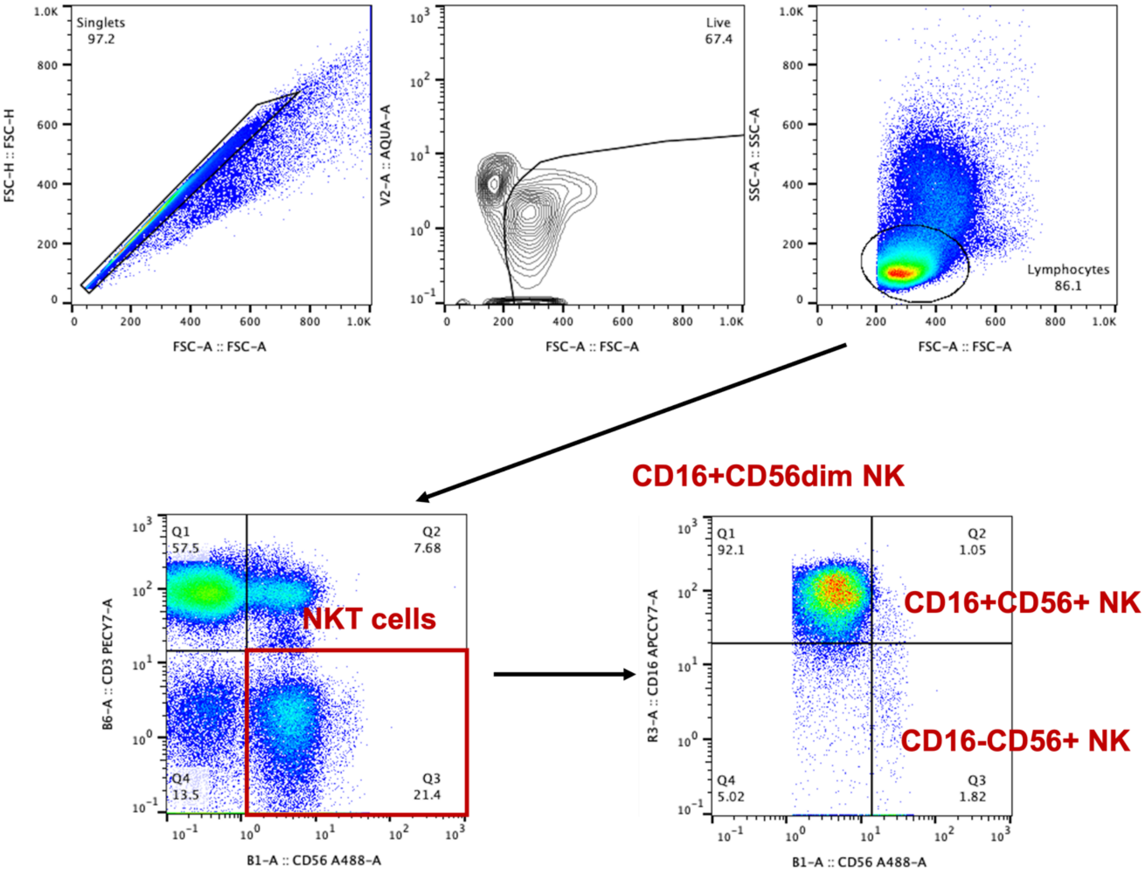

Supplementary Fig. 6: Gating strategy for NK and NKT cell subsets.

PBMCs were gated on singlets and viable cells, followed by identification of lymphocytes based on forward and side scatter (FSC/SSC) properties. NKT cells were defined as $CD3^{+}CD56^{+}$ cells. NK cells were identified as $CD3^{-}CD56^{+}$ cells and further subdivided into $CD16^{+}$CD56^dim and $CD16^{-}CD56^{+}$ subsets. Representative plots are shown. Abbreviations: FSC/SSC, forward and side scatter; PBMCs, peripheral blood mononuclear cells; NK, natural killer; NKT, natural killer T.

Supplementary Note: Study Protocol

Abbreviated Title: Ph I/II Durvalumab +O/+C/+OC

NIH Protocol #: 15-C-0145

Version Date: September 9, 2021

NCT Number: NCT02484404

Title: Phase I/II study of the anti-programmed death ligand-1 antibody Durvalumab (MEDI4736) in combination with olaparib and/or cediranib for advanced solid tumors and advanced or recurrent ovarian, triple negative breast, lung, prostate and colorectal cancers

NCI Principal Investigator: Jung-min Lee, M.D.
Women's Malignancies Branch (WMB), CCR, NCI
Building 10, Room 4B54
National Institutes of Health
9000 Rockville Pike
Bethesda, MD 20892
Phone: (240) 760-6128
Email: leej6@mail.nih.gov

Investigational Agents:

Drug Name:	Durvalumab (MEDI4736)	Olaparib (AZD 2281)	Cediranib
IND Number:	126389	126389	126389
Sponsor:	Center for Cancer Research	Center for Cancer Research	Center for Cancer Research
Manufacturer:	MedImmune, LLC, a wholly owned subsidiary of AstraZeneca	AbbVie, under contract with AstraZeneca	AstraZeneca
Supplier:	AstraZeneca	AstraZeneca	AstraZeneca

PRÉCIS

Background:

- Disruption of the immune checkpoint PD-1/PD-L1 pathway yielded clinical activity in subsets of advanced solid tumors, such as melanoma and lung cancer.
- Olaparib (O), a PARP inhibitor (PARPi), has demonstrated single agent activity in recurrent ovarian cancer (OvCa), and subsets of prostate, triple negative breast or lung cancers.
- Our recent randomized phase 2 study showed that O and cediranib (C), a VEGFR1-3 inhibitor was clinically superior to O alone in platinum-sensitive recurrent OvCa.
- We hypothesize that increased DNA damage by PARP inhibition and/or reduced angiogenesis by VEGFR inhibition will complement the anti-tumor activity of an immune checkpoint inhibitor, Durvalumab, in recurrent OvCa and other solid tumors.

Objectives:

- Phase I: To determine the recommended phase II dose (RP2D) and the safety of doublet therapies (durvalumab/olaparib [Durvalumab+O] and durvalumab/cediranib [Durvalumab+C]) and triplet therapy (Durvalumab+O+C) in patients with advanced solid tumors.
- Phase II Cohort 1 OvCa; Durvalumab+O, Durvalumab+C and Durvalumab+O+C arms: To determine clinical efficacy as measured by overall response rate (ORR)
- Phase II Cohort 2 non-small cell lung cancer (NSCLC); Durvalumab+O and Durvalumab+C arms: To determine clinical efficacy as measured by progression-free survival (PFS)
- Phase II Cohort 3 small cell lung cancer (SCLC); Durvalumab+O arm: To determine clinical efficacy as measured by ORR
- Phase II Cohort 4 metastatic castrate-resistant prostate cancer (mCRPC); Durvalumab+O arm: To determine clinical efficacy as measured by PFS
- Phase II Cohort 5 triple negative breast cancer (TNBC); Durvalumab+O arm: To determine clinical efficacy as measured by ORR
- Phase II Cohort 6 colorectal cancer (CRC): C+Durvalumab arm: To determine clinical efficacy as measured by PFS

Eligibility:

- Phase I: Advanced or recurrent solid tumors with evaluable disease.
- Phase II Cohort 1 Durvalumab+O, Durvalumab+C and Durvalumab+O+C arms: Advanced or recurrent OvCa
- Phase II Cohort 2 Durvalumab+O and Durvalumab+C arms: Advanced or recurrent NSCLC

- Phase II Cohort 3 Durvalumab+O arm: Advanced or recurrent SCLC
- Phase II Cohort 4 Durvalumab+O arm: mCRPC
- Phase II Cohort 5 Durvalumab+O arm: Advanced or recurrent TNBC
- Phase II Cohort 6 C+Durvalumab arm: Advanced or recurrent CRC
- Patients must be off prior chemotherapy, radiation therapy or biologic therapy for at least 3 weeks. mCPRC patients (Cohort 4) may be on hormonal therapy with GnRH agonists/antagonists.
- Adults with ECOG performance status 0-2, and adequate organ and marrow function.

Design:

- Phase I: Durvalumab+O, Durvalumab+C and Durvalumab+O+C will dose escalate simultaneously. Durvalumab will be administered once every 2 weeks or once every 4 weeks until disease progression. O tablets and C will be given orally on a continuous or intermittent dosing schedule. The DLT period will be one cycle, 28 days. Patients on the 2-week schedule greater than one year will be changed to the 4-week schedule until progression.
  - Durvalumab+O: Durvalumab (3 mg/kg or 10 mg/kg IV every 2 weeks, or a fixed dose of 1500 mg every 4 weeks) and O tablets (150 mg or 200 mg or 300 mg BID)
  - Durvalumab+C: Durvalumab (3 mg/kg or 10 mg/kg IV every 2 weeks, or a fixed dose of 1500 mg every 4 weeks) and C (15 mg or 20 mg or 30 mg daily or 5 days/week)
  - Durvalumab+O+C: Durvalumab (a fixed dose of 1500mg every 4 weeks) with O tablets (200 mg or 300 mg BID) and C (15 mg or 20 mg 5 days/week)
- Phase II Cohort 1 OvCa Durvalumab+O arm: Patients will be treated with Durvalumab+O at RP2D (O 300mg tablets bid daily and Durvalumab at 1500 mg IV every 4 weeks).
- Phase II Cohort 1 OvCa Durvalumab+C arm: Patients will be treated with Durvalumab+C at RP2D (C 20mg once a day [5 days on/2 days off] and Durvalumab at 1500 mg every 4 weeks).
- Phase II Cohort 1 OvCa Durvalumab+O+C arm: Patients with OvCa (Cohort 1) will be treated with RP2D (O tablets 300mg BID, C 20mg once a day [5 days on/2 days off] and Durvalumab at 1500 mg every 4 weeks).
- Phase II Cohort 2 NSCLC; Durvalumab+O arm: Patients will be treated with Durvalumab+O at RP2D (O 300mg tablets bid daily and Durvalumab at 1500 mg IV every 4 weeks).
- Phase II Cohort 2 NSCLC; Durvalumab+C arm: Patients will be treated with Durvalumab+C at RP2D (C 20mg once a day [5 days on/2 days off] and Durvalumab at 1500 mg every 4 weeks).
- Phase II Cohort 3 SCLC; Durvalumab+O arm: Patients will be treated with Durvalumab+O at RP2D (O 300mg tablets bid daily and Durvalumab at 1500 mg IV every 4 weeks).
- Phase II Cohort 4 mCRPC; Durvalumab+O arm: Patients will be treated with Durvalumab+O at RP2D (O 300mg tablets bid daily and Durvalumab at 1500 mg IV every 4 weeks).

- Phase II Cohort 5 TNBC; Durvalumab+O arm: Patients will be treated with Durvalumab+O at RP2D (O 300mg tablets bid daily and Durvalumab at 1500 mg IV every 4 weeks).
- Phase II Cohort 6 CRC; C+Durvalumab arm: Patients in the Cohort 6 will be treated with C 20mg daily alone for 14 days followed by the combination at RP2D (C 20mg once a day [5 days on/2 days off] and Durvalumab at 1500 mg every 4 weeks).
- Phase II Correlative studies: Research samples including whole blood, CTCs, cell free DNA and plasma will be obtained at pretreatment, prior to cycle 1 day 15, prior to cycle 3 day 1 and at progression. Mandatory baseline core biopsy and two optional biopsies will be obtained.
- Patients will be evaluated for toxicity every 4 weeks by CTCAEv4.0, and for response every two cycles (8 weeks) by RECIST 1.1. Patients with mCRPC (Durvalumab+O Cohort 4) will be evaluated for response initially at 8 weeks then every 12 weeks using RECIST v1.1 criteria as per the Prostate Cancer Clinical Trials Working Group 2 (PCWG2).

STUDY SCHEMA

PHASE I: DOSE ESCALATION

Durvalumab+O Dose Escalation Schedule Table

Dose Level (DL)	Durvalumab (intravenously)	Olaparib tablet (oral, twice a day)
DL -2	3 mg/kg every 2 weeks	150 mg
DL -1	3 mg/kg every 2 weeks	200 mg
**DL 1 (starting dose)**	**10 mg/kg every 2 weeks**	**200 mg**
DL 2	10 mg/kg every 2 weeks	300 mg
DL 3	A fixed dose of 1500mg every 4 weeks	300 mg

Durvalumab+C Daily Schedule Dose Escalation Table

Dose Level (DL)	Durvalumab (intravenously)	Cediranib (oral, once daily)
DL -2	3 mg/kg every 2 weeks	15 mg
DL -1	3 mg/kg every 2 weeks	20 mg
**DL 1 (starting dose)**	**10 mg/kg every 2 weeks**	**20 mg**
DL 2	10 mg/kg every 2 weeks	30 mg

Durvalumab+C Intermittent Schedule Dose Escalation Table

Dose Level (DL)	Durvalumab (intravenously)	Cediranib (oral, 5 days on/2 days off)
DL -2	A fixed dose of 500mg every 4 weeks	15 mg
DL -1	A fixed dose of 1500mg every 4 weeks	15 mg

**DL 1 (starting dose)**	**A fixed dose of 1500mg every 4 weeks**	**20 mg**

Durvalumab+O+C Dose Escalation Table

**Dose Level (DL)**	**Durvalumab (intravenously)**	**Olaparib tablet (oral, twice a day)**	**Cediranib (oral, 5 days on/ 2 days off)**
DL -1	A fixed dose of 1500 mg every 4 weeks	200 mg	15 mg
**DL 1 (starting dose)**	A fixed dose of 1500 mg every 4 weeks	300 mg	15 mg
DL 2	A fixed dose of 1500 mg every 4 weeks	300 mg	20 mg

PHASE II SCHEMA

PHASE II Cohorts 1-5 Durvalumab+O Arm SCHEMA: OvCa (Cohort 1), NSCLC (Cohort 2), SCLC (Cohort 3), mCRPC (Cohort 4) AND TNBC (Cohort 5)

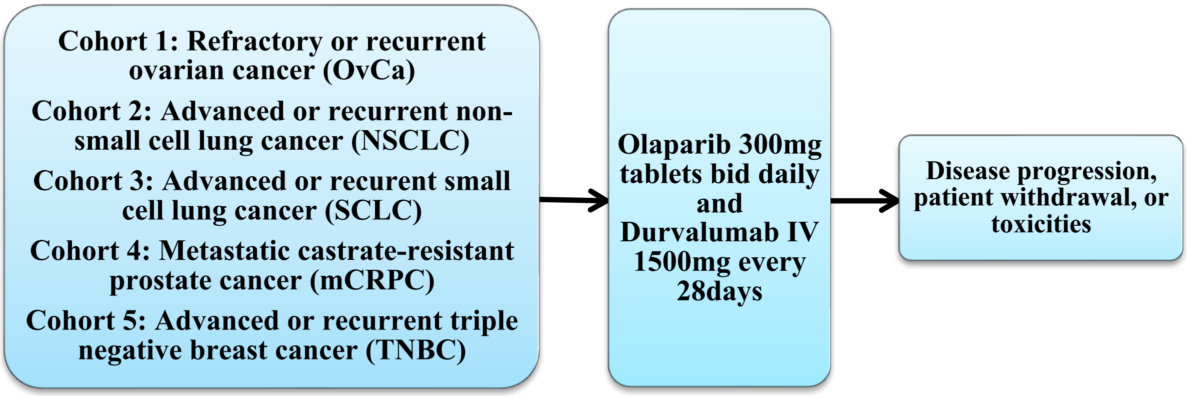

O 300mg tablets bid daily po, Durvalumab IV 1500mg q 4 weeks

O 300mg tablets bid daily po, Durvalumab IV 1500mg q 4 weeks

PBMCs: pretreatment, prior to cycle 1 day 15, pre- cycle 3 day 1, and progression (phase II only)

Tumor core biopsy: pretreatment and two optional bx (Phase II only)

Phase II Cohorts 1-2 Durvalumab+C Arm Schema: OvCa (Cohort 1) and NSCLC (Cohort 2)

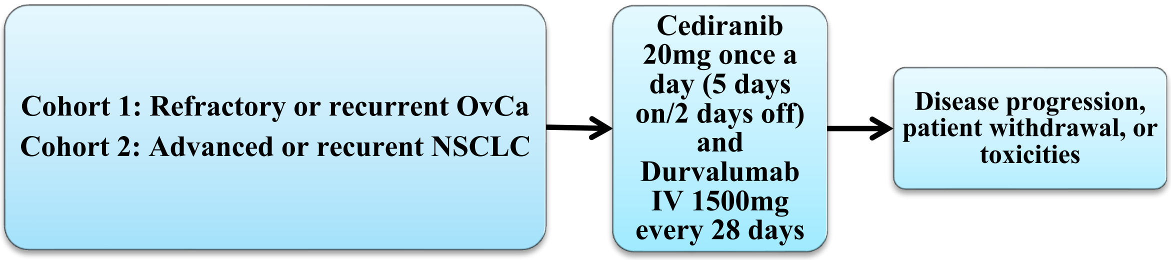

C 20mg 5 days/week po, Durvalumab IV 1500mg q 4 weks

C 20mg 5 days/week po, Durvalumab IV 1500mg q 4 weks

PBMCs: pretreatment, prior to cycle 1 day 15, pre- cycle 3 day 1, and progression (phase II only)

Tumor core biopsy: pretreatment and two optional bx (Phase II only)

* Randomization procedures do not apply to the Phase I and Phase II portions of the study. The arms will be open concurrently and treatment assignment for each patient will be at the PI's discretion and/or based on prior treatment history.

Phase II Cohort 6 C+Durvalumab Arm Schema (CRC)

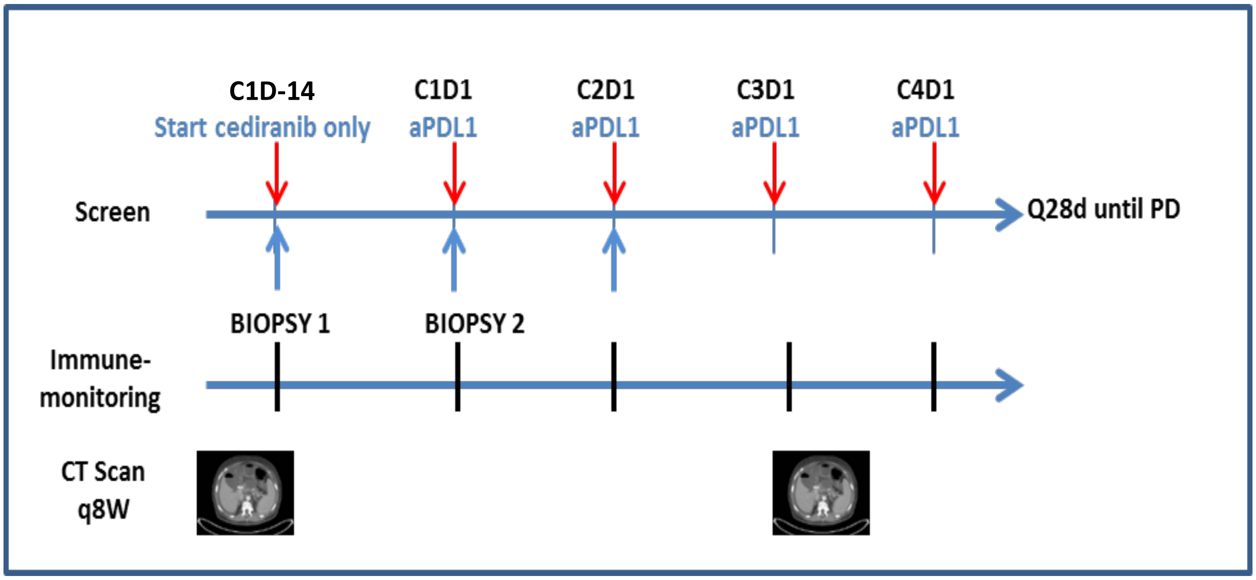

* Patients with CRC will be treated with C 20mg daily alone for 14 days (Day-14 through Day -1) followed by the combination at RP2D (C 20mg once a day [5 days on/2 days off] and Durvalumab at 1500 mg every 4 weeks). Of note, there is no Cycle 1 Day 0.

Phase II Cohort 1 MEDI+O+C Arm Schema (OvCa)

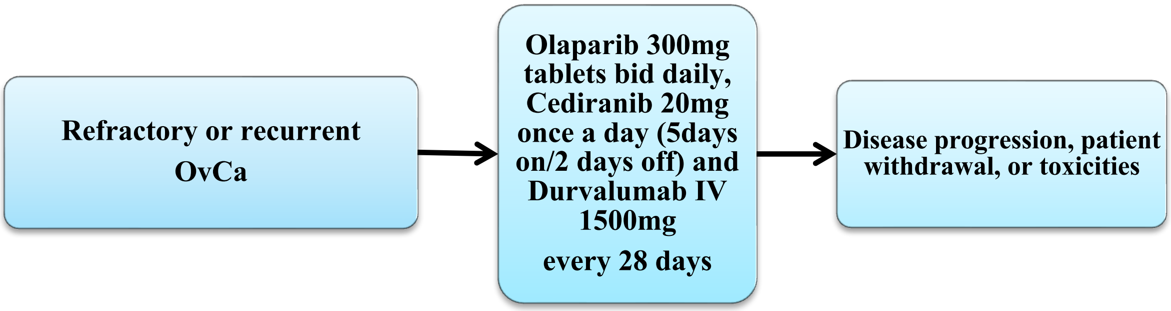

O 300mg tablets bid daily po,
C 20mg 5 days/week po,
Durvalumab IV 1500mg q 4 weks

O 300mg tablets bid daily po,
C 20mg 5 days/week po,
Durvalumab IV 1500mg q 4 weks

PBMCs: pretreatment, prior to cycle 1 day 15, pre- cycle 3 day 1, and progression (phase II only)

Tumor core biopsy: pretreatment and two optional bx (Phase II only)

TABLE OF CONTENTS

PRÉCIS 2
STUDY SCHEMA 5
Phase I: Dose Escalation 5
Phase II Schema 7
TABLE OF CONTENTS 10
TABLE OF TABLES 14
STATEMENT OF COMPLIANCE 16
1 INTRODUCTION 16
1.1 Study Objectives 16
1.2 Background and Rationale 19
1.3 Background for Correlative Studies 38
1.4 Rationale 41
1.5 Phase I Results and Implementation of Phase II 42
1.6 Pharmaceutical Manufacturer Updates 43
2 ELIGIBILITY ASSESSMENT AND ENROLLMENT 43
2.1 Phase I Study Eligibility Criteria 43
2.2 Phase II Study Cohort 1 Ovarian Cancer Eligibility Criteria (Durvalumab+O, Durvalumab+C AND Durvalumab+O+C) 47
2.3 Phase II Study cohort 5 Triple Negative Breast Cancer Eligibility Criteria (Durvalumab+O Only) 52
2.4 Phase II Study Non-Small Cell Lung Cancer (cohort 2; Durvalumab+O And Durvalumab+C) And Small Cell Lung Cancer (cohort 3; Durvalumab+O Only) Eligibility Criteria 56
2.5 Phase II Study Metastatic Castrate-Resistant Prostate Cancer cohort 4 Eligibility Criteria (Durvalumab+O Only) 60
2.6 Phase II Colorectal Cancer Cohort 6 (Durvalumab+C Only) 64
2.7 Screening Evaluation 68
2.8 Participant Registration and Status Update Procedures 70
2.9 Treatment Assignment and Randomization/Stratification Procedures 71
2.10 RESTRICTIONS DURING THE STUDY 74
3 STUDY IMPLEMENTATION 74

3.1 Study Design....74
3.2 Drug Administration....79
3.3 Self Administered IND agents....81
3.4 Dosing Delays/Dose Modifications And Management Of Toxicities Associated With Durvalumab, Olaparib, Cediranib....82
3.5 Study Calendar....110
3.6 Cost and Compensation....113
3.7 Criteria for Removal from Protocol Therapy and Off Study Criteria....114
4 CONCOMITANT MEDICATIONS/MEASURES....115
4.1 Prohibited Medications....115
4.2 Supportive Care....116
5 CORRELATIVE STUDIES....116
5.1 Rationale for Selected Endpoints....122
5.2 Tumor biopsies (phase II study only)....122
5.3 Studies Performed....123
5.4 Blood samples....126
5.5 Radiomics....130
5.6 Patient sample protections, storage, tracking, disposition....131
6 SAMPLE STORAGE, TRACKING AND DISPOSITION....131
6.1 Blood Processing Core (BPC)....131
7 DATA COLLECTION AND EVALUATION....133
7.1 Data Collection....133
7.2 Source Documents....133
7.3 Case Report Forms....134
7.4 Genomic Data Sharing Plan....134
7.5 Response Criteria....134
7.6 Toxicity Criteria....142
8 NIH REPORTING REQUIREMENTS/DATA AND SAFETY MONITORING PLAN..142
8.1 Definitions....142
8.2 OHSRP Office of Compliance and Training / IRB Reporting....142
8.3 NCI Clinical Director Reporting....143
8.4 NIH Required Data and Safety Monitoring Plan....143

9 SPONSOR SAFETY REPORTING 143
9.1 Definitions 143
9.2 Assessment of Safety Events 147
9.3 Reporting of Serious Adverse Events 148
9.4 Safety Reporting Criteria to the Pharmaceutical Collaborators 148
9.5 Reporting Pregnancy 149
9.6 Regulatory Reporting for Studies Conducted Under CCR-Sponsored IND 150
10 CLINICAL MONITORING PLAN 150
11 STATISTICAL CONSIDERATIONS 150
11.1 Phase I and Phase II Ovarian Cancer Cohort 1 (Durvalumab+O and Durvalumab+C) 150
11.2 Phase II triple negative breast cancer (TNBC) Cohort 5 (Durvalumab+O only) 152
11.3 Phase II non-small cell lung cancer (NSCLC) Cohort 2 (Durvalumab+O and Durvalumab+C) 153
11.4 Phase II small cell lung cancer (SCLC) Cohort 3 (Durvalumab+O only) 154
11.5 Phase II metastatic castrate-resistance prostate cancer (mCRPC) Cohort 4 (Durvalumab+O only) 154
11.6 Phase II colorectal cancer (CRC) Cohort 6 (C+Durvalumab only) 156
11.7 Phase II Durvalumab+O+C in patients with ovarian cancer 156
11.8 Expected patient enrollment and accrual ceiling 157
12 COLLABORATIVE AGREEMENTS 158
13 HUMAN SUBJECTS PROTECTIONS 158
13.1 Rationale For Subject Selection 158
13.2 Participation of Children 158
13.3 Participation of Subjects Unable to Give Consent 158
13.4 Evaluation of Benefits and Risks/Discomforts 159
13.5 Risks/Benefits Analysis 160
13.6 Consent Process and Documentation 161
13.7 Study Documentation 162
13.8 Records Retention 162
14 REGULATORY AND OPERATIONAL CONSIDERATIONS 163
14.1 STUDY DISCONTINUATION AND CLOSURE 163
14.2 QUALITY ASSURANCE AND QUALITY CONTROL 163

14.3 CONFLICT OF INTEREST POLICY 164
14.4 CONFIDENTIALITY AND PRIVACY 164
15 PHARMACEUTICAL INFORMATION: DRUG FORMULATION AND ADMINISTRATION 165
15.1 Durvalumab 165
15.2 Olaparib 169
15.3 Cediranib 171
16 REFERENCES 173
17 APPENDICES 186
17.1 Appendix A: Performance Status Criteria 186
17.2 Appendix B: Inhibitors and Inducers of CYP3A4 187
17.3 Appendix C: Oral Medication Diary 191
17.4 Appendix D: Patient’s Blood Pressure Diary (Durvalumab+C or Durvalumab+O+C Arm only) 195
17.5 Appendix E: Patient’s DIARRHEA Diary (ALL ARMS) 196
17.6 Appendix F: Suggested Anti-hypertensive Medications, Starting Doses and Hepatic Metabolism 198
17.7 Appendix G: Lee Laboratory Standard Operating Procedure-Tissue Core Collection 201
17.8 APPENDIX H: SOP FOR preserving a needle biopsy tissue in formalin for paraffin embedding 203
17.9 APPENDIX I: METHOD FOR PREPARING PBMC SAMPLES FROM BLOOD FOR PHARMACODYNAMIC (PD) STUDIES 204
17.10 APPENDIX J: Durvalumab DOSE CALCULATIONS 206
17.11 APPENDIX K: Dosing Modification and Toxicity Management Guidelines (TMG) for Immune-Mediated, Infusion-Related, and Non-Immune–Mediated Reactions (Durvalumab Monotherapy or in Combination with other Products) Version 14 October 2020 (CTCAE v5.0) 207

TABLE OF TABLES

Table 1. Durvalumab Monotherapy Clinical Studies (2015 IB)....33
Table 2. Durvalumab Combination Therapy Clinical Studies (2015 IB)*....34
Table 3. Durvalumab PK parameters from the first and steady-state doses....37
Table 4. MesoScaleDiscovery Panel of Chemokines and Cytokines....40
Table 5 Effective methods of contraception (two methods must be used)....74
Table 6. Durvalumab+O Dose Escalation Schedule....76
Table 7. Durvalumab-C Daily Schedule Dose Escalation Table....76
Table 8. Durvalumab+C Intermittent Schedule Dose Escalation Table....77
Table 9. Durvalumab+O+C Dose Escalation Table....77
Table 10. Dose Escalation Decisions....77
Table 11. Management of Hematologic Adverse Events....83
Table 12. General Management of Adverse Events (Non-Hematologic)....86
Table 13: Hypertension Monitoring and Management....87
Table 14. Management and Monitoring of Decreased LVEF....90
Table 15. Management of Proteinuria....91
Table 16. Management of Infusion-Related Reactions....93
Table 17. Immune-related Adverse Events (Overall Management)....94
Table 18. Pneumonitis/Interstitial Lung Disease (ILD)....95
Table 19. Diarrhea and enterocolitis....97
Table 20. Hepatitis (Elevated LFTs)....99
Table 21. Rash (excluding Bullous skin formations)....101
Table 22. Endocrinopathy (e.g., hyperthyroidism, hypothyroidism, hypopituitarism, adrenal insufficiency, etc.)....103
Table 23. Immune mediated Neurotoxicity (except Myasthenia Gravis and Guillain-Barré)....105
Table 24. Immune-mediated peripheral neuromotor syndromes, such as Guillain-Barre and Myasthenia Gravis....106
Table 25. Correlative studies for Phase II Ovarian cancer cohort 1....118
Table 26. Correlative studies for Phase II TNBC cohort 5....118
Table 27. Correlative studies for Phase II NSCLC (cohort 2) and SCLC (cohort 3)....119
Table 28. Correlative studies for Phase II Colorectal cancer cohort 6....120

Table 29. Evaluation of Best Overall Response for Patients with Measurable Disease (i.e., Target Disease)....139

Table 30. Evaluation of Best Overall Response for Patients with Non-Measurable Disease (i.e., Non-Target Disease)....140

Table 31 Frequency of ADRs for Durvalumab Monotherapy From Pooled Data Across Multiple Tumour Types (N=3006) 166

STATEMENT OF COMPLIANCE

The trial will be carried out in accordance with International Conference on Harmonisation Good Clinical Practice (ICH GCP) and the following:

- United States (US) Code of Federal Regulations (CFR) applicable to clinical studies (45 CFR Part 46, 21 CFR Part 50, 21 CFR Part 56, 21 CFR Part 312, and/or 21 CFR Part 812)

National Institutes of Health (NIH)-funded investigators and clinical trial site staff who are responsible for the conduct, management, or oversight of NIH-funded clinical trials have completed Human Subjects Protection and ICH GCP Training.

The protocol, informed consent form(s), recruitment materials, and all participant materials will be submitted to the Institutional Review Board (IRB) for review and approval. Approval of both the protocol and the consent form must be obtained before any participant is enrolled. Any amendment to the protocol will require review and approval by the IRB before the changes are implemented to the study. In addition, all changes to the consent form will be IRB-approved; an IRB determination will be made regarding whether a new consent needs to be obtained from participants who provided consent, using a previously approved consent form.

1 INTRODUCTION

1.1 STUDY OBJECTIVES

1.1.1 Phase I of doublet treatment: Primary objective

- To establish recommended phase II doses (RP2D) of doublet therapies, Durvalumab/olaparib (Durvalumab+O) and Durvalumab/cediranib (Durvalumab+C), in patients with advanced solid tumors.

1.1.2 Phase I of doublet treatment: Secondary objectives

- To determine the safety of the doublets, Durvalumab+O and Durvalumab+C.
- To determine preliminary response rates of the doublets using RECIST v1.1.
- To determine the pharmacokinetics of the doublets and correlate with safety.
- To explore changes in peripheral immune subsets, plasma cytokines and circulating endothelial cells with safety and/or clinical outcome of Durvalumab+C.
- To determine the potential relationship between PD-L1 expression obtained from archival tissue samples and clinical response.

1.1.3 Phase I of triplet treatment: Primary objective

- To establish recommended phase II doses (RP2D) of triplet therapy, Durvalumab/olaparib/cediranib (Durvalumab+O+C) in patients with advanced solid tumors.

1.1.4 Phase I of triplet treatment: Secondary objectives

- To determine the safety of Durvalumab+O+C.
- To determine preliminary response rates of Durvalumab+O+C using RECIST v1.1.
- To determine the pharmacokinetics of the triplet and correlate with safety.
- To explore changes in peripheral immune subsets, plasma cytokines and circulating endothelial cells with safety and/or clinical outcome of Durvalumab+O+C.
- To determine the potential relationship between PD-L1 expression obtained from archival tissue samples and clinical response.

1.1.5 Phase II Cohorts 1-6 Primary objective

- Phase II Cohort 1 recurrent ovarian cancer (OvCa); Durvalumab+O, Durvalumab+C and Durvalumab+O+C arms: To determine clinical efficacy as measured by overall response rate (ORR) using RECIST v1.1.
- Phase II Cohort 2 non-small cell lung cancer (NSCLC); Durvalumab+O and Durvalumab+C arms: To determine clinical efficacy as measured by progression-free survival (PFS)
- Phase II Cohort 3 small cell lung cancer (SCLC); Durvalumab+O arm: To determine clinical efficacy as measured by ORR
- Phase II Cohort 4 metastatic castrate-resistant prostate cancer (mCRPC); Durvalumab+O arm: To determine clinical efficacy as measured by PFS
- Phase II Cohort 5 triple negative breast cancer (TNBC); Durvalumab+O arm: To determine clinical efficacy as measured by ORR
- Phase II Cohort 2 Cohort 6 colorectal cancer (CRC): C+Durvalumab arm: To determine clinical efficacy as measured by PFS

1.1.6 Phase II Cohorts 1-6 Secondary objectives

- Phase II Cohort 1 OvCa; Durvalumab+O, Durvalumab+C and Durvalumab+O+C arms: To evaluate PFS, safety by CTCAE v4.0, and potential relationship between pretreatment tumor PD-L1 expression obtained from biopsies and clinical response
- Phase II Cohort 2 NSCLC; Durvalumab+O and Durvalumab+C arms: To determine ORR, and safety by CTCAE v4.0
- Phase II Cohort 3 SCLC; MEDI+O arm: To determine PFS and safety by CTCAE v4.0
- Phase II Cohort 4 mCRPC; Durvalumab+O arm: To determine ORR, safety by CTCAE v4.0, duration of response and PSA responses.

- Phase II Cohort 5 TNBC; Durvalumab+O arm: To determine PFS, safety by CTCAE v4.0, and potential relationship between pretreatment tumor PD-L1 expression obtained from biopsies and clinical response
- Phase II Cohort 6 CRC; C+Durvalumab arm: To determine clinical efficacy as measured by ORR, safety by CTCAE v4.0,

1.1.7 Translational and Exploratory Objectives (Phase II)

- To explore tumor mutational loads, tumor or peripheral immune characteristics changes, or cell free DNA in response to treatment.
- To explore changes in plasma cytokines and correlate with clinical response.
- To correlate levels of circulating endothelial cells with clinical outcome.
- To correlate levels of circulating tumor cells (CTCs) with clinical outcome.
- To explore the correlation of immune-related response criteria with standard RECIST criteria.
- OvCa and TNBC only: To examine differential immune characteristics of gBRCAm carriers and explore if they predict susceptibility to treatment.
- OvCa only: To explore whether a previously defined immunoreactive subtype of OvCa, as identified by TCGA and AOCS, correlates with clinical response.
- OvCa only: To explore the correlation of baseline activity or changes of OvCa tumor NF-kB, with clinical benefit.
- CRC only: To evaluate tumor PDL1 expression relative to baseline following single agent cediranib.
- To explore the correlation of radiomics changes with clinical outcome.

1.2 Background and Rationale

High grade serous epithelial ovarian cancers are of Mullerian duct origin, are genetically unstable and aggressive neoplasms, and account for the majority of deaths in the United States in women with gynecologic malignancies[1]. Most patients with epithelial ovarian cancer present with advanced disease at diagnosis[2]. Disease recurrence is common, leading to incurable disease with limited treatment options[2].

1.2.1 Disruption of the PD-1/PD-L1 pathway as a therapeutic approach in advanced ovarian cancer

A potential therapeutic target in advanced solid tumors and recurrent ovarian cancers is the modulation of the host immune response to tumor[3-5]. There is recent evidence that native host anti-tumor cell mediated immune mechanisms play a role in clinical outcomes of epithelial ovarian cancer[6,7]. Tumor cells create an immunosuppressive milieu with activation of multiple mechanisms to evade immune destruction, including disruption of effective antigen presentation, reduction of effector T cell function, and upregulation of pathways that promote tolerance and T cell anergy[7].

Multiple studies showed that antitumor immunity, including the presence of CD3+ and CD8+ lymphocytes within the tumor microenvironment, was associated with subsequent clinical benefit in a subset of patients with advanced ovarian cancer[8]. The presence of intra-tumoral CD3+ T cells was predictive of clinical outcome, and correlated with prolonged overall survival in advanced ovarian cancer[6,9,13]. Immunosuppressive cells, specifically, regulatory T cells (Tregs) and myeloid-derived suppressor cells (MDSCs), down-regulate anti-tumor immunity[10]. Increased Treg infiltration is associated with poor prognosis, and high grade, advanced stage disease in ovarian cancer patients[11-13]. MDSCs are a heterogeneous family of myeloid cells that suppress T cell immunity in tumor-bearing hosts and promote cancer cell proliferation, epithelial-mesenchymal transition, and dissemination in preclinical models[14,15]. MDSCs have a role in immune suppression of ovarian cancer in mouse models. However, their relevance in human ovarian cancers needs to be studied[16].

Blocking tumor-mediated immunosuppression, thus enhancing anti-tumor immunity, is a rational therapeutic approach. The programmed death (PD) -1/ PD-ligand-1 (PD-L1) pathway is a critical component of tumor-mediated immunosuppression. It suppresses immune activation and the expansion of antigen-specific T cells[17]. These immune checkpoints normally promote tolerance and prevention of collateral host tissue damage during immune responses[17]. Upregulation of the PD-1/PD-L1 axis results in an exhausted T cell phenotype that allows disease growth and disruption of cytotoxic T lymphocyte (CTL)-mediated killing of tumor targets[18]. PD-1 has two known ligands, PD-L1 (B7-H1, CD274),[19,20] and PD-L2 (B7-DC, CD273)[21,22]. PD-L1 expression is upregulated on tumor cells, antigen-presenting cells, and dendritic cells; whereas, PD-L2 expression is limited to hematopoietic and endothelial cells. Nearly all human tumor-reactive tumor-infiltrating lymphocytes (TILs) in epithelial ovarian cancer express high levels of PD-1[23], requiring signaling through its ligands to suppress T cell function (**Figure 1**)[4].

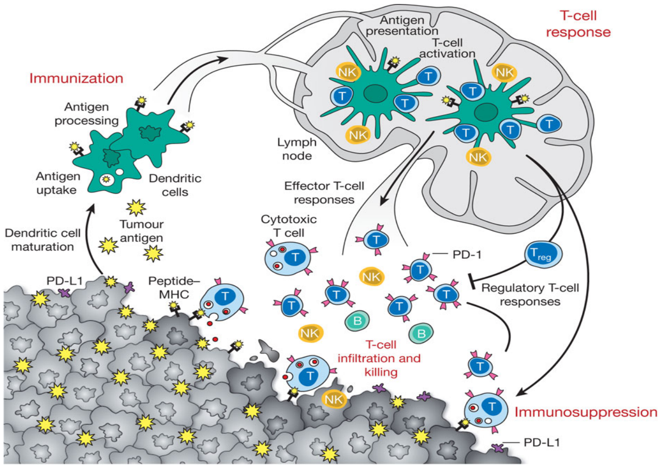

Figure 1. Immunosuppression by the PD-1/PD-L1 pathway activation

Interaction of PD-1 with PD-L1 suppresses T cell responses, and may promote differentiation into Tregs. Antigen-educated T cells exit the lymph node and enter the tumor bed, where a host of immunosuppressive defense mechanism can be produced by tumors that oppose effector T cell function; Figure reproduced from Melman et al.[4].

PD-L1 expressed on tumor cells from epithelial ovarian cancers inhibits anti-cancer immunity[19]. In a preclinical model, PD-L1 bearing ovarian tumors inhibited tumor-directed cytotoxic T cell activity[24]. Furthermore, blocking the interaction of PD-L1 with PD-1 on T cells has resulted in tumor regression in a mouse epithelial ovarian cancer model[24]. Maine *et al.* showed that PD-L1 was over-expressed on monocytes within ascites of patients with ovarian cancer; they demonstrated functional links between PD-L1 expression on those monocytes and tumor cells[25]. Hamanishi *et al.* reported PD-L1 and PD-L2 expression in human ovarian cancers is associated with poor survival and that expression of PD-L1 was inversely related to the presence of CD8+ suppressor T cells in tumor islets[26]. Thus, disruption of the PD-1/PD-L1 interaction is a rational strategy for immunotherapy in patients with advanced epithelial ovarian cancer.

Recently, Brahmer *et al.* presented the first in human study of anti-PD-L1 antibody (BMS-936559) in patients with advanced malignancies[27]. Treatment was well tolerated in the 207 patients treated for a median of 12 weeks. Treatment related grade 3-4 adverse events occurred in only 9% of patients. Immune-mediated adverse events, including rash, hypothyroidism, and hepatitis, were predominantly mild, although 9 patients required transient administration of glucocorticoids. Treatment responses were observed in 9 out of 52 patients with melanoma, 5 of 49 patients with non-small cell lung cancer, 2 of 17 patients with renal cancer, and 1 of 17 patients with ovarian cancer.

A phase II study of anti-PD1 antibody (nivolumab, BMS-936558) in recurrent platinum-resistant ovarian cancer was reported at 2014 ASCO[28]. Eighteen platinum-resistant ovarian cancer patients were treated with nivolumab on two dose cohorts; 1 mg/kg (10 patients) and 3 mg/kg (8 patients), administered every 2 weeks for up to 1 year. More than half of the patients received > 4 prior treatment regimens. Median treatment duration was 14 weeks and objective response rate (ORR) by RECIST was 17% (3/18). Two patients in the 3 mg/kg group experienced complete response [29], one with serous epithelial ovarian cancer and another with clear cell ovarian cancer. One experienced a partial response (PR) and two patients experienced stable disease (SD) in the 1 mg/kg group. This result suggests disruption of the PD-1/PD-L1

pathway is a promising direction for recurrent ovarian cancer patients for whom there are limited therapeutic options.

Targeting the PD-1/PD-L1 pathway in combination with angiogenesis inhibition and/or Poly(ADP-ribose) polymerase (PARP) inhibition in recurrent ovarian cancer

Emerging data suggest modulation of other pathways can alter host immune responses, which raises the possibility of innovative therapeutic combinations[30,31]. Active therapeutic targets in recurrent ovarian cancer include angiogenesis and DNA damage repair pathways.

1.2.1.1 Angiogenesis inhibition

Angiogenic factors influence lymphocyte trafficking across endothelia into tumor deposits[32] and suppress inflammation to promote vessel growth[33]. VEGF negatively alters antitumor immune responses in preclinical and clinical models, including the suppression of dendritic cell maturation[34, 35,36], proliferation of Tregs, inhibition of T cell responses[37], and accumulation of MDSCs[38-40]. VEGF suppresses lymphocyte trafficking across endothelia into tumor deposits and sites of inflammation to promote vessel growth[32,33]. In a metastatic renal cell cancer phase I study, both nivolumab with sunitinib or with pazopanib yielded an approximately 50 % RR[41]. CTLA4 blockade with ipilimumab combined with bevacizumab was reported as an active and tolerable combination in metastatic melanoma patients[42]. There, the combination significantly increased trafficking of $CD8^+$ T cells and $CD163^+$ dendritic macrophages across the tumor vasculature compared with ipilimumab alone. These results indicate that angiogenesis inhibition may promote antitumor immune response, two targets important in recurrent ovarian cancer, making such a combination an important new direction.

Angiogenesis inhibition has demonstrated therapeutic potential in ovarian cancer. Bevacizumab contributes to PFS in both front-line[43,44] and recurrent settings[45]. Cediranib, an oral VEGFR1-3 tyrosine kinase inhibitor[42], also is active in recurrent ovarian cancer as monotherapy or in combination with chemotherapy[46,47 48]. Its adverse events are generally class effects including diarrhea, fatigue, hypertension, hoarseness, hand-foot syndrome, and uncommonly, nausea and vomiting[46]. Diarrhea is the most common adverse event (AE, 91% all grade), predominantly grade 1 or 2[46]. Two phase II studies of cediranib (30 mg daily) in recurrent ovarian or peritoneal or fallopian tube cancer demonstrated a clinical benefit rate of up to 30% (CBR; defined as CR, PR, SD $\geq$ 16 weeks, or CA-125 non-progression >16 weeks) and PFS of 4.1 and 5.2 months, respectively. Overall, eight patients (17 %) had a PR, and six patients (13 %) had SD[46]. ICON6 is a randomized 3-arm, double-blind, placebo-controlled phase III trial for first relapse of platinum-sensitive ovarian cancer. Cediranib was added to platinum-based chemotherapy in 2 of 3 arms, one of which also had cediranib maintenance. The combination/maintenance resulted in a significantly improved PFS of 11.1 months in the cediranib maintenance arm compared to 8.7 months in the chemotherapy arm (hazard ratio [HR], 0.57; $p < 0.001$, **Figure 2A**). Median OS was 26.3 months in the cediranib maintenance arm compared to 20.3 months in the chemotherapy arm (HR, 0.70; $p = 0.04$, **Figure 2B**)[47].

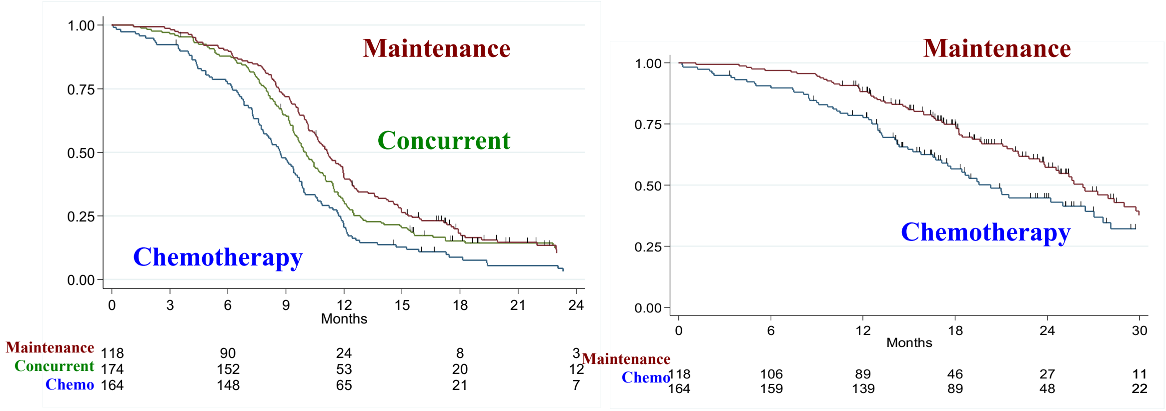

A. **B.**

Figure 2. Combination therapy followed by cediranib maintenance led to improved PFS (A) and overall survival (B) in patients with platinum-sensitive recurrent ovarian cancer[38].

1.2.1.2 PARP inhibition

Preclinical studies have demonstrated promotion of local antigen release and systemic anti-tumor responses after tumor exposure to radiation or DNA damaging agents[30,49-51]. Recently, Snyder *et al*. reported mutational load by exome sequencing was associated with clinical benefit in malignant melanoma patients treated with anti-CTLA-4 antibodies (p=0.01)[52]. Candidate tumor neoantigens were identified, using genome-wide somatic neoepitope analysis and patient-specific HLA typing; a neoantigen was specifically identified in tumors with a strong response to CTLA-4 blockade[52]. PARP-1 mediates DNA damage repair mechanisms by supporting repair of single stranded DNA breaks through the base excision repair pathway[28,29]. Increased DNA damage by PARPi would thus be expected to further the antigenic microenvironment[53].

PARPi are a recognized active drug class for recurrent ovarian cancer with activity in germline BRCA mutation (gBRCAm) -associated and sporadic high-grade serous ovarian cancers[54,55]. Women with gBRCAm-associated ovarian cancer have marked tumor genomic instability, a feature that can increase antigen quantity and exposure leading to activation of the immune response[56,5]. It is plausible to speculate that increased DNA damage by PARPi may contribute to further genomic instability, activation of immune response, and thus the favorable clinical outcome in this population.

Olaparib (Lynparza™), a recently FDA approved PARPi for gBRCAm-related advanced ovarian cancer, is well tolerated at doses of 400 mg twice daily in capsule formulation[54,57]. Class-based AEs include bone marrow toxicity, fatigue, headache, and nausea in 25-40% of patients, predominantly grade 1 or 2. Olaparib monotherapy demonstrated clinical activity in both women with gBRCAm-related and BRCA wild type (BRCAwt)-ovarian cancers, with reported RRs of ~40 % in gBRCAm and 24 % of BRCAwt patients[58 57]. Data also indicate that RR and duration of response to PARPi monotherapy are associated with platinum sensitivity[59 60]. Olaparib is being examined in combination with a variety of cytotoxic chemotherapies. Our investigator-initiated phase I study of olaparib and carboplatin in gBRCAm-associated recurrent ovarian cancer (NCT01445418) yielded a RR of 52 % with a median PFS of 12 months (6-45⁺

months)[61]. This suggests unrecognized opportunities for olaparib combinations in women with recurrent ovarian cancer, a next step to consider upon completion of the present study.

1.2.1.3 The combination of olaparib and cediranib in recurrent ovarian cancer

Recently, we reported the combination of olaparib with cediranib, was clinically superior to olaparib monotherapy in platinum-sensitive recurrent ovarian cancer patients (median PFS 17.7 vs. 9.0 months; HR=0.42, p=0.005, **Figure 3**)[62]. Furthermore, there was a marked increase in PFS from 5.7 months with olaparib alone to 17.7 months with combination therapy in patients with BRCAwt or unknown BRCA status (p=0.003). This unexpected anti-tumor activity of the combination led to development of a multi-institutional randomized phase II/III studies of olaparib/cediranib compared with standard chemotherapy in recurrent platinum-resistant ovarian cancer.

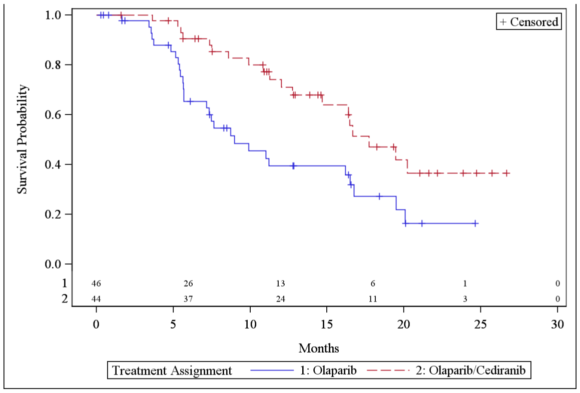

Figure 3. Combination olaparib and cediranib [3] is clinically superior to olaparib alone (blue) in platinum sensitive recurrent ovarian cancer.

There are preclinical studies demonstrating an interaction between hypoxia and inhibition of DNA damage repair[63,64]. Our preliminary work showed a more than additive inhibition of ovarian cancer cell invasion and microvascular endothelial cell tube formation *in vitro* by olaparib and cediranib (unpublished data). We hypothesized assessment of vascular and DNA repair endpoints within the olaparib/cediranib study would identify lead biomarker candidates. Blood samples were collected pre- and day 3 of therapy to measure circulating endothelial cells (CEC: nucleated CD133^{-}CD146^{+}CD31^{+}CD45^{-}), circulating endothelial progenitor cells (CEP: viable nucleated CD133^{+}, CD146^{-}, CD31^{+} CD45^{-} or dim), plasma for cytokine concentrations of IL-6, IL-8, VEGF, and sVEGFR-2. In a test set of patients for the correlative endpoints, women on combination therapy had a median 350 % increase in circulating endothelial cells (CEC: nucleated CD133^{-}CD146^{+}CD31^{+}CD45^{-}) compared to olaparib alone (p=0.01; Lee et al., manuscript submitted; **Figure 4A**). The fold increase in CEC on day 3 was associated with PFS in all patients (R^2=0.77, 95%CI 0.55-0.97, p<0.001; **Figure 4B**). A greater decrease in IL-8 was also noted in patients on combination compared to olaparib alone (p=0.026).

Significant changes of IL-8 concentration and CEC number with olaparib/cediranib suggest greater inhibition in angiogenesis compared with olaparib. It is plausible to speculate that hypoxic condition by cediranib may result in increased DNA damage[65], facilitating induction of cell death by olaparib. Further studies of this combination with prospectively planned validation

of these potential predictive biomarkers are warranted to focus therapy to recurrent ovarian cancer patients who may best respond.

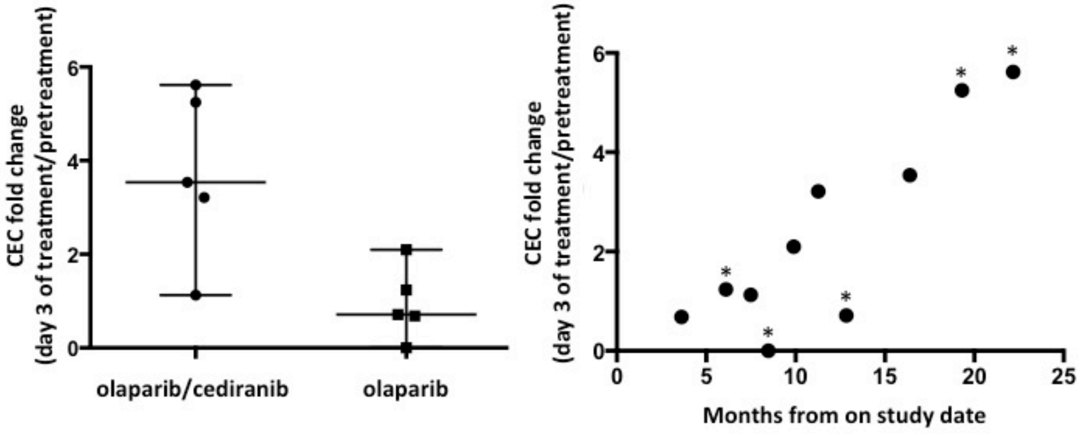

A. **B.**

Figure 4. Patients receiving olaparib/cediranib had a larger fold increase median increase in CEC compared to olaparib alone (A) and the fold increase was associated with PFS (B). * represents patients whose times at risk were censored on the date of their last contact and were last reported alive and progression-free.

1.2.1.4 Immune system modulation by targeted agents and in combination with immune checkpoint blockade

Emerging data further elucidates the molecular mechanisms underlying the immunogenic effects of tyrosine kinase inhibitors[30] and VEGF has been shown to negatively alter antitumor immune responses in preclinical and clinical models, including the proliferation of Tregs, inhibition of T cell responses[37], and accumulation of MDSCs[38-40]. VEGFR tyrosine kinase inhibitors such as sunitinib have been associated with decreased levels of infiltrating Treg cells and MDSCs in patients with renal cell carcinoma (RCC)[66-69], and facilitate the development of antitumor T_{H1} responses[67]. These results indicate that VEGFR inhibitors may promote antitumor immune response in recurrent ovarian cancers[46,57] and suggest that combination with a PD-L1 inhibitor a logical combination for examination.

Nivolumab in combination with sunitinib or pazopanib is currently under evaluation in metastatic clear-cell RCC patients who have received prior antiangiogenic therapy (NCT01472081). Preliminary results of this phase I study were reported at 2014 ASCO. Sunitinib (50 mg, 4 weeks on, 2 weeks off) or pazopanib (800 mg daily) was administered with nivolumab at 2 mg/kg or 5 mg/kg IV every 3 weeks. Thirty-three patients were treated with sunitinib/nivolumab (S/N; 7 patients on 2 mg/kg of N and 26 patients on 5 mg/kg of N), no dose-limiting toxicities (DLTs) were observed. There were 4 DLTs in 20 patients treated with pazopanib/nivolumab (P/N) at 2 mg/kg (elevated ALT/AST [n=3], fatigue [n=1]), leading to closure of this arm. This indicates that combination of some tyrosine kinase inhibitors with a PD-

1 inhibitor may have increased toxicity. Most common related grade 3 or 4 AEs included elevated ALT (18 %), hypertension and hyponatremia (15 % each) for S/N, and elevated ALT/AST (20 % each) and fatigue (15 %) for P/N. ORR was 52 % (17/33) in S/N arm and 45 % (9/20) in P/N arm. These findings provide a basis for further investigating the combination of immune checkpoint blockade and VEGFR tyrosine kinase inhibitors in advanced solid tumors.

There are limited data on DNA damaging agents such as platinums, and their immune modulation, warranting further evaluation. Lesterhuis *et al.* reported that patients who were treated with weekly cisplatin in combination with radiotherapy had markedly reduced STAT6-regulated expression of PD-L2 on both dendritic cells and tumor cells[70]. Our group has begun studying olaparib and its immune modulation in ovarian cancer patients. Further work is ongoing in collaboration with Dr. Matulonis at Dana Farber Cancer Institute. Our recent success combining inhibition of angiogenesis with modulation of DNA repair with cediranib and olaparib provides a baseline upon which to build for a better therapeutic combination. Both pathways have been linked to the immune system and can activate the immune system in a fashion that ultimately leads to immune exhaustion. Combination therapy with a PD-L1 inhibitor and olaparib and/or cediranib has not been studied to date, and will be an important question to guide patient- and resource-efficient use of this important, novel drug class.

1.2.2 Background and Rationale of the PD-1/PD-L1 pathway inhibition in combination with a PARP inhibitor or VEGFR inhibition in lung, prostate, triple negative breast, and colorectal cancers

1.2.2.1 Non-small cell lung cancer cohort

Non-small cell lung cancer (NSCLC) is a devastating disease with more than 200,000 cases diagnosed annually and 159,000 deaths related to the disease.[71] Standard therapy for advanced NSCLC is based on platinum-based chemotherapy which is associated with a RR of 30% and median OS of about 12 months.[72] The identification of molecular subsets of NSCLC and use of small molecular inhibitors to target specific genomic aberrations has resulted in significant improvements in RRs and survival.[73] Yet, significant challenges need to be addressed including the presence of mutations that cannot presently be targeted therapeutically, development of resistance to targeted therapy and the occurrence of tumor heterogeneity.[73]

Within the past few years the development of immune checkpoint inhibitors, especially drugs targeting the PD-1 or its ligand, PD-L1 has resulted in significant changes in the therapeutic landscape for advanced NSCLC.[74] These treatments are relatively well-tolerated and associated with durable responses and improvements in survival rates.[75,76] The anti-PD-1 antibodies, nivolumab and pembrolizumab are now FDA-approved for treatment of recurrent NSCLC. There is emerging evidence that illustrates the impact of various biological variables on the efficacy of immune checkpoint inhibition. These factors include PD-L1 expression on tumor cells, the mutational burden of the underlying tumor and the tumor microenvironment.[77,78] Furthermore, resistance to immunotherapy emerges eventually resulting in disease progression. In an attempt to increase RRs and delay the onset of resistance immune checkpoint inhibitors are currently being evaluated in combination with other drugs known to be active against NSCLC including chemotherapy, molecular-targeted therapy and other forms of immune therapy.

A subset of NSCLCs has been found to have defects in HR DNA DSB repair, which can potentially sensitize them to PARPi.[79] Several PARPi have been evaluated in patients with NSCLC including olaparib and veliparib.[80] Deficient DNA damage repair in the presence of a PARPi can be expected to increase the number of tumor antigens against which an immune response can be mounted.[53] Although not all antigens are immunologically relevant, the presence of specific epitopes (rejection antigens) can modulate the process of tumor rejection and increase the likelihood that tumors will respond to immune checkpoint inhibition. Newer treatment approaches need to be developed for patients with NSCLC who are not candidates for targeted therapy and do not derive benefit from immune checkpoint inhibition. Thus, the combination of drugs that impair the DNA repair process with immune checkpoint inhibitors is a novel approach that needs to be investigated further in NSCLC.

NSCLCs are also sensitive to antiangiogenic drugs. We plan to evaluate Durvalumab in combination with cediranib due to the potential benefits of targeting angiogenesis. Previous trials have demonstrated the benefits of anti-angiogenic drugs in combination with chemotherapy (bevacizumab in combination with carboplatin and taxol) or when used as single-agents (e.g. multikinase inhibitors with anti-angiogenic properties, such as sorafenib). A meta-analysis of 20 randomized controlled trials involving 10,834 subjects showed that multi-targeted antiangiogenic tyrosine kinase inhibitors improved RR (OR1.29, 95%CI, 1.08 to 1.55, p=0.006) and prolonged PFS (HR 0.83, 95%CI, 0.78 to 0.90, p=0.005).[81] The phase III ZODIAC trial in the second-line setting in patients with advanced NSCLC also demonstrates the benefit of the multi-targeted antiangiogenic tyrosine kinase inhibitor, vandetanib in combination with docetaxel versus docetaxel alone.[82] A significant improvement in PFS was noted in the combination arm compared to docetaxel alone (median PFS 4.0 months versus 3.2 months, HR 0.79, 97.58% CI, 0.70-0.90, p<0.0001). Therefore, immune checkpoint inhibition in combination with VEGFR inhibition will be a new direction to be explored in NSCLC.

1.2.2.2 Small cell lung cancer cohort

Small cell lung cancer (SCLC) is the most aggressive form of lung cancer. It represents 15% of all lung cancers and has an annual incidence of over 34,000 in the United States. Although SCLC is highly responsive to chemotherapy (platinum and etoposide, or topotecan), responses are not durable and it becomes refractory to treatment within a few months.[83] There is a critical need for newer therapeutic approaches.

SCLC is the most frequent cancer histology associated with paraneoplastic syndromes. SCLC associated paraneoplastic syndromes are thought to be related to immune-mediated tissue destruction due to neural antigen expression from cancer cells and are associated with more favorable outcomes.[84] Preclinical studies indicate that a number of immune escape mechanisms are at play in the SCLC tumor microenvironment. SCLC over-express TGF-β; high levels of TGF-β are also detected in the serum of patients with lung cancer compared with normal individuals. [85] Additionally, the expression of MHC antigens is reduced in SCLC, and this may underlie the tumor's ability to escape immunosurveillance .[86]

There are limited data on PD-L1 expression in SCLC. PD-L1 expression in tumor-infiltrating macrophages was correlated with tumor-infiltrating lymphocytes.[87] Expression of PD-L1 in tumor cells was also observed in 71.6% (73 of 102) of SCLCs, and was significantly correlated

with a limited disease stage. In this study, patients with PD-L1-positive tumors showed longer OS than those with PD-L1-negative tumors (median OS, 16.3 vs 7.3 months; $p < 0.001$, respectively). [88]

Recent reports indicate early clinical activity of immunotherapy targeting the PD-1/PD-L1 pathway in patients with relapsed SCLC. Pembrolizumab (10 mg/kg q 2 weeks IV) showed ORR of 35% (95% CI, 15%, 59%) in patients with previously treated, PD-L1-positive, extensive-stage SCLC.[89] Here, PD-L1 expression in defined as expression on ≥ 1% of cells in tumor nests or PD-L1+ bands in stroma by IHC. Responses were durable; median duration of response was 29.1 weeks (range: 0.1-29.1 weeks) and median time to response was 8.6 weeks (range: 7.7-16.1 weeks). Nivolumab also resulted in RR of 18% as monotherapy and 17% in combination with ipilimumab in a study of patients with progressive disease after one or more lines of therapy, including a first-line platinum-based regimen.[90] The disease control rate was 38% with monotherapy and 54% with combination therapy. The median OS was 4.4 months with monotherapy (95% CI, 2.9, 9.4) and 8.2 months with combination therapy (95% CI, 3.7, not reached).

Mutational burden appears to be an important determinant of response to immune checkpoint inhibition. In a recent study analyzing tumor mutational burden in patients with NSCLC treated with the PD-1 antibody pembrolizumab, higher non-synonymous mutation burden in tumors was associated with improved objective response, durable clinical benefit, and PFS. Efficacy also correlated with the molecular smoking signature, higher neoantigen burden, and DNA repair pathway mutations; each factor was also associated with mutation burden.[91] The association between response to PD-1 inhibitors, mutation burden, and tobacco exposure and the high frequency of DNA repair pathway alterations may have important implications for SCLC.

PARP1 is highly expressed at the mRNA and protein levels in SCLCs and SCLC growth is inhibited by PARP1 knockdown. PARPi downregulates key components of the DNA repair machinery and enhances the efficacy of chemotherapy.[92] PARPi monotherapy has shown clinical activity in SCLC patients; in a phase I trial, confirmed responses were reported in 2 of 11 (18%) previously treated SCLC patients with BMN 673.[93] In pre-clinical studies, sensitivity to BMN 673 was associated with elevated baseline expression levels of several DNA repair proteins. [94] PARPi are actively being investigated in combination with chemotherapy (NCT01642251; NCT02289690) and with alkylating agents (NCT01638546) in SCLC. Collectively, these findings support examining the clinical activity of a PARPi and immune checkpoint inhibition in SCLC.

1.2.2.3 Metastatic castrate-resistant prostate cancer

Prostate cancer is the second most common cancer in men in the United States with an estimated 220,800 new cases and 27,540 deaths in 2015.[95] While prostate cancer is initially responsive to androgen deprivation therapy (ADT), the median duration of sensitivity is approximately 24-36 months.[96] While ADT can prolong life, prostate cancer will eventually progress, and a majority of men will go on to develop castration-resistant prostate cancer (CRPC) which is defined as disease progression despite castrate levels of testosterone (<50 ng/dL).[97] In 2004, two landmark studies showed OS benefit in metastatic CRPC (mCRPC) with docetaxel.[98,99] However, patients eventually develop resistance to docetaxel. Over the last several years, the landscape of treatment in mCRPC has heralded new treatment options

including potent hormonal agents abiraterone[100,101] and enzalutamide[102,103] which target the androgen receptor (AR). Both abiraterone and enzalutamide have shown OS benefits both pre- and post-chemotherapy, unfortunately, again men with mCPRC will develop resistance to these agents.[104] Alternate treatment options need to be identified in this patient population.

PARP1 is implicated in androgen receptor (AR) driven gene expression.[105,106] [105] The AR plays a key role in prostate cancer proliferation and progression. In mCRPC, the AR is an important target in therapeutic treatment, but resistance to agents targeting the AR is quite common. Preclinical studies have shown PARP1 inhibitors suppress AR activity and tumor growth in the absence of DNA damaging agents or BRCA alterations.[106] In mCRPC models of disease, PARP1 is recruited to sites of AR transcriptional function, and PARP1 enzymatic activity is required for AR-driven gene expression and cancer cell proliferation.[105] PARP1 inhibition leads to decreased AR activity and is associated with altered capacity of androgen stimulation to elicit pro-transcriptional changes in histone modifications and chromatic architecture.[105] With PARPi, AR activity decreased which led to decrease of tumor growth in vivo and ex vivo. PARP1 has also been implicated in interaction with the TMPRSS2-ERG fusion protein. In more than 50% of prostate cancers, gene fusions occur between TMPRSS2, an AR target gene, and ERG (an ETS transcription factor) which induces cancer cell growth and invasion.[105] The fusion gene product interacts with PARP1 and PARP1 is involved in the transcriptional activation function of ERG. In fusion positive tumor cells, PARP1 inhibition decreases ERG cell invasion and xenograft tumor growth.[105] Thus, PARPi plays a role in the treatment of mCRPC where options become limited over time.

Next generation sequencing (NGS) has identified recurrent mutations and genomic alterations in mCRPC that are clinically actionable. An international consortium of 8 medical center clinical sites conducted prospective whole-exome and trascriptome sequencing of bone or soft tissue tumor biopsies in 150 patients with mCRPC.[104] Biallelic inactivations of *BRCA2*, *BRCA1*, or *ATM* were observed in 19.3% of patients, a much higher frequency in mCRPC when compared to primary prostate cancers. Of note, 19 of 150 cases (12.7%) had loss of *BRCA2* (90% with biallelic loss). A total of 8 affected individuals were found to have pathogenic germline *BRCA2* mutations. Mutations in DNA damage response factors *BRCA2* and *BRCA1*, and *ATM* mutations may sensitize tumor cells to PARPi.[107] The 29/150 patients (19.3%) with aberrations in *BRCA2, BRCA1*, or *ATM* would therefore be predicted to benefit from treatment with PARPi. NGS has also identified genomic aberrations causing DNA repair defects in the TOPARP study, a phase II trial of olaparib in mCRPC.[108] The first stage of the TOPARP-A clinical trial was presented at 2015 AACR. Fifty patients with mCRPC were enrolled and treated with olaparib monotherapy.[109] All patients had been treated previously with docetaxel, 48 patients (96%) had been previously treated with abiraterone, and 20 patients (58%) with cabazitaxel. Among 49 evaluable patients, 16 had a response to olaparib, 6 had radiologic responses as assessed by RECSITv1.1 criteria, 11 patients had a greater than 50% decrease in prostate-specific antigen (PSA) levels.[110] In 30.6% of patients (15/49), NGS revealed homozygous deletions and/or deleterious mutations in DNA repair genes, with the majority in *BRCA2* and *ATM*. In the 7 patients with *BRCA2* loss, 4/7 (57%) were somatic aberrations and 3/7 (43%) were germline. This trial indicates PARPi has antitumor activity in mCRPC.[110] Targeting genetic defects in DNA repair genes in mCRPC offer possibilities for treatment options in heavily pre-treated patients.

T cell based immunotherapy has been utilized in mCRPC for many years. Therapeutic vaccines such as PROSTVAC-VF[111] and sipuleucel-T[112] have shown promise in mCRPC, but new therapeutic approaches in men with mCRPC are needed. Overexpression of PD-L1 has been described in CRPC.[113] PD-L1 is expressed mainly on tumor cells making it a target for blockade. PDL-1 binds PD-1, expressed mainly on T cells, causing T cell suppression and reducing anti-tumor immunity. Blocking this interaction may reverse T cell suppression, allowing for T cell mediated killing of tumor cells. Although PD-L1 has not been found to be highly expressed in prostate tumor samples, both PD-L1 and PD-1 have been found in the prostate cancer microenvironment and appear to play a role in prostate cancer.[113] PD-1 is upregulated on lymphocyte clusters around prostate cancer lesions.[114] Bishop *et al*. investigated whether PD-L1/2, PD-1, and CTLA-4 were upregulated in enzalutamide resistant patients and in a pre-clinical model of enzalutamide resistance.[115] Enzalutamide resistance was associated with high frequency of PD-1 and PD-L1 in tumor and in circulating immune cells. PD-L1/2 and PD-1 were assessed by flow cytometry on DCs and T cells from a small cohort of men with mCRPC who were enzalutamide naïve or men classified as progressing or responding to enzalutamide.[115] There was an increased frequency of PD-L1/2 (DCs) in men progressing on enzalutamide compared to naïve patients (p=0.0037) or responding patients (p=0.0060). PD-L1 expression on tumor cells may be a mechanism of enzalutamide resistance in mCRPC. In a pre-clinical model, enzalutamide resistant xenografts could cause increases in PDL1/2 dendritic cells in tumor bearing mice blood. Results from this study of a small cohort of patients suggest that patients with enzalutamide resistant prostate cancer have targetable expression of the PD-L1 pathway. Thus, disruption of the PD-1/PD-L1 interaction would be a rational strategy for immunotherapy in patients with mCRPC.

The rationale for combination of PARPi and immune checkpoint inhibition is based upon the complementary mechanisms of action of the two therapies. Exploiting the synergy in the treatment of prostate cancer is of therapeutic potential. PARPi plays a role in immune modulation and can potentially complement the anti-tumor activity of an immune checkpoint inhibitor. PARP-1 has been reported to play a role in immune and inflammatory responses independent of DNA damage.[116] PARP expression is involved in T cell development and function.[117] PARP1 has been implicated in the gene transcription in different types of immune cells and PARP deficiency has effects on regulatory T cell differentiation.[118] In PARP-1 knock-out mice, regulatory T cells are increased in the thymus and peripheral lymphatic organs.[116] PARPi may render tumor cells more amenable to T cell mediated immune attack. Thus, there is rational for clinical evaluation of the combination of olaparib and a PD-LI inhibitor in mCRPC.

1.2.2.4 Preliminary data from mCRPC cohort

As of July 2017, our preliminary data shows the combination of durvalumab plus olaparib is well tolerated with activity in an unselected population. Expanding this cohort will enable us to further evaluate mechanism of action of the combination, obtain additional correlative data and assess potential biomarkers of response, and obtain additional response data. To date, 22 patients have enrolled onto the clinical trial. Of those, 19 patients have been on-study > 2 months. Of the 19 patients, median age is 65 years old (range 45-79 years old). The median

PSA is 79.67 ng/mL (3.93-2356 ng/mL). Twelve patients have a Gleason score of >8. Seven patients have bone only disease and 12 patients have bone and soft tissue/visceral disease. Grade 3/4 adverse events include anemia 3/19 (16%), lymphopenia 2/19 (10%), thrombocytopenia, leukopenia, neutropenia, urinary tract infection, hypertension, lung infection, nausea, vomiting, fatigue, tenosynovitis, hearing impairment, syncope, oral mucositis, muscle weakness, muscle cramps, hyperglycemia (diabetes), and colitis [1/19 each (5%)]. No patients have discontinued treatment secondary to toxicities. Eight of 19 patients (42%) on-study > 2 months have PSA declines > 50%. (Figure 1) Six month PFS is 86.7% (95% CI: 56.4-96.5%); 9 month PFS is 57.8% (95% CI: 8.5-88.5%). Median PFS has not been reached.

1.2.2.5 Triple negative breast cancer

Triple-negative breast cancer (TNBC) accounts for 10-20% of all breast cancers, and is defined by the absence of estrogen and progesterone receptors (ER, PR) and the absence of HER2 amplification[119]. Thus, patients with TNBC do not benefit from known targeted therapies to ER or HER2[120]. Standard therapy for TNBC is chemotherapy and, in metastatic patients, ORRs are around 30-35%, with median PFS 4.5-6 months, and median OS around 12 months[121]. Clearly, new approaches to the treatment of TNBC are needed to improve outcomes.

Two recently reported phase I trials with immune checkpoint inhibitors in TNBC demonstrated promising activity in subsets of heavily pretreated patients. Pembrolizumab resulted in an ORR of 25.8% in 27 patients with advanced TNBC[122]. In another phase I trial with 21 TNBC patients, the PD-L1 inhibitor, MPDL3280A (atezolizumab) demonstrated an ORR of 19%[123]. In addition, 85% of patients received ≥4 prior systemic regimens, including taxanes (82%), anthracyclines (78%), carboplatin (41%), and cisplatin (15%). All patients in both trials had tumors expressing PD-L1 by IHC[122,123], although tumor PD-L1 IHC expression had not been proven to be the sole determinant of which tumors respond to PD-1/PD-L1 directed inhibition[124]. Those RRs seen with single-agent treatments, though low, are encouraging considering the high-risk disease and heavily pretreated population of the studies. Combinations of therapy with immune checkpoint inhibitors are thus necessary to increase the number of patients who benefit from these therapies.

TNBCs frequently have alterations in DNA damage-response and repair mechanisms. Approximately 22% of TNBC patients carried germline mutations to a DNA repair gene including *BRCA1* or *BRCA2* and, less frequently, *NBM, CHEK2, FANCM, PALB2* and *ATM* [125-128]. Somatic mutations in DNA repair pathways are also frequent[129]. In addition, epigenetic factors mediate the loss of BRCA1 or BRCA2 expression[130-132], and that of other DNA repair proteins[133,134] in TNBC. Loss of BRCA 1 and 2 has been demonstrated to sensitize tumors to PARPi[135,136]. PARPi have been clinically investigated in TNBC either monotherapy or in combination with chemotherapy. Dose-dependent response has been observed in gBRCAm associated TNBC patients; a RR was higher with 400 mg olaparib capsule monotherapy compared to 100 mg olaparib capsule monotherapy (54% vs. 25%)[137]. Olaparib in combination with paclitaxel has demonstrated an ORR of 37% in TNBC patients, not selected for gBRCAm[138]. This included one CR in a patient with BRCAwt. It has been known that RRs to PARPi are lower in TNBC patients without gBRCAm[139]. In studies from our group (08-C-0092), the combination of olaparib with carboplatin showed an 87.5% ORR (7/8) and a disease control rate of 100% (8/8) in recurrent or metastatic breast cancer patients with gBRCAm, including one

CR and 3 PRs in four patients with TNBC [140]. TNBC patients without gBRCAm treated on the same studies had a ORR of 19% (5/27) and a disease control rate of 48% (13/27; Lee *et al*, manuscript in preparation). Overall PARPi have clinical activity in subsets of TNBC patients with both gBRCAm and BRCAwt although the RRs in TNBC with BRCAwt are considerably lower. Optimal benefit from PARPi in TNBC, especially those with BRCAwt, will require combination therapy. Thus, disruption of the PD-1/PD-L1 interaction is a rational strategy for combination therapy with olaparib in patients with TNBC.

1.2.2.6 Colorectal cancer

The immune system seems to play a role in both the occurrence and the progression of colorectal cancer (CRC). In early, surgically resectable cases, elevated numbers of intratumoral cytotoxic and memory T cells have been shown to correlate with an aggressive phenotype reflected by tumor microinvasive status[141,142]. In advanced disease, however CRC appears to be less immunogenic, as evidenced by the lack of infiltrating lymphocytes with advancing T stage.

VEGF exhibits proangiogenic properties but also has a key role in the induction and maintenance of an immunosuppressive microenvironment, which is one of the explanations for the ineffectiveness of immune checkpoint inhibitors in CRC (with the exception of MSI-hi tumors). VEGF has been shown to inhibit of dendritic cell maturation, result in the accumulation of MDSC and Tregs[143,144]. In addition, VEGF-A modulates expression of PD1 on $CD8^+$ T cells in colorectal cancer-bearing mice[145]. Terme *et al.* showed that VEGF-A could directly induce Treg cell proliferation in a VEGFR2-dependent manner in CRC model (CT26) and in the blood of metastatic CRC patients[37]. Treatment with bevacizumab inhibited Treg accumulation.

Anti-VEGF agents play a prominent role in the management of CRC, suggesting the importance of the VEGF pathway. Cediranib has been shown to be comparable to bevacizumab when combined with chemotherapy in patients with previously treated CRC[146]. Therefore, disruption of the VEGF/VEGFR pathway would be a promising approach for immunotherapy combination in patients with advanced or recurrent CRC.

Collectively, we hypothesize that PARPi and/or VEGF/VEGFR pathway inhibition will enhance the newly observed anti-tumor activity of immune checkpoint inhibition in subsets of ovarian, non-small cell lung, small cell lung, prostate, triple negative breast, and colorectal cancer patients. We believe that it is reasonable and necessary to prospectively elucidate the activity of the two doublets (a PD-L1 inhibitor/olaparib or a PD-L1 inhibitor/cediranib) in these diseases. The data used with the two doublets will be the foundation of a randomized study of the doublets or the triplet therapy in this patient population.

Durvalumab, an anti-PD-L1 inhibitor (Data source: 2015 Investigator's Brochure [IB] v8.0, 2015)

1.2.2.7 Preclinical studies

Durvalumab is a human IgG1κ monoclonal antibody that selectively binds human PD-L1 with high affinity and high specificity. Drug binding to PD-L1 prevents the ligand from binding to PD-1 and CD80, a T cell activation ligand on B cells or monocytes. Durvalumab also contains a mutation engineered within the constant domain of the Ig G1 heavy chain that reduces binding to complement protein C1q and the fragment gamma Fcγ receptor. This mutation is critical for eliminating off-target complement-mediated cytotoxicity and antibody-dependent cell-mediated cytotoxicity (ADCC).

In vitro studies demonstrated that Durvalumab blocked PD-L1, thereby preventing PD-L1-mediated suppression of human T cell activation. The effect of Durvalumab on the growth of human tumor cell lines in sets of 6 nonobese diabetic (NOD)/severe combined immunodeficient (SCID) mice were studied. NOD/SCID mice were engrafted subcutaneously on day 1 with human pancreatic adenocarcinoma (HPAC) tumor cell lines alone or mixed with alloreactive human CD4+ and CD8+ T cell lines. Durvalumab and mutant IgG isotype control antibody (CAT254) were administered intra-peritoneally to animals inoculated with HPAC and human T cells, 1 hour after the implantation and on days 3, 5, 8, and 10. Tumor growth is shown in **Figure 5**, with a significant inhibition of HPAC growth in a dose-dependent fashion, up to 74% compared to CAT254.

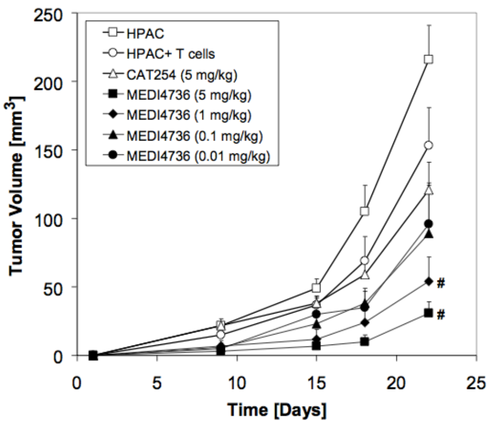

Figure 5. Effect of Durvalumab on the growth of human pancreatic adenocarcinoma tumor cells in a mouse model containing human T cells

#: Tumor growth inhibition > 50%, p<0.05, compared to the isotype-control group.

An anti-mouse PD-L1 antibody demonstrated improved survival in the syngeneic CT26 colorectal tumor model when given as monotherapy, and further, resulted in complete tumor regression in >50% of treated mice when given in combination with oxaliplatin. Responsive mice remained tumor-free for 3 months post-study (**Figure 6**).

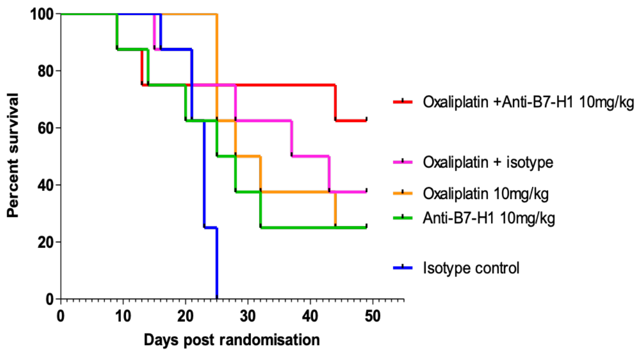

Figure 6. Augmented survival by the combination of anti-mouse PD-L1 antibody (anti-B7-H1) and oxaliplatin compared to single agent in CT26 colorectal *in vivo* model

1.2.2.8 Clinical Studies with Durvalumab

Durvalumab has been administered as monotherapy or in combination to a total of 1,883 patients in 30 clinical studies as of July 12, 2015. Of the 1,883 subjects, 1,279 received Durvalumab monotherapy (**Table 1**), 440 received Durvalumab in combination with tremelimumab or other anticancer agents, 14 received other agents (1 gefitinib, 13 MEDI6383), and 150 have been treated with blinded investigational product. (**Table 2**). No studies have been completed or terminated prematurely due to toxicity. See the 2015 investigator's brochure (IB) for the comprehensive list of the studies.

Table 1. Durvalumab Monotherapy Clinical Studies (2015 IB)

Study	Patients	Dose	Number of patients treated as of May 2015
Phase 1/2 Dose Escalation and Dose Expansion in Subjects with Advanced Solid Tumors: Durvalumab monotherapy	Advanced solid tumors;<br>Multi-arm expansion cohorts: Triple-negative breast cancer, Nonsquamous NSCLC, squamous NSCLC, hepatocellular carcinoma, pancreatic adenocarcinoma, gastroesophageal cancer, advanced cutaneous melanoma, uveal melanoma, squamous cell cancer of the head and neck, and other	• mg/kg Q2W<br>• mg/kg Q2W<br>1 mg/kg Q2W<br>3 mg/kg Q2W<br>10 mg/kg Q2W<br>15 mg/kg Q3W<br>20 mg/kg Q4W	750
Phase 1 Dose Escalation and Dose Expansion: Durvalumab monotherapy	Japanese patients with Advanced Solid Tumors	1 mg/kg Q2W<br>3 mg/kg Q2W<br>10 mg/kg Q2W<br>15 mg/kg Q3W<br>20 mg/kg Q4W	70
Phase 1 Dose Escalation and Dose Expansion: DurvalumabMonotherapy	Myelodysplastic Syndrome	10 mg/kg Q2W	32
ATLANTIC[a] Phase 2<br>Open Label: Durvalumab Monotherapy	NSCLC	10 mg/kg Q2W	303

Study	Patients	Dose	Number of patients treated as of May 2015
HWAK Phase 2 Durvalumab Monotherapy	Recurrent or metastatic PD-L1 positive squamous cell cancer of the head and neck	10 mg/kg Q2W	8
PACIFIC[b] Phase 3 Randomized: Durvalumab monotherapy	Advanced stage refractory squamous cell carcinoma of the lung	10 mg/kg Q2W	115

NSCLC = non-small cell lung cancer; Q2W = every 2 weeks; Q3W = every 3 weeks.

[a] ATLANTIC study: A Global Study to Assess the Effects of MEDI4736 in Patients with Locally Advanced or Metastatic Non Small Cell Lung Cancer

[b]PACIFIC study: A Global Study to Assess the Effects of MEDI4736 Following Concurrent Chemoradiation in Patients with Stage III Unresectable Non-Small Cell Lung Cancer

Table 2. Durvalumab Combination Therapy Clinical Studies (2015 IB)*

Study	Patients	Dose	Number of patients treated as of May 2015
Durvalumab + Dabrafenib (B-raf inhibitor) and/or Trametinib (MEK1/2 inhibitor): Durvalumab Combination Therapy	Metastatic Melanoma	3 mg/kg Durvalumab Q2W + 150 mg dabrafenib + 2 mg trametinib<br>10 mg/kg Durvalumab Q2W + 150 mg dabrafenib + 2 mg trametinib<br>concomitant 10 mg/kg Durvalumab Q2W and 2 mg trametinib)<br>sequential 10 mg/kg Durvalumab Q2W and 2 mg trametinib	65

Study	Patients	Dose	Number of patients treated as of May 2015
Phase Ib: Durvalumab + Tremelimumab Combination Therapy	NSCLC	Durvalumab 10 mg/kg Q2W or Durvalumab 20 mg/kg Q4W + tremelimumab Q4W	102
Phase I: Durvalumab+gefitinib Combination Therapy	NSCLC	Durvalumab Q2W + gefitinib	33
Phase I: Durvalumab+MEDI0680 Combination Therapy	Advanced malignancies	Durvalumab Q2W + MEDI0680 (AMP-514) Q2W or MEDI0680 (AMP-514) Q2W Q4W	21
Phase I: Durvalumab +Tremelimumab Combination Therapy	Advanced solid tumors	Durvalumab Q2W + tremelimumab Q4W	51

NSCLC = non-small cell lung cancer; Q2W = every 2 weeks; Q3W = every 3 weeks; Q4W = every 4 weeks.

* See the 2015 IB for the comprehensive list of the combination studies

1.2.2.9 Durvalumab Clinical activity

As of July 2015, the preliminary activity data are available in 456 of 694 patients with advanced solid tumors, treated with Durvalumab at 10 mg/kg q2 weeks. In PD-L1 unselected patients, the ORR based on RECIST 1.1 ranged form 0% in uveal melanoma to 20% in bladder cancer. PD-L1 status was known for 383 of 456 response evaluable patients. Across the PD-L1 positive tumors, ORR was highest for bladder cancer, advanced cutaneous melanoma and hepatocellular cancer (33.3% each), non-small cell lung cancer (26.7%) and squamous cell carcinoma in head and neck (18.2%). These results are consistent with reported ORR of other PD-1 inhibitors[147,148]. Women with recurrent or refractory ovarian cancer are currently being enrolled into one cohort of an ongoing phase I trial with Durvalumab (NCT01693562). In one combination therapy trial with Durvalumab and tremelimumab, 38 % (5/13 evaluable) of patients had PR.

1.2.2.10 Durvalumab Safety

As of July 2015, 1883 patients were treated with either Durvalumab monotherapy (1279 patients) or in combination with tremelimumab or other anticancer agents (440 patients). Important potential risks based on the mechanism of action of Durvalumab and its related molecules include immune-mediated reactions such as enterocolitis, dermatitis, hepatitis/hepatotoxicity, endocrinopathy, pneumonitis, and neuropathy. Additional important potential risks include infusion-related reactions, hypersensitivity, serious allergic reactions, serious infections, and immune complex disease.

The majority of the safety data are from the monotherapy study (NCT01693562), specifically the 10 mg/kg q2 weeks cohort (N = 694). In this cohort, patients were exposed to a median of 6 doses of Durvalumab, ranging from 1 to 27 doses; the most frequently reported (>10 % of subjects) AEs (all grades, regardless of causality) were fatigue (35.4 %), nausea (25.4 %), dyspnea (24.1 %), decreased appetite (23.8%), cough (18.7%), constipation, diarrhea and vomiting (17 % each), pyrexia and back pain (14 % each), and arthralgia (12 %). In approximately half of the patients, the highest AE severity was grade 1 or grade 2. Most of these events were managed clinically without the need for dose modifications or delays.

Serious adverse events (SAEs, regardless of causality) occurred in 345 of 694 subjects treated with Durvalumab 10 mg/kg q2 weeks in the monotherapy study (NCT01693562). The most frequently reported SAEs (regardless of causality; > 10 subjects) were dyspnea, pneumonia, sepsis, abdominal pain, pleural effusion, and dehydration. 25 patients (3.6%) had SAEs considered to be related to Durvalumab. Durvalumab-related SAEs in > 2 patients were colitis and pneumonitis (3 patients each). A majority of the treatment-related SAEs were grade 3 or higher in severity and recovered with or without sequelae. One patient died due to pneumonia considered to be related to Durvalumab. Before this fatal event of pneumonia, this patient experienced treatment-related to grade 4 pneumonitis and grade 3 pneumonia. A total of 76 patients (11%) treated with 10 mg/kg q2 weeks permanently discontinued of Durvalumab due to AEs; events that resulted in discontinuation of Durvalumab in > 2 subjects were general physical health deterioration (6 subjects), non-small cell lung cancer (5 subjects) and colitis (3 subjects).

For adverse events of special interest (AESI), 357 patients (51.4%) treated with Durvalumab 10 mg/kg q2 weeks experienced at least 1 AESI, with potential immune-related causes and infusion reactions. Approximately half of these patients with AESIs (198, 28.5%) were considered to have Durvalumab related. AESI reported in > 2% of the patients were diarrhea, pruritus, rash, increased AST, increased GGT, increased ALT, hypothyroidism, increased blood bilirubin, increased blood creatinine, hyperthyroidism, maculo-papular rash, and erythema. Most AESIs are grade 1 or grade 2 severity. Nine patients (1.3%) had serious AESIs that were considered to be treatment related. Of the treatment related serious AESIs, colitis (2 subjects), colitis and diarrhea (1 subject), and acute kidney injury and pneumonitis (1 subject).

No dose-limiting toxicities (DLTs) have been reported in any of the dose-escalation cohorts of the monotherapy studies. Overall, the AE profile of Durvalumab was consistent with the pharmacology of the target. No tumor types appeared to be associated with unique AEs.

1.2.2.11 Durvalumab Pharmacokinetics (PK)

PK data are available in 378 patients in dose-escalation and in the expansion cohort of the

phase1/2 Durvalumab monotherapy study (NCT01693562). PK parameters including C_{max} and AUC_{0-14} were estimated after the first dose of Durvalumab at 0.1, 0.3, 1, 3, 10 mg/kg q2 weeks, and 15 mg/kg mg/kg q2 weeks. Peak concentration increased in an approximately dose-proportional manner over the dose range of 0.1 to 15 mg/kg. Area under the concentration-time curve from 0 to 14 days increased in a greater than dose-proportional manner over the dose range of 0.1 to 15 mg/kg and approached linearity at ≥ 3 mg/kg (**Table 3**). These results suggest that Durvalumab exhibits nonlinear PK likely due to saturable target-mediated clearance.

Exposures following multiple doses demonstrated accumulation consistent with PK parameters estimated from the first dose. Similar steady state PK exposure was achieved following 10 mg/kg q2 weeks and 15 mg/kg q3 weeks dosing regimens.

Table 3. Durvalumab PK parameters from the first and steady-state doses

Dosing Regimen	N	AUC_{0-14} (d·µg/mL)	C_{max} (µg/mL)
0.1 mg/kg Q2W	4	5.4 (2.3)	2.8 (0.6)
0.3 mg/kgQ2W	4	26.2 (14.3)	8.0 (1.7)
1 mg/kg Q2W	3	132 (28.4)	22.8 (2.5)
3 mg/kg Q2W	2	455 (54.5)	70.8 (12.6)
10 mg/kg Q2W	18	1241 (615)	214 (70.7)
15 mg/kg Q3W	6	2373 (702)	421 (90.6)

1.2.2.12 A fixed dosing schedule of Durvalumab

A population PK model was developed for Durvalumab using monotherapy data from a phase 1 study (NCT01693562; N=292; doses= 0.1 to 10 mg/kg q2 weeks or 15 mg/kg q 3 weeks in solid tumors). Population PK analysis indicated only minor impact of body weight on PK of Durvalumab (coefficient of ≤ 0.5). The impact of body weight -based (10 mg/kg q2 weeks) and fixed dosing (750 mg q2 weeks) of Durvalumab was evaluated by comparing predicted steady state PK concentrations (5th, median and 95th percentiles) using the population PK model. A fixed dose of 750 mg was selected to approximate 10 mg/kg (based on median body weight of ~75 kg). A total of 1000 patients were simulated using body weight distribution of 40–120 kg. Simulation results demonstrate that body weight-based and fixed dosing regimens yield similar median steady state PK concentrations with slightly less overall between-subject variability with fixed dosing regimen. Similar findings have been reported by others [Ng et al 2006, Wang et al. 2009, Zhang et al, 2012, Narwal et al 2013].[149-152] Wang and colleagues investigated 12 monoclonal antibodies and found that fixed and body size-based dosing perform similarly, with fixed dosing being better for 7 of 12 antibodies [150]. In addition, they investigated 18 therapeutic proteins and peptides and showed that fixed dosing performed better for 12 of 18 in terms of reducing the between-subject variability in pharmacokinetic/pharmacodynamics parameters [149].

A fixed dosing approach is preferred by the prescribing community due to ease of use and reduced dosing errors. Given expectation of similar pharmacokinetic exposure and variability,

we considered it feasible to switch to fixed dosing regimens. Based on average body weight of 75 kg, a fixed dose of 750 mg q2 weeks Durvalumab (equivalent to 10 mg/kg q2 weeks), 1500 mg q4 weeks Durvalumab (equivalent to 20 mg/kg q4 weeks) is included in the current study. Fixed dosing of Durvalumab is recommend only for subjects with > 30kg body weight due to endotoxin exposure. Patients with a body weight less than or equal to 30 kg should be dosed using a weight-based dosing schedule (Section **0** Appendix J).

1.2.3 Hypothesis

We hypothesize that combination therapy with olaparib and cediranib will induce increased levels of DNA damage, creating a more antigenic environment in which to stimulate the immune microenvironment. Thus, immune checkpoint inhibition could enhance the anti-tumor activity of olaparib and/or cediranib.

For the ovarian cancer cohort, we believe that it is reasonable and necessary to prospectively elucidate the safety and activity of the two doublets (a PD-L1 inhibitor/olaparib [Durvalumab+O] and a PD-L1 inhibitor/cediranib [Durvalumab+C]) due to overlapping toxicity of Durvalumab and cediranib. Our preliminary data on the phase I study of Durvalumab+C suggest an intermittent cediranib schedule (5 days on/2 days off) is more tolerable than daily schedule. We will thus examine the safey of the triplet (Durvalumab/olaparib/cediranib [Durvalumab+O+C]) using an intermittent cediranib schedule. We will subsequently examine our hypotheses of the triplet (Durvalumab+O+C) in women with recurrent ovarian cancer upon completion of phase 1 studies. The data used with the two doublets will be the foundation of a randomized phase 2 study of the triplet (Durvalumab+O+C) compared with O+C in recurrent ovarian cancer.

For the non-small cell lung cancer cohort, a positive signal in this phase 2 two-doublets study will lead to discussions of appropriate next steps (e.g. randomized phase 2 study) or the triplet to broaden evaluation of this agent in non-small cell lung cancer patients.

1.3 BACKGROUND FOR CORRELATIVE STUDIES

Despite much enthusiasm for the immunotherapy approach, only a subset of patients benefit from immune checkpoint inhibition and potentially serious side effects can sometimes accompany therapy. Predictive and pharmacodynamics biomarkers are therefore vital to further efforts by not only informing clinical decision-making but also by identifying new targets for immunomodulation. Careful histologic and genomic analysis of the tumor and microenvironment are providing additional substantive evidence for the importance of host immunity in determining clinical outcomes.

1.3.1 Immune characteristics in ovarian cancer

Recent data from The Cancer Genome Atlas (TCGA) analysis and the Australian Ovarian Cancer Study (AOCS) defined 4 subtypes of high grade epithelial ovarian cancers based upon

transcriptional array analysis[5,153]. The immunoreactive/C2 (TCGA/AOCS) groups are characterized by frequent tumor infiltrating lymphocytes. gBRCAm cancers were more likely to be classified as the immune subtype (**Figure 7**).

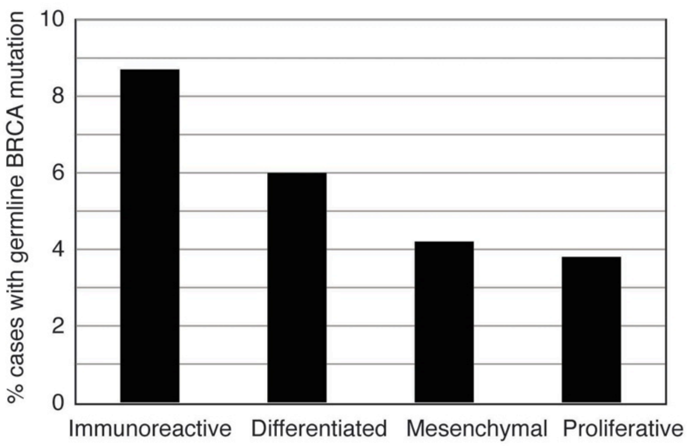

Figure 7. Immunoreactive ovarian cancer subtype cases are more likely to harbor gBRCAm. Gene expression profiling from TCGA defined 4 subsets of ovarian cancer: immunoreactive, differentiated, mesenchymal, and proliferative. Shown is the percentage of cases within each subgroup that are known to have gBRCAm (Immunoreactive = 4 of 46 cases; Differentiated = 5 of 83 cases; Mesenchymal = 2 of 48 cases; Proliferative = 3 of 79 cases).

This group of women has potential underlying global genomic instability and marked tumor genomic instability, a feature that can increase antigen quantity and exposure, leading to activation of the immune response[56]. It has been demonstrated that some gBRCAm patients have increased tumor immune cell infiltrates (described by CD20+ and cytotoxic T cell intracytoplasmic antigen-1 [TIA-1]+), compared with BRCAwt high-grade serous ovarian cancer patients (p=0.034, 0.027)[56].

We examined the peripheral blood mononuclear cell (PBMC) immune subset markers in samples obtained from ovarian cancer patients enrolled on two olaparib and carboplatin studies at NCI (08-C-0092 and 11-C-0022, unpublished data). Our goals were to investigate whether olaparib treatment yields changes of immune subset markers and whether gBRCAm patients have differences in the relative proportions of peripheral blood lymphocyte subpopulations. In collaboration with Jane Trepel's lab in the Developmental Therapeutics Branch/CCR/NCI, 41 patient samples were studied, including 25 gBRCAm and 16 BRCAwt prior to treatment of olaparib and carboplatin. Of these samples, 22 patients had paired (pre- and day 3 of olaparib therapy) samples. Quantities of CD8+ T-cells, Tregs and MDSCs (described by surface markers as Lineage$^{-/low}$, DR-, CD11b+, CD33+ cells and CD14+) were studied. Expression of CTLA-4, TIM-3 and PD-1, inhibitory immune checkpoint receptors, on CD8+ T-cell and Tregs, and % of Tregs in CD4+ T-cells were evaluated.

Overall, our preliminary assessment of immune subset markers indicated that ovarian cancer patients with gBRCAm were related to immune activated status. Specifically, these patients had both greater expression of CTLA4+ CD8+ cytotoxic T cells, and lower levels of immunosuppressive MDSCs. There was a statistically significant difference in expression of CTLA-4 on CD8+ T-cells, between gBRCAm and BRCAwt, indicating enhanced T-cell activation status. Quantities of MDSCs were significantly lower in gBRCAm patients compared to BRCAwt patients (p<0.01), suggesting gBRCAm carriers may have a distinct immune signature, in which lower MDSC quantities may be associated with enhanced anti-tumor immune

response. Further work is ongoing in collaboration with Drs. Mark Raffeld, and Stefania Pittaluga, Laboratory of Pathology/CCR/NCI, to correlate these findings with matched archival tissue samples.

1.3.2 Cytokine and chemokines analysis for immune response

Multiple cytokines and chemokines are necessary for effective host immune response against tumor cells[154]. These chemical signals attract T cells to infiltrate the tumor and also activate targeted cytotoxic responses[154]. Thus, circulating systemic levels of cytokines, chemokines, and growth factors secreted by immune cells as a consequence of immune reaction may provide insights about the immunomodulation by a PD-L1 inhibitor. We will analyze comprehensive cytokine and chemokines using V-PLEX multiplex assay (Meso Scale Discovery, Rockville, MD)[154,155] through Liang Cao's laboratory (Molecular Targets Core/NCI; **Table 4**) or other equivalent available assays. Biomarker analyses will further our understanding of the mechanisms of T cell activation in ovarian cancer, with potential applications for predictive biomarker development.

Table 4. MesoScaleDiscovery Panel of Chemokines and Cytokines

Chemokine Panel	Cytokine Panel
Eotaxin, Eotaxin-3, MCP-1, MCP-4, MDC, IL-8, IP-10, MIP-1$^{\alpha}$, MIP-1β, TARC	GM-CSF, IL-1$^{\alpha}$, IL-5, IL-7, IL-12/IL-23p40, IL-15, IL-16, IL-17A, IL-13, TNF-α, TNF-β, VEGF, IFN-γ, IL-1β, IL-2, IL-4, IL-6, IL-8, IL-10, IL-12p70

1.3.3 NF-kB signaling in immune subtypes of ovarian cancer

We hypothesize that ovarian cancers characterized by elevated NF-kB activation will be susceptible to treatment with immune checkpoint inhibitors, and that the combination of a PD-L1 inhibitor and olaparib will be particularly effective in treating gBRCAm cancers with elevated NF-kB activity. The NF-kB pathway is well studied in inflammation, and Dr. Annunziata's group in the WMB/CCR has also demonstrated this pathway to be active in a subset of ovarian cancers[156-158]. NF-kB is a family of transcription factors held in the cytoplasm when signaling is inactive, and translocated to the nucleus upon pathway activation. Dr. Annunziata defined an ovarian cancer-specific gene signature of NF-kB activity[157]. This signature is overexpressed in both the TCGA immunoreactive and the AOCS C2 immune-related subtypes of ovarian cancer, both of which contain a strong T cell infiltrate[5,153]. Expression of the NF-kB gene signature correlates with high levels of nuclear NF-kB p65 transcription factor in the ovarian cancer cells, suggesting that the gene expression is derived from the cancer cells and not purely expressed from the infiltrating immune cells. Furthermore, the NF-kB signature was more highly expressed in gBRCAm cancers compared to the group overall, thus supporting the study of immune checkpoint inhibitors specifically in this population of women (**Figure 8**).

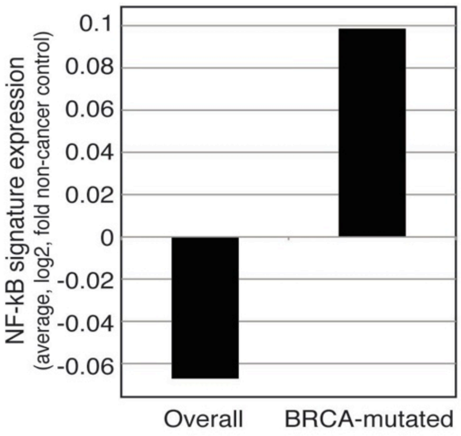

Figure 8. Ovarian cancer-specific NF-kB genes are overexpressed in gBRCAm-associated cancers. Fifteen of the TCGA samples from women known to harbor gBRCAm were available for analysis. Data contain log2-transformed values normalized to non-cancer controls.

1.4 RATIONALE

The purpose of this phase 1/2 pilot study is to test the **hypothesis** that the PD-L1 inhibitor, Durvalumab in combination with olaparib and/or cediranib can be safely administered in patients with advanced solid tumors and in an exploratory fashion will benefit women with recurrent ovarian cancer and other solid tumors.

High-grade serous ovarian cancer is a genetically unstable neoplasm. Expression of PD-L1 in ovarian cancer is prognostic of poor survival. Preliminary clinical data of a PD-1 inhibitor yielded anti-tumor activity in a subset of recurrent ovarian cancer patients. Olaparib, a PARP inhibitor, and cediranib, a VEGFR1-3 inhibitor, have single agent activity in recurrent ovarian cancer. We recently reported the combination of olaparib and cediranib was clinically superior to olaparib alone in a randomized phase 2 study. The data led to two international phase 3 trials assessing the efficacy of the combination in women with recurrent ovarian cancer.

We hypothesize that increased DNA damage and reduced VEGF signaling, subsequently creating a more antigenic environment in which to stimulate the immune microenvironment, should complement the anti-tumor activity of an immune checkpoint inhibitor in relapsed ovarian cancer. Our ultimate goal is to examine the activity of triplet therapy (Durvalumab/olaparib/cediranib) compared to the combination of olaparib and cediranib in ovarian cancer patients. We first assessed the safety and activity of the two doublets (Durvalumab/olaparib and Durvalumab/cediranib) due to the predicted overlapping toxicity of cediranib and Durvalumab. Our preliminary data on phase 1 study of Durvalumab/cediranib suggest an intermittent cediranib schedule (5 days on/2 days off) is tolerated better than a daily schedule. We examined the safety of the triplet using an intermittent cediranib schedule and found the RP2D for a phase 2 study. We will thus examine the efficacy of the triplet therapy in ovarian cancer with planned biomarkers analysis. These data should provide the foundation of a randomized phase 2 study of the triplet in women with recurrent ovarian cancer.

Additionally, we are investigating the two doublet treatments in other tumor types including advanced or recurrent NSCLC, SCLC, mCRPC, TNBC and CRC. Emerging data indicate

subsets of these cancers have dysfunctions in genes related to DNA damage repair pathways. The purpose of the expansion cohorts is to test the hypothesis that the PD-L1 inhibitor, Durvalumab in combination with olaparib or cediranib can be safely administered and may yield clinical benefit in patients with NSCLC, SCLC, mCRPC, TNBC or CRC. Increased DNA damage by olaparib, creating a more antigenic environment in which to stimulate the immune microenvironment, should complement the anti-tumor activity of an immune checkpoint inhibitor in NSCLC, SCLC, TNBC or mCRPC. A positive signal in the pilot Phase 2 studies in other solid tumors will lead to discussions of appropriate next steps (e.g. randomized phase 2 study with one of Durvalumab+O or Durvalumab+C or the triplet study) to broaden evaluation of these agents.

1.5 PHASE I RESULTS AND IMPLEMENTATION OF PHASE II

Twenty six women were treated on Phase I study of Durvalumab with olaparib or cediranib [159]; Durvalumab/olaparib (Durvalumab+O) arm included 10 ovarian cancer and 2 triple negative breast cancer patients. The MTD for Durvalumab+O is dose level 3. The recommended phase II dose of Durvalumab+O is olaparib tablets 300mg bid daily and Durvalumab at a fixed dose of 1500mg every 28 days. Effective with Amendment C (version date 12/28/2015), the Phase II study of Durvalumab+O opened in ovarian cancer, TNBC, NSCLC, SCLC and mCRPC with a RP2D of olaparib 300mg tablets bid daily and Durvalumab at a fixed dose of 1500mg IV every 28days.

For the Durvalumab/cediranib (Durvalumab+C), Durvalumab+C with cediranib daily schedule arm had 9 ovarian cancer, 2 cervical cancer and 3 uterine cancer patients. No DLT was identified for the daily cedrinaib schedule; however, 2 patients on DL1 required early discontinuation of cediarnib due to pulmonary thromboembolism and 1 patient on DL1 had dose reduction to cediranib15mg daily due to recurrent grade 2 fatigue on cycle 2. Three of 4 patients on DL2 also had cediranib dose reduction to 20mg daily due to recurrent grade 2 fagitue, grade 2 abdominal pain and grade 2 diarrhea during cycles 2-3. In addition, the preclinical *in vivo* data from Astrazeneca/MedImmune (personal communications) showed no difference in anti-tumor activity with intermittent cediranib schedules (5 days on/2 days off) compared with daily cediranib schedule. Thus, effective with Amendment C (version date 12/28/2015), we opened the new Durvalumab+C dose levels with an intermittent cediranib schedule (5 days on/2 days off) and Durvalumab at a fixed dose of 1500mg every 28days to improve tolerability. Six patients (5 ovarian cancer and 1 endometrial cancer) were treated on cediranib 20mg 5 days on/2 days off schedule. One patient developed DLT (grade 4 hypertension and grade 2 TIA) who had previous history of cardiovascular disease. Five patients tolerated an intermittent cediranib schedule and three of them had PR. Effective with Amendment H (version date 07/07/2016), we opened the Phase II study of Durvalumab+C in ovarian cancer, NSCLC, and CRC with RP2D of Durvalumab+C (cediranib 20mg once a day (5 days on/2 days off) and Durvalumab at a fixed dose of 1500mg every 28days).

In addition, effective with Amendment G (version date 4/28/2016), we opened the Phase I triplet (Durvalumab+O+C) cohort using an intermittent cediranib dose schedule in advanced solid tumors to examine the safety and tolerability. As of April 2017, nine women were treated in this cohort. Of the 9 patients, 7 had ovarian, 1 endometrial and 1 triple negative breast cancer. Grade 3 and 4 AEs include hypertension (1/9), anemia (1/9) and lymphopenia (3/9). No one

experienced DLTs. One patient required dose reduction during cycle 5, for grade 3 anemia. Three PRs were observed in 2 ovarian and 1 endometrial cancer (response rate of 33 % , median 5 months [4+-6+]). We identified the RP2D of a triplet therapy (olaparib tablets 300mg twice daily, cediranib 20mg once a day (5 days on/2 days off) and Durvalumab at a fixed dose of 1500mg every 28days). Effective with amendment M (version date 7/19/2017), we will open the Phase II study of Durvalumab+O+C to study clinical activity in ovarian cancer patients.

1.6 PHARMACEUTICAL MANUFACTURER UPDATES

1.6.1 Cediranib

As of July 20, 2020, the overall safety profile of cediranib is based on data from approximately 3178 patients treated in AstraZeneca sponsored trials. These patients had various malignancies and were exposed to cediranib in combination with chemotherapy or as monotherapy, at doses ranging from 0.5 to 90 mg. A further 37 patients have received cediranib in a Named Patient Supply program. In addition, approximately 6077 patients have been enrolled in clinical trials in investigator-led or collaborative group-sponsored studies in which cediranib was supplied in one or more treatment arms.

1.6.2 Olaparib

Based on data collected through December 15, 2020, substantial changes were made to the adverse drug reactions with the addition of Myelodysplastic syndrome, Normocytic anemia, Refractory anemia with an excess of blasts, Myeloid leukemia, Acute myeloid leukemia, Acute erythroid leukemia, Chronic myelomonocytic leukemia, Angioedema, Erythema nodosum, and Rash macular to the Rare and Serious category.

2 ELIGIBILITY ASSESSMENT AND ENROLLMENT

2.1 PHASE I STUDY ELIGIBILITY CRITERIA

2.1.1 Inclusion criteria

2.1.1.1 Patients must have histologically or cytologically confirmed advanced solid tumor that is refractory to standard treatment or for which no standard treatment exists.

2.1.1.2 All patients must have evaluable disease; biomarker-only disease is not considered evaluable; eligibility of breast cancer with bone only disease is a principal investigator (PI) decision on an individual patient basis.

2.1.1.3 Age ≥18 years.

2.1.1.4 ECOG performance status ≤2. (See **APPENDICES**

Appendix A)

2.1.1.5 Patients must have adequate organ and marrow function as defined below:

- absolute neutrophil count ≥ 1,500/mcL
- white blood cell (WBC) ≥ 3,000/mcL
- platelets ≥ 100,000/mcL
- hemoglobin (Hgb) ≥ 10 g/dL in the absence of packed red blood cell transfusion 28 days prior to

dosing

OR

- Hgb 9-9.9 g/dL and the value is constant across 2 readings within 2 weeks, in the absence of packed red blood cell transfusion 28 days prior to dosing
- total bilirubin $\leq$ 1.5 X the normal institutional limits
- AST(SGOT)/ALT(SGPT) $\leq$ 2.5 X institutional upper limit of normal
- creatinine $\leq$ 1.5 X within normal institutional limits

OR

measured creatinine clearance $\geq$ 50 mL/min/1.73 m^2

- Spot urine protein/creatinine ratio $\leq$ 1

OR

24 hour urine protein $\leq$ 1000 mg

2.1.1.6 Patients are allowed to have received prior PARP inhibitors (PARPi), and/or anti-angiogenesis therapy including but not limited to thalidomide, bevacizumab, sunitinib, sorafenib, or other anti-angiogenics. However, patients who were treated with both olaparib and cediranib, either in combination or sequentially are not eligible. For this study, BSI-201 (iniparib) is not considered as PARPi.

2.1.1.7 Patients must be able to swallow oral medications (capsules and tablets) without chewing, breaking, crushing, opening or otherwise altering the product formulation. They should not have gastrointestinal illnesses that would preclude the absorption of cediranib or olaparib, which are oral agents.

2.1.1.8 Adequately controlled blood pressure (SBP < 140 mm Hg and DBP < 90mmHg) on a maximum of three antihypertensive medications.

2.1.1.9 Toxicities of prior therapy (excepting alopecia) should be resolved to less than or equal to Grade 1 as per CTCAE v 4.03 (http://ctep.cancer.gov/protocolDevelopment/electronic_applications/ctc.htm) except hemoglobin as shown in 2.1.1.6. Patients with long-standing stable grade 2 neuropathy may be considered after discussion with the PI.

2.1.1.10 Ability of subject to understand and the willingness to sign a written informed consent document prior to any protocol related procedures, including screening evaluations.

2.1.1.11 Ability of subject to understand and the willingness to record twice-daily blood pressure readings if the patient is enrolled to the Durvalumab+C arm or Durvalumab+O+C arm.

2.1.1.12 Female patients must either be of non-reproductive potential (i.e., post-menopausal by history: $\geq$60 years old and no menses for $\geq$1 year without an alternative medical cause; OR history of hysterectomy, OR history of bilateral tubal ligation, OR history of bilateral oophorectomy) or must have a negative serum pregnancy test upon study entry and agree to use contraception or abstinence (See Section **2.10**) for female patients with

reproductive potential. See Section **2.10** for male patients for contraception while on the study.

2.1.2 Exclusion criteria

2.1.2.1 Patients who have received chemotherapy, radiotherapy, any other investigational agents within 4 weeks (6 weeks for nitrosoureas or mitomycin) prior to study enrollment.

2.1.2.2 Patients who were treated with both olaparib and cediranib, either in combination or sequentially.

2.1.2.3 Patients who have had prior immune checkpoint inhibitors, such as Durvalumab or other PD1 or PD-L1 inhibitors or an anti-CTLA4 therapy.

2.1.2.4 Patients receiving any medications or substances that are strong inhibitors or inducers of CYP3A4 are ineligible. A list of CYP3A4 inhibitors and inducers is provided in **Appendix B**. Dihydropyridine calcium-channel blockers are permitted for management of hypertension.

2.1.2.5 Major surgical procedure (as defined by the investigator) within 30 days prior to the first dose of Durvalumab or still recovering from prior surgery.

2.1.2.6 Patients with any other concomitant or prior invasive malignancies are ineligible, However, patients with prior cancer treated with a curative intent with no evidence of recurrent disease 5 years following diagnosis and judged by the investigator to be at low risk of recurrence are eligible. Patients with treated limited stage basal cell or squamous cell carcinoma of the skin or carcinoma in situ of the breast or cervix are eligible.

2.1.2.7 Patients with evidence of CNS metastasis, spinal cord compression, or leptomeningeal disease within one year prior to enrollment will be excluded from this clinical trial because of their poor prognosis and because they often develop progressive neurologic dysfunction that would confound the evaluation of neurologic and other adverse events. Patients with a remote history of brain metastases may be considered if they received sterilizing therapy to the CNS (resection or radiation) and have been CNS progression-free for the 1-year period. Baseline imaging to rule out brain metastases is not required for screening, but should be performed prior to study enrollment if clinically indicated.

2.1.2.8 History of allergic reactions attributed to compounds of similar chemical or biologic composition to Durvalumab, olaparib, cediranib, or to other humanized monoclonal antibodies. Known history of anaphylaxis, angioedema, laryngeal edema, serum sickness, or uncontrolled asthma.

2.1.2.9 History of auto-immune disease requiring steroid maintenance, or history of primary immunodeficiency.

2.1.2.10 Current or prior use of immunosuppressive medication within 28 days before the first dose of Durvalumab, with the exception of intranasal and inhaled corticosteroids or systemic corticosteroids at physiological doses, which are not to exceed 10 mg/day of prednisone or an equivalent corticosteroid. In the case of short term use of systemic corticosteroids (less than 24 hours within 28 days) of greater than 10 mg/day of

prednisone or an equivalent corticosteroid, the required washout period prior to starting the first dose of Durvalumab is 7 days.

2.1.2.11 Active or prior documented inflammatory bowel disease (e.g., Crohn's disease, ulcerative colitis). Eligibility for patients with asymptomatic and a previous diagnosis of immune or inflammatory colitis, or patients with chronic diarrhea > 1 month without immune or inflammatory colitis is a PI decision on an individual patient basis.

2.1.2.12 Receipt of live attenuated vaccination within 30 days before the first dose of Durvalumab.

2.1.2.13 Patients who have the following clinical conditions are considered to be at increased risk for cardiac toxicities. Patients with any cardiac history of the following conditions within 1 year prior to Durvalumab+O study or within 2 years prior to Durvalumab+C or Durvalumab+O+C enrollment are excluded from the study:

- Prior events including myocardial infarction, clinically significant pericardial effusion, and myocarditis.
- Prior cardiac arrhythmia including atrial fibrillation and atrial flutter, or requiring concurrent use of drugs or biologics with pro-arrhythmic potential.
- NYHA Class II or greater heart failure.
- If cardiac function assessment is clinically indicated or performed, an LVEF less than normal per institutional guidelines, or <55%, if threshold for normal is not otherwise specified by institutional guidelines.
- Mean QT interval corrected for heart rate (QTc) ≥470 ms calculated from 3 electrocardiograms (ECGs) using Frediricia's Correction or other significant ECG abnormality noted within 14 days of treatment.
- Hypertensive crisis or hypertensive encephalopathy.
- Clinically significant peripheral vascular disease or vascular disease, including rapidly growing aortic aneurysm or abdominal aortic aneurysm >5 cm or aortic dissection.
- Unstable angina.

2.1.2.14 History of cerebrovascular accident, transient ischemic attack within 1 year prior to study enrollment.

2.1.2.15 History of abdominal fistula, gastrointestinal perforation, or intra-abdominal abscess within 6 months prior to study enrollment.

2.1.2.16 Significant hemorrhage (> 30 mL bleeding/episode within 3 months before study enrollment) or hemoptysis (> 5mL fresh blood within 28 days before study enrollment).

2.1.2.17 Current signs and/or symptoms of bowel obstruction or signs and/or symptoms of bowel obstruction within 28 days before study enrollment.

2.1.2.18 Current dependency on total parenteral nutrition (TPN) or IV fluid hydration.

2.1.2.19 Any prior grade ≥ 3 immune-related adverse event (irAE)[160] while receiving any previous immunotherapy agent, or any unresolved irAE > grade 1. Toxicities of prior therapy, excepting alopecia, should be resolved to less than or equal to grade 1 as per NCI-CTCAE v4.0 (located on the CTEP website at

http://ctep.cancer.gov/protocolDevelopment/electronic_applications/ctc.htm). Patients with greater than 1+ proteinuria at entry are ineligible.

2.1.2.20 Uncontrolled intercurrent illness including, but not limited to, ongoing or active infection, symptomatic congestive heart failure, unstable angina pectoris, cardiac arrhythmia, active peptic ulcer disease, or psychiatric illness/social situations that would limit compliance with study requirements.

2.1.2.21 Pregnant and breastfeeding women are excluded from this study.

2.1.2.22 HIV-positive patients on antiretroviral therapy are ineligible because of potential pharmacokinetic interactions with study drugs. However, patients with long-standing (>5 years) HIV on antiretroviral therapy > 1 month (undetectable HIV viral load and CD4 count > 150 cells/µL) may be eligible if the PI determines no anticipated clinically significant drug-drug interactions.

2.1.2.23 HBV-or HCV-positive patients are ineligible because of potential reactivation of hepatitis virus following steroids.

2.1.2.24 Known history of previous clinical diagnosis of tuberculosis.

2.1.2.25 No baseline features suggestive of myelodysplastic syndrome or acute myelogenous leukemia on peripheral blood smear or bone marrow biopsy, if clinically indicated.

2.1.2.26 No prior or current evidence of coagulopathy or bleeding diathesis. Therapeutic anticoagulation for prior thromboembolic events is permitted.

2.1.2.27 Concurrent enrollment in another clinical study, unless it is an observational non-interventional clinical study or the follow-up of an interventional study.

2.1.2.28 Any concurrent chemotherapy, immunotherapy, biologic or hormonal therapy for cancer treatment. Concurrent use of hormones for non-cancer-related conditions (e.g., insulin for diabetes and hormone replacement therapy) is acceptable. NOTE: Local treatment of isolated lesions for palliative intent is acceptable (e.g., by local surgery or radiotherapy).

2.2 Phase II Study Cohort 1 Ovarian Cancer Eligibility Criteria (Durvalumab+O, Durvalumab+C and Durvalumab+O+C)

2.2.1 Inclusion criteria

2.2.1.1 Durvalumab+O and Durvalumab+C: Patients must have histologically or cytologically confirmed persistent or recurrent ovarian, fallopian tube, or primary peritoneal cancer who are either platinum-sensitive, platinum resistant or refractory during or after a first platinum containing regimen. For platinum-sensitive recurrent disease, the patients must have received at least two prior regimens prior to study enrollment.

2.2.1.2 Durvalumab+O+C: Patients must have histologically or cytologically confirmed persistent or recurrent non-mucinous epithelial ovarian, fallopian tube, or primary peritoneal cancer who are either platinum-sensitive, platinum resistant or refractory during or after a first platinum containing regimen. For platinum-sensitive recurrent serous epithelial ovarian cancer, the patients must have received at least two prior regimens prior to study enrollment.

2.2.1.3 Documentation of gBRCAm status will be requested. A documented deleterious germline BRCA1 and BRCA2 mutation (gBRCAm) obtained in a CLIA-certified laboratory will be requested, but it is not mandated for enrollment. Due to the long acceptance of BRCA testing through Myriad, Myriad testing will be accepted. If testing for BRCA is done by other organizations either by multi-gene panels or individual testing, genetic consultation report from a qualified medical professional listing the mutation and confirming that the laboratory results showed a recognized gBRCAm or germline deleterious BRCA rearrangement is required. If the patient refused genetic counseling, it should be documented in the medical records.

Variants of uncertain significance (VUS) of BRCA1 and BRCA2 are not considered deleterious. Patients with VUS or deleterious mutation in other genes without gBRCAm or patients with negative BRCA testing are still eligible.

2.2.1.4 Patients must have measurable disease as defined by RECIST v1.1. See Section **7.5** for the evaluation of measurable disease.

2.2.1.5 Patients must have at least one lesion deemed safe to biopsy and be willing to undergo a mandatory baseline biopsy.

2.2.1.6 Age ≥18 years.

2.2.1.7 ECOG performance status ≤ 2. (See **APPENDICES**

2.2.1.8 **Appendix** A)

2.2.1.9 Patients must have adequate organ and marrow function as defined below:

• absolute neutrophil count	≥ 1,500/mcL
• white blood cell (WBC)	≥ 3,000/mcL
• platelets	≥ 100,000/mcL
• hemoglobin (Hgb)	≥ 9 g/dL in the absence of packed red blood cell transfusion 28 days prior to dosing
• AST(SGOT)/ALT(SGPT)	≤ 2.5 X institutional upper limit of normal (ULN); for subjects with liver metastases, AST or ALT ≤ 5 × ULN
• Total bilirubin	≤ 1.5 × ULN; for subjects with documented/suspected Gilbert's disease, bilirubin ≤ 3 × ULN
• creatinine	≤ 1.5 X within normal institutional limits

OR

measured creatinine clearance ≥ 50 mL/min/1.73 m^2

- For OvCa Durvalumab+C and Durvalumab+O+C arms only:

  Spot urine protein/creatinine ratio ≤ 1

  OR

  24 hour urine protein ≤ 1000 mg

2.2.1.10 Patients are allowed to have received prior PARP inhibitors (PARPi), and/or anti-angiogenesis therapy including but not limited to thalidomide, bevacizumab, sunitinib, sorafenib, or other anti-angiogenics. However, patients who were treated with both olaparib and cediranib, either in combination or sequentially are not eligible. For this study, BSI-201 (iniparib) is not considered as PARPi.

2.2.1.11 Patients must be able to swallow oral medications (capsules and tablets) without chewing, breaking, crushing, opening or otherwise altering the product formulation. They should not have gastrointestinal illnesses that would preclude the absorption of cediranib or olaparib, which are oral agents.

2.2.1.12 Adequately controlled blood pressure (SBP < 140 mm Hg and DBP < 90mmHg) on a maximum of three antihypertensive medications.

2.2.1.13 Toxicities of prior therapy (excepting alopecia) should be resolved to less than or equal to Grade 1 as per CTCAE v 4.03 (http://ctep.cancer.gov/protocolDevelopment/electronic_applications/ctc.htm) except hemoglobin as shown in 2.2.1.8. Patients with long-standing stable grade 2 neuropathy may be considered after discussion with the PI.

2.2.1.14 Ability of subject to understand and the willingness to sign a written informed consent document prior to any protocol related procedures, including screening evaluations.

2.2.1.15 Ability of subject to understand and the willingness to record twice-daily blood pressure readings if the patient is enrolled to the Durvalumab+C or Durvalumab+O+C arm.

2.2.1.16 Female patients must either be of non-reproductive potential (i.e., post-menopausal by history: ≥60 years old and no menses for ≥1 year without an alternative medical cause; OR history of hysterectomy, OR history of bilateral tubal ligation, OR history of bilateral oophorectomy) or must have a negative serum pregnancy test upon study entry and agree to use contraception or abstinence (See Section **2.10**) for female patients with reproductive potential. See Section **2.10** for male patients for contraception while on the study.

2.2.2 Exclusion criteria

2.2.2.1 Patients who have received chemotherapy, radiotherapy, any other investigational agents within 3 weeks (4 weeks for platinum agents and 6 weeks for nitrosoureas or mitomycin) prior to study enrollment.

2.2.2.2 Patients who were treated with both olaparib and cediranib, either in combination or sequentially.

2.2.2.3 Patients who have had Durvalumab. Prior treatment with other immune checkpoint inhibitors is allowed.

2.2.2.4 Patients receiving any medications or substances that are strong inhibitors or inducers of CYP3A4 are ineligible. A list of CYP3A4 inhibitors and inducers is provided in **Appendix B**. Dihydropyridine calcium-channel blockers are permitted for management of hypertension.

2.2.2.5 Major surgical procedure (as defined by the investigator) within 30 days prior to the first dose of Durvalumab or still recovering from prior surgery.

2.2.2.6 Patients with any other concomitant or prior invasive malignancies are ineligible. However, patients with prior cancer treated with a curative intent with no evidence of recurrent disease 5 years following diagnosis and judged by the investigator to be at low risk of recurrence are eligible. Patients with treated limited stage basal cell or squamous cell carcinoma of the skin or carcinoma in situ of the breast or cervix are eligible.

2.2.2.7 Patients with evidence of CNS metastasis, spinal cord compression, or leptomeningeal disease within one year prior to enrollment will be excluded from this clinical trial because of their poor prognosis and because they often develop progressive neurologic dysfunction that would confound the evaluation of neurologic and other adverse events. Patients with a remote history of brain metastases may be considered if they received sterilizing therapy to the CNS (resection or radiation) and have been CNS progression-free for the 1-year period. Baseline imaging to rule out brain metastases is not required for screening, but should be performed prior to study enrollment if clinically indicated. Patients with spinal cord compression may be considered if the condition is medically managed and currently asymptomatic.

2.2.2.8 History of allergic reactions attributed to compounds of similar chemical or biologic composition to Durvalumab, olaparib, cediranib, or to other humanized monoclonal antibodies. Known history of anaphylaxis, angioedema, laryngeal edema, serum sickness, or uncontrolled asthma.

2.2.2.9 History of auto-immune disease requiring steroid maintenance, or history of primary immunodeficiency.

2.2.2.10 Current or prior use of immunosuppressive medication within 28 days before the first dose of Durvalumab, with the exception of intranasal and inhaled corticosteroids or systemic corticosteroids at physiological doses, which are not to exceed 10 mg/day of prednisone or an equivalent corticosteroid. In the case of short term use of systemic corticosteroids (less than 24 hours within 28 days) of greater than 10 mg/day of prednisone or an equivalent corticosteroid, the required washout period prior to starting the first dose of Durvalumab is 7 days.

2.2.2.11 Active or prior documented inflammatory bowel disease (e.g., Crohn's disease, ulcerative colitis). Eligibility for patients with asymptomatic and a previous diagnosis of immune or inflammatory colitis, or patients with chronic diarrhea > 1 month without immune or inflammatory colitis is a PI decision on an individual patient basis.

2.2.2.12 Receipt of live attenuated vaccination within 30 days before the first dose of Durvalumab.

2.2.2.13 Patients who have the following clinical conditions are considered to be at increased risk for cardiac toxicities. Patients with any cardiac history of the following conditions

within 1 year prior to Durvalumab+O arm or within 2 years prior to Durvalumab+C or Durvalumab+O+C arm enrollment are excluded from the study:

- Prior events including myocardial infarction, clinically significant pericardial effusion, and myocarditis.
- Prior cardiac arrhythmia including atrial fibrillation (except chronic atrial fibrillation with controlled vascular rate), and atrial flutter, or requiring concurrent use of drugs or biologics with pro-arrhythmic potential.
- NYHA Class II or greater heart failure.
- If cardiac function assessment is clinically indicated or performed, an LVEF less than normal per institutional guidelines, or <55%, if threshold for normal is not otherwise specified by institutional guidelines.
- Mean QT interval corrected for heart rate (QTc) ≥470 ms calculated from 3 electrocardiograms (ECGs) using Frediricia's Correction or other significant ECG abnormality noted within 14 days of treatment.
- Hypertensive crisis or hypertensive encephalopathy.
- Clinically significant peripheral vascular disease or vascular disease, including rapidly growing aortic aneurysm or abdominal aortic aneurysm >5 cm or aortic dissection.
- Unstable angina.

2.2.2.14 History of cerebrovascular accident, transient ischemic attack within 1 year prior to study enrollment.

2.2.2.15 History of abdominal fistula, gastrointestinal perforation, or intra-abdominal abscess within 6 months prior to study enrollment.

2.2.2.16 Significant hemorrhage (> 30 mL bleeding/episode within 3 months before study enrollment) or haemoptysis (> 5mL fresh blood within 28 days before study enrollment).

2.2.2.17 Current signs and/or symptoms of bowel obstruction or signs and/or symptoms of bowel obstruction within 28 days before study enrollment.

2.2.2.18 Current dependency on TPN or IV fluid hydration.

2.2.2.19 Any prior grade ≥ 3 immune-related adverse event (irAE)[160] while receiving any previous immunotherapy agent, or any unresolved irAE > grade 1. Toxicities of prior therapy, excepting alopecia, should be resolved to less than or equal to grade 1 as per NCI-CTCAEv4.0 (located on the CTEP website at http://ctep.cancer.gov/protocolDevelopment/electronic_applications/ctc.htm). Patients with greater than 1+ proteinuria at entry are ineligible.

2.2.2.20 Uncontrolled intercurrent illness including, but not limited to, ongoing or active infection, symptomatic congestive heart failure, unstable angina pectoris, cardiac arrhythmia (except chronic atrial fibrillation with controlled vascular rate), active peptic ulcer disease, or psychiatric illness/social situations that would limit compliance with study requirements.

2.2.2.21 Pregnant and breastfeeding women are excluded from this study.

2.2.2.22 HIV-positive patients on antiretroviral therapy are ineligible because of potential pharmacokinetic interactions with study drugs. However, patients with long-standing (>5 years) HIV on antiretroviral therapy > 1 month (undetectable HIV viral load and CD4 count > 150 cells/µL) may be eligible if the PI determines no anticipated clinically significant drug-drug interactions.

2.2.2.23 HBV-or HCV-positive patients are ineligible because of potential reactivation of hepatitis virus following steroids.

2.2.2.24 Known history of previous clinical diagnosis of tuberculosis.

2.2.2.25 No baseline features suggestive of myelodysplastic syndrome or acute myelogenous leukemia on peripheral blood smear or bone marrow biopsy, if clinically indicated.

2.2.2.26 No prior or current evidence of coagulopathy or bleeding diathesis. Therapeutic anticoagulation for prior thromboembolic events is permitted.

2.2.2.27 Concurrent enrollment in another clinical study, unless it is an observational non-interventional clinical study or the follow-up of an interventional study.

2.2.2.28 Any concurrent chemotherapy, immunotherapy, biologic or hormonal therapy for cancer treatment. Concurrent use of hormones for non-cancer-related conditions (e.g., insulin for diabetes and hormone replacement therapy) is acceptable. NOTE: Local treatment of isolated lesions for palliative intent is acceptable (e.g., by local surgery or radiotherapy).

2.3 Phase II Study cohort 5 Triple Negative Breast Cancer Eligibility Criteria (Durvalumab+O Only)

2.3.1 Inclusion criteria

2.3.1.1 Patients must have histologically confirmed persistent or recurrent triple-negative breast cancer (TNBC) for which standard curative measures do not exist or are no longer effective. ER/PR/HER2 status needs to be documented either by an outside source or at NCI.

2.3.1.2 Documentation of germline *BRCA1* and *BRCA2* mutation (gBRCAm) status will be requested for eligibility. A documented deleterious gBRCAm obtained in a CLIA-certified laboratory, including but not limited to Myriad Genetics, either by multi-gene panels or individual testing, will be required prior to study enrollment. Variants of uncertain significance (VUS) of BRCA1 and BRCA2 are not considered deleterious mutation. Patients with VUS or deleterious mutation in other genes without gBRCAm or patients with negative BRCA testing are still eligible.

2.3.1.3 Patients must have measurable disease as defined by RECIST v1.1. See Section **7.5** for the evaluation of measurable disease.

2.3.1.4 Patients must have at least one lesion deemed safe to biopsy and be willing to undergo a mandatory baseline biopsy.

2.3.1.5 Age ≥18 years.

2.3.1.6 ECOG performance status ≤ 2. (See **Appendix A: Performance Status Criteria**.)

2.3.1.7 Patients must have adequate organ and marrow function as defined below:

- absolute neutrophil count — ≥ 1,500/mcL
- white blood cell (WBC) — ≥ 3,000/mcL
- platelets — ≥ 100,000/mcL
- hemoglobin (Hgb) — ≥ 9 g/dL in the absence of packed red blood cell transfusion 28 days prior to dosing
- AST(SGOT)/ALT(SGPT) — ≤ 2.5 X institutional upper limit of normal (ULN); for subjects with liver metastases, AST or ALT ≤ 5 × ULN

a. Total bilirubin — ≤ 1.5 × ULN; for subjects with documented/suspected Gilbert's disease, bilirubin ≤ 3 × ULN

- creatinine — ≤ 1.5 X within normal institutional limits

OR

measured creatinine clearance ≥ 50 mL/min/1.73 m^2

2.3.1.8 Patients must be able to swallow oral medications (capsules and tablets) without chewing, breaking, crushing, opening or otherwise altering the product formulation. They should not have gastrointestinal illnesses that would preclude the absorption of cediranib or olaparib, which are oral agents.

2.3.1.9 Toxicities of prior therapy (excepting alopecia) should be resolved to less than or equal to Grade 1 as per CTCAE v 4.03 (http://ctep.cancer.gov/protocolDevelopment/electronic_applications/ctc.htm) except hemoglobin as shown in 2.3.1.8. Patients with long-standing stable grade 2 neuropathy may be considered after discussion with the PI.

2.3.1.10 Ability of subject to understand and the willingness to sign a written informed consent document prior to any protocol related procedures, including screening evaluations.

2.3.1.11 Female patients must either be of non-reproductive potential (i.e., post-menopausal by history: ≥60 years old and no menses for ≥1 year without an alternative medical cause; OR history of hysterectomy, OR history of bilateral tubal ligation, OR history of bilateral oophorectomy) or must have a negative serum pregnancy test upon study entry and agree to use contraception or abstinence (See Section **2.10**) for female patients with reproductive potential. See Section **2.10** for male patients for contraception while on the study.

2.3.2 Exclusion criteria

2.3.2.1 Patients who have received chemotherapy in the previous 3 weeks (6 weeks for nitrosoureas or mitomycin); or who received radiotherapy or any other investigational agents within 3 weeks prior to study enrollment.

2.3.2.2 Patients who have received prior PARP inhibitors (PARPi) are ineligible. For this study, BSI-201 (iniparib) is not considered as PARPi.

2.3.2.3 Patients who have had Durvalumab. Prior treatment with other immune checkpoint inhibitors is allowed.

2.3.2.4 Patients receiving any medications or substances that are strong inhibitors or inducers of CYP3A4 are ineligible. A list of CYP3A4 inhibitors and inducers is provided in **Appendix B**. Dihydropyridine calcium-channel blockers are permitted for management of hypertension.

2.3.2.5 Major surgical procedure (as defined by the investigator) within 30 days prior to the first dose of Durvalumab or still recovering from prior surgery.

2.3.2.6 Patients with any other concomitant or prior invasive malignancies are ineligible. However, patients with prior cancer treated with a curative intent with no evidence of recurrent disease 5 years following diagnosis and judged by the investigator to be at low risk of recurrence are eligible. Patients with treated limited stage basal cell or squamous cell carcinoma of the skin or carcinoma in situ of the breast or cervix are eligible.

2.3.2.7 Patients with evidence of CNS metastasis, spinal cord compression, or leptomeningeal disease within six months prior to enrollment will be excluded from this clinical trial because of their poor prognosis and because they often develop progressive neurologic dysfunction that would confound the evaluation of neurologic and other adverse events. Patients with a remote history of brain metastases may be considered if they received sterilizing therapy to the CNS (resection or radiation) and have been CNS progression-free for the six months period. Baseline imaging to rule out brain metastases is not required for screening, but should be performed prior to study enrollment if clinically indicated. Patients with spinal cord compression may be considered if the condition is medically managed and currently asymptomatic.

2.3.2.8 History of allergic reactions attributed to compounds of similar chemical or biologic composition to Durvalumab, olaparib or to other humanized monoclonal antibodies. Known history of anaphylaxis, angioedema, laryngeal edema, serum sickness, or uncontrolled asthma.

2.3.2.9 History of auto-immune disease requiring steroid maintenance, or history of primary immunodeficiency.

2.3.2.10 Current or prior use of immunosuppressive medication within 28 days before the first dose of Durvalumab, with the exception of intranasal and inhaled corticosteroids or systemic corticosteroids at physiological doses, which are not to exceed 10 mg/day of prednisone or an equivalent corticosteroid. In the case of short term use of systemic corticosteroids (less than 24 hours within 28 days) of greater than 10 mg/day of prednisone or an equivalent corticosteroid, the required washout period prior to starting the first dose of Durvalumab is 7 days.

2.3.2.11 Active or prior documented inflammatory bowel disease (e.g., Crohn's disease, ulcerative colitis). Eligibility for patients with asymptomatic and a previous diagnosis of immune or inflammatory colitis, or patients with chronic diarrhea > 1 month without immune or inflammatory colitis is a PI decision on an individual patient basis.

2.3.2.12 Receipt of live attenuated vaccination within 30 days before the first dose of Durvalumab.

2.3.2.13 Patients who have the following clinical conditions are considered to be at increased risk for cardiac toxicities. Patients with any cardiac history of the following conditions within 1 year prior to study enrollment are excluded from the study:

- Prior events including myocardial infarction, clinically significant pericardial effusion, and myocarditis.
- Prior cardiac arrhythmia including atrial fibrillation (except chronic atrial fibrillation with controlled vascular rate) and atrial flutter, or requiring concurrent use of drugs or biologics with pro-arrhythmic potential.
- NYHA Class II or greater heart failure.
- If cardiac function assessment is clinically indicated or performed, an LVEF less than normal per institutional guidelines, or <55%, if threshold for normal is not otherwise specified by institutional guidelines.
- Mean QT interval corrected for heart rate (QTc) ≥470 ms calculated from 3 electrocardiograms (ECGs) using Frediricia's Correction or other significant ECG abnormality noted within 14 days of treatment.
- Clinically significant peripheral vascular disease or vascular disease, including rapidly growing aortic aneurysm or abdominal aortic aneurysm >5 cm or aortic dissection.
- Unstable angina.

2.3.2.14 History of cerebrovascular accident, transient ischemic attack within 1 year prior to study enrollment.

2.3.2.15 Significant hemorrhage (> 30 mL bleeding/episode within 3 months before study enrollment) or haemoptysis (> 5mL fresh blood within 28 days before study enrollment).

2.3.2.16 Current dependency on TPN or IV fluid hydration.

2.3.2.17 Any prior grade ≥ 3 immune-related adverse event (irAE)[160] while receiving any previous immunotherapy agent, or any unresolved irAE > grade 1. Toxicities of prior therapy, excepting alopecia, should be resolved to less than or equal to grade 1 as per NCI-CTCAEv4.0 (located on the CTEP website at http://ctep.cancer.gov/protocolDevelopment/electronic_applications/ctc.htm).

2.3.2.18 Uncontrolled intercurrent illness including, but not limited to, ongoing or active infection, symptomatic congestive heart failure, unstable angina pectoris, cardiac arrhythmia (except chronic atrial fibrillation with controlled vascular rate), active peptic ulcer disease, or psychiatric illness/social situations that would limit compliance with study requirements.

2.3.2.19 Pregnant and breastfeeding women are excluded from this study.

2.3.2.20 HIV-positive patients on antiretroviral therapy are ineligible because of potential pharmacokinetic interactions with study drugs. However, patients with long-standing (>5 years) HIV on antiretroviral therapy > 1 month (undetectable HIV viral load and CD4 count > 150 cells/µL) may be eligible if the PI determines no anticipated clinically significant drug-drug interactions.

2.3.2.21 HBV-or HCV-positive patients are ineligible because of potential reactivation of hepatitis virus following steroids.

2.3.2.22 Known history of previous clinical diagnosis of tuberculosis.

2.3.2.23 No baseline features suggestive of myelodysplastic syndrome or acute myelogenous leukemia on peripheral blood smear or bone marrow biopsy, if clinically indicated.

2.3.2.24 No prior or current evidence of coagulopathy or bleeding diathesis. Therapeutic anticoagulation for prior thromboembolic events is permitted.

2.3.2.25 Concurrent enrollment in another clinical study, unless it is an observational non-interventional clinical study or the follow-up of an interventional study.

2.3.2.26 Any concurrent chemotherapy, immunotherapy, biologic or hormonal therapy for cancer treatment. Concurrent use of hormones for non-cancer-related conditions (e.g., insulin for diabetes and hormone replacement therapy) is acceptable. NOTE: Local treatment of isolated lesions for palliative intent is acceptable (e.g., by local surgery or radiotherapy).

2.4 Phase II Study Non-Small Cell Lung Cancer (cohort 2; Durvalumab+O And Durvalumab+C) And Small Cell Lung Cancer (cohort 3; Durvalumab+O Only) Eligibility Criteria

2.4.1 Inclusion Criteria

Inclusion criteria (specific criteria for the NSCLC cohort)

2.4.1.1 Histologically or cytologically confirmed advanced NSCLC with at least one prior line of platinum-based chemotherapy (or treatment with EGFR, ALK, or BRAF-targeted tyrosine kinase inhibitors if tumors harbor an EGFR-sensitizing mutation, ALK translocation, or BRAF V600E mutation, respectively).

2.4.1.2 For Durvalumab+C NSCLC arm only, adequately controlled blood pressure (SBP < 140 mm Hg and DBP < 90mmHg) on a maximum of three antihypertensive medications.

2.4.1.3 Ability of subject to understand and the willingness to record twice-daily blood pressure readings if the patient is enrolled to the Durvalumab+C arm (NSCLC patients only

Inclusion criteria (specific criteria for the SCLC cohort)

2.4.1.4 Histologically or cytologically confirmed SCLC with at least one prior line of platinum-based chemotherapy are eligible. Patients with both platinum-sensitive and platinum-refractory disease will be eligible.

Inclusion criteria for both NSCLC and SCLC Cohorts

2.4.1.5 Presence of measurable disease as defined by RECIST v1.1. See Section 7.5 for the evaluation of measurable disease.

2.4.1.6 Patients must have at least one lesion deemed safe to biopsy and be willing to undergo a mandatory baseline biopsy.

2.4.1.7 Age ≥18 years.

2.4.1.8 ECOG performance status ≤ 2. (See **APPENDICES**

2.4.1.9 **Appendix** A)

2.4.1.10 Patients must have adequate organ and marrow function as defined below:

- absolute neutrophil count ≥ 1,500/mcL
- white blood cell (WBC) ≥ 3,000/mcL
- platelets ≥ 100,000/mcL
- hemoglobin (Hgb) ≥ 9 g/dL in the absence of packed red blood cell transfusion 28 days prior to dosing
- AST(SGOT)/ALT(SGPT) ≤ 2.5 X institutional upper limit of normal (ULN); for subjects with liver metastases, AST or ALT ≤ 5 × ULN
- Total bilirubin ≤ 1.5 × ULN; for subjects with documented/suspected Gilbert's disease, bilirubin ≤ 3 × ULN
- creatinine ≤ 1.5 X within normal institutional limits

  OR

  measured creatinine clearance ≥ 50 mL/min/1.73 m^2
- For NSCLC cohort Durvalumab-C arm only: Spot urine protein/creatinine ratio ≤ 1

  OR

  24 hour urine protein ≤ 1000 mg

2.4.1.11 Patients who have received anti-angiogenesis therapy are eligible, including but not limited to thalidomide, bevacizumab, sunitinib, sorafenib, or other anti-angiogenics. However, patients who were treated with cediranib, either in combination or monotherapy are not eligible.

2.4.1.12 Patients must be able to swallow oral medications (capsules and tablets) without chewing, breaking, crushing, opening or otherwise altering the product formulation. They should not have gastrointestinal illnesses that would preclude the absorption of cediranib or olaparib, which are oral agents.

2.4.1.13 Toxicities of prior therapy (excepting alopecia) should be resolved to less than or equal to Grade 1 as per CTCAE v 4.03 (http://ctep.cancer.gov/protocolDevelopment/electronic_applications/ctc.htm) except hemoglobin as shown in **2.4.1.10**. Patients with long-standing stable grade 2 neuropathy may be considered after discussion with the PI.

2.4.1.14 Ability of subject to understand and the willingness to sign a written informed consent document prior to any protocol related procedures, including screening evaluation.

2.4.1.15 Female patients must either be of non-reproductive potential (i.e., post-menopausal by history: ≥60 years old and no menses for ≥ 1 year without an alternative medical cause; OR history of hysterectomy, OR history of bilateral tubal ligation, OR history of bilateral oophorectomy) or must have a negative serum pregnancy test upon study entry and agree to use contraception or abstinence (See Section **2.10**) for female patients with reproductive potential. See Section **2.10** for male patients for contraception while on the study.

2.4.2 Exclusion criteria for both NSCLC and SCLC cohorts

2.4.2.1 Patients who have had prior PARP inhibitors. For this study, BSI-201 (iniparib) is not considered as PARPi.

2.4.2.2 Patients who have received chemotherapy, radiotherapy any other investigational agents within 3 weeks (6 weeks for nitrosoureas or mitomycin) prior to study enrollment are ineligible with the following exceptions:

2.4.2.2.1 SCLC patients who have received radiation therapy within 14 days before the first dose of study treatment.

2.4.2.2.2 NSCLC patients who have received Erlotinib, Afatinib, Osimertinib, Crizotinib, or Ceritinib within 14 days of the first dose of study treatment.

2.4.2.3 Patients receiving any medications or substances that are strong inhibitors or inducers of CYP3A4 are ineligible. A list of CYP3A4 inhibitors and inducers is provided in **Appendix B**. Dihydropyridine calcium-channel blockers are permitted for management of hypertension.

2.4.2.4 Major surgical procedure (as defined by the investigator) within 30 days prior to the first dose of Durvalumab or still recovering from prior surgery.

2.4.2.5 Patients with symptomatic brain metastases will be excluded from trial secondary to poor prognosis. However, patients who have had treatment for their brain metastasis and whose brain disease is stable without steroid therapy for 2 weeks may be enrolled. Baseline imaging to rule out brain metastases is not required for screening, but should be performed prior to study enrollment if clinically indicated.

2.4.2.6 Patients with history of a previous malignancy within the last 2 years are ineligible, except non-melanoma skin cancer, papillary carcinoma of the thyroid or non-invasive cancer of the uterine cervix. Eligibility of SCLC patients with inactive prior malignancy is a PI decision on an individual patient basis.

2.4.2.7 History of allergic reactions attributed to compounds of similar chemical or biologic composition to Durvalumab4736, olaparib, cediranib, or to other humanized monoclonal antibodies. Known history of anaphylaxis, angioedema, laryngeal edema, serum sickness, or uncontrolled asthma.

2.4.2.8 History of auto-immune disease requiring steroid maintenance, or history of primary immunodeficiency.

2.4.2.9 Patients with prior history of pneumonitis and/or interstitial lung disease will be excluded.

2.4.2.10 Current or prior use of immunosuppressive medication within 28 days before the first dose of Durvalumab, with the exception of intranasal and inhaled corticosteroids or systemic corticosteroids at physiological doses, which are not to exceed 10 mg/day of prednisone or an equivalent corticosteroid. In the case of short term use of systemic corticosteroids (less than 24 hours within 28 days) of greater than 10 mg/day of prednisone or an equivalent corticosteroid, the required washout period prior to starting the first dose of Durvalumab is 7 days.

2.4.2.11 Active or prior documented inflammatory bowel disease (e.g., Crohn's disease, ulcerative colitis). Eligibility for patients with asymptomatic and a previous diagnosis of immune or inflammatory colitis, or patients with chronic diarrhea > 1 month without immune or inflammatory colitis is a PI decision on an individual patient basis.

2.4.2.12 Receipt of live attenuated vaccination within 30 days before the first dose of Durvalumab.

2.4.2.13 Patients who have the following clinical conditions are considered to be at increased risk for cardiac toxicities. Patients with any cardiac history of the following conditions within 1 year prior to Durvalumab+O arm or within 2 years prior to Durvalumab+C arm enrollment are excluded from the study:

- Prior events including myocardial infarction, clinically signficant pericardial effusion, and myocarditis.
- Prior cardiac arrhythmia including atrial fibrillation (except chronic atrial fibrillation with controlled vascular rate) and atrial flutter, or requiring concurrent use of drugs or biologics with pro-arrhythmic potential.
- NYHA Class II or greater heart failure.
- If cardiac function assessment is clinically indicated or performed, an LVEF less than normal per institutional guidelines, or <55%, if threshold for normal is not otherwise specified by institutional guidelines.
- Mean QT interval corrected for heart rate (QTc) ≥470 ms calculated from 3 electrocardiograms (ECGs) using Frediricia's Correction or other significant ECG abnormality noted within 14 days of treatment.
- Hypertensive crisis or hypertensive encephalopathy.
- Clinically significant peripheral vascular disease or vascular disease, including rapidly growing aortic aneurysm or abdominal aortic aneurysm >5 cm or aortic dissection.
- Unstable angina.

2.4.2.14 History of cerebrovascular accident, transient ischemic attack within 1 year prior to study enrollment.

2.4.2.15 History of abdominal fistula, gastrointestinal perforation, or intra-abdominal abscess within 6 months prior to study enrollment.

2.4.2.16 Significant hemorrhage (> 30 mL bleeding/episode within 3 months before study enrollment) or hemoptysis (> 5mL fresh blood within 28 days before study enrollment).

2.4.2.17 Current signs and/or symptoms of bowel obstruction or signs and/or symptoms of bowel obstruction within 28 days before study enrollment.

2.4.2.18 Current dependency on total parenteral nutrition (TPN) or IV fluid hydration.

2.4.2.19 Any prior grade ≥ 3 immune-related adverse event (irAE)[160] while receiving any previous immunotherapy agent, or any unresolved irAE > grade 1. Toxicities of prior therapy, excepting alopecia, should be resolved to less than or equal to grade 1 as per NCI-CTCAEv4.0 (located on the CTEP website at http://ctep.cancer.gov/protocolDevelopment/electronic_applications/ctc.htm). Patients with greater than 1+ proteinuria at entry are ineligible.

2.4.2.20 Uncontrolled intercurrent illness including, but not limited to, ongoing or active infection, symptomatic congestive heart failure, unstable angina pectoris, cardiac arrhythmia (except chronic atrial fibrillation with controlled vascular rate), active peptic ulcer disease, or psychiatric illness/social situations that would limit compliance with study requirements.

2.4.2.21 Pregnant and breastfeeding women are excluded from this study.

2.4.2.22 HIV-positive patients on antiretroviral therapy are ineligible because of potential pharmacokinetic interactions with study drugs. However, patients with long-standing (>5 years) HIV on antiretroviral therapy > 1 month (undetectable HIV viral load and CD4 count > 150 cells/µL) may be eligible if the PI determines no anticipated clinically significant drug-drug interactions.

2.4.2.23 HBV-or HCV-positive patients are ineligible because of potential reactivation of hepatitis virus following steroids.

2.4.2.24 Known history of previous clinical diagnosis of tuberculosis.

2.4.2.25 No baseline features suggestive of myelodysplastic syndrome or acute myelogenous leukemia on peripheral blood smear or bone marrow biopsy, if clinically indicated.

2.4.2.26 No prior or current evidence of coagulopathy or bleeding diathesis. Therapeutic anticoagulation for prior thromboembolic events is permitted.

2.4.2.27 Concurrent enrollment in another clinical study, unless it is an observational non-interventional clinical study or the follow-up of an interventional study. Any concurrent chemotherapy, immunotherapy, biologic or hormonal therapy for cancer treatment. Concurrent use of hormones for non-cancer-related conditions (e.g., insulin for diabetes and hormone replacement therapy) is acceptable. NOTE: Local treatment of isolated lesions for palliative intent is acceptable (e.g., by local surgery or radiotherapy).

2.5 PHASE II STUDY METASTATIC CASTRATE-RESISTANT PROSTATE CANCER COHORT 4 ELIGIBILITY CRITERIA (DURVALUMAB+O ONLY)

2.5.1 Inclusion Criteria

2.5.1.1 Must have metastatic, progressive, castrate resistant prostate cancer (mCRPC).

2.5.1.2 Histopathological confirmation of prostate cancer by the Laboratory of Pathology of the NCI or Pathology Department of the Walter Reed National Military Medical Center is required prior to entering this study. Patients whose pathology specimens are no longer available may be enrolled if the patient has a clinical course that is consistent with prostate cancer and available documentation from an outside pathology laboratory of

the diagnosis. All efforts should be made to have the material forwarded to the research team for use in correlative studies in cases where original tissue blocks or archival biopsy material is available.

2.5.1.3 All patients must have at least one lesion deemed safe to biopsy and be willing to undergo a mandatory baseline biopsy.

2.5.1.4 Patients must have received prior treatment with enzalutamide and/or abiraterone with the exception of patients who were treated with docetaxel and androgen deprivation therapy for metastatic castrate-sensitive prostate cancer and progressed on docetaxel treatment or who progress within one month of the last docetaxel dose.

2.5.1.5 Patients must have castrate levels of testosterone (<50 ng/dl [1.74 nmol/l]).

2.5.1.6 Patients must have undergone bilateral surgical castration or must agree to continue on GnRH agonists/antagonists for the duration of the study.

2.5.1.7 ECOG performance status ≤ 2 (See **APPENDICES**

2.5.1.8 **Appendix** A)

2.5.1.9 Patients must have adequate bone marrow, hepatic, and renal function with:

• absolute neutrophil count	≥ 1,500/mcL
• white blood cell (WBC)	≥ 3,000/mcL
• platelets	≥ 100,000/mcL
• hemoglobin (Hgb)	≥ 9 g/dL in the absence of packed red blood cell transfusion 28 days prior to dosing
• AST(SGOT)/ALT(SGPT)	≤ 2.5 X institutional upper limit of normal (ULN); for subjects with liver metastases, AST or ALT ≤ 5 × ULN
• Total bilirubin	≤ 1.5 × ULN; for subjects with documented or suspected Gilbert's disease, bilirubin ≤ 3 × ULN
• creatinine	≤ 1.5 X within normal institutional limits OR measured creatinine clearance ≥ 50 mL/min/1.73 m^2

2.5.1.10 Men must be at least 18 years of age.

2.5.1.11 Patient must be capable of understanding and complying with protocol requirements and is willing to give informed consent.

2.5.1.12 Men treated or enrolled on this protocol must also agree to use adequate contraception prior to the study and for the duration of study participation and for 3 months after last dose of study drug. Sexually active subjects and their female partners must agree to use medically accepted barrier methods of contraception (eg, male or female condom) during the course of the study and for 3 months after the last dose of study drug(s), even

if oral contraceptives are also used. All subjects of reproductive potential must also agree to use both a barrier method and a second method of birth control during the course of the study and for 3 months after the last dose of study drug(s). Should a woman become pregnant or suspect she is pregnant while her partner is participating in this study, she should inform her treating physician immediately.

2.5.1.13 Patients who were treated for metastatic castrate-sensitive prostate cancer with docetaxel and androgen deprivation therapy who progress on docetaxel treatment or who progress within one month of the last docetaxel dose are eligible.

2.5.2 Exclusion Criteria

2.5.2.1 Patients who have had prior treatment with olaparib or other PARP inhibitors.

2.5.2.2 Patients who have had prior Durvalumab. Prior treatment with other immune checkpoint inhibitors is allowed.

2.5.2.3 The patient has received chemotherapy, radiotherapy, biologic agents or enzalutamide within 3 weeks before the first dose of study treatment (nitrosoureas or mitomycin within 6 weeks). However, for patients receiving abiraterone, they must discontinue the medication at least 14 days before the first dose of study treatment.

2.5.2.4 The patient has received any other type of investigational agent within 28 days before the first dose of study treatment.

2.5.2.5 The patient has received radionuclide treatment within 6 weeks prior to the first dose of the study treatment

2.5.2.6 The patient is unable to swallow tablets or capsules.

2.5.2.7 History of allergic reactions (known history of anaphylaxis, angioedema, laryngeal edema, serum sickness, or uncontrolled asthma) attributed to compounds of similar chemical or biologic composition to Durvalumab, olaparib, or to other humanized monoclonal antibodies.

2.5.2.8 History of auto-immune disease requiring steroid maintenance, or history of primary immunodeficiency.

2.5.2.9 Current or prior use of immunosuppressive medication within 28 days before the first dose of Durvalumab, with the exception of intranasal, inhaled, opthomalogical, or topical corticosteroids or systemic corticosteroids at physiological doses, which are not to exceed 10 mg/day of prednisone or an equivalent corticosteroid. In the case of short term use of systemic corticosteroids (less than 24 hours within 28 days) of greater than 10 mg/day of prednisone or an equivalent corticosteroid, the required washout period prior to starting the first dose of Durvalumab is 7 days.

2.5.2.10 Patients with any other concomitant or prior invasive malignancies are ineligible, However, patients with prior cancer treated with a curative intent with no evidence of recurrent disease 2 years following diagnosis and judged by the investigator to be at low risk of recurrence are eligible. Patients with treated limited stage basal cell or squamous cell carcinoma of the skin or carcinoma in situ of the breast are eligible.

2.5.2.11 Active or prior documented inflammatory bowel disease (e.g., Crohn's disease, ulcerative colitis). Eligibility for patients with asymptomatic and a previous diagnosis of immune or inflammatory colitis, or patients with chronic diarrhea > 1 month without immune or inflammatory colitis is a PI decision on an individual patient basis.

2.5.2.12 Receipt of live attenuated vaccination within 30 days before the first dose of Durvalumab.

2.5.2.13 Patients with evidence of CNS metastasis, or leptomeningeal disease within 1 year prior to enrollment will be excluded from this clinical trial because of their poor prognosis and because they often develop progressive neurologic dysfunction that would confound the evaluation of neurologic and other adverse events. Patients with a remote history of brain metastases may be considered if they received sterilizing therapy to the CNS (resection or radiation) and have been CNS progression-free for the 1-year period. Baseline imaging to rule out brain metastases is not required for screening, but should be performed prior to study enrollment if clinically indicated. Patients with spinal cord compression may be considered if the condition is medically managed and currently asymptomatic.

2.5.2.14 The patient has not recovered to baseline or CTCAE ≤ Grade 1 from toxicity due to all prior therapies, including surgery, except alopecia and other non-clinically significant AEs.

2.5.2.15 The patient has uncontrolled, significant intercurrent or recent illness including, but not limited to, the following conditions:

a. Cardiovascular disorders including

i. congestive heart failure (CHF): New York Heart Association (NYHA) Class III (moderate) or Class IV (severe) at the time of screening

ii. concurrent uncontrolled hypertension defined as sustained BP >140 mm Hg systolic, or > 90 mm Hg diastolic despite optimal antihypertensive treatment (BP must be controlled at screening)

iii. any history of congenital long QT syndrome

iv. any of the following within 6 months before the first dose of study treatment:

- unstable angina pectoris
- clinically-significant cardiac arrhythmias with the exception of asymptomatic atrial fibrillation controlled on therapy
- stroke (including TIA, or other ischemic event)
- myocardial infarction
- thromboembolic event requiring therapeutic anticoagulation (Note: subjects with a venous filter (e.g. vena cava filter) are not eligible for this study)
- Mean QT interval corrected for heart rate (QTc) ≥470 ms calculated from 3 electrocardiograms (ECGs) using Fredericia's Correction or other significant ECG abnormality noted within 14 days of treatment.

b. Other clinically significant disorders such as:

i. active infection requiring intravenous treatment within 7 days of starting protocol treatment

ii. serious non-healing wound/ulcer/bone fracture (excluding stable compression fracture) within 28 days before the first dose of study treatment

2.5.2.16 HIV-positive patients on combination antiretroviral therapy are ineligible because of the potential for pharmacokinetic interactions with the study agent. However, patients with long-standing (>5 years) HIV on antiretroviral therapy > 1 month (undetectable HIV viral load and CD4 count > 150 cells/µL) may be eligible if the PI determines no anticipated clinically significant drug-drug interactions.

2.5.2.17 HBV-or HCV-positive patients are ineligible

2.5.2.18 Any prior grade ≥ 3 immune-related adverse event (irAE)[160] while receiving any previous immunotherapy agent, or any unresolved irAE > grade 1.

2.6 PHASE II COLORECTAL CANCER COHORT 6 (DURVALUMAB+C ONLY)

2.6.1 Inclusion criteria

2.6.1.1 Histologically or cytologically confirmed advanced colorectal cancer. Patients must have progressed on, been intolerant of or refused prior oxaliplatin- and irinotecan-containing chemotherapeutic regimen, and have disease that is not amenable to potentially curative resection. Patients who have a known KRAS (or NRAS or BRAF) wild type tumor must have progressed, been intolerant of or refused cetuximab or panitumumab-based chemotherapy.

2.6.1.2 Patients are allowed to have received prior anti-angiogenesis therapy with the exception of prior cediranib.

2.6.1.3 Patients must be MSI-stable (or low).

2.6.1.4 Patients must have at least one focus of metastatic disease that is amenable to pre- and on-treatment biopsy.

2.6.1.5 All patients must have measurable disease.

2.6.1.6 Age ≥18 years.

2.6.1.7 ECOG performance status ≤ 2. (See **APPENDICES**

2.6.1.8 **Appendix** A)

2.6.1.9 Patients must have adequate organ and marrow function as defined below:

• absolute neutrophil count	≥ 1,500/mcL
• white blood cell (WBC)	≥ 3,000/mcL
• platelets	≥ 100,000/mcL
• hemoglobin (Hgb)	≥ 9 g/dL in the absence of packed red blood cell transfusion 28 days prior to dosing
• AST(SGOT)/ALT(SGPT)	≤ 2.5 X institutional upper limit of normal (ULN); for subjects with liver metastases, AST or

ALT ≤ 5 × ULN

- Total bilirubin ≤ 1.5 × ULN; for subjects with documented/suspected Gilbert's disease, bilirubin ≤ 3 × ULN
- creatinine ≤ 1.5 X within normal institutional limits

OR

measured creatinine clearance ≥ 50 mL/min/1.73 m^2

- Spot urine protein/creatinine ratio ≤ 1

OR

24 hour urine protein ≤ 1000 mg

2.6.1.10 Patients must be able to swallow oral medications without chewing, breaking, crushing, opening or otherwise altering the product formulation. They should not have gastrointestinal illnesses that would preclude the absorption of cediranib which are an oral agent.

2.6.1.11 Adequately controlled blood pressure (SBP < 140 mm Hg and DBP < 90mmHg) on a maximum of three antihypertensive medications.

2.6.1.12 Toxicities of prior therapy (excepting alopecia) should be resolved to less than or equal to Grade 1 as per CTCAE v 4.03 (http://ctep.cancer.gov/protocolDevelopment/electronic_applications/ctc.htm except hemoglobin as shown in **2.6.1.9**Patients with long-standing stable grade 2 neuropathy may be considered after discussion with the PI.

2.6.1.13 Ability of subject to understand and the willingness to sign a written informed consent document prior to any protocol related procedures, including screening evaluations.

2.6.1.14 Ability of subject to understand and the willingness to record twice-daily blood pressure readings.

2.6.1.15 Female patients must either be of non-reproductive potential (i.e., post-menopausal by history: ≥60 years old and no menses for ≥1 year without an alternative medical cause; OR history of hysterectomy, OR history of bilateral tubal ligation, OR history of bilateral oophorectomy) or must have a negative serum pregnancy test upon study entry and agree to use contraception or abstinence (See Section **2.10**) for female patients with reproductive potential. See Section **2.10** for male patients for contraception while on the study.

2.6.2 Exclusion criteria

2.6.2.1 Patients who have received chemotherapy, radiotherapy, any other investigational agents within 3 weeks (6 weeks for nitrosoureas or mitomycin) prior to study enrollment.

2.6.2.2 Patients who were previously treated with cediranib.

2.6.2.3 Patients who have had prior immune checkpoint inhibitors, such as Durvalumab or other PD1 or PD-L1 inhibitors or an anti-CTLA4 therapy.

2.6.2.4 Patients receiving any medications or substances that are strong inhibitors or inducers of CYP3A4 are ineligible. A list of CYP3A4 inhibitors and inducers is provided in **Appendix B**. Dihydropyridine calcium-channel blockers are permitted for management of hypertension.

2.6.2.5 Major surgical procedure (as defined by the investigator) within 30 days prior to the first dose of Durvalumab or still recovering from prior surgery.

2.6.2.6 Patients with any other concomitant or prior invasive malignancies are ineligible, However, patients with prior cancer treated with a curative intent with no evidence of recurrent disease 5 years following diagnosis and judged by the investigator to be at low risk of recurrence are eligible. Patients with treated limited stage basal cell or squamous cell carcinoma of the skin or carcinoma in situ of the breast or cervix are eligible.

2.6.2.7 CNS disease: Subjects with symptomatic brain metastases will be excluded from trial secondary to poor prognosis. However, subjects who have had treatment for their brain metastasis and whose brain disease is stable without steroid therapy for 4 weeks may be enrolled. Baseline imaging to rule out brain metastases is not required for screening, but should be performed prior to study enrollment if clinically indicated.

2.6.2.8 History of allergic reactions attributed to compounds of similar chemical or biologic composition to Durvalumab, cediranib, or to other humanized monoclonal antibodies. Known history of anaphylaxis, angioedema, laryngeal edema, serum sickness, or uncontrolled asthma.

2.6.2.9 History of auto-immune disease requiring steroid maintenance, or history of primary immunodeficiency.

2.6.2.10 Patients with prior history of pneumonitis and/or interstitial lung disease will be excluded.

2.6.2.11 Current or prior use of immunosuppressive medication within 28 days before the first dose of Durvalumab, with the exception of intranasal and inhaled corticosteroids or systemic corticosteroids at physiological doses, which are not to exceed 10 mg/day of prednisone or an equivalent corticosteroid. In the case of short term use of systemic corticosteroids (less than 24 hours within 28 days) of greater than 10 mg/day of prednisone or an equivalent corticosteroid, the required washout period prior to starting the first dose of Durvalumab is 7 days.

2.6.2.12 Active or prior documented inflammatory bowel disease (e.g., Crohn's disease, ulcerative colitis). Eligibility for patients with asymptomatic and a previous diagnosis of immune or inflammatory colitis, or patients with chronic diarrhea > 1 month without immune or inflammatory colitis is a PI decision on an individual patient basis.

2.6.2.13 Receipt of live attenuated vaccination within 30 days before the first dose of Durvalumab.

2.6.2.14 Patients who have the following clinical conditions are considered to be at increased risk for cardiac toxicities. Patients with any cardiac history of the following conditions within 2 years prior to study enrollment are excluded from the study:

- Prior events including myocardial infarction, clinically significant pericardial effusion, and myocarditis.

- Prior cardiac arrhythmia including atrial fibrillation and atrial flutter, or requiring concurrent use of drugs or biologics with pro-arrhythmic potential.
- NYHA Class II or greater heart failure.
- If cardiac function assessment is clinically indicated or performed, an LVEF less than normal per institutional guidelines, or <55%, if threshold for normal is not otherwise specified by institutional guidelines.
- Mean QT interval corrected for heart rate (QTc) ≥470 ms calculated from 3 electrocardiograms (ECGs) using Fredricia's Correction or other significant ECG abnormality noted within 14 days of treatment.
- Hypertensive crisis or hypertensive encephalopathy.
- Clinically significant peripheral vascular disease or vascular disease, including rapidly growing aortic aneurysm or abdominal aortic aneurysm >5 cm or aortic dissection.
- Unstable angina.

2.6.2.15 History of cerebrovascular accident, transient ischemic attack within 1 year prior to study enrollment.

2.6.2.16 History of abdominal fistula, gastrointestinal perforation, or intra-abdominal abscess within 6 months prior to study enrollment.

2.6.2.17 Significant hemorrhage (> 30 mL bleeding/episode within 3 months before study enrollment) or hemoptysis (> 5mL fresh blood within 28 days before study enrollment).

2.6.2.18 Current signs and/or symptoms of bowel obstruction or signs and/or symptoms of bowel obstruction within 28 days before study enrollment.

2.6.2.19 Current dependency on total parenteral nutrition (TPN) or IV fluid hydration.

2.6.2.20 Any prior grade ≥ 3 immune-related adverse event (irAE)[160] while receiving any previous immunotherapy agent, or any unresolved irAE > grade 1. Toxicities of prior therapy, excepting alopecia, should be resolved to less than or equal to grade 1 as per NCI-CTCAEv4.0 (located on the CTEP website at http://ctep.cancer.gov/protocolDevelopment/electronic_applications/ctc.htm). Patients with greater than 1+ proteinuria at entry are ineligible.

2.6.2.21 Uncontrolled intercurrent illness including, but not limited to, ongoing or active infection, symptomatic congestive heart failure, unstable angina pectoris, cardiac arrhythmia, active peptic ulcer disease, or psychiatric illness/social situations that would limit compliance with study requirements.

2.6.2.22 Pregnant and breastfeeding women are excluded from this study.

2.6.2.23 HIV-positive patients on antiretroviral therapy are ineligible because of potential pharmacokinetic interactions with study drugs. However, patients with long-standing (>5 years) HIV on antiretroviral therapy > 1 month (undetectable HIV viral load and CD4 count > 150 cells/µL) may be eligible if the PI determines no anticipated clinically significant drug-drug interactions.

2.6.2.24 HBV-or HCV-positive patients are ineligible because of potential reactivation of hepatitis virus following steroids.

2.6.2.25 Known history of previous clinical diagnosis of tuberculosis.

2.6.2.26 No baseline features suggestive of myelodysplastic syndrome or acute myelogenous leukemia on peripheral blood smear or bone marrow biopsy, if clinically indicated.

2.6.2.27 No prior or current evidence of coagulopathy or bleeding diathesis. Therapeutic anticoagulation for prior thromboembolic events is permitted.

2.6.2.28 Concurrent enrollment in another clinical study, unless it is an observational non-interventional clinical study or the follow-up of an interventional study. Any concurrent chemotherapy, immunotherapy, biologic or hormonal therapy for cancer treatment. Concurrent use of hormones for non-cancer-related conditions (e.g., insulin for diabetes and hormone replacement therapy) is acceptable. NOTE: Local treatment of isolated lesions for palliative intent is acceptable (e.g., by local surgery or radiotherapy).

2.6.3 Recruitment Strategies

This study will be posted on NIH websites and on NIH social media forums. Participants may also be identified through referrals from physicians, or from populations in NIH Clinics.

2.7 SCREENING EVALUATION

2.7.1 Screening activities performed prior to obtaining informed consent

Minimal risk activities that may be performed before the subject has signed a consent include the following:

- Email, written, in person or telephone communications with prospective subjects
- Review of existing medical records to include H&P, laboratory studies, etc.
- Review of existing MRI, x-ray, or CT images
- Review of existing photographs or videos
- Review of existing pathology specimens/reports from a specimen obtained for diagnostic purposes

A waiver of consent for these activities has been requested in section **13.6.2**

2.7.2 Screening activities performed after a consent for screening has been signed

The following activities will be performed only after the subject has signed the study consent OR the consent for study 01-C-0129 (provided the procedure is permitted on that study) on which screening activities may also be performed. Screening requirements are described below, and in the Study Calendar (Section **3.5**).

History and Physical, Height, and Weight

Medical history and physical examination will be conducted at screening and at subsequent visits as indicated in the schedule of assessments. Results of the physical examination including any abnormalities will be documented and should be obtained within 14 days prior to initiation of therapy. Abnormal findings will be reassessed at subsequent visits.

- o Body weight will be measured at screening and at subsequent visits as indicated in the schedule of assessments and documented in the clinical record.
- o Body height will be measured at screening, or at subsequent visit before starting study treatment.

- On subsequent visits, any newly diagnosed or worsening conditions, signs and symptoms, whether related or unrelated to the trial, will be recorded as adverse events.

Performance Status

The ECOG performance status will be assessed at screening and at subsequent visits as indicated in the schedule of assessments and documented in the clinical record.

Pathology confirmation of diagnosis at screening.

CT scans for disease evaluation at screening.

- Except in mCRPC patients, scan should be obtained within 17 days prior to initiation of therapy. If the screening scan has been obtained within this timeframe, it may be used for baseline purposes.
- mCRPC patients will have baseline CT scans and technetium-99 bone scan imaging within 30 days prior to initiation of therapy.

Laboratory Studies

Laboratory studies except viral titers should be obtained within 10 days prior to initiation of therapy.

Laboratory studies to include:

- CBC with differential, PT/INR/PTT
- Biochemical profile:
- Screening: AST (SGOT), ALT (SGPT), total bilirubin, serum creatinine, spot urine protein creatinine ratio (UPCR), and TSH.
- Anti-HIV, anti-HCV, Hepatitis B surface Ag, and anti-CMV: viral titers will be obtained within 28 days.
- Baseline - 14 comprehensive metabolic panel (sodium, potassium, chloride, carbon dioxide, BUN, creatinine or measured creatinine clearance, glucose, AST, ALT, bilirubin, calcium, total protein, albumin, alkaline phosphatase), magnesium, urinalysis.

2.7.2.1 Additional laboratory studies for mCRPC cohort 4 (Phase II Durvalumab+O) baseline assessment and monitoring:

- LDH, Creatine kinase, Uric Acid, Total Protein
- Serum PSA level
- Serum testosterone (baseline only, if applicable)

2.7.2.2 Additional laboratory studies for SCLC cohort 3 (Phase II Durvalumab+O) baseline assessment and monitoring:

- Complete neurological examination including assessment of cranial nerve function and muscle strength will be performed at screening and subsequent visits on all eligible patients before starting treatment.
- Additional blood tests to be done at baseline include

- Creatine kinase, serum aldolase, and anti-acetylcholine receptor antibody titers.
- One additional red top tube (10 ml) will be collected and frozen at baseline for future evaluation of paraneoplastic autoantibodies. The antibodies of interest include Anti-Hu, Anti-Ri, Anti-amphiphysin, antineuronal antibodies Ma1 and Ma2 and anti-Yo or anti-Purkinje cell antibody. Since these antibodies are not truly paraneoplastic antibodies, as they can also occur in the non-paraneoplastic setting, but their baseline levels will be of use in the work-up of patients who develop paraneoplastic syndromes on the trial.

2.7.2.3 Additional laboratory studies for CRC cohort 6 (Phase II C+Durvalumab) baseline assessment and monitoring:

1. CEA level

An EKG should be obtained within 7 days prior to initiation of therapy.

CA125 (Ovarian cancer patients only)

CA125 will be obtained at screening and at subsequent visits from all ovarian cancer patients in whom it has been shown to be abnormal. CA125 will not be used in any patients for disease assessment decisions.

Pregnancy Testing

For female subjects of childbearing potential, urine or serum HCG will be performed on initial screening.

- A urine beta-HCG will be performed before each administration of Durvalumab during the treatment phase, at the end-of-treatment visit, and at the post-treatment follow-up visit.
- Patients who are postmenopausal (age-related amenorrhea for 12 or more consecutive months, or documented FSH > 40 mIU/mL), or who had undergone hysterectomy or bilateral oophorectomy are exempt from pregnancy testing.

2.8 PARTICIPANT REGISTRATION AND STATUS UPDATE PROCEDURES

Registration and status updates (e.g. when a participant is taken off protocol therapy and when a participant is taken off-study) will take place per CCR SOP ADCR-2, CCR Participant Registration & Status Updates found **here**.

2.8.1 Screen Failures

Screen failures are defined as participants who consent to participate in the clinical trial but are not subsequently assigned to the study intervention or entered in the study. A minimal set of screen failure information is required to ensure transparent reporting of screen failure participants, to meet the Consolidated Standards of Reporting Trials (CONSORT) publishing requirements and to respond to queries from regulatory authorities. Minimal information includes demography, screen failure details, eligibility criteria, and any serious adverse event (SAE).

Individuals who do not meet the criteria for participation in this trial (screen failure) may not be rescreened.

2.9 TREATMENT ASSIGNMENT AND RANDOMIZATION/STRATIFICATION PROCEDURES

Cohorts

Number	Name	Description
1	P1 Cohort 1	Patients with advanced solid tumors
2	P2 Cohort 1	Patients with ovarian cancer
3	P2 Cohort 2	Patients with non-small cell lung cancer
4	P2 Cohort 3	Patients with small cell lung cancer
5	P2 Cohort 4	Patients with metastatic castrate-resistant prostate cancer
6	P2 Cohort 5	Patients with triple negative breast cancer
7	P2 Cohort 6	Patients with colorectal cancer

Arms

Number	Name	Description
1	P1 Durvalumab+O	Ph I Durvalumab + olaparib dose escalation
2	P1 Durvalumab+C	Ph I Durvalumab + cediranib dose escalation
3	P2 Durvalumab+O	Ph II Durvalumab + olaparib at RP2D
4	P2 Durvalumab+C	Ph II Durvalumab + cediranib at RP2D
5	P1 Durvalumab+O+C	Ph I Durvalumab + olaparib + cediranib dose escalation

Number	Name	Description
6	P2 Durvalumab+O+C	Ph II Durvalumab + olaparib + cediranib at RP2D

Randomization and stratification procedures do not apply to the Phase I and Phase II portions of the study. The arms will be open concurrently and treatment assignment for each patient will be at the PI's discretion and/or based on prior treatment history.

Phase I

The Phase I dose escalation study will accrue patients to each arm in non-randomized fashion. The CRO will contact a medically-responsible study investigator in the Clinical Center Pharmacy (301-496-8092) to confirm each patient's Phase I treatment assignment.

Phase II

Effective with Amendment C (version date 12/28/2015), Durvalumab+O accrual will move to the stage 1 portion of Phase II study Durvalumab+O.

Effective with Amendment H (version date 07/07/2016), Durvalumab+C accrual will move to the stage 1 portion of Phase II study as described in Section **11**.

Effective with Amendment M (version date 7/19/2017), Durvalumab+O+C accrual will move to the stage 1 portion of Phase II study as described in Section 8.

Effective with Amendment O (version date 7/25/2018), the Phase II Durvalumab+O OvCa and SCLC cohorts were closed due to completed enrollment; the Phase II Durvalumab+C NSCLC cohort was closed due to slow accrual; and the Phase II Durvalumab+C CRC cohort was closed due to completed enrollment, as described in Section **11**.

- Subjects in the Phase II study OvCa cohort 1 will be allocated to 3 possible treatment arms at the PI's discretion and/or based on their prior treatment history:
  1) Durvalumab+O arm: Durvalumab4736 in combination with olaparib, or
  2) Durvalumab+C arm: Durvalumab in combination with cediranib, or
  3) Durvalumab+O+C arm: Durvalumab in combination with olaparib and cediranib
  Effective with Amendment O (version date 7/25/2018), the Phase II Durvalumab+O OvCa cohort is closed due to completed enrollment.

- Subjects in the Phase II study NSCLC cohort 2 will be allocated to 2 possible arms at the PI's discretion and/or based on their prior treatment history:
  1) Durvalumab+O: Durvalumab in combination with olaparib, or
  2) Durvalumab+C: Durvalumab in combination with cediranib
  Effective with Amendment O (version date 7/25/2018), the Phase II Durvalumab+C NSCLC cohort is closed due to slow accrual.

- Subjects in the Phase II SCLC (cohort 3), mCRPC (cohort 4) and TNBC (cohort 5) cohorts will be assigned to only Durvalumab+O arm (Durvalumab 1500mg IV every 28 days in combination with olaparib 300mg tablets bid daily)
  Effective with Amendment O (version date 7/25/2018), the Phase II Durvalumab+O SCLC cohort is closed due to completed enrollment.

- For patients in the Phase II TNBC cohort 5, documentation for gBRCAm status will be required prior to enrollment (See Section 8). For those who have no gBRCAm testing results, the study team will contact the referring physician or the health insurance company, or other resources if necessary, to get a testing performed prior to enrollment.

- Subjects in the Phase II study CRC cohort 6 will be allocated to C+Durvalumab arm (cediranib alone followed by Durvalumab in combination with cediranib). As a pilot biomarker study, the CRC patients will start cediranib alone for 14 days (cycle 1 day – (minus) 14 to -1) then begin Durvalumab with cediranib on cycle 1 day 1. Of note, there is no cycle 1 day 0. Effective with Amendment O (version date 7/25/2018), the Phase II Durvalumab+C CRC cohort is closed due to completed enrollment.

Figure 9 Schema for CRC Cohort 6

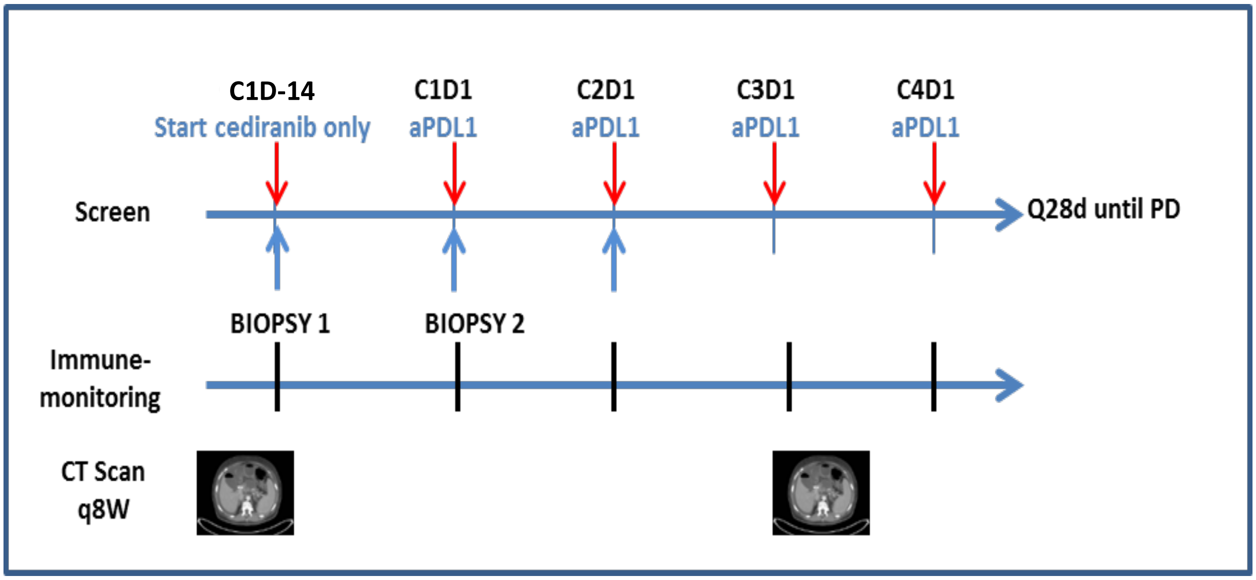

The CRO will send an email confirmation of the registration and cohort assignment to the person initiating the registration immediately following the registration and/or treatment assignment. If only one arm warrants further investigation, accrual will proceed without continuing another treatment arm. If accrual to the stage 2 portion is warranted in both treatment arms, the CRO will also contact the Clinical Center Pharmacy (301-496-8092) to identify each patient’s treatment assignment.

2.10 RESTRICTIONS DURING THE STUDY

Contraception

- Females of childbearing potential who are sexually active with a nonsterilized male partner must use 2 methods of effective contraception from screening, and must agree to continue using such precautions for 90 days after the final dose of any study-related medications (olaparib, cediranib, and Durvalumab); cessation of birth control after this point should be discussed with a responsible physician. Periodic abstinence, the rhythm method, and the withdrawal method are not acceptable methods of birth control.
- Females of childbearing potential are defined as those who are not surgically sterile (i.e., bilateral tubal ligation, bilateral oophorectomy, or complete hysterectomy) or postmenopausal (defined as 12 months with no menses without an alternative medical cause). Subjects must use 2 acceptable methods of effective contraception as described in **Table 5**.
- Nonsterilized males who are sexually active with a female partner of childbearing potential must use 2 acceptable methods of effective contraception (see **Table 5**) from day 1 and for 90 days after receipt of the final dose of investigational product.

Table 5 Effective methods of contraception (two methods must be used)

Barrier Methods	Intrauterine Device Methods	Hormonal Methods
Male condom plus spermicide	Copper T	Implants
Cap plus spermicide	Progesterone T[a]	Hormone shot or injection
Diaphragm plus spermicide	Levonorgestrel-releasing intrauterine system (e.g., Mirena®)[a]	Combined pill
		Minipill
		Patch

[a] This is also considered a hormonal method.

Blood donation

Patients should not donate blood while participating in this study or for 90 days following the last infusion of Durvalumab.

3 STUDY IMPLEMENTATION

3.1 STUDY DESIGN

This is an open label Phase I/II trial, simultaneously accruing three cohorts to determine the RP2D of Durvalumab+O, Durvalumab+C and Durvalumab+O+C in patients with advanced solid tumors (Phase I), and to examine the safety and clinical activity of these combinations in patients with advanced or recurrent ovarian, lung, prostate, triple negative breast and colorectal cancers (Phase II cohorts 1-6). Patients will be dosed in 28-day cycles.

Administration of Durvalumab may be given one day (for every 2 weeks schedule) or 4 days (for every 4 weeks schedule) earlier or delayed up to 7 days due to holidays, inclement weather, conflicts, or similar reasons. The timing of subsequent administrations is then adjusted to maintain a 28 day-interval for a fixed dose schedule or a 14 day-interval for every 2 weeks schedule dose levels.

The phase I of Durvalumab+O, Durvalumab+C and Durvalumab+O+C will dose escalate simultaneously. This simultaneous allocation of patients in the phase I study will allow us to accrue patients as efficiently as possible so that we have the dosing for the phase II study in a timely manner.

Effective with Amendment E (version date 03/17/2016), the patients on Phase I study Durvalumab every 2 weeks schedule dose levels may change Durvalumab schedule to every 4 weeks (a fixed dose 1500mg) if they have clinical benefit (CR/PR/SD) more than 1 year.

Phase I

3.1.1.1 Dose Limiting Toxicity (DLT)

The DLT period is one cycle, 28 days. DLT is defined as any of the following:

- Non-Hematologic dose-limiting toxicity
    - Grade 3 or higher adverse events (AEs), ***excluding***
        - Grade 3 hypertension controlled with anti-hypertensive therapy, or grade 3 asymptomatic electrolytes imbalance with optimal and continuing repletion that downgrades to grade 1 or better within 3 days after onset of the event.
        - Grade 3 endocrinopathy that is managed with or without systemic corticosteroid therapy and/or hormone replacement therapy and the patient is asymptomatic.
        - Grade 3 inflammatory reaction attributed to a local antitumor response (e.g., inflammatory reaction at sites of metastatic disease, lymph node, etc.).
        - Grade 3 asymptomatic increase in gamma glutamyl transferase (GGT) OR grade 3 asymptomatic increase in amylase or lipase that downgrades to grade1 or better within 7 days after onset of the event.

    - Persistent (>14 days) non-hematologic Grade 2 AEs despite optimal medical management **and** treatment delay > 14 days.

- Hematologic Toxicity
    - Grade ≥ 4 neutropenia of ≥ 5 days duration, grade 4 neutropenia of any duration with fever or documented infection, all other grade 4 hematologic toxicities.
    - **Exceptions**:
        - Grade 3 lymphopenia or leukopenia that downgrades to grade 2 or better within 14 days after onset of the event, and resolves to grade 1 or better, OR baseline within 14 days.
        - Grade ≥ 3 lymphopenia, or leukopenia in the absence of grade 3 or higher neutropenia.

3.1.1.2 Dose Escalation

Dose escalation will proceed in cohorts of 3–6 patients. The MTD is the dose level at which no more than 1 of up to 6 patients experience DLT during cycle 1 of treatment, and the dose below that at which at least 2 (of ≤6) patients have DLT as a result of the drug. If a patient does not finish cycle 1 for any reason other than DLT, the patient will not be evaluable for DLT and will be replaced in the dose level.

Table 6. Durvalumab+O Dose Escalation Schedule

**Dose Level (DL)**	**Durvalumab (intravenously)**	**Olaparib tablets (oral, twice a day)**
**DL** -2	3 mg/kg every 2 weeks	150 mg
**DL** -1	3 mg/kg every 2 weeks	200 mg
**DL** 1 (starting dose)	10 mg/kg every 2 weeks	200 mg
**DL** 2	10 mg/kg every 2 weeks	300 mg
**DL** 3	A fixed dose of 1500mg every 4 weeks	300 mg

Table 7. Durvalumab-C Daily Schedule Dose Escalation Table

**Dose Level (DL)**	**Durvalumab (intravenously)**	**Cediranib (oral, once daily)**
**DL** -2	3 mg/kg every 2 weeks	15 mg
**DL** -1	3 mg/kg every 2 weeks	20 mg
**DL** 1 (starting dose)	10 mg/kg every 2 weeks	20 mg
**DL** 2	10 mg/kg every 2 weeks	30 mg

Table 8. Durvalumab+C Intermittent Schedule Dose Escalation Table

Dose Level (DL)	Durvalumab (intravenously)	Cediranib (oral, 5 days on/2 days off)
DL -2	A fixed dose of 500mg every 4 weeks	15 mg
DL -1	A fixed dose of 1500mg every 4 weeks	15 mg
**DL 1 (starting dose)**	**A fixed dose of 1500mg every 4 weeks**	**20 mg**

Table 9. Durvalumab+O+C Dose Escalation Table

Dose Level (DL)	Durvalumab (intravenously)	Olaparib tablet (oral, twice a day)	Cediranib (oral, 5 days on/ 2 days off)
DL -1	A fixed dose of 1500 mg every 4 weeks	200 mg	15 mg
**DL 1 (starting dose)**	A fixed dose of 1500 mg every 4 weeks	300 mg	15 mg
DL 2	A fixed dose of 1500 mg every 4 weeks	300 mg	20 mg

Table 10. Dose Escalation Decisions

Number of patients with DLT at a given dose level (DL)	Escalation Decision Rule
0 out of 3	Enter up to 3 patients at the next DL. If 0 of 3 have DLT at DL 2, a total of 6 patients per arm will be enrolled, and if $\leq$1 of 6 experience DLT, this will be considered the RP2D although it may not be the MTD.

Number of patients with DLT at a given dose level (DL)	Escalation Decision Rule
≥ 2	Dose escalation will be stopped. This DL will be declared the maximally administered dose (highest dose administered). Up to three (3) additional patients will be entered at the next lowest DL if two of from two to six patients experience DLT at DL 1.
1 out of 3	Enter 3 more patients at this DL.<br>• If 0 of these 3 patients experience DLT, proceed to the next DL.<br>• If 1 of 3 additional patients on DL1 experience DLT, then dose escalation is stopped, and this dose is declared the maximally administered dose.<br>• Three additional patients will be entered at the next lowest DL if two of from two to six patients experience DLT at DL1.
≤1 out of 6 at highest DL	This is generally the recommended phase 2 dose. At least 6 patients must be treated at the recommended phase 2 dose.

Phase II

The phase II of Durvalumab+O, Durvalumab+C or C+Durvalumab and Durvalumab+O+C will be examined in different tumor types simultaneously. Randomization procedures do not apply to the Phase II OvCa cohort 1 and Phase II NSCLC cohort 2. The treatment arms will be open concurrently and treatment assignment for each patient will be at the PI's discretion and/or based on prior treatment history.

3.1.1.3 Phase II OvCa Cohort 1 Stage 1

- For patients with OvCa, 12 evaluable patients will be enrolled to the appropriate doublet treatment arm once RP2D is determined for each cohort at the PI's discretion
- For patients with OvCa, 17 evaluable will be enrolled to the triplet treatment arm once RP2D is determined at the PI's discretion

3.1.1.4 Phase II OvCa Cohort 1 Stage 2

For patients with OvCa, stage 2 for an active arm will begin after stage 1 has been completed. If the minimum level of activity is not observed in stage 1 for either one or both arms, no further enrollment will occur into that arm (see Section 8). For Durvalumab+O and Durvalumab+C arms, 23 additional evaluable patients will be accrued in each arm if at least 2 of the 12

evaluable subjects from Stage 1 demonstrate an objective response (see Section **11**). For Durvalumab+O+C arm, 20 additional evaluable patients will be accrued in triplet arm if at least 4 of the 17 evaluable subjects from Stage 1 demonstrate an objective response (see Section **11)**. A total of 107 evaluable patients will be allocated to receive Durvalumab+C or Durvalumab+O or Durvalumab+O+C at RP2D without stratification.

3.1.1.5 Phase II Other tumor types (See Section 8)

- For patients with NSCLC (cohort 2),
  37 evaluable patients will be assigned to Durvalumab+O and 37 evaluable patients will be assigned to Durvalumab+C at PI's discretion, without randomization.
- For patients with SCLC (cohort 3),
  - Stage 1: 19 evaluable patients will be enrolled to Durvalumab+O only, and if 0 to 3 of the first 19 have a response, then no further patients will be accrued in this cohort.
  - Stage 2: If 4 or more of the first 19 patients have a response, then accrual would continue until a total of 33 patients have been enrolled.
- For patients with mCRPC (cohort 4),
  - 25 evaluable patients will be enrolled to Durvalumab+O only.
  - An early stopping rule will be implemented: if the 4 month PFS is 50% or less, after the first 12 patients have been enrolled and potentially followed for 6 months, this mCRPC cohort will no longer enroll any patients.
- For patients with TNBC (cohort 5),
  - BRCAwt patients will be assigned to Durvalumab+O only:
    - Stage 1: If 0 to 1 of the first 16 evaluable patients have a clinical response, then no further patients will be accrued.
    - Stage 2: If 2 or more of the first 16 patients have a response, then accrual would continue until a total of 25 evaluable patients have been enrolled.
  - gBRCAm patients will be assigned to Durvalumab+O only:
    - A single cohort of 10 gBRCAm patients will be enrolled, if 0 clinical responses are identified in the first 5 patients with gBRCAm then no further gBRCAm patients will be enrolled.
- For patients with CRC (cohort 6),
  A single cohort of 13 patients will be enrolled to C+Durvalumab only. As a pilot biomarker study, CRC patients will start cediranib alone for 14 days (cycle 1 days – (minus) 14 to -1) and will begin Durvalumab in combinaition with cediranib on cycle 1 day 1.

3.2 DRUG ADMINISTRATION

3.2.1 Durvalumab (All Arms)

See section **15.1.3** for preparation instructions.

Durvalumab will be administered once every 28 days for a fixed dose schedule or once every 14 days for other dose levels except a fixed dose schedule in each 28-day cycle until disease progression, patient withdrawal or toxicities. Durvalumab is administered intravenously, through

an intravenous line containing a sterile, low-protein binding 0.2 or 0.22-micrometer in-line filter, at the assigned dose over approximately 60 +/- 5 minutes. An infusion time of less than 55 minutes is a deviation. If the infusion is interrupted, the reason for interruption will be documented in the clinical record.

Administration of Durvalumab may be given one day (every 2 weeks schedule) or 4 days (every 4 weeks schedule) earlier or delayed up to 8 days due to holidays, inclement weather, conflicts, or similar reasons. The timing of subsequent administrations is then adjusted to maintain a 28 days-interval for dose level 3 or a 14 days-interval for other dose levels except dose level 3.

Since the compatibility of Durvalumab with other IV medications and solutions, other than normal saline (0.9% [weight/volume] sodium chloride for injection), is not known, the Durvalumab solution should not be infused through an IV line in which other solutions or medications are being administered.

Patients will be monitored during and after the infusion with assessment of vital signs at the times specified in the Study Calendar (see Section **3.5**).

In the event of a ≤ grade 2 infusion-related reaction, the infusion rate of study drug may be decreased by 50% or interrupted until resolution of the event (up to 4 hours) and re-initiated at 50% of the initial rate until completion of the infusion. For subjects with a ≤ grade 2 infusion related reaction, subsequent infusions may be administered at 50% of the initial rate. Acetaminophen and/or an antihistamine (e.g., diphenhydramine) or equivalent medications may be administered at the discretion of the investigator (see **Table 16**).

3.2.2 Olaparib (Durvalumab+O and Durvalumab+O+C Arms only)

Olaparib at the appropriate dose level will be given orally continuously twice daily. Olaparib tablet maintenance 300 mg twice daily will be administered, even if the patient discontinues Durvalumab due to Durvalumab-related toxicities; olaparib will be given until disease progression, patient withdrawal or toxicities. The correct number of tablets comprising the appropriate dose should be taken at the same times each day with approximately 240 mL of water. Patients may take olaparib tablet with a snack or light meals and should avoid eating grapefruit or Seville oranges (including juice of these fruits), while on study, due to cytochrome P450 interactions. The olaparib tablets should be swallowed whole and not chewed, crushed, dissolved, or divided.

Olaparib will be dispensed at the start of each cycle. Patients will be provided with a pill diary (**Appendix C**), instructed in its use, and asked to bring it with them to each appointment. This will be used as a memory aide for subjects. A clinical research team maintains the primary source record in CRIS.

If vomiting occurs shortly after olaparib is swallowed, the dose should only be replaced if all of the intact product can be seen and counted. Should any patient enrolled on the study miss a scheduled dose, the patient will be allowed to take the scheduled dose up to a maximum of 2 hours before/after that scheduled dose time. If greater than 2 hours before/after the scheduled

dose time, the missed dose should not be taken, and the patient should take their allotted dose at the next scheduled time.

3.2.3 Cediranib (Durvalumab+C, C+Durvalumab and Durvalumab+O+C Arms only)

Patients will take cediranib once daily (+/- 2 hours) for cediranib daily schedule of Durvalumab+C arm or will take cediranib every 24 hours (5 days on/2 days off) for cediranib intermittent schedule of Durvalumab+C, C+Durvalumab and Durvalumab+O+C arms. Patients will continue cediranib maintenance with an initially assigned dose, even if the patient discontinues Durvalumab due to Durvalumab-related toxicities; cediranib will be given until disease progression, patient withdrawal or toxicities. Cediranib at the appropriate dose level will be given orally each morning on an empty stomach, either 1 hour before or 2 hours after breakfast. Patients should not "make up" a missed dose or a dose that was vomited. Patients should take cediranib with a glass of water. Cediranib will be dispensed at the start of each cycle. Patients will be provided with a pill diary for each drug (**Appendix C**), instructed in its use, and asked to bring it with them to each appointment. This will be used as a memory aide for subjects. A clinical research team maintains the primary source record in CRIS.

For patients who later become unable to swallow whole tablets, and who are determined to be benefitting from therapy, tablets may be administered as a dispersion in water; cediranib tablets may be dispersed by dropping the tablet in 50 mL (approximately half a glass) of non-carbonated drinking water, and stirring for 10 minutes, and immediately swallowing this suspension. Any residues left in the glass are to be mixed with another half a glass of water as described above, and then swallowed immediately.

Frequent blood pressure monitoring is important in patients receiving cediranib. Patients during the combinations (Durvalumab+cediranib or Durvalumab+olaparib+cediranib) or cediranib maintenance will be asked to record twice-daily blood pressure readings and bring a BP diary at subsequent visits (see **Appendix D**). If two successive systolic readings are > 140 mmHg OR two successive diastolic readings are > 90 mmHg OR any combination of elevated systolic and diastolic blood pressure are observed, patients will be instructed to contact their physician as soon as possible.

Patients should seek medical advice if their BP exceeds 180 mmHg (systolic) or 105 mmHg (diastolic) at any time and should also be encouraged to contact their physician if they are concerned about any symptoms that may be associated with high blood pressure (e.g., headache). Section **3.4** includes specific guidelines on the management and, if appropriate, dose modifications for treatment-emergent hypertension. Clinical trials of cediranib demonstrate that increases in blood pressure may occur following dosing with cediranib for a number of weeks and that these increases may occur relatively quickly when starting the drug.

3.3 Self Administered IND agents

The cediranib and olaparib used in this study are self-administered investigational agents. Such agents are dispensed from the pharmacy to a participant or to a Patient Care Unit for self-medication and a record of the dispensed investigational agent is generated and kept by the dispensing pharmacy.

As indicated above, patients will be asked to keep a medication diary and bring it with them on each study visit. Patients will also bring any remaining pills to each study visit. The Research Nurse reviews and validates the completeness and accuracy of the participant's diary with the participant.

If a participant goes off study while at home, the Research Nurse will ensure and document the return of the unused oral investigational agents from the participant. Unused investigational agent will be destroyed per dispensing pharmacy procedure.

3.4 DOSING DELAYS/DOSE MODIFICATIONS AND MANAGEMENT OF TOXICITIES ASSOCIATED WITH DURVALUMAB, OLAPARIB, CEDIRANIB

Dose delays and modifications will be made using the following recommendations.

BRCA mutant patients who are benefiting from treatment on the study drugs may also benefit from risk-reducing prophylactic surgery such as risk-reducing bilateral salpingo-oophorectomy or mastectomy. In these cases study drugs may be held for up to 4 weeks (up to 6 weeks for total mastectomy) at the discretion of the PI.

Management of specific toxicities including pneumonitis, diarrhea/enterocolitis, hepatitis (elevated LFTs), rash, endocrinopathy, immune mediated neurotoxicity, immune mediated peripheral neuromotor syndromes, infusion-related reactions, hypertension, proteinuria, decreased in LVEF, fever and neutropenia, thyroid toxicities, and reversible posterior leukoencephalopathy syndrome (RPLS) will be as further outlined in specific subsections to follow.

3.4.1 General Management of Toxicities Related to Durvalumab, Olaparib, Cediranib

- In the case of toxicity, appropriate medical treatment should be used (including anti-emetics, anti-diarrheals, etc.).
- Once a patient has a dose reduction for toxicity, the dose will not be increased.
- Participants continuing to experience toxicity at the off study visit will be contacted for additional assessments until the toxicity has resolved or is deemed irreversible. Patients must remain on the study to have additional assessment.
- For AEs that are unrelated to the study drugs, study drug may be held for up to 14 days at the discretion of the PI.

The dose levels and the general approach to dose modification of Durvalumab and olaparib or cediranib combination therapy are shown below. AEs should be treated with the appropriate maximum intervention, and dose reductions should be clearly documented in the note.

3.4.2 General Recommendation for Dose Modification

Dose Level	Olaparib tablets
1	300 mg twice daily
-1	250 mg twice daily
-2	200 mg twice daily

Dose level	Cediranib tablets
1	20 mg (5 days on/2 days off)
-1	15 mg (5 days on/2 days off)
-2	10 mg (5 days on/2 days off)

Dose Level	Durvalumab IV
1	A fixed dose of 1500 mg every 4 weeks
-1	A fixed dose of 500 mg every 4 weeks

Patients experiencing ongoing clinical benefit who experience a related AE where continuation of one of the drugs is considered, **in the judgment of the treating investigator AND the PI**, to be potentially life-threatening or with the potential for long-term harm to the patient, may be allowed to continue on the unrelated drug **after discussion with the PI.**

The associate investigator and/or PI will determine whether one or both drugs is responsible for an observed toxicity and will manage that toxicity as described below.

3.4.3 General Recommendations for Management of Hematologic Adverse Events

Table 11. Management of Hematologic Adverse Events

Observation	Action
Absolute neutrophil count (ANC) ≥ 1000/mcL<br>**AND**	No interruption.

Table 11. Management of Hematologic Adverse Events

Observation	Action
Platelets ≥ 75,000/mcL<br>**AND**<br>Hemoglobin ≥ 8 mg/dL	
ANC < 1000/mcL<br>**OR**<br>Platelets < 75,000/mcL<br>**OR**<br>Hemoglobin < 8 mg/dL	*On first occurrence*, hold the drug(s) causing the toxicity for up to 14 days until ANC ≥ 1000/mcL, platelets ≥ 75,000/mcL, and hemoglobin ≥ 9 mg/dL.<br>The associate investigator and/or PI will determine whether one or both drugs are responsible for an observed toxicity and will initiate appropriate medical therapy and no change in dose upon re-initiation. Treatment with olaparib may be restarted at one dose level (DL) lower on first occurrence after discussion with the PI if the patient's bone marrow may not tolerate the higher dose of olaparib due to prior radiation therapy or multiple chemotherapies, considering a transfusion has already been required to achieve recovery of anemia.<br><br>*On second occurrence*, hold the drug(s) causing the toxicity for up to 14 days until ANC ≥ 1000/mcL, platelets ≥ 75,000/mcL, and hemoglobin ≥ 9 mg/dL. Initiate appropriate medical therapy. Treatment with olaparib or cediranib may be restarted at one dose level (DL) lower.<br><br>Patients whose counts have not recovered to ANC ≥ 1000/mcL, platelets ≥ 75,000/mcL, and hemoglobin ≥ 9 mg/dL after 14 days should be removed from study regimen.
Grade 4 hematologic AE	*On first occurrence,* hold the drug(s) causing the toxicity for up to 14 days until ANC ≥ 1000/mcL, platelets ≥ 75,000/mcL, and hemoglobin ≥ 9 mg/dL. Initiate appropriate medical therapy. Treatment with olaparib or cediranib may be restarted at one DL lower.<br><br>*On second occurrence,* discontinue the drug(s) causing the toxicity. Durvalumab may be continued at PI's discretion if counts have recovered to ANC ≥ 1000/mcL,

**Table 11**. Management of Hematologic Adverse Events	
**Observation**	**Action**
	platelets ≥ 75,000/mcL, and hemoglobin ≥ 9 mg/dL within 14 days, Follow patient until resolution/stabilization of toxicity.

3.4.3.1 Hematologic toxicities

- Treatment may be delayed for a maximum of 14 days after holding the treatment for toxicities that develop and do not resolve as defined above (exemptions: lymphopenia, or leukopenia in the absence of grade 3 or higher neutropenia).
- Weekly blood counts will be obtained during the first cycle, and then at the start of each cycle. If any weekly evaluation demonstrates grade ≥ 3 neutropenia or grade ≥ 2 thrombocytopenia, a repeat hematology assessment will be obtained 2-4 days later.
- Management of prolonged hematological toxicities while on study treatment
  - If a patient develops prolonged hematological toxicity such as:
    - ≥ 14 days of interruption/delay in study treatment due to grade 3 or worse anemia and/or development of blood transfusion dependence
    - ≥ 14 days interruption/delay in study treatment due to grade 3 or worse neutropenia (ANC < 1000/mcL)
    - ≥ 14 days interruption/delay in study treatment due to grade 3 or worse thrombocytopenia (Platelets < 50,000/mcL)
- Weekly differential blood counts including reticulocytes (calculate reticulocyte index (RI), RI = reticulocyte count x hematocrit (Hct)/normal Hct; a value of 45 is usually used for normal Hct) and peripheral blood smear should be performed. If any blood parameters remain clinically abnormal after 4 weeks of dose interruption, the patient should be referred to hematologist for further investigations. Bone marrow analysis and/or blood cytogenetic analysis should be considered at this stage according to standard hematological practice.
- Development of a confirmed myelodysplastic syndrome or other clonal blood disorder should be reported as an SAE to the sponsor and manufacturer (see section 9) and full reports must be provided by the investigator to AstraZeneca Patient Safety. Study treatment should be discontinued if diagnosis of myelodysplastic syndrome is confirmed.

3.4.3.2 Neutropenia

- Growth factors to prevent neutropenia will not be administered prophylactically, but can be used during a drug hold to assist the recovery.
- Filgrastim/PEG filgrastim will be used only if the patient has neutropenia with sepsis for the purpose of facilitating recovery of the neutropenic sepsis.

3.4.3.3 Thrombocytopenia

- Thrombocytopenia will be treated conservatively. In the absence of bleeding, or a necessary invasive procedure, platelet transfusions should be given for a platelet count ≤ 10,000/mcL.

- If invasive procedure(s) is (are) planned, or the patient develops bleeding, platelet transfusions should be administered in accordance with the standard of practice, usually maintaining a platelet count above 50,000/mcL.

3.4.3.4 Anemia

- Symptomatic anemia should be treated with red blood cell transfusion and is recommended if the hemoglobin falls below 8 g/dL or the patient is symptomatic.
- The initiation of erythropoietic therapy for the management of chemotherapy-induced anemia follows the American Society of Hematology/ASCO clinical practice guidelines (http://www.asco.org).

3.4.4 General Recommendations for Management of Non-Hematologic Adverse Events

- The management of general AEs not otherwise specified in the following sections should be as per **Table 12**. Management of specific toxicities, including hypertension, diarrhea, proteinuria, decrease in LVEF, thyroid toxicities, and RPLS should be as further outlined in the below specific subsections and not per **Table 12**.
- Dose modifications for *nausea, vomiting, and diarrhea* will be made *only if* they are refractory to treatment. The time a given drug is held should not exceed 14 days.
- Asymptomatic electrolyte abnormalities with optimal repletion will not require dose reduction if resolution to grade 1 or less is documented within 4 days after onset of the event.
- Treatment with olaparib may be restarted at 150 mg tablets twice daily after discussion with the PI to avoid potential drug interactions if a permanent use of anti-arrhythmic drug e.g. amiodarone, is clinically indicated.

Table 12. General Management of Adverse Events (Non-Hematologic)

**Observation**	**Action**
Grade 1 or 2 AEs resolves promptly (within 48 hours) with supportive care	Maintain dose level (DL)
Any ≥ grade 3 non-hematologic*	*During DLT evaluation period:*<br><br>Patient will be removed from study regimen.<br><br>*At any other time*<br><br>Hold study drug(s)** for up to 14 days until toxicity resolves to ≤ grade 1. Treatment with olaparib or cediranib may be restarted at one DL lower, as per the dose reduction levels in **Table 6** and **Table 7**.

Any grade 2 non-hematologic AE or grade 3 fatigue or hypertension related to study drug(s) lasting > 7 days despite maximal intervention.	Hold study drug(s)** for up to 14 days until toxicity resolves to ≤ grade 1.<br><br>Treatment with olaparib or cediranib may be restarted at one DL lower, as per the dose reduction levels in **Table 6** and **Table 7**.<br><br>Patients whose toxicity has not resolved after 14 days will be removed from study regimen.
Grade 3 or 4 non-hematologic AE related to Durvalumab*, cediranib or olaparib combination therapy that does not resolve to grade 1 or less within 14 days despite maximum intervention after treating patient at the lowest reduced DL***	Remove patient from study regimen.

* Excluding 1) Grade 3 hypertension controlled with anti-hypertensive therapy, or grade 3 asymptomatic electrolytes imbalance with optimal and continuing repletion that downgrades to grade 1 or better within 3 days after onset of the event; 2) Grade 3 asymptomatic increase in gamma glutamyl transferase (GGT) OR grade 3 asymptomatic increase in amylase or lipase that downgrades to Grade 1 or better within 7 days after onset of the event; 3) Grade 3 endocrinopathy that is managed with or without systemic corticosteroid therapy and/or hormone replacement therapy and the patient is asymptomatic.

** For patients on combination therapy, if the observed AE is specifically attributed to only 1 of the drugs, that drug may be held while the patient continues to receive the drug not associated with the observed AE. The time a given drug is held should not exceed 14 days.

*** Excluding hypertension, see below.

3.4.4.1 Cediranib

3.4.4.1.1 Dose Modification and Management of Hypertension for Cediranib

Only doses of cediranib will be modified for hypertension; olaparib or Durvalumab doses will not be altered unless other toxicities are experienced. Patients receiving cediranib should be provided with blood pressure monitors for home use and will check and record their blood pressures at least twice daily while on study treatment (see **Appendix D**). Please refer to **Appendix F** for additional information on antihypertensive therapeutic options and their interactions with other medications.

**Table 13: Hypertension Monitoring and Management**
• See table for suggested antihypertensive medications by class (**Appendix F**).<br>• Abbreviations: Angiotensin Converting Enzyme (ACE) Inhibitors, Angiotensin II Receptor Blockers (ARB),

selective beta blockers (BB), Calcium channel blockers (CCB)

- If patients require a delay of >2 weeks for management of hypertension, discontinuation of cediranib may be considered after discussion with the PI.
- Patients may have up to 4 drugs for management of hypertension prior to any dose reduction in cediranib
- Hypertension should be graded using the NCI CTCAE v4.0. Please note: patients may have baseline hypertension meeting CTCAE grading criteria on study entry. Should patients require increase in dosing of BP medication or increased number of medications, they should then be noted to have hypertension related to study drug, with grading as per CTCAE v4.0 criteria. Baseline grade of hypertension should also be recorded in the patient's record.
- **Note**: Stopping or reducing the dose of cediranib is expected to cause a decrease in BP. <u>The treating physician should monitor the patient for hypotension and adjust the number and dose of antihypertensive medications accordingly.</u>

Event	Definition	Antihypertensive Therapy	Blood Pressure Monitoring	Cediranib Dose Modification
**Grade 1**	Asymptomatic transient (<24 hrs) increase by >20 mmHg diastolic or to >140/90 mmHg if previously WNL	Consider early initiation of BP medication for BP > 140/90 mmHg that is confirmed on a second reading. Cediranib can cause rapid escalation in BP, and early initiation of BP management can reduce likelihood of HTN-related complications.	Continue standard BP monitoring per treating MD and confirm resolution of BP to <140/90 mmHg within 36 hours.	None
**Grade 2**	Recurrent or persistent (>24 hrs) or symptomatic increase by >20 mmHg (diastolic) or to > 140/90 mmHg if previously WNL<br><br>Monotherapy may be indicated	Initiate BP medication for first line treatment:<br>• *Suggestion:* ACE inhibitor<br><br>Escalate dose of medication until BP is controlled or at a maximum dose<br><br>If BP is not controlled to < 140/90 mmHg with one "maximized" drug regimen, then add a second agent:	Increase frequency of monitoring (daily) until stabilized to BP <140/90 mmHg	Do not hold cediranib unless otherwise clinically necessary

		**Study drug does not need to be held unless otherwise clinically necessary**<br><br>*Consider cardiology or renal consult*		
**Grade 3**	Requiring more than one drug or more intensive therapy than previously.	Maximize 2 drug regimen<br>• *Suggestions:*<br>ACE inhibitor + BB or<br>ACE inhibitor + CCB<br><br>Escalate doses of existing medication until BP is controlled or at a maximum dose.<br><br>If BP is not controlled to < 140/90 mmHg with two drug regimen, then add a third agent.<br><br>**Study Drug will not be held during trial of two drug combinations.**<br><br>**Additional antihypertensive drugs, up to a total of 4, may be maximized for blood pressure control.**<br><br>*Consider consult with a blood pressure management specialist if greater than 3 drugs are required for BP control.*	Increased frequency of monitoring (BID) until stabilized to BP <140/90 mmHg	Do not hold cediranib or other study drug unless BP is not decreased to less than 150/100 mmHg 48 hours after multi-drug therapy is instituted or if clinical symptoms worsen (e.g. headache).<br><br>If BP is not controlled to less than 150/100 mmHg with maximal therapy or if clinical symptoms worsen, then hold drug (up to 14 days) until maximum effect of the antihypertensive agents is achieved.<br><br>If BP is reduced to Grade 1 within 14 days, cediranib may be resumed at prior dose.
**Grade 4**	If threatening consequences	Initiate treatment	Intensive BP monitoring	**Hold cediranib.**

	**OR**<br>SBP ≥ 180mmHg<br>**OR**<br>DBP ≥ 110mmHg	Hospitalize patient for ICU management, IV therapy as necessary<br><br>14 days are allowed to maximize the full effect of antihypertensive agents.	(hospitaliza-tion if necessary)	If BP is reduced to Grade 1 within 14 days, cediranib may be resumed at a reduced dose after discussion with the PI

Notes:

- While patients are receiving treatment with cediranib, the early initiation of antihypertensive treatment for grade 1 or 2 hypertension to minimize more severe or persistent hypertension is not considered a grade 3 adverse event.
- Decisions to hold or decrease the cediranib dose during treatment must be based on BP readings taken in the clinic by a medical professional.

3.4.4.1.2 Dose Modification and Management of Decreased Left Ventricular Ejection Fraction

Patients who have any of the following should undergo an echocardiogram or MUGA at baseline and every four cycles while on study:

- Prior treatment with anthracyclines
- Prior treatment with trastuzumab
- A New York Heart Association classification of II controlled with treatment
- Prior central thoracic radiation therapy (RT), including RT to the heart
- History of myocardial infarction within the prior 12 months.

Only doses of cediranib will be modified for LVEF; olaparib or Durvalumab doses will not be altered unless other toxicities are experienced. The decision to continue or hold cediranib is based on the LVEF as it relates to the institution's lower limit of normal (LLN) and change in ejection fraction from screening (LVEF as measured at registration) according to the following table:

Table 14. Management and Monitoring of Decreased LVEF

Relationship of LVEF to Institution's LLN	LVEF Decrease < 10%	LVEF Decrease 10-15%	LVEF Decrease > 16%
Normal	Continue	Continue	Continue and repeat MUGA/ECHO

			within 1-2 cycles
1-5% below LLN	Continue and repeat MUGA/ECHO within 1-2 cycles	Continue and repeat MUGA/ECHO within 1-2 cycles	HOLD and repeat MUGA/ECHO within 1-2 cycles
> 6% below LLN	Continue and repeat MUGA/ECHO within 1-2 cycles	HOLD and repeat MUGA/ECHO within 1-2 cycles	HOLD and repeat MUGA/ECHO within 1-2 cycles

3.4.4.1.3 Dose Modification and Management of Proteinuria for Cediranib

Patients with greater than 1+ proteinuria at entry are ineligible. Proteinuria has not been reported as a significant adverse event associated with use of this agent, although it has been observed in trials with similar agents. Thus, increases in proteinuria may occur during treatment and should be managed as follows:

Table 15. Management of Proteinuria

Proteinuria Value if following by U/A	Value Monitoring	Dose modification
Greater than 2+ on urine dipstick or U/A<br>**AND**<br>Creatinine ≤1.5x ULN	Perform UPC.	Continue study drugs at planned dose.
Greater than 2+ on urine dipstick or U/A **AND**<br>Creatinine >1.5x ULN	Perform UPC.	**HOLD cediranib** until results of UPC are known, and see below
**Based on results of the UPC†:**		
UPC ≤ 1.0	Continue monitoring prior to each cycle as per previous.	Continue study drugs at planned dose.
UPC > 1.0 and ≤ 3.5 **AND** Creatinine ≤1.5x ULN	Perform UPC prior to each cycle.	Continue study drugs at planned dose.

UPC > 3.5 **OR** Creatinine >1.5x ULN	Perform UPC prior to each cycle.	**Hold cediranib** for up to 7 days and repeat UPC and Creatinine assessment. If UPC resolves to <3.5 and Creatinine to ≤1.5x ULN, resume cediranib with reduction in cediranib by one dose level. Consider consultation with nephrologist.
†If UPC is <1.0 and creatinine >1.5x ULN, AE management should be followed as per **Table 12**.		

3.4.4.1.4 Dose Modifications for Reversible Posterior Leukoencephalopathy Syndrome (RPLS) with cediranib

Cediranib should be held in patients with symptoms/signs suggestive of RPLS, pending work-up and management, including control of blood pressure. Cediranib should be discontinued upon diagnosis of RPLS.

3.4.4.2 Durvalumab

3.4.4.2.1 Infusion-related reaction with Durvalumab

- Patients will be monitored during and after the infusion with assessment of vital signs at the times specified in the study protocol.
- In the event of grade 1 or 2 infusion-related reaction, the infusion rate of study drug may be decreased by 50% or interrupted until resolution of the event (up to 4 hours) and re-initiated at 50% of the initial rate until completion of the infusion. For patients with grade 1 or 2 infusion-related reaction, subsequent infusions may be administered at 50% of the initial rate. Acetaminophen and/or an antihistamine (e.g., diphenhydramine) or equivalent medications may be administered at the discretion of the PI.
- If the infusion-related reaction is grade 3 or higher OR recurrent grade 2 or higher in severity, Durvalumab will be discontinued and olaparib or cediranib will continue the maintenance dose.

<table>
<tr><td colspan="2">Table 16. Management of Infusion-Related Reactions</td></tr>
<tr><th>Dose Modifications</th><th>Toxicity Management</th></tr>
<tr><td>Any Grade</td><td>• Management per institutional standard at the discretion of investigator
• Monitor patients for signs and symptoms of infusion-related reactions (e.g., fever and/or shaking chills, flushing and/or itching, alterations in heart rate and blood pressure, dyspnea or chest discomfort, skin rashes etc.) and anaphylaxis (e.g., generalized urticaria, angioedema, wheezing, hypotension, tachycardia, etc.)</td></tr>
<tr><td>Grade 1
The infusion rate of Study regimen may be decreased by 50% or temporarily interrupted until resolution of the event</td><td rowspan="2">For Grade 1 or Grade 2:
• Acetaminophen and/or antihistamines may be administered at the discretion of the investigator
• Consider premedication prior to subsequent doses</td></tr>
<tr><td>Grade 2
The infusion rate of Study regimen may be decreased 50% or temporarily interrupted until resolution of the event (up to 4 hours). Subsequent infusions may be given at 50% of the initial infusion rate</td></tr>
<tr><td>Grade 3 or 4
Permanently discontinue Study regimen.</td><td>For Grade 3 or 4:
Manage severe infusion-related reactions (e.g., IM epinephrine, followed by IV diphenhydramine and ranitidine, and IV glucocorticoid)</td></tr>
</table>

3.4.4.2.2 Immune-mediated adverse events

- Based on the mechanism of action of Durvalumab leading to T-cell activation and proliferation, there is the possibility of observing immune related Adverse Events (irAEs) during the conduct of this study. Potential irAEs include immune mediated enterocolitis, dermatitis, hepatitis, and endocrinopathies. Subjects should be monitored for signs and symptoms of irAEs. In the absence of an alternate etiology (e.g., infection or progression) signs or symptoms of enterocolitis, dermatitis, hepatitis, and endocrinopathy should be considered to be immune-related.
- Dose modification recommendations and toxicity management guidelines for Durvalumab, immune-mediated reactions are detailed in **Table 17 - Table 24**.

Table 17. Immune-related Adverse Events (Overall Management)

Dose Modifications		Toxicity Management
Drug administration modifications of Study regimen will be made to manage potential<br>irAEs per NCI CTCAE v4.03.		It is recommended that management of irAEs follow the guidelines presented in this table<br><br>– Patients should be thoroughly evaluated to rule out any alternative etiology (e.g., disease progression, concomitant medications, infections, etc.).<br>– In the absence of a clear alternative etiology, all events should be considered potentially immune related.<br>– Symptomatic and topical therapy should be considered for low-grade (grade 1 or 2, unless otherwise specified) events.<br>– For persistent (greater than 3 to 5 days) low-grade (Grade 2) or severe (Grade ≥3) events promptly start prednisone PO 1-2mg/kg/day or IV equivalent.<br>– If symptoms recur or worsen during corticosteroid tapering (≥4 weeks of taper), increase the corticosteroid dose (prednisone dose [e.g. up to 2-4mg/kg/day or IV equivalent]) until stabilization or improvement of symptoms, then resume corticosteroid tapering at a slower rate.<br>– More potent immunosuppressives – (refer to individual sections of the immune related adverse event for specific type of immunosuppressive) should be considered for events not responding to systemic steroids.<br>– Discontinuation of study drug is not mandated for Grade 3 / Grade 4 inflammatory reactions attributed to local tumour response (e.g., inflammatory reaction at sites of metastatic disease, lymph nodes etc.). Continuation of study drug in this situation should be based upon a benefit/risk analysis for that patient
**Grade 1**	No dose modification	
**Grade 2**	Hold Study regimen dose until grade 2 resolution to ≤ grade 1.<br>If toxicity worsens then treat as grade 3 or grade 4.<br>If toxicity improves to baseline then treat at next scheduled treatment date.	
**Grade 3**	Depending on the individual toxicity, may permanently discontinue Study regimen. Please refer to guidelines below.	
**Grade 4**	Permanently discontinue Study regimen.<br><br>Note: For Grade 3 and above asymptomatic amylase or lipase levels hold study drug/regimen and if complete work up shows no evidence of pancreatitis, may continue or resume study drug/regimen	

**Table 17. Immune-related Adverse Events (Overall Management)**	
**Dose Modifications**	**Toxicity Management**
	Note: In addition to the criteria for permanent discontinuation of study drug/regimen based on CTC grade/severity (table below), permanently discontinue study drug/study regimen for the following conditions:<br>– Inability to reduce corticorsteroid to a dose of ≤10 mg of prednisone per day (or equivalent) within 12 weeks after last dose of study drug/regimen<br>– Recurrence of a previously experienced Grade 3 treatment-related AE following resumption of dosing.

	**Table 18**. Pneumonitis/Interstitial Lung Disease (ILD)	
	**Dose Modifications**	**Toxicity Management**
	Any Grade	- Monitor patients for signs and symptoms of pneumonitis or ILD (new onset or worsening shortness of breath or cough). Patients should be evaluated with imaging and/or pulmonary function tests including other diagnostic procedures as described below.<br>- Initial work-up may include clinical evaluation, monitoring of oxygenation via pulse oximetry (resting and exertion), laboratory work-up and high-resolution CT scan.
**Grade 1**	No dose modification required. However, consider holding Study regimen dosing as clinically appropriate and during diagnostic work-up for other etiologies	For Grade 1 (Radiographic Changes Only)<br>- Monitor and closely follow up in 2-4 days for clinical symptoms, pulse oximetry (resting and exertion) and laboratory work-up and then as clinically indicated<br>- Consider pulmonary and infectious disease consult.

**Table 18**. Pneumonitis/Interstitial Lung Disease (ILD)		
	**Dose Modifications**	**Toxicity Management**
**Grade 2**	Hold Study regimen dose until grade 2 resolution to grade 1 or better<br>• If worsens then treat as grade 3 or grade 4<br>• If improves to baseline then treat at next scheduled treatment date	For Grade 2 (Mild to Moderate New Symptoms)<br>- Monitor symptoms daily and consider hospitalization<br>- Discuss with the PI and consider systemic steroids (e.g., prednisone 1-2mg/kg/day or IV equivalent<br>- Reimaging as clinically indicated<br>- If no improvement within 3-5 days, additional workup should be considered and prompt treatment with IV methylprednisolone 2-4mg/kg/day started<br>- If still no improvement within 3-5 days despite IV methylprednisone at 2-4m4mg/kg/day, promptly start immunosuppressive therapy (infliximab at 5mg/kg every 2 weeks). Caution: Important to rule out sepsis and refer to infliximab label for general guidance before using infliximab<br>- Once improving, gradually taper steroids over ≥4 weeks and consider prophylactic antibiotics, antifungal or anti PCP treatment.<br>- Consider Pulmonary and Infectious Disease consult
**Grade 3 or 4**	Permanently discontinue Study regimen	For Grade 3 or 4 (Severe or New Symptoms, New/Worsening Hypoxia, Life threatening)<br>- Promptly initiate empiric IV methylprednisolone 1 to 4 mg/kg/day or equivalent<br>- Obtain pulmonary and infectious disease consult<br>- Hospitalize the patient<br>- Supportive Care (oxygen, etc.)<br>- If no improvement within 3-5 days, additional workup should be considered and prompt treatment with additional immunosuppressive therapy infliximab at 5mg/kg every 2 weeks dose) started. Caution: rule out sepsis and refer to infliximab label for general guidance before using infliximab.<br>- Once improving, gradually taper steroids over ≥ 4 weeks and consider prophylactic antibiotics, antifungal or anti PCP treatment

**Table 19**. Diarrhea and enterocolitis		
Dose modifications		Toxicity Management
Any Grade		- Monitor for symptoms that may be related to diarrhea/enterocolitis (abdominal pain, cramping, or changes in bowel habits) and daily document bowel movements (Appendix E)<br>- Patients should be thoroughly evaluated to rule out any alternative etiology (e.g., disease progression, infections, etc.)<br>- Steroids should be considered if an alternative etiology is not determined, even for low grade events, in order to prevent potential progression to higher grade event<br>- Use analgesics carefully; they can mask symptoms of perforation and peritonitis
**Grade 1**	No dose modification	For Grade 1:<br>- Close monitoring for worsening symptom<br>- Consider symptomatic treatment including hydration, electrolyte replacement, dietary changes (e.g., ADA colitis diet), and loperamide<br>- Patients can take loperamide (per standard practice) and continue to take loperamide until patients are free from diarrhea for at least 12 hours. The dose of loperamide should not exceed 16mg in a 24-hour period. Patients will be followed by phone every day until they are free from diarrhea for at least 12 hours.<br>- If diarrhea persists despite 24 hours of loperamide treatment, hold the drug(s) causing the toxicity for a maximum of 7 days, continue loperamide, and maintain hydration. The drug(s) causing the toxicity may be restarted at the same dose once patients have been free from diarrhea for 12 hours

Table 19. Diarrhea and enterocolitis

Dose modifications		Toxicity Management
**Grade 2**	Hold Study regimen dose until grade 2 resolution to ≤ grade 1<br>• If worsens then treat as grade 3 or grade 4<br>• If improves to baseline then treat at next scheduled treatment date	For Grade 2:<br>- NPO<br>- Consider symptomatic treatment including hydration, electrolyte replacement, and loperamide and/or budesonide<br>- Promptly start prednisone 1 to 2 mg/kg/day or IV equivalent<br>- If event is not responsive within 3-5 days or worsens despite prednisone at 1-2 mg/kg/day or IV equivalent, GI consult should be obtained for consideration of further workup such as imaging and/or colonoscopy to confirm colitis and rule out perforation, and prompt treatment with IV methylprednisolone 2-4mg/kg/day started.<br>- If still no improvement within 3-5 days despite 2-4mg/kg IV methylprednisolone, promptly start immunosuppressives (infliximab at 5mg/kg once every 2 weeks). Caution: Important to rule out bowel perforation and refer to infliximab label for general guidance before using infliximab<br>- Once improving, gradually taper steroids over ≥4 weeks and consider prophylactic antibiotics, antifungal or anti PCP treatment<br>- Consider GI consult and imaging as appropriate.
**Grade 3 or 4**	Permanently discontinue Study regimen	For Grade 3 or 4:<br>- NPO<br>- Monitor stool frequency and volume and maintain hydration<br>- Promptly initiate empiric IV methylprednisolone 1 to 4 mg/kg/day or equivalent<br>- Urgent GI consult and imaging and/or colonoscopy as appropriate<br>- If still no improvement within 3-5 days of IV methylprednisolone 1 to 4mg/kg/day or equivalent, promptly start further immunosuppressives (infliximab at 5mg/kg once every 2 weeks).<br>- Caution: Ensure GI consult to rule out bowel perforation and refer to infliximab label for general guidance before using infliximab.<br>- Once improving, gradually taper steroids over ≥4 weeks and consider prophylactic antibiotics, antifungals and anti PCP treatment

**Table 20**. Hepatitis (Elevated LFTs)		
**Dose Modifications**		**Toxicity Management**
Any Grade		- Monitor and evaluate liver function test: AST, ALT, ALP and total bilirubin<br>- Evaluate for alternative etiologies (e.g., viral hepatitis, disease progression, concomitant medications)
**Grade 1**	No dose modification	Continue LFT monitoring per protocol<br>If it worsens, treat as grade 2
**Grade 2**	Hold Study regimen dose until grade 2 resolution to ≤ grade 1<br>• If worsens then treat as grade 3 or 4<br>• If improves to baseline then treat at next scheduled treatment date	For Grade 2:<br>- Regular and frequent checking of LFTs (e.g. every 1-2 days) until elevations of these are improving or resolved.<br>- If no resolution to ≤ Grade 1 in 1-2 days, discuss with study physician.<br>- If event is persistent (> 3-5 days) or worsens, promptly start prednisone 1-2mg/kg/day or IV equivalent.<br>- If still no improvement within 3-5 days despite 1-2mg/kg/day of prednisone or IV equivalent, consider additional workup and prompt treatment with IV methylprednisolone 2-4mg/kg/day started.<br>- If still no improvement within 3-5 days despite 2-4mg/kg/day of IV methylprednisolone, promptly start immunosuppressives (mycophenolate mofetil at 500mg every 12 hours). **Infliximab should NOT be used**.<br>- Once improving, gradually taper steroids over ≥4 weeks and consider prophylactic antibiotics, antifungals and anti PCP treatment.

Table 20. Hepatitis (Elevated LFTs)

Dose Modifications		Toxicity Management
**Grade 3**	For elevations in transaminases ≤ 8 × ULN, or elevations in bilirubin ≤ 5 × ULN<br>− Hold study regimen dose until resolution to ≤ grade 1 or baseline<br>− Resume study regimen administration at the next scheduled dose if elevations downgrade ≤ grade 1 or baseline within 14 days<br>Permanently discontinue study regimen if the elevations do not downgrade to ≤ grade 1 or baseline within 14 days.<br>For elevations in transaminases > 8 × ULN or elevations in bilirubin > 5 × ULN, discontinue study regimen.<br>Discontinue Study regimen for any case meeting Hy's law criteria*	For Grade 3 or 4:<br>- Promptly initiate empiric IV methylprednisolone at 1 to 4 mg/kg/day or equivalent<br>- If still no improvement within 3-5 days despite 1 to 4 mg/kg/day methylprednisolone IV or equivalent, promptly start treatment with immunosuppressive therapy (mycophenolate mofetil at 500mg every 12 hours) Discuss with study physician if mycophenolate is not available. Infliximab should NOT be used.<br>- Hepatology consult, abdominal workup, and imaging as appropriate.<br>- Once improving, gradually taper steroids over ≥4 weeks and consider prophylactic antibiotics, antifungals and anti PCP treatment.
**Grade 4**	Permanently discontinue Study regimen	

* Hy's Law

A prognostic indicator that pure drug-induced liver injury (DILI) leading to jaundice, without a hepatic transplant, has a case fatality rate of 10 – 50%. The principle is based on observations by Hy Zimmerman, a scholar of drug-induced liver injury.

Hy's Law cases include the following three components:

1. An implicated drug causes hepatocellular injury, generally shown by more frequent increase in ALT or AST ≥ 3x ULN than a (non-hepatotoxic) control or placebo.

2. Among subjects showing ↑'d transaminases, often with transaminases >> 3x ULN, some subjects also show increased serum total bilirubin >2x ULN, without initial evidence of cholestasis (serum alkaline phosphatase activity >2 x ULN).

3. No other reason explains the combination of ↑'d transaminases & total bilirubin; e.g., viral hepatitis A, B, or C; preexisting or acute liver disease; another drug capable of causing injury.

Defined by the U.S. Department of Health and Human Services, Food and Drug Administration, Center for Drug Evaluation and Research [42] Center for Biologics Evaluation and Research (CBER) in "Guidance for Industry Drug-Induced Liver Injury: Premarketing Clinical Evaluation, July 2009 Drug Safety"

Table 21. Rash (excluding Bullous skin formations)

**Dose Modifications**		**Toxicity Management**
Any Grade		Monitor for signs and symptoms of dermatitis (rash and pruritus)<br>**IF THERE IS ANY BULLOUS FORMATION, THE STUDY PHYSICIAN SHOULD BE CONTACTED AND STUDY DRUG DISCONTINUED**
**Grade 1**	No dose modification	For Grade 1:<br>- Consider symptomatic treatment including oral antipruritics (e.g., diphenhydramine or hydroxyzine) and topical therapy (e.g., urea cream)
**Grade 2**	For persistent (> 1- 2 weeks) grade 2 events, hold scheduled study regimen until resolution to ≤ grade 1 or baseline and resume administration at next scheduled dose	For Grade 2:<br>- Obtain dermatology consult<br>- Consider symptomatic treatment including oral antipruritics (e.g., diphenhydramine or hydroxyzine) and topical therapy (e.g., urea cream)<br>- Consider moderate-strength topical steroid<br>- If no improvement of rash/skin lesions occurs within 3-5 days or is worsening despite symptomatic treatment and/or use of moderate strength topical steroid, discuss with study physician and promptly start systemic steroids prednisone 1-2 mg/kg/day or IV equivalent<br>- Consider skin biopsy if persistent for >1-2 weeks or recurs

<table>
<tr><th colspan="3">Table 21. Rash (excluding Bullous skin formations)</th></tr>
<tr><th colspan="2">Dose Modifications</th><th>Toxicity Management</th></tr>
<tr><td>Grade 3</td><td>Hold study regimen until resolution to ≤ grade 1 or baseline
If temporarily holding the study regimen does not provide improvement of the grade 3 skin rash to ≤ grade 1 or baseline within 14 days, then permanently discontinue study regimen</td><td rowspan="2">For Grade 3 or 4:
- Consider hospitalization
- Consult dermatology
- Consider skin biopsy (preferably more than 1) as clinically feasible.
- Promptly initiate empiric IV methylprednisolone IV at 1 to 4 mg/kg/day or equivalent.
- Once improving, gradually taper steroids over ≥4 weeks and consider prophylactic antibiotics</td></tr>
<tr><td>Grade 4</td><td>Permanently discontinue study regimen</td></tr>
</table>

Table 22. Endocrinopathy (e.g., hyperthyroidism, hypothyroidism, hypopituitarism, adrenal insufficiency, etc.)		
**Dose Modifications**		**Toxicity Management**
Any Grade		- Monitor patients for signs and symptoms of endocrinopathies. Non-specific symptoms include headache, fatigue, behavior changes, changed mental status, vertigo, abdominal pain, unusual bowel habits, hypotension and weakness.<br>- Patients should be thoroughly evaluated to rule out any alternative etiology (e.g., disease progression **including brain metastases**, infections, etc.).<br>- Monitor and evaluate thyroid function tests: TSH, free T_3 and free T_4 and other relevant endocrine labs depending on suspected endocrinopathy.<br>- Consult endocrinologist.<br>- If a patient experiences an AE that is thought to be possibly of autoimmune nature (e.g., thyroiditis, pancreatitis, hypophysitis, diabetes insipidus), the investigator should send a blood sample for appropriate autoimmune antibody testing.
**Grade 1**	No dose modification	For Grade 1: (including those with asymptomatic TSH elevation)<br>– Monitor patient with appropriate endocrine function tests<br>- If TSH < 0.5 LLN, or TSH > 2X ULN or consistently out of range in 2 subsequent measurements, include FT4 at subsequent cycles as clinically indicated and consider Endocrinology consult.
**Grade 2**	Hold Study regimen dose until grade 2 resolution to ≤ grade 1<br>• If worsens then treat as grade 3 or 4<br>• If improves to baseline then treat at next scheduled treatment date	For Grade 2: (including those with symptomatic endocrinopathy)<br>- Isolated hypothyroidism may be treated with replacement therapy without treatment interruption and without corticosteroids<br>- Initiate hormone replacement as needed for management<br>- Evaluate endocrine function, and as clinically indicated consider pituitary scan<br>- For patients with abnormal endocrine work up, except for those with isolated hypothyroidism, consider short-term, corticosteroids (e.g., 1-2mg/kg/day methylprednisolone or IV equivalent) and prompt initiation of treatment with relevant hormone replacement (e.g. Levothyroxine, hydrocortisone, or sex hormones).<br>- For patients with normal endocrine work up (lab or MRI scans), repeat labs/MRI as clinically indicated.

**Table 22. Endocrinopathy (e.g., hyperthyroidism, hypothyroidism, hypopituitarism, adrenal insufficiency, etc.)**		
**Dose Modifications**		**Toxicity Management**
**Grade 3**	Hold Study regimen dose until endocrinopathy symptom(s) are controlled<br>Resume Study regimen administration if controlled at the next scheduled dose	For Grade 3 or 4:<br>- Isolated hypothyroidism may be treated with replacement therapy without treatment interruption and without corticosteroids<br>- Promptly initiate empiric IV corticosteroids (e.g., methylprednisolone IV or equivalent) at 1 to 2 mg/kg/day<br>- Administer hormone replacement therapy as necessary<br>- For adrenal crisis, severe dehydration, hypotension, or shock: immediately initiate intravenous corticosteroids with mineralocorticoid activity<br>- Consult endocrinologist<br>- Once improving, gradually taper immunosuppressive steroids over ≥4 weeks and consider prophylactic antibiotics, antifungals and anti PCP treatment.
**Grade 4**	Permanently discontinue Study regimen	

Table 23. Immune mediated Neurotoxicity (except Myasthenia Gravis and Guillain-Barré)

**Dose Modifications**	**Toxicity Management**
Any Grade	- Patients should be evaluated to rule out any alternative etiology (e.g., disease progression, infections, metabolic syndromes and medications, etc.)<br>- Monitor patient for general symptoms (headache, nausea, vertigo, behavior change, or weakness)<br>- Consider appropriate diagnostic testing (e.g., electromyogram and nerve conduction investigations)<br>- Consider neurological consult
**Grade 1**	No dose modifications
**Grade 2**<br>For acute motor neuropathies or neurotoxicity, hold Study regimen dose until resolution to ≤ grade 1<br>For sensory neuropathy/neuropathic pain, consider holding Study regimen dose until resolution to ≤ grade 1.<br>•If worsens then treat as grade 3 or grade 4<br>•If improves to baseline then treat at next scheduled treatment date	- Neurology Consult<br>- Sensory neuropathy/neuropathic pain may be managed by appropriate medications (e.g., gabapentin, duloxetine, etc.)<br>- Promptly start systemic steroids prednisone 1-2mg/kg/day or IV equivalent<br>- If no improvement within 3-5 days, despite 1-2mg/kg/day prednisone or IV equivalent consider additional workup and promptly treat with additional immunosuppressive therapy (e.g. IV IgG)
**Grade 3**<br>Hold Study regimen dose until resolution to ≤ grade 1<br>Permanently discontinue Study regimen if grade 3 irAE does not resolve to ≤ grade 1 within 30 days.	For Grade 3 or 4:<br>- Consult Neurologist<br>- Consider hospitalization<br>- Promptly initiate empiric IV corticosteroids (e.g., methylprednisolone or IV equivalent) at 1 to 2 mg/kg/day<br>- If no improvement within 3-5 days after IV steroids, consider additional workup and treatment with additional immunosuppresants (e.g. IV IGg)<br>- Once stable, gradually taper steroids over ≥ 4 weeks
**Grade 4**<br>Permanently discontinue Study regimen	

Table 24. Immune-mediated peripheral neuromotor syndromes, such as Guillain-Barre and Myasthenia Gravis

Dose Modifications		Toxicity Management
Any Grade		- The prompt diagnosis of immune-mediated peripheral neuromotor syndromes is important, since certain patients may unpredictably experience acute decompensations, which can result in substantial morbidity or in the worst case, death. Special care should be taken for certain sentinel symptoms which may predict a more severe outcome, such as prominent dysphagia, rapidly progressive weakness, and signs of respiratory insufficiency or autonomic instability<br>- Patients should be evaluated to rule out any alternative etiology (e.g., disease progression, infections, metabolic syndromes and medications, etc.). It should be noted that the diagnosis of immune-mediated peripheral neuromotor syndromes can be particularly challenging in patients with underlying cancer, due to the multiple potential confounding effects of cancer (and its treatments) throughout the neuraxis. Given the importance of prompt and accurate diagnosis, it is essential to have a low threshold to obtain a neurological consult<br>- Neurophysiologic diagnostic testing (e.g., electromyogram and nerve conduction investigations, and "repetitive stimulation" if myasthenia is suspected) are routinely indicated upon suspicion of such conditions and may be best facilitated by means of a neurology consultation<br>- Important to consider that the use of steroids as the primary treatment of Guillain-Barre is not typically considered effective. Patients requiring treatment should be considered for plasmapheresis (or IV IgG, as an alternative)
**Grade 1**	No dose modification	- Care should be taken to monitor patients for sentinel symptoms of a potential decompensation as described above<br>- Obtain a neurology consult unless the symptoms are very minor and stable

Table 24. Immune-mediated peripheral neuromotor syndromes, such as Guillain-Barre and Myasthenia Gravis

**Dose Modifications**		**Toxicity Management**
**Grade 2**	For immune-mediated peripheral neuromotor syndromes such as, Guillain-Barre, and Myasthenia Gravis-like neurotoxicity, hold Study regimen dose until resolution to ≤ grade 1.<br>Permanently discontinue study regimen if does not resolve to ≤ grade 1 within 14 days or if there are signs of respiratory insufficiency or autonomic instability.	Grade 2: Moderate<br>- Care should be taken to monitor patients for sentinel symptoms of a potential decompensation as described above<br>- Obtain a Neurology Consult<br>- Sensory neuropathy/neuropathic pain may be managed by appropriate medications (e.g., gabapentin, duloxetine, etc.)<br>*MYASTHENIA GRAVIS*<br>- Steroids may be successfully used to treat Myasthenia Gravis. Important to consider that steroid therapy (especially with high doses) may result in transient worsening of myasthenia and should typically be administered in a monitored setting under supervision of a consulting neurologist.<br>- Patients unable to tolerate steroids may be candidates for treatment with plasmapheresis or IV IgG. Such decisions are best made in consultation with a neurologist, taking into account the unique needs of each patient.<br>- If Myasthenia Gravis-like neurotoxicity present, consider starting acetylcholine esterase (AChE) inhibitor therapy in addition to steroids. Such therapy, if successful, can also serve to reinforce the diagnosis.<br>*GUILLAIN-BARRE:*<br>- Important to consider here that the use of steroids as the primary treatment of Guillain-Barre is not typically considered effective. Patients requiring treatment should be considered for plasmapheresis (or IV IgG, as an alternative).
**Grade 3**	Hold Study regimen dose until resolution to ≤ grade 1<br>Permanently discontinue Study regimen if Grade 3 irAE does not resolve to ≤ grade 1 within 14 days or if there are signs of respiratory insufficiency or autonomic instability	For severe or life threatening (Grade 3 or 4) events:<br>- Consider hospitalization<br>- Monitor symptoms and obtain neurological consult<br>*MYASTHENIA GRAVIS*<br>- Steroids may be successfully used to treat Myasthenia Gravis. It should typically be administered in a monitored setting under supervision of a consulting neurologist.<br>- Patients unable to tolerate steroids may be candidates for treatment with plasmapheresis or.<br>- If Myasthenia Gravis-like neurotoxicity present, consider starting acetylcholine esterase (AChE) inhibitor therapy in addition to steroids.

**Table 24**. Immune-mediated peripheral neuromotor syndromes, such as Guillain-Barre and Myasthenia Gravis		
**Dose Modifications**		**Toxicity Management**
**Grade 4**	Permanently discontinue Study regimen	Such therapy, if successful, can also serve to reinforce the diagnosis.<br>*GUILLAIN-BARRE*:<br>- Important to consider here that the use of steroids as the primary treatment of Guillain-Barre is not typically considered effective. Patients requiring treatment should be considered for plasmapheresis (or, as an alternative).

3.4.4.3 Management of new or worsening Pulmonary Symptoms with olaparib and/or Durvalumab

Pneumonitis has been seen with olaparib in less than 1% of patients treated, and is a potential effect of Durvalumab. If new or worsening pulmonary symptoms such as shortness of breath develop, or pulmonary abnormalities on radiological study, an interruption in study dosing is recommended, and diagnostic workup (including CT scan) should be performed, to exclude pneumonitis **(see Table 18. Pneumonitis/Interstitial Lung Disease (ILD)**. Following investigation, if no abnormalities are seen and/or prior abnormalities resolve, then olaparib may be restarted as deemed appropriate by the PI.

3.4.4.4 Management of Drug Overdose with Durvalumab, Olaparib, or Cediranib

- There is no data on overdose with Durvalumab or olaparib. In the event of overdose, appropriate supportive management should be instigated.
- There is limited clinical experience with overdose of cediranib. In the event of overdose, appropriate supportive management should be instigated. There is no known antidote for cediranib.

3.4.4.5 Management of Durvalumab, Olaparib, or Cediranib for Surgery/Procedure

- At this time there is no recommendation for holding Durvalumab in the event of surgery; decision to defer or hold treatment in the event of surgery is per discretion of the PI.
- Olaparib should be stopped at least 3 days prior to planned surgery. After surgery, olaparib may be restarted when the wound has healed. For biopsies, olaparib may be continued.
- Cediranib has the potential to affect wound healing. Cediranib should be stopped two weeks prior to elective surgery and restarted when the wound has healed. In the event of emergency surgery, cediranib should be stopped and appropriate precautions should be taken to minimize potential risks of bleeding and thrombosis associated with this class of agents.

- Cediranib and olaparib should be discontinued for a minimum of 3 days before administration of a therapeutic or palliative radiation treatment. Study treatments should be restarted within 4 weeks after completion of radiation treatment as long as bone marrow toxicity has recovered.

3.4.5 Gastrointestinal disorders

Guidelines on management of diarrhea and colitis in patients receiving Durvalumab are provided in **Table 19**.

3.4.6 Endocrine disorders

Immune-mediated endocrinopathies include hypophysitis, adrenal insufficiency, and hyper- and hypothyroidism. Guidelines for the management of patients with immune-mediated endocrine events are provided in **Table 22**.

3.4.7 Pancreatic disorders

Immune-mediated pancreatitis includes autoimmune pancreatitis, and lipase and amylase elevation. Guidelines for the management of patients with immune-mediated pancreatic disorders are provided in **Table 17**.

3.4.8 Neurotoxicity

Immune-mediated nervous system events include encephalitis, peripheral motor and sensory neuropathies, Guillain-Barré, and myasthenia gravis. Guidelines for the management of patients with immune-mediated neurotoxic events are provided in **Table 23**.

3.4.9 Nephritis

Consult with Nephrologist. Monitor for signs and symptoms that may be related to changes in renal function (e.g. routine urinalysis, elevated serum BUN and creatinine, decreased creatinine clearance, electrolyte imbalance, decrease in urine output, proteinuria, etc)

Patients should be thoroughly evaluated to rule out any alternative etiology (e.g., disease progression, infections etc.)

Steroids should be considered in the absence of clear alternative etiology even for low-grade events (grade 2), in order to prevent potential progression to higher grade event. Guidelines for the management of patients with immune-mediated neurotoxic events are provided in **Table 17**.

3.5 Study Calendar

Procedure	Screening	Cycle 1		Cycles 2 and beyond		***Post Therapy Follow-up***[11]
		**Baseline/ Day 1**	**Day 15**	**Day 1**	**Day 15**	
History and PE	X	X[1]		X[1]		
Height	X	X[1]				
Weight[2]	X	X	X[2]	X	X[2]	
Vital signs[3]: Temp, BP, pulse	X	X	X*	X	X[3]	
Performance Score[1]	X	X[1]		X[1]		
Disease confirmation by CT scans	X					
Screening labs[4]	X					
Documentation of gBRCAm status if known (For TNBC; gBRCAm status will be required)	X					
NIH Advance Directives Form[12]	X					
Protocol Labs[5]		X[1]	X*	X[1]		
Pharmacokinetics (Phase I only)		X	X*	X*		
Tumor Biopsies and archival tumor tissues[6]	X[5]	X[1]	X [1] (optional)			X (optional at progression)
Correlative Research Studies (whole blood or plasma) in selected cohorts (section 5.4)		X[1]	X	X [1] (C3D1) only		X (at progression)

Procedure	Screening	Cycle 1		Cycles 2 and beyond		***Post Therapy Follow-up***[11]
		**Baseline/ Day 1**	**Day 15**	**Day 1**	**Day 15**	
Other Specific Assessments (such as EKG/ECHO [7])	X	X		X[1]		X (at the end of treatment)
Response Evaluation (CT Scan or other as appropriate)		X[8]		Prior to each odd numbered cycle[9]		
Adverse Events		X	→	→	→	X[10]
Concomitant Medications		X[1]	→	→	→	

1 Baseline/cycle 1 day 1, day 1 of subsequent cycles of H&PE, height, performance score, protocol labs, tumor biopsies, correlative research studies, other specific assessments or concomitant medications review could be performed within 2 days before the infusion of Durvalumab. For NSCLC patients, pre-treatment biopsy could be performed within 28 days before the infusion of Durvalumab.

2 Subject will have their body weight measured on the day of infusion OR within 2 days before the infusion of Durvalumab. Subjects on the monthly Durvalumab schedule will have their body weight measured monthly on the day of infusion (approximately, every 4 weeks) OR within 2 days before the infusion of Durvalumab. Effective with Amendment E (version date 03/17/2016), the patients on Phase I study Durvalumab every 2 weeks schedule dose levels may change Durvalumab schedule to every 4 weeks (a fixed dose 1500mg) if they have clinical benefit (CR/PR/SD) more than 1 year.

3 Vital signs will be done every 2 weeks for subjects on every 2 weeks Durvalumab schedule and will be done every 4 weeks for those on monthly Durvalumab schedule except cycle 1 day 15.

For the first infusion only: subjects will have their BP and pulse measured before, during and after the infusion of Durvalumab at the following times (based on a 60-minute infusion): At the beginning of the infusion (at 0 minutes) or within 60 minutes prior to the beginning of the infusion, and at the end of the infusion (at 60 minutes ± 10 minutes).

In addition, in the 1 hour observation period post-infusion: 60 minutes after the infusion (i.e., 120 minutes from the start of the infusion) (± 30 minutes).

For subsequent infusions: subjects will have their BP and pulse measured before and after the infusion of Durvalumab at the following times (based on a 60-minute infusion): At the beginning of the infusion (at 0 minutes) or within 60 minutes prior to the beginning of the infusion, and at the end of the infusion (at 60 minutes ± 10 minutes). If the infusion takes longer than 60 minutes, then blood pressure and pulse measurements should follow the principles described here, or more frequently if clinically indicated.

4 Screening labs: CBC with differential, AST/ALT, total bilirubin, serum creatinine, PT/PTT, spot UPCR (for Durvalumab+C, C+Durvalumab and Durvalumab+O+C only), urine or serum HCG, anti HIV, anti

HCV, Hepatitis B surface antigen, and anti-CMV IgG/M. CA125 for ovarian cancer only. Please see Sections **2.7.2.1** and **2.7.2.2** for mCRPC and SCLC cohorts.

5 Protocol labs: CBC with differential, Sodium, Potassium, Chloride, Bicarbonate, BUN, serum creatinine, glucose, albumin, calcium, magnesium, phosphorus, AST, ALT, alkaline phosphatase, total bilirubin, LDH, TSH, free T4, UPCR or urinalysis, TBNK lymphocyte subsets. CA125 for ovarian cancer only. CA125 will be collected on the day 1 of cycles but not on cycle 1 day 15.

For mCRPC patients, changes in PSA and measurable lesions will be analyzed for efficacy according to the Prostate Cancer Clinical Trials Working Group 2 recommendations. PSA alone will not be utilized as a means to take a patient off-treatment.

6 Archival tissue samples will be requested from the primary surgery or the recent biopsies at entry from all study participants. Biopsies may be collected on study in Phase 2 participants per section **5.2**

7 Effective with Amendment C, (version date 12/28/2015), Electrocardiogram (EKG) is required for all patients at screening, before and after Durvalumab on C1D1, then before and after Durvalumab every 4 cycles, and at end of study treatment, as clinically indicated based on Durvalumab guidelines. EKGs recorded during the screening period will be obtained in triplicate (with 2-5 minute lag time between each); EKGs recorded during the treatment phase will be single tracing. All 12-lead EKGs should be recorded while the subject is in the supine position. The same method of assessment should be used throughout the study. Twelve-lead EKGs will be obtained after the subject has been resting in a supine position for at least 5 minutes in each case. EKGs on study will be recorded within 0 to 3 hours prior to start of Durvalumab infusion and at least one time point 0 to 3 hours after the infusion.

ECHO or MUGA is required at baseline and every 2 cycles while on study for the patients on Durvalumab+C and Durvalumab+O+C arms. Effective with Amendment S (version date 04/09/2020), patients on the Durvalumab+C and Durvalumab+O+C arms ≥ 3 years may have ECHO or MUGA every 4 cycles (+/- 7 days).

For Durvalumab+O arm, ECHO or MUGA is required at baseline and every 4 cycles while on study for those who have any of the following; 1) Prior treatment with anthracyclines; 2) Prior treatment with trastuzumab; 3) A New York Heart Association classification of II controlled with treatment ; 4) Prior central thoracic radiation therapy (RT), including RT to the heart; 5) History of myocardial infarction within the prior 12 months. Baseline ECHO or MUGA can be obtained within 10 days before enrollment. Follow-up ECHO or MUGA may be done +/- 7 days due to holidays, inclement weather, conflicts, or similar reasons.

8 Obtained within 30 days prior to initiation of therapy for mCRPC and 17 days prior to initiation of therapy for other tumor types. If the screening scan has been obtained within this timeframe, it may be used for baseline purposes.

mCRPC patients with bone disease will have baseline CT scans and technetium-99 bone scan imaging within 30 days prior to initiation of therapy.

Restaging CT scans may be done +/- 7 days due to holidays, inclement weather, conflicts, or similar reasons.

MRI may be used as clinically indicated for patients who cannot tolerate CT contrast.

9 All patients will be evaluated at baseline and prior to each cycle by history and physical examination (H&P).

All patients except mCRPC will be evaluated by imaging studies every eight weeks using RECISTv1.1 criteria (CT scan or other as appropriate).

mCRPC patients will be evaluated initially at 8 weeks by imaging studies (CT scan and technetium 99 bone scan97) and then every 12 weeks.

After PFS is determined in the phase II study, all patients who have responses a year longer than the median PFS may increase scanning interval to every 3 months at the PI's discretion. Other patients in the phase I study will continue on the every odd cycle schedule. Patients who became off- the treatment due to adverse events will be followed with CT scans until documented progression every two months (+/- 1 month). If the patient has a response (PR or CR), the response should be confirmed in ≥ 4 weeks.

Effective with Amendment O (version date 7/25/2018), the patients who achieve durable response (PR or CR) and have been on study ≥ 3 years may have response evaluation by imaging every 3 months (+/- 7 days).

10 AE data after off-treatment should be collected either by phone or by clinic visit monthly until subsequent anti-tumor intervention OR until 90 days post-the last dose of Durvalumab OR until 30 days post-last dose of olaparib or cediranib, whichever occurs later.

* PK for dose level 3 of Durvalumab+O, new dose levels of Durvalumab+C intermittent dose schedule and Durvalumab+O+C arms (phase I study) will be collected on cycle 2 day 1 given that Durvalumab is administered every 4 weeks.

* For Phase I Durvalumab+C intermittent dose schedule and Durvalumab+O+C arm will include blood collection for PD endpoints at pretreatment, on cycle 1 day 15, cycle 3 day 1 and at progression. The patients on cediranib intermittent schedule (5 days on/2days off) may begin with 2-4 days on/2 days off during the first week of the cycle to have weekdays (Monday through Friday) on/weekend (Saturday and Sunday) off schedule, subsequent administrations are then adjusted to maintain a 5 days on/2 days off-interval.

11 Patients will remain on study and followed for adverse events after drug discontinuation for a minimum of **90 days** post the final infusion of Durvalumab, OR **30 days** after the final dose of cediranib or olaparib, whichever occurs later, OR until initiation of alternative cancer therapy.

12 As indicated in section 13.3, all subjects ≥ age 18 will be offered the opportunity to complete an NIH advance directives form. This should be done preferably at baseline but can be done at any time during the study as long as the capacity to do so is retained. The completion of the form is strongly recommended, but is not required.

3.6 COST AND COMPENSATION

3.6.1 Costs

NIH does not bill health insurance companies or participants for any research or related clinical care that participants receive at the NIH Clinical Center. If some tests and procedures performed outside the NIH Clinical Center, participants may have to pay for these costs if they are not covered by insurance company. Medicines that are not part of the study treatment will not be provided or paid for by the NIH Clinical Center.

3.6.2 Compensation

Participants will not be compensated on this study.

3.6.3 Reimbursement

The NCI will cover the costs of some expenses associated with protocol participation. Some of these costs may be paid directly by the NIH and some may be reimbursed to the participant/guardian as appropriate. The amount and form of these payments are determined by the NCI Travel and Lodging Reimbursement Policy.

3.7 CRITERIA FOR REMOVAL FROM PROTOCOL THERAPY AND OFF STUDY CRITERIA

Prior to removal from study, effort must be made to have all subjects complete a safety visit approximately 90 days past the last infusion of Durvalumab, or 30 days following the last dose of cediranib or olaparib, whichever is later.

3.7.1 Criteria for removal from protocol therapy

In the absence of treatment delays due to adverse events, treatment may continue until one of the following criteria applies:

- Disease progression,
- Intercurrent illness that prevents further administration of treatment,
- Unacceptable adverse event(s): Any severe (grade 3 or 4) toxicity that has not resolved to NCI/CTEP grade 1 toxicity by three weeks or the best medical judgment of the PI or one of the associate investigators will result in the cessation of therapy.
- Patient requests to be withdrawn from an active therapy.
- Patient withdraws consent. In this event, the reasons for withdrawal will be documented and the patient will be taken off study at that time.
- If patients are non-compliant with the protocol guidelines, they may be removed from the study at the discretion of the PI.
- Positive pregnancy test

3.7.2 Follow-up

Patients will remain on study and be followed for adverse events after drug discontinuation for minimum of **90 days** post the final infusion of Durvalumab, OR **30 days** after the final dose of cediranib or olaparib, or until the patient receives the subsequent treatment after progression.

At the final visit they can be deemed off-study. In the event that a patient cannot return for a clinic visit, follow-up will be conducted in the form of a phone call.

Patients removed from treatment for unacceptable adverse events will be followed monthly until resolution or stabilization of the adverse event or until minimum of 90 days after the last infusion of Durvalumab or 30 days after the last dose of olaparib or cediranib, whichever occurs later, or until the initiation of other tumor treatment, whichever occurs later. Also, patients removed from treatment for unacceptable adverse events will be followed with CT scan every two months (+/- 1 month) until documented progression or until the initiation of other tumor treatment, whichever occurs later.

3.7.3 Off-Study Criteria

Patients will be considered off-study after:

- Withdrawal of consent
- Completion of follow-up
- Lost to follow-up
- Death
- Screen failure

3.7.4 Lost to Follow-Up

A participant will be considered lost to follow-up if he or she fails to return for one scheduled treatment visit and is unable to be contacted by the study site staff.

The following actions must be taken if a participant fails to return to the clinic for a required study visit:

- The site will attempt to contact the participant by the methods described below to reschedule the missed visit within 7 days of the missed visit, counsel the participant on the importance of maintaining the assigned visit schedule, and ascertain if the participant wishes to and/or should continue in the study.
- Before a participant is deemed lost to follow-up, the investigator or designee will make every effort to regain contact with the participant within 7 days (a minimum of 3 contact attempts using the participant's preferred method of contact via phone, email, or a combination of both; and if necessary, a letter sent through a trackable delivery service to the participant's last known mailing address. These contact attempts should be documented in the participant's medical record or study file.

Should the participant continue to be unreachable, he or she will be considered to have withdrawn from the study with a primary reason of lost to follow-up.

4 CONCOMITANT MEDICATIONS/MEASURES

4.1 PROHIBITED MEDICATIONS

- No concomitant use of alternative, complementary therapies or over-the-counter agents will be allowed without approval of the PI. All medications must be recorded in the case report form and be reviewed by the treating physician at each visit.
- Immunosuppressive medications including, but not limited to, systemic corticosteroids at doses exceeding 10 mg/day of prednisone or equivalent, methotrexate, azathioprine, and TNF-α blockers are prohibited while patients are receiving treatment with Durvalumab. Use of immunosuppressive medications for the management of investigational product-related AEs or in subjects with contrast allergies is acceptable. In addition, use of inhaled and intranasal corticosteroids is permitted.
- Live attenuated vaccines within 30 days of Durvalumab dosing (ie, 30 days prior to the first dose, during treatment with Durvalumab and for 30 days post discontinuation of Durvalumab). Inactivated viruses, such as those in the influenza vaccine, are permitted.
- Oral anticoagulants such as warfarin are not contraindicated during treatment with cediranib, provided that there is increased vigilance with respect to monitoring INR. If medically

appropriate, low molecular weight heparin may be considered preferable to warfarin, due to shorter half-life and more predictable anticoagulant effect.

- For patients on cediranib or olaparib, caution should be exercised in the concomitant use of any medication that may markedly affect renal function. Such medications may be used with caution as deemed essential for treatment, or if already in use prior to entry in the study without any effect on renal function.
- For patients on cediranib or olaparib, caution should be exercised in concomitant use of any medication that may significantly affect hepatic CYP450 drug metabolizing activity by way of enzyme induction (e.g. Phenytoin) or inhibition (e.g. ketoconazole, ritonavir, erythromycin) within 2 weeks before the first dose of cediranib/olaparib and throughout the study period.
- Based on *in vitro* and clinical exposure data, olaparib is considered unlikely to cause clinically significant drug interactions through inhibition or induction of cytochrome P450 enzyme activity. However, *in vitro* data have also shown that cytochrome P450 3A4 (CYP 3A4) is the principal isoenzyme responsible for the formation of the 3 main metabolites of olaparib. Formal *in vivo* drug-drug interaction studies have not yet been conducted for an investigational tablet formulation of olaparib.
- Given this data, potent inhibitors or inducers of CYP3A4 (as outlined in **Appendix B**) must not be used during this study for patients receiving olaparib. Dihydropyridine calcium-channel blockers are allowed for management of hypertension.

  - CYP3A4 known potent inhibitors: wash-out period 1 week, or at least 4.5x elimination half-lives for drugs and metabolites known to inhibit CYP3A subfamily enzymes: Ketoconazole, itraconazole, ritonavir, indinavir, saquinavir, telithromycin, clarithromycin and nelfinavir
  - CYP3A4 inducers (potential reduction in efficacy of olaparib) a wash-out period for 3 weeks for Phenytoin, rifampicin, rifapentin, rifabutin, carbamazepine, phenobarbital, nevirapine, modafinil and St John's Wart.

4.2 Supportive Care

Patient should receive general concomitant and supportive care medications (including antibiotics, nutritional support, growth factor support, correction of metabolic disorders, optimal symptom control, and pain management, etc.) based on best medical practice. Supportive care can include palliative radiation therapy, including bone-directed radiation therapy for pain control in subjects deriving clinical benefit from treatment. Specific management for adverse events related to study drugs can be found in section **3.4**.

5 CORRELATIVE STUDIES

The primary goal of the correlative studies is to identify potential predictive biomarkers of response to immune checkpoint inhibition with a PARP inhibitor or VEGFR inhibition. Although PD-L1 expression in tumor was highly predictive of response to a PD-1 inhibitor, nivolumab in treatment-naive lung cancer patients (50% in PD-L1–positive patients v. 0% in PD-L1–negative patients), this was not the case for pretreated patients (15% in PD-L1–positive vs

14% in PD-L1–negative; Brahmer, J Clin Oncol. 2014;32(suppl 5S):abstr 8021). It is still unclear what value is an optimal PD-L1 expression cutoff, questions of assay sensitivity, and use of archival tissue v. "fresh" pretreatment biopsy. Several ongoing phase III trials of nivolumab in advanced NSCLC require mandatory tissue samples and will explore the role of PD-L1 as a predictive biomarker for nivolumab. Thus, pretreatment biopsy samples will provide important information on development of predictive biomarkers in ovarian cancer patients. Other correlative study aims include investigation of proof-of-concept biomarkers of immune checkpoint inhibition and DNA damage repair or angiogenesis inhibition in recurrent ovarian cancer. These will be performed in an exploratory fashion.

- Phase II only: Patients will undergo one mandatory pretreatment biopsy and two optional research biopsies (prior to cycle 1 day 15 and at progression) to assess the effect of response to Durvalumab with olaparib or cediranib therapy at minimal risk.
- Blood samples will be collected pretreatment, cycle 1 day 15, before cycle 3 day 1 of treatment (approximately C2D29), and at progression for Phase I Durvalumab+C intermittent schedule, Durvalumab+O+C cohorts and Phase II all cohorts.
- Should patients undergo thoracentesis or paracentesis (pretreatment or while on treatment), fluid samples are optional. A minimum of 50 ml of non-heparinized effusion will be picked up by the Lee lab. Ten aliquots of supernatant and 10 viably frozen cell pellets will be stored in liquid nitrogen until use. Correlative studies including but not limited to immune subsets, DNA damage repair, angiogenesis and other pathways will be done in collaboration with Dr. Patricia Steeg of WMB/CCR/NCI.
- For Phase II CRC cohort (C+Durvalumab) only: The correlative studies to be performed are summarized in **Table 28**. All samples will be sent to Dr. Figg's lab for processing and storage until they are distributed to Dr. Greten's lab or MedImmune/Quintiles for sample analysis as described in the protocol. Collaborator(s) of MedImmune/Quintiles has yet to be determined, but will be submitted for IRB approval via a future amendment prior to the shipment of any samples during the Phase II portion of the trial.
- For Phase II prostate cancer cohort patients only: A one-time saliva sample will be collected anytime during the study as a source of normal tissue for genomics comparison. Saliva sample will be collected in one Oragene Saliva Kit at room temperature. Please e-mail NCIBloodcore@mail.nih.gov at least 24 hours before transporting samples (the Friday before is preferred).

For sample pickup, page 102-11964.

For immediate help, call 240-760-6180 (main blood processing core number) or, if no answer, 240-760-6190 (main clinical pharmacology lab number).

For questions regarding sample processing, contact NCIBloodcore@mail.nih.gov.

Dr. Figg's lab will barcode the sample, remove patient identifier, and send the sample to Dr. Meltzer's lab via courier. Patients will be instructed not to eat, drink, smoke or chew gum for 30 minutes before giving their saliva sample. Sequencing of DNA will be performed by the Genetics Branch/CCR/NCI (Drs. Khan/Meltzer).

- All patients on the study are requested to submit a block of tissue or 20 paraffin-embedded unstained slides from a recent resection or barring that, from the original surgery. All efforts will be made to obtain these samples to achieve the secondary objectives.
- The following tests will be performed on patients' specimens, in order of priority (**Table 25**).

Table 25. Correlative studies for Phase II Ovarian cancer cohort 1

PBMC	***Plasma/whole blood***	***Archived tissue samples***	***Tumor and/or Effusion Cells***
Immune subsets by Flow<br>Circulating endothelial cells	Comprehensive cytokine/chemokine<br>Circulating tumor cells<br>Cell free DNA<br>Pharmacokinetics (Phase I only)	IHC for PD-L1 expression<br>BROCA-HR next generation sequencing and whole exome sequencing<br>NF-kB activity<br>Immune subsets	Immune subsets by RNAseq or IHC<br>IHC for PD-L1 expression<br>BROCA-HR next generation sequencing and/or whole exome sequencing<br>Tissue lysate array for angiogenesis and DNA repair endpoints<br>STING pathway activation<br>NF-kB activity

Table 26. Correlative studies for Phase II TNBC cohort 5

PBMC	***Plasma/whole blood***	***Archived tissue samples***	***Tumor (and/or Effusion Cells)***
Immune subsets by flow	Comprehensive cytokine / chemokine<br>Circulating tumor cells<br>Cell free DNA	IHC for PD-L1 and peritumoral lymphocytes expression<br>BROCA-HR next generation sequencing and whole exome sequencing<br>Immune subsets	RNAseq for TNBC molecular sybtypes<br>IHC for PD-L1 expression and peritumoral lymphocytes /macrophages expression<br>BROCA-HR next generation sequencing and whole exome sequencing pre and post olaparib treatment<br>Tissue lysate array for DNA repair endpoints

Table 27. Correlative studies for Phase II NSCLC (cohort 2) and SCLC (cohort 3)

Sample	***Assay***	***Volume blood***	***Type of tube***	***Time points***	***Location***
Tumor	Exome / transcriptome	NA	Flash frozen	Pre-treatment on cycle 1 day 1, Two optional biopsies (prior to cycle 1 day 15 and at progression)	IR/ Trepel lab for processing
Peripheral blood	Immune subsets	16 ml	Two 8mL BD Vacutainer Cell Preparation Tubes (CPT; blue and black top)	Pre-treatment on cycle 1 day 1, prior to cycle 1 day 15 and cycle 3 day 1, and at progression)	Trepel lab
Plasma	Comprehensive cytokine / chemokine	20 ml	Two 10-mL EDTA tubes (lavender top tubes)	Pre-treatment on cycle 1 day 1, prior to cycle 1 day 15 and cycle 3 day 1, and at progression)	Figg Lab
Circulating tumor cells	Enumeration and gene expression	20 ml	Two 10-mL EDTA tubes (lavender top tubes)	Pre-treatment on cycle 1 day 1, prior to cycle 1 day 15 and cycle 3 day 1, and at progression)	Trepel lab
Whole blood	Transcriptome (SCLC only)	2.5ml	One 2.5 mL PAXgene RNA tubes	Pre-treatment on cycle 1 day 1, prior to cycle 1 day 15 and cycle 3 day 1, and at progression)	Trepel lab
Whole blood	Assays for paraneoplastic autoantibodies (SCLC only)	10 ml	One red top tube (10ml)	Baseline only	Trepel lab

Sample	***Assay***	***Volume blood***	***Type of tube***	***Time points***	***Location***
Whole blood	NanoString Pan Cancer Immune Panel (SCLC only)	2.5ml	One 2.5-ml PAXgene RNA tubes	Pre-treatment on cycle 1 day 1, prior to cycle 1 day 15 and cycle 3 day 1, and at progression)	Trepel lab

Table 28. Correlative studies for Phase II Colorectal cancer cohort 6

**Test/assay**	**Volume blood (approx)**	**Type of tube**	**Collection point (+/- 48hrs)**	**Location of specimen analysis**
Immune- monitoring	120 mL (for PBMC)	Twelve 10 mL EDTA	Q28d	Greten Lab
	5-10 mL (for serum)	One 10 mL EDTA Tube		
T cell activation/ICO S expression (PBMC)	12 mL	Two 6 mL EDTA collection tubes	Q28d	Greten Lab or Medimmune/ Quintiles
Plasma-based assays for circulating receptors/ligands e.g. PDL1	6 mL	One 6ml EDTAtube	Q28d	Greten Lab or Medimmune

Test/assay	Volume blood (approx)	Type of tube	Collection point (+/- 48hrs)	Location of specimen analysis
Tumor biopsy	NA	NA	Mandatory Baseline (cycle 1 day -14 and two optional biopsies (prior to cycle 1 day 1 and at progression)	Laboratory of Pathology

Table 29: Correlative studies for phase II mCRPC cohort 4

Test/Assay	Volume blood (approx.)	Type of Tube	Number of Tubes	Location of specimen analysis	Collection Time Point
Chemokine and cytokine analysis	10 cc	Purple	2	Blood Processing Core	C1D1, C1D15, C3D1, Progression/end of treatment
PBMC	8 cc	Blue/Black	2	Trepel lab	C1D1, C1D15, C3D1, Progression/end of treatment
CTC	10 cc	Purple	2	Trepel lab	C1D1, C1D15, C3D1, Progression/end of treatment
Nanostring Immune Panel	2.5 cc	PAX red	1	Trepel lab	C1D1, C1D15, C3D1, Progression/end of treatment
PAXgene	2.5 cc	PAX red	1	Trepel lab	On-study, post cycle 2
Tumor biopsy	NA	NA	NA	Laboratory of Pathology, and Meltzer lab (if sufficient	Mandatory baseline

Test/Assay	Volume blood (approx.)	Type of Tube	Number of Tubes	Location of specimen analysis	Collection Time Point
				sample) and/or Kelly lab (if sufficient sample)	
Oragene Saliva Kit	NA	NA	NA	Meltzer lab	One time sample on-study or during treatment

5.1 RATIONALE FOR SELECTED ENDPOINTS

See section 1.3 for details.

5.2 TUMOR BIOPSIES (PHASE II STUDY ONLY)

Timing*

Biopsies will be performed at the following times:

- Mandatory – after consent, prior to treatment on cycle 1 day 1
- Optional - prior to cycle 1 day 15 +/- 48 hours
- Optional - at the time of progression

*Biopsies may be not performed on the specific dates and times due to the following reasons, including but not limited to, delayed recovery of hematologic toxicities, delayed clinic schedule, or national holidays.

* See **Figure 9** for the biopsy time points of CRC cohort.

Tissue Sampling and Handling

- Attempts will be made to obtain up to four cores if safe and feasible, which will be frozen for research studies. These tumor core biopsies will be obtained percutaneously or per vagina through interventional radiology as long as considered minimal surgical risk. Two 3-millimeter punch biopsies of skin will be acceptable in lieu of 18 gauge core biopsies for patients with skin involvement. Inability to get tissue with a reasonable attempt will not preclude treatment and the patient will remain eligible for all other translational components.
- The use of imaging to facilitate biopsies will be decided upon by members of the interventional radiology team. Should CT scans be needed for biopsy, a limit of 10 scans for each procedure will be observed to minimize radiation exposure to the patient.

- For Phase II OvCa and TNBC cohorts 1 and 5: the schedule for the biopsies will be made with Special Procedures (Dr. Brad Wood). Members of the Lee lab will be on call to receive and embed biopsies: jayakumar.nair@nih.gov, Phone (301) 451-8403, beeper 102-11155. The biopsies are to be immediately embedded, frozen, and stored, barcoded, in the Lee lab at -80 °C on site until use according to our laboratory SOP (**Appendix G**). At least one tissue core will be paraffin-embedded, barcoded and stored in the Lee lab until use (**APPENDIX H**).
- For Phase II NSCLC or SCLC cohorts 2 and 3: the Trepel lab will be contacted by email to trepel@helix.nih.gov and Sunmin Lee; lees@pop.nci.nih.gov. Interventional Radiology will call the lab at 240-760-6330 when the patient arrives in IR and a lab member will be present at Interventional Radiology for the procedure. The cores will be flash frozen, 2D barcoded, and stored in liquid nitrogen. Additional core biopsies in liquid nitrogen will be obtained at each biopsy time point if deemed safe and feasible to study changes in the tumor microenvironment and gene expression after treatment with Durvalumab and olaparib. These samples will be used for future correlative studies including whole exome and transcriptome sequencing.
- For Phase II mCRPC cohort 4: core biopsies will be obtained via Interventional Radiology and members of the Kelly lab will be on call to receive the biopsies: mike.beshiri@nih.gov, phone 301.594.2529, pager 102-12110.
- For Phase II CRC cohort 6: core biopsies will be obtained via Interventional Radiology and Milan Sandhu will be on call to receive the biopsies: milan.sandhu@nih.gov, phone 603-714-9453 (page 109). For CRC correlative studies please refer to section 5.3.2.1.

5.3 Studies Performed

5.3.1 PD-L1 expression by IHC and/or other immune subset markers by IHC or RNAseq (All patients)

Preliminary biomarker data suggest tumor PD-L1 expression by IHC status in the tumor microenvironment may be associated with anti-tumor response to PD-1 or PD-L1 inhibitors. Daud *et al.* reported preliminary findings on PD-L1 expression in 135 evaluable patients with advanced melanoma at 2014 AACR, from the ongoing study of MK-3475, a PD-1 inhibitor[161]. Tumor PD-L1 expression was assessed by IHC. A preliminary cutoff of 1% of stained cells was used to define PD-L1 positivity. Overall RR was 41% in the 116 patients with measurable disease. Among 71 patients with measurable disease and interpretable IHC results, PFS and RR were significantly associated with tumor PD-L1 expression (median PFS of 10.6 months v. 2.9 months, p=0.034 RR=53% (29/55) v. 6% (1/16), p<0.004 in PD-L1 positive group compared to negative group, respectively).

The expression of PD-L1 and/or other immune subsets including but not necessarily limited to CD3+, CD4+, CD8+ and Foxp3 by IHC and/or by RNAseq at pretreatment core biopsy or archival tissue samples to correlate with response for Phase I study and OvCa and TNBC cohorts of Phase II study will be studied in collaboration with Dr. Cimino-Mathews at Johns Hopkins University Hospital.

Coded patient samples will be batched to

Ashley Cimino-Mathews, MD

Assistant Professor, Department of Pathology

The Johns Hopkins Hospital

401 N. Broadway St, Rm 2242

Baltimore, MD21287

Phone; 410-955-3580

Email: acimino@jhmi.edu

Collaborator(s) for SCLC, NSCLC, mCRPC and CRC cohorts has yet to be determined, but will be submitted for IRB approval via a future amendment prior to the assignment of any samples during the Phase II portion of the trial.

5.3.1.1 BROCA-HR next gene sequencing and whole exome sequencing (All Phase II except CRC patients)

Emerging data suggest that tumor mutational loads and neo-antigens may correlate with clinical response to immune checkpoint inhibition[162] [29]. It has been reported that mutated peptides resulting from DNA mutations are recognized by CD8+ T-cells and CD4+ T-cells in a large fraction of melanoma patients, and these neo-antigen-specific T cell responses are likely to contribute to the clinical effects of cancer immunotherapy[162] [163] [164]. Anonymized patient samples will be provided to a collaborator or core lab/company for a fee-for-service for next generation sequencing or whole exome sequencing to elucidate other DNA damage repair genes, the mutational loads resulting from mutations in genes involved in DNA damage repair pathways and potential neo-antigens as an exploratory endpoint.

- Core biopsy samples from OvCa and TNBC cohorts of Phase II study will be processed for BROCA panel by Laboratory Medicine at University of Washington in a fee-for-service transaction.
- Recent data also indicate subsets of mCRPC and lung cancers have dysfunctions in genes related to DNA damage repair pathways. The purpose of the expansion cohorts is to test the hypothesis that the PD-L1 inhibitor, Durvalumab in combination with olaparib can be safely administered and may yield clinical benefit in patients with these diseases. Exome sequencing will be done for mCRPC cohort to investigate potential predictive biomarkers in responders and non-responders in collaboration with Javed Khan and Paul Meltzer in the Genetics Branch/CCR/NCI. Additionally, culturing of patient-derived organoids (cell lines) will be performed in collaboration with Kathleen Kelly Siebenlist in the Laboratory of Genitourinary Cancer Pathogenesis/CCR/NCI, if there is biopsy core specimen available.
- Collaborator(s) for SCLC, NSCLC or CRC cohorts has yet to be determined, but will be submitted for IRB approval via a future amendment prior to the shipment of any samples during the Phase II portion of the trial.

All samples sent to the Blood Processing Core (BPC) will be barcoded, with data entered and stored in Labmatrix utilized by the BPC. This is a secure program, with access to Labmatrix limited to defined Figg lab personnel, who are issued individual user accounts. Installation of Labmatrix is limited to computers specified by Dr. Figg. These computers all have a password restricted login screen.

Labmatrix creates a unique barcode ID for every sample and sample box, which cannot be traced back to patients without Labmatrix access. The data recorded for each sample includes the patient ID, name, trial name/protocol number, time drawn, cycle time point, dose, material type, as well as box and freezer location. Patient demographics associated with the clinical center patient number are provided in the system. For each sample, there are notes associated with the processing method (delay in sample processing, storage conditions on the ward, etc.).

The results of molecular studies conducted using specimens are for research purposes only and will not be disclosed to individual subjects. The exception to this is potential incidental findings that are deemed clinically significant and actionable. A scientific collaborator will notify these findings to the PI. The policy for this disclosure from the PI to the patient is outlined below and in the Phase II consent form for this study.

1. Subjects will be contacted if a clinically actionable gene variant is discovered. Clinically actionable findings for the purpose of this study are defined as disorders appearing in the American College of Medical Genetics and Genomics recommendations for the return of incidental findings that is current at the time of primary analysis. (A list of current guidelines is maintained on the CCR intranet: https://ccrod.cancer.gov/confluence/display/CCRCRO/Incidental+Findings+Lists)

   Subjects will be contacted at this time with a request to provide a blood sample to be sent to a CLIA certified laboratory. If the research findings are verified in the CLIA certified lab, the subject will be referred to a genetics counseling service for the disclosure of the results.

   This is the only time during the course of the study that incidental findings will be returned. No interrogations regarding clinically actionable findings will be made after the primary analysis.

2. Funding mechanism for the CLIA laboratory confirmation will be decided upon discussion with the patients, and/or appropriate funding resources at CCR.
3. Subject's genetic data will be deposited in a database such as database of Genotypes and Phenotypes (dbGaP). Although there is controlled access to such a database, such a submission carries theoretical risks of revealing the identity of the subject. This is discussed in the Phase II consent.

5.3.1.2 NF-κB activity (Phase II ovarian cancer only)

NF-kB activity in ovarian cancer can be assessed by presence of tumor-infiltrating lymphocytes and nuclear NF-kB p65 in tumor cells by IHC, or expression of the 9-gene NF-kB signature and the Immunoreactive gene panel by gene expression profiling. We hypothesize that these markers

could serve as biomarkers predictive of response in future clinical trials with immune checkpoint inhibitors.

Archived tumor tissue will be evaluated by IHC for the presence of nuclear NF-kB p65 as an estimate of NF-kB activation, in collaboration with Dr. Annunziata, WMB/CCR. If sufficient material from archived tumor samples, RNA will be extracted and gene expression profiled on Affymetrix U133 arrays, in order to classify cases into pre-defined subsets[5,153] and quantify expression of the NF-kB gene signature. Alternatively, quantitative PCR will be used. Freshly obtained tumor biopsies will be formalin-fixed and paraffin-embedded, and subjected to similar analyses.

5.4 BLOOD SAMPLES

The following tests will be performed on patients' peripheral blood samples, in order of priority (Phase I Durvalumab+C intermittent schedule cohort, Phase I Durvalumab+O+C cohort and Phase II study only). These correlative studies will be done in an exploratory fashion.

Timing

Blood samples will be collected prior to treatment on cycle 1 day 1, prior to cycle 1 day 15 +/- 48 hours, cycle 3 day 1, and at progression.

For colorectal cancer patients, blood for immune subsets monitoring will be collected every 28 days until off-treatment.

5.4.1 Studies Performed

5.4.1.1 Immune subset markers (Phase I Durvalumab+C intermittent schedule arm, Phase I Durvalumab+O+C arm and all Phase II participants except CRC cohort)

Emerging data suggest there is interplay between chemotherapies and the host immune system[30,165]. Further, tumors with high genomic instability, such as ovarian cancer, may trigger host immune reaction, which may correlate with patient clinical outcome[56]. Hence, monitoring immune subsets in ovarian cancer patients may facilitate the establishment of personalized therapy in ovarian cancer.

PBMCs will be processed and stored at Trepel Lab except colorectal cancer patients, and will be assessed using multi-parameter flow cytometry for immune subsets including but not necessarily limited to Tregs, MDSC, effector and exhausted CD8+ T-cells. Assessment will include functional markers, i.e. PD-1, TIM3, CTLA-4 and/or CD40. This study will be done in collaboration with Jane Trepel of Developmental Therapeutics Branch/CCR/NCI.

- PBMC Collection of Specimen(s): Two 8-ml BD Vacutainer Cell Preparation Tubes (CPT; blue and black top) will be collected from each patient. Immediately after collection, mix the blood sample by gentle inversion several times. The date and exact time of each blood draw should be recorded on the tube.
- Notification and Handling of Samples for all patients in Phase II study except colorectal cancer patients: as soon as possible after the patient is scheduled please send email notification to the Trepel lab at trepel@helix.nih.gov; Min-Jung Lee at leemin@mail.nih.gov

and yusuke.tomita@nih.gov) and call the Trepel lab at 240-760-6330 to arrange for immediate pick-up when the sample is drawn.

- For colorectal cancer patients (cohort 6) on Durvalumab+C arm,
  - Notification and Handling of Samples: as soon as possible after the patient is scheduled please send email notification to Melissa Walker RN: email: walkerme@mail.nih.gov phone 301.827.3456.
  - PBMC Collection of Specimen(s): Thirteen 10-ml EDTA tubes, three 6mL EDTA tubes, will be collected from each patient (**Table 28**).
  - Tumor biospies will be tested in by IHC and nanostring array. PBMC will be tested for a immune cell subsets including but not limited to CD4/CD8 effector and memory, Tregs, Th subsets, activation markers, myeloid subsets including MDSC and DC, NKs.

5.4.1.2 Comprehensive chemokine and cytokine analysis (Phase I Durvalumab+C intermittent schedule arm, Phase I Durvalumab+O+C arm and all Phase II participants except CRC cohort)

- The cytokine analysis may be done for Phase 1 Durvalumab+O and Durvalumab+C daily schedule cohorts if remaining plasma samples from PK draws are available.
- The MSD platform chemokine and cytokine panel study will be done in collaboration with Dr. Liang Cao of Molecular Targets Core (Clinical Biomarker Core)/Genetics Branch/CCR/NCI.
- Plasma samples will be processed using a Duke Angiome Panel by Phase I Biomarker Laboratory at Duke University in a fee-for-service transaction. Coded and linked frozen plamsa samples will be batched to

Andrew B. Nixon, Ph.D., M.B.A
Director, Phase I Biomarker Laboratory
Duke University Medical Center
395 MSRB Building
Research Dr.
Durham, NC 27710
Phone: 919-613-7883
FAX: 919-668-3925
Email: anixon@duke.edu

- Two 10 ml-EDTA lavender top tubes will be collected from each patient.
- The tubes will be immediately packed on wet ice and picked up by the Clinical Pharmacology Core (CPC). Please e-mail NCIBloodcore@mail.nih.gov at least 24 hours before transporting samples (the Friday before is preferred).

For sample pickup, page 102-11964.

For immediate help, call 240-760-6180 (main blood processing core number) or, if no answer, 240-760-6190 (main clinical pharmacology lab number).

For questions regarding sample processing, contact NCIBloodcore@mail.nih.gov.

Serum and plasma will be separated and aliquoted according to the CPC SOPs and stored, barcoded, in the CPC repository until use.

5.4.1.3 Circulating Endothelial Cells (CEC: Phase I Durvalumab+C intermittent schedule arm, Phase I Durvalumab+O+C arm and Phase II ovarian cancer cohort only)

- Two 8 ml- CPT (blue and black top) will be collected from each patient.
- Notification and Handling of Samples: as soon as possible after the patient is scheduled please send email notification to the Trepel lab at trepel@helix.nih.gov; Min-Jung Lee at leemin@mail.nih.gov) and call the Trepel lab at 240-760-6330 to arrange for immediate pick-up when the sample is drawn.
- The sample is to be kept at room temperature until pick up.

5.4.1.4 Circulating tumor cells (CTC) (Phase I Durvalumab+C intermittent schedule arm, Phase I Durvalumab+O+C arm and all Phase II participants except CRC cohort)

- Blood will be collected to correlate changes in CTC[166] enumeration with clinical response. CTC will be investigated using ferrofluidic enrichment and multi-parameter flow cytometric detection. CTCs are identified by positive expression of epithelial markers and a viability marker and negative expression of hematopoietic markers. Immune markers including but not limited to functional markers, i.e. PD-1 or PD-L1 in CTC will be studied.
- Two 10-mL EDTA tubes (lavender top tubes) will be collected from each patient. Plasma from these samples will be stored at Trepel lab after CTCs are harvested.
- Immediately after collection, invert the blood tubes 3-4 times.
- Notification and Handling of Samples: as soon as possible after the patient is scheduled please send email notification to the Trepel lab: Jane Trepel at trepel@helix.nih.gov; Min-Jung Lee at leemin@mail.nih.gov that the sample is scheduled. As soon as the sample is drawn, please call the Trepel lab at 240-760-6330 to communicate that the sample is ready. Keep the sample on the unit at room temperature. The sample will be picked up by the lab and processed for CTC enumeration.

5.4.1.5 Cell free DNA (Phase II ovarian and TNBC cohorts only)

- One 10 ml cell free DNA BCT Streck® tube will be collected from each patient. DNA will be isolated and assessed by QIAamp Circulating Nucleic Acid Kit or other platform as appropriate in the Lee Lab.
- The key is to maximize removal of contaminating cells and genomic DNA from these cells as large genomic DNA fragments hinder plasma DNA analysis.
- **CRITICAL STEP**: Time between blood draw and processing should be **less than 1 hour**. Gently invert tubes after blood draw. Do not shake or vortex tubes as cellular lysis could occur.

- Notification and Handling of Samples: members of the Lee lab on call to receive specimens: jayakumar.nair@nih.gov, Phone (301) 451-8403, beeper 102-11155.

5.4.1.6 SCLC transcriptome (Phase II SCLC cohort only)

- Peripheral blood will be drawn into one 2.5 ml PAXgene RNA tube at baseline, after 2 cycles of treatment and at disease progression. RNA will be isolated and assessed by NanoString, droplet digital PCR, RNA-Seq or other platform for DNA repair gene panel as appropriate. These assessments will be performed by the Trepel lab.
- Notification and Handling of Samples: as soon as possible after the patient is scheduled please send email notification to the Trepel lab at trepel@helix.nih.gov; Min-Jung Lee at leemin@mail.nih.gov) and call the Trepel lab at 240-760-6330 to arrange for immediate pick-up when the sample is drawn.

5.4.1.7 Pharmacokinetic Studies (Phase I study only)

During the phase I portion of the study, pharmacokinetics for olaparib and cediranib will be evaluated. Blood will be collected as below. Analyses will be done by the CPC.

- Samples will be analyzed using an ultra HPLC with tandem mass spectrometric detection (UPLC-MS/MS), validated for processing human samples.
- The samples are to be drawn, placed on wet ice, and refrigerated. The sample label should contain the following information: Cycle number, Day number, time after dosing, and exact draw date and time.

  Please e-mail NCIBloodcore@mail.nih.gov at least 24 hours before transporting samples (the Friday before is preferred).

  For sample pickup, page 102-11964.

  For immediate help, call 240-760-6180 (main blood processing core number) or, if no answer, 240-760-6190 (main clinical pharmacology lab number).

  For questions regarding sample processing, contact NCIBloodcore@mail.nih.gov.
- Serum and plasma will be separated and aliquoted according to the CPC SOPs and stored, barcoded, in the CPC repository until use.
- Blood samples (7ml green top [heparin] tube) for olaparib measurement by LC-MS/MS will be drawn around the first dose on C1D1 at: pre-dose, 30min, 1hr, 2hr, 4hr, 8hr, and 12hr (just before PM dose; +/- 5 min is allowed for each time points except 12hr time point; +/- 2 hr is allowed for 12 hr time point).
- Blood samples (7ml green top [heparin] tube) for cediranib measurement by LC-MS/MS will be drawn around the first dose on C1D1 at: pre-dose, 30min, 1hr, 2hr, 4hr, 8hr, and 12hr (+/- 5 min is allowed for each time points except 12hr time point; +/- 2 hr is allowed for 12 hr time point).

5.5 RADIOMICS

5.5.1 Rationale

- Tissue based biomarkers remain the gold standard for pharmacodynamics studies but need to be carefully calibrated to the most informative tumor region, which is currently very difficult to achieve in a multi-site heterogeneous disease such as recurrent ovarian cancer. The addition of radiomics to the correlative studies conducted under this protocol will provide an opportunity to explore the correlation of radiomics changes with clinical outcome.
- Professor Sala's lab at the University of Cambridge in U.K. has been a leading expert in the quantitative assessment of image features, known as radiomics, using their artificial intelligence tools for (a) automatic whole volume tumor segmentation and (b) deriving predictive and prognostic models of response and outcome. Radiomics biomarkers therefore could serve as "virtual biopsies" and may provide biomarkers reporting changes in both tumor and microenvironment during therapy and have the potential to personalize treatment by objective assessment of these effects. This project is supported by the Biomarkers Consortium by Foundaiton of National Institutes of Health. All imaging, reports, and clinical information will be de-identified.

5.5.2 Collaborators

Evis Sala, MD, PhD, FRCR
Professor of Oncological Imaging
University of Cambridge
Department of Radiology
Box 218, Cambridge Biomedical Campus
Cambridge CB2 0QQ
United Kingdom
Phone: +44 1223 746440
Email: es220@cam.ac.uk

Professor James D. Brenton PhD FRCP
Senior Group Leader and Honorary Consultant in Medical Oncology
Functional Genomics of Ovarian Cancer Laboratory
Cancer Research UK Cambridge Institute
University of Cambridge
Li Ka Shing Centre
01223 769761 Assistant
mailto:james.brenton@cruk.cam.ac.uk
http://www.cruk.cam.ac.uk/research-groups/brenton-group

5.5.3 Procedure

- An independent review of all scans of ovarian cancer participants will be conducted by Professor Sala's lab in order to develop and validate precise and robust radiogenomics biomarkers to optimize response assessment.

- Copies of all imaging assessments including unscheduled visit scans will be collected on an ongoing basis and sent to the collaborators listed in Section **5.5.2**. The scans will be de-identified by a research nurse in collaboration with staff in the film library prior to being sent to the collaborators.
- Prior radiation therapy reports for participants (at baseline) and information on any lesions that were biopsied to provide a tumor sample for study entry will be provided to allow the selection of appropriate target lesions.
- Results of this scan review will not be communicated to Investigators to make a clinical decision.

5.6 PATIENT SAMPLE PROTECTIONS, STORAGE, TRACKING, DISPOSITION

5.6.1 Patient sample protections

Each patient sample set will be coded with a unique patient identifier. No patient specific information is encoded in this identifier. The protocol scientific investigators handling samples will be blinded as to as to the patient identification, patient data and outcome. Tissue samples obtained by and stored through Lee Lab will be labeled with unique barcodes.

Blood samples obtained by and stored through the Clinical Pharmacology Core and Trepel Lab will be labeled with unique barcodes.

The amount of blood that may be drawn from adult patients (i.e., those persons 18 years of age or older) for research purposes shall not exceed 10.5 mL/kg or 550 mL, whichever is smaller, over any eight week period.

If a patient needs to have a malignant effusion or ascites tapped for diagnostic or therapeutic purposes, a sample will be collected for research.

6 SAMPLE STORAGE, TRACKING AND DISPOSITION

Samples collected at NCI will be ordered in CRIS and tracked through a Clinical Trial Data Management system. Should a CRIS screen not be available, the CRIS downtime procedures will be followed. Samples will not be sent outside NIH without appropriate approvals and/or agreements, if required.

6.1 BLOOD PROCESSING CORE (BPC)

Sample Data Collection

All samples sent to the Blood Processing Core (BPC) will be barcoded, with data entered and stored in the Labmatrix utilized by the BPC. This is a secure program, with access to Labmatrix limited to defined Figg lab personnel, who are issued individual user accounts. Installation of Labmatrix is limited to computers specified by Dr. Figg. These computers all have a password restricted login screen.

Lamatrix creates a unique barcode ID for every sample and sample box, which cannot be traced back to patients without Labmatrix access. The data recorded for each sample includes the patient ID, name, trial name/protocol number, time drawn, cycle time point, dose, material type, as well as box and freezer location. Patient demographics associated with the clinical center

patient number are provided in the system. For each sample, there are notes associated with the processing method (delay in sample processing, storage conditions on the ward, etc.).

Sample Storage and Destruction

Barcoded samples are stored in barcoded boxes in a locked freezer at either -20 or -80°C according to stability requirements. These freezers are located onsite in the BPC and offsite at NCI Frederick Central Repository Services in Frederick, MD. Visitors to the laboratory are required to be accompanied by laboratory staff at all times.

Access to stored clinical samples is restricted. Samples will be stored until requested by a researcher named on the protocol. All requests are monitored and tracked in Labmatrix. All researchers are required to sign a form stating that the samples are only to be used for research purposes associated with this trial (as per the IRB approved protocol) and that any unused samples must be returned to the BPC. It is the responsibility of the NCI Principal Investigator to ensure that the samples requested are being used in a manner consistent with IRB approval.

Following completion of this study, samples will remain in storage as detailed above. Access to these samples will only be granted following IRB approval of an additional protocol, granting the rights to use the material.

If, at any time, a patient withdraws from the study and does not wish for their existing samples to be utilized, the individual must provide a written request. Any samples lost (in transit or by a researcher) or destroyed due to unknown sample integrity (i.e. broken freezer allows for extensive sample thawing, etc.) that meet expedited reporting requirements (see section **8.2.1**) will be reported to the IRB.

Sample barcodes are linked to patient demographics and limited clinical information. This information will only be provided to investigators listed on this protocol, via registered use of the Labmatrix. It is critical that the sample remains linked to patient information such as race, age, dates of diagnosis and death, and histological information about the tumor, in order to correlate genotype with these variables.

6.1.1 End of Study

All specimens obtained in the protocol are used as defined in the protocol. Any specimens that are remaining at the completion of the protocol will be stored in the conditions described below. The study will remain open so long as sample or data analysis continues. Samples from consenting subjects will be stored until they are no longer of scientific value or if a subject withdraws consent for their continued use, at which time they will be destroyed. The PI will record any loss or unanticipated destruction of samples as a deviation. Reporting will be per the requirments in section **8.2**. Any new use of identified or coded samples, specimens, or data will undergo prospective and continuing IRB review and approval.

If researchers have samples remaining once they have completed all studies associated with the protocol, they must be returned to the Lee laboratory or to the BPC repository. Samples can only be saved at the completion of the study for future use if subjects consented. Access to these samples will only be granted following IRB approval of an additional protocol, granting the rights to use the material.

If the patient withdraws consent, the participant's data will be excluded from future distributions,

but data that have already been distributed for approved research use will not be able to be retrieved.

7 DATA COLLECTION AND EVALUATION

7.1 DATA COLLECTION

The PI will be responsible for overseeing entry of data into an in-house password protected electronic system and ensuring data accuracy, consistency and timeliness. The principal investigator, associate investigators/research nurses and/or a contracted data manager will assist with the data management efforts. Primary and final analyzed data will have identifiers so that research data can be attributed to an individual human subject participant.

All adverse events, including clinically significant abnormal findings on laboratory evaluations, regardless of severity, will be followed until return to baseline or stabilization of event.

Document AEs from Study Day 1 through the end of the follow-up period (90 days after the last dose of Durvalumab or 30 days after the last dose of olaparib or cediranib, whichever is later, or until subsequent other cancer treatment following progression). Beyond this window, only adverse events which are serious and related to the study intervention need to be recorded.

An abnormal laboratory value will be recorded in the database as an AE **only** if the laboratory abnormality is characterized by any of the following:

- Results in discontinuation from the study
- Is associated with clinical signs or symptoms
- Requires treatment or any other therapeutic intervention
- Is associated with death or another serious adverse event, including hospitalization.
- Is judged by the Investigator to be of significant clinical impact
- If any abnormal laboratory result is considered clinically significant, the investigator will provide details about the action taken with respect to the test drug and about the patient's outcome.

End of study procedures: Data will be stored according to HHS, FDA regulations and NIH Intramural Records Retention Schedule as applicable.

Loss or destruction of data: Should we become aware that a major breach in our plan to protect subject confidentiality and trial data has occurred, this will be reported expeditiously per requirements in section 8.2.1.

7.2 SOURCE DOCUMENTS

Source documents are defined as original documents, data and records. This may include hospital records, clinical and office charts, laboratory data/information, patients' diaries or evaluation checklists, pharmacy dispensing and other records, recorded data from automated instruments, microfiches, photographic negatives, microfilm or magnetic media, X-rays. The

investigator will permit trial-related monitoring, audits, IRB review, and regulatory inspection(s), providing direct access to source documents.

7.3 Case Report Forms

Data may be entered from the source documents directly into eCRFs in C3D for each patient enrolled in this study. The principal investigator or research nurse will review the eCRFs for completeness and accuracy. Independent audits may also be conducted by NCI personnel to ensure completeness and accuracy of data in C3D.

7.4 Genomic Data Sharing Plan

Unlinked genomic data will be deposited in public genomic databases such as dbGaP in compliance with the NIH Genomic Data Sharing Policy. (**See http://bit.ly/CCR_GDShttp://bit.ly/CCR_GDS**)

7.5 Response Criteria

Response will be documented by physical exam and/or noninvasive imaging using techniques that demonstrate lesions and tumor markers where appropriate. Response and progression will be evaluated in this study using the international criteria proposed by the Response Evaluation Criteria in Solid Tumors (RECIST) Committee (Einsenhauer et al. 2009).

Changes in only the largest diameter (unidimensional measurement) of the tumor lesions are used in the RECIST version 1.1 criteria.

For the purposes of this study, patients should be re-evaluated for response every 8 weeks. In addition to a baseline scan, confirmatory scans should also be obtained at least 4 weeks following initial documentation of objective response.

Response and progression will be evaluated in this study using the new international criteria proposed by the revised Response Evaluation Criteria in Solid Tumors (RECIST) guideline (version 1.1) [*Eur J Ca* 45:228-247, 2009]. Changes in the largest diameter (unidimensional measurement) of the tumor lesions and the shortest diameter in the case of malignant lymph nodes are used in the RECIST criteria.

For Phase II mCRPC cohort, patients will be assessed primarily by CT scan of the chest, abdomen and pelvis and by technetium 99 bone scan at baseline, 8 weeks and then every 12 weeks.[167] Response and progression will be evaluated in this study using the new international criteria proposed by the revised Response Evaluation Criteria in Solid Tumors (RECIST) guideline (version 1.1)[168] and Prostate Cancer Clinical Trials Working Group criteria (PCWG2)[167]. Changes in the largest diameter (unidimensional measurement) of the tumor lesions and the shortest diameter in the case of malignant lymph nodes are used in the RECIST criteria.

Metastatic Bone Lesions

Disease progression is considered if a minimum of two new lesions is observed on bone scan. New lesions seen by the end of cycle 2 or before cycle 3 (after the first staging bone scan) may represent disease that was not detected on the pre-study scan, and a confirmatory scan will be required at the next scheduled staging bone scan. If confirmed, progression should be dated by the initial time when the lesions are first detected. If new lesions are seen after cycle 2 but no additional lesions are seen on confirmatory scans, the scans from after cycle 2 would serve as the baseline scan to evaluate for disease progression.[167]

Definitions

Evaluable for toxicity: All patients will be evaluable for toxicity from the time of their first treatment with Durvalumab, olaparib or cediranib (whichever is administered first).

Evaluable for objective response: Only those patients who have measurable disease present at baseline, have received at least one cycle of therapy, and have had their disease re-evaluated will be considered evaluable for response. These patients will have their response classified according to the definitions stated below. (Note: Patients who exhibit objective disease progression prior to the end of cycle 1 will also be considered evaluable.)

Evaluable Non-Target Disease Response: Patients who have lesions present at baseline that are evaluable but do not meet the definitions of measurable disease, have received at least one cycle of therapy, and have had their disease re-evaluated will be considered evaluable for non-target disease. The response assessment is based on the presence, absence, or unequivocal progression of the lesions.

7.5.1 Disease Parameters

Measurable disease: Measurable lesions are defined as those that can be accurately measured in at least one dimension (longest diameter to be recorded) as:

- By chest x-ray: ≥20 mm;
- By CT scan:
    - Scan slice thickness 5 mm or under as ≥10 mm with CT scan
    - Scan slice thickness >5 mm: double the slice thickness
- With calipers on clinical exam≥10 mm.

All tumor measurements must be recorded in millimeters (or decimal fractions of centimeters).

Malignant lymph nodes. To be considered pathologically enlarged and measurable, a lymph node must be ≥15 mm in short axis when assessed by CT scan (CT scan slice thickness recommended to be no greater than 5 mm). At baseline and in follow-up, only the short axis will be measured and followed.

Non-measurable disease. All other lesions (or sites of disease), including small lesions (longest diameter <10 mm or pathological lymph nodes with ≥10 to <15 mm short axis), are considered non-measurable disease. Bone lesions, leptomeningeal disease, ascites, pleural/pericardial

effusions, lymphangitis cutis/pulmonitis, inflammatory breast disease, and abdominal masses (not followed by CT or MRI), are considered as non-measurable.

Note: Cystic lesions that meet the criteria for radiographically defined simple cysts should not be considered as malignant lesions (neither measurable nor non-measurable) since they are, by definition, simple cysts.

'Cystic lesions' thought to represent cystic metastases can be considered as measurable lesions, if they meet the definition of measurability described above. However, if non-cystic lesions are present in the same patient, these are preferred for selection as target lesions.

Target lesions. All measurable lesions up to a maximum of 2 lesions per organ and 5 lesions in total, representative of all involved organs, should be identified as **target lesions** and recorded and measured at baseline. Target lesions should be selected on the basis of their size (lesions with the longest diameter), be representative of all involved organs, but in addition should be those that lend themselves to reproducible repeated measurements. It may be the case that, on occasion, the largest lesion does not lend itself to reproducible measurement in which circumstance the next largest lesion, which can be measured reproducibly, should be selected. A sum of the diameters (longest for non-nodal lesions, short axis for nodal lesions) for all target lesions will be calculated and reported as the baseline sum diameters. If lymph nodes are to be included in the sum, then only the short axis is added into the sum. The baseline sum diameters will be used as reference to further characterize any objective tumor regression in the measurable dimension of the disease.

Non-target lesions. All other lesions (or sites of disease) including any measurable lesions over and above the 5 target lesions should be identified as **non-target lesions** and should also be recorded at baseline. Measurements of these lesions are not required, but the presence, absence, or in rare cases unequivocal progression of each should be noted throughout follow-up.

Methods for Evaluation of Measurable Disease

All measurements should be taken and recorded in metric notation using a ruler or calipers. All baseline evaluations should be performed as closely as possible to the beginning of treatment and never more than 4 weeks before the beginning of the treatment.

The same method of assessment and the same technique should be used to characterize each identified and reported lesion at baseline and during follow-up. Imaging-based evaluation is preferred to evaluation by clinical examination unless the lesion(s) being followed cannot be imaged but are assessable by clinical exam.

- Clinical lesions: Clinical lesions will only be considered measurable when they are superficial (e.g., skin nodules and palpable lymph nodes) and ≥10 mm diameter as assessed using calipers (e.g., skin nodules). In the case of skin lesions, documentation by color photography, including a ruler to estimate the size of the lesion, is recommended.
- Chest x-ray: Lesions on chest x-ray are acceptable as measurable lesions when they are clearly defined and surrounded by aerated lung. However, CT is preferable.
- Conventional CT and MRI: This guideline has defined measurability of lesions on CT scan based on the assumption that CT slice thickness is 5 mm or less. If CT scans have slice

thickness greater than 5 mm, the minimum size for a measurable lesion should be twice the slice thickness. MRI is also acceptable in certain situations (e.g. for body scans).

Use of MRI remains a complex issue. MRI has excellent contrast, spatial, and temporal resolution; however, there are many image acquisition variables involved in MRI, which greatly impact image quality, lesion conspicuity, and measurement. Furthermore, the availability of MRI is variable globally. As with CT, if an MRI is performed, the technical specifications of the scanning sequences used should be optimized for the evaluation of the type and site of disease. Furthermore, the modality used at follow-up should be the same as was used at baseline and the lesions should be measured/assessed on the same pulse sequence. It is beyond the scope of RECIST guidelines to prescribe specific MRI pulse sequence parameters for all scanners, body parts, and diseases. Ideally, the same type of scanner should be used and the image acquisition protocol should be followed as closely as possible to prior scans. Body scans should be performed with breath-hold scanning techniques, if possible.

PET-CT: At present, the low dose or attenuation correction CT portion of a combined PET-CT is not always of optimal diagnostic CT quality for use with RECIST measurements. However, if the site can document that the CT performed as part of a PET-CT is of identical diagnostic quality to a diagnostic CT (with IV and oral contrast), then the CT portion of the PET-CT can be used for RECIST measurements and can be used interchangeably with conventional CT in accurately measuring cancer lesions over time. Note, however, that the PET portion of the CT introduces additional data which may bias an investigator if it is not routinely or serially performed.

Ultrasound: Ultrasound is not useful in assessment of lesion size and should not be used as a method of measurement. Ultrasound examinations cannot be reproduced in their entirety for independent review at a later date and, because they are operator dependent, it cannot be guaranteed that the same technique and measurements will be taken from one assessment to the next. If new lesions are identified by ultrasound in the course of the study, confirmation by CT or MRI is advised. If there is concern about radiation exposure at CT, MRI may be used instead of CT in selected instances.

Endoscopy, Laparoscopy: The utilization of these techniques for objective tumor evaluation is not advised. However, such techniques may be useful to confirm complete pathological response when biopsies are obtained or to determine relapse in trials where recurrence following complete response [29] or surgical resection is an endpoint.

Tumor markers: Tumor markers alone cannot be used to assess response. If markers are initially above the upper normal limit, they must normalize for a patient to be considered in complete clinical response. Specific guidelines for both CA-125 response (in recurrent ovarian cancer) and PSA response (in recurrent prostate cancer) have been published [*JNCI* 96:487-488, 2004; *J Clin Oncol* 17, 3461-3467, 1999; *J Clin Oncol* 26:1148-1159, 2008]. In addition, the Gynecologic Cancer Intergroup has developed CA-125 progression criteria which are to be integrated with objective tumor assessment for use in first-line trials in ovarian cancer [*JNCI* 92:1534-1535, 2000].

Cytology, Histology: These techniques can be used to differentiate between partial responses (PR) and complete responses [29] in rare cases (e.g., residual lesions in tumor types, such as germ cell tumors, where known residual benign tumors can remain).

The cytological confirmation of the neoplastic origin of any effusion that appears or worsens during treatment when the measurable tumor has met criteria for response or stable disease is mandatory to differentiate between response or stable disease (an effusion may be a side effect of the treatment) and progressive disease.

FDG-PET: While FDG-PET response assessments need additional study, it is sometimes reasonable to incorporate the use of FDG-PET scanning to complement CT scanning in assessment of progression (particularly possible 'new' disease). New lesions on the basis of FDG-PET imaging can be identified according to the following algorithm:

a. Negative FDG-PET at baseline, with a positive FDG-PET at follow-up is a sign of PD based on a new lesion.

b. No FDG-PET at baseline and a positive FDG-PET at follow-up: If the positive FDG-PET at follow-up corresponds to a new site of disease confirmed by CT, this is PD. If the positive FDG-PET at follow-up is not confirmed as a new site of disease on CT, additional follow-up CT scans are needed to determine if there is truly progression occurring at that site (if so, the date of PD will be the date of the initial abnormal FDG-PET scan). If the positive FDG-PET at follow-up corresponds to a pre-existing site of disease on CT that is not progressing on the basis of the anatomic images, this is not PD.

c. FDG-PET may be used to upgrade a response to a CR in a manner similar to a biopsy in cases where a residual radiographic abnormality is thought to represent fibrosis or scarring. The use of FDG-PET in this circumstance should be prospectively described in the protocol and supported by disease-specific medical literature for the indication. However, it must be acknowledged that both approaches may lead to false positive CR due to limitations of FDG-PET and biopsy resolution/sensitivity.

Note: A ‘positive’ FDG-PET scan lesion means one which is FDG avid with an uptake greater than twice that of the surrounding tissue on the attenuation corrected image.

Response Criteria

7.5.1.1 Evaluation of Target Lesions

- Complete Response [29]: Disappearance of all target lesions. Any pathological lymph nodes (whether target or non-target) must have reduction in short axis to <10 mm.
- Partial Response (PR): At least a 30% decrease in the sum of the diameters of target lesions, taking as reference the baseline sum of diameters.
- Progressive Disease (PD): At least a 20% increase in the sum of the diameters of target lesions, taking as reference the smallest sum on study (this includes the baseline sum if that is the smallest on study). In addition to the relative increase of 20%, the sum must also demonstrate an absolute increase of at least 5 mm. (Note: the appearance of one or more new lesions is also considered progressions).

- Stable Disease (SD): Neither sufficient shrinkage to qualify for PR nor sufficient increase to qualify for PD, taking as reference the smallest sum of diameters while on study.

7.5.1.2 Evaluation of Non-Target Lesions

- Complete Response [29]: Disappearance of all non-target lesions and normalization of tumor marker level. All lymph nodes must be non-pathological in size (<10 mm short axis).
- Note: If tumor markers are initially above the upper normal limit, they must normalize for a patient to be considered in complete clinical response.
- Non-CR/Non-PD: Persistence of one or more non-target lesion(s) and/or maintenance of tumor marker level above the normal limits.
- Progressive Disease (PD): Appearance of one or more new lesions and/or *unequivocal progression* of existing non-target lesions. *Unequivocal progression* should not normally trump target lesion status. It must be representative of overall disease status change, not a single lesion increase.
- Although a clear progression of "non-target" lesions only is exceptional, the opinion of the treating physician should prevail in such circumstances, and the progression status should be confirmed at a later time by the review panel (or the PI).

7.5.1.3 Evaluation of Best Overall Response

- The best overall response is the best response recorded from the start of the treatment until disease progression/recurrence (taking as reference for progressive disease the smallest measurements recorded since the treatment started).
- The patient's best response assignment will depend on the achievement of both measurement and confirmation criteria.

Table 29. Evaluation of Best Overall Response for Patients with Measurable Disease (i.e., Target Disease)

<table>
<tr><th>Target Lesions</th><th>Non-Target Lesions</th><th>New Lesions</th><th>Overall Response</th><th>Best Overall Response when Confirmation is Required*</th></tr>
<tr><td>CR</td><td>CR</td><td>No</td><td>CR</td><td>≥4 wks. Confirmation**</td></tr>
<tr><td>CR</td><td>Non-CR/Non-PD</td><td>No</td><td>PR</td><td rowspan="3">≥4 wks. Confirmation**</td></tr>
<tr><td>CR</td><td>Not evaluated</td><td>No</td><td>PR</td></tr>
<tr><td>PR</td><td>Non-CR/Non-PD/not evaluated</td><td>No</td><td>PR</td></tr>
<tr><td>SD</td><td>Non-CR/Non-PD/not evaluated</td><td>No</td><td>SD</td><td>Documented at least once ≥4 wks. from baseline**</td></tr>
</table>

PD	Any	Yes or No	PD	
Any	PD***	Yes or No	PD	no prior SD, PR or CR
Any	Any	Yes	PD	

*	See RECIST 1.1 manuscript for further details on what is evidence of a new lesion.
**	Only for non-randomized trials with response as primary endpoint.
***	In exceptional circumstances, unequivocal progression in non-target lesions may be accepted as disease progression.
Note:	Patients with a global deterioration of health status requiring discontinuation of treatment without objective evidence of disease progression at that time should be reported as "*symptomatic deterioration.*" Every effort should be made to document the objective progression even after discontinuation of treatment.

Table 30. Evaluation of Best Overall Response for Patients with Non-Measurable Disease (i.e., Non-Target Disease)

Non-Target Lesions	New Lesions	Overall Response
CR	No	CR
Non-CR/non-PD	No	Non-CR/non-PD*
Not all evaluated	No	not evaluated
Unequivocal PD	Yes or No	PD
Any	Yes	PD

* 'Non-CR/non-PD' is preferred over 'stable disease' for non-target disease since SD is increasingly used as an endpoint for assessment of efficacy in some trials so to assign this category when no lesions can be measured is not advised

6.4.4.4 Evaluation of pseudoprogression (Immune-related RECIST [irRECIST])

Modification of RECIST as described below may discourage the early discontinuation of Durvalumab and provide a more complete evaluation of its anti-tumor activity than would be seen with conventional response criteria. Nonetheless, the efficacy analysis will be conducted by programmatically deriving each efficacy endpoint based on RECIST 1.1 criteria and irRECIST is the exploratory endpoint.

The response to immunotherapy may differ from the typical responses observed with cytotoxic chemotherapy including the following (Wolchok et al 2009):

- Response to immunotherapy may be delayed
- Response to immunotherapy may occur after PD by conventional RECIST criteria
- The appearance of new lesions may not represent PD with immunotherapy

- SD while on immunotherapy may be durable and represent clinical benefit.

Based on the above-described unique response to immunotherapy and based on guidelines from regulatory agencies, e.g., European Medicines Agency's "Guideline on the evaluation of anti-cancer medicinal products in man" (EMA/CHMP/205/95/Rev.4) for immune modulating anti-cancer compounds, the following may be implemented in addition to standard RECIST 1.1 criteria as one of exploratory endpoints:

- RECIST will be modified so that PD must be confirmed at the next scheduled visit, preferably, and no earlier than 4 weeks after the initial assessment of PD **in the absence of clinically significant deterioration**. Treatment with Durvalumab would continue between the initial assessment of progression and confirmation for progression.

- In addition, subjects may continue to receive Durvalumab beyond confirmed PD **in the absence of clinically significant deterioration** and if investigators consider that subjects continue to receive benefit from treatment. Of note, clinically significant deterioration is considered to be a rapid tumor progression that necessitates treatment with anti-cancer therapy other than Durvalumab or with symptomatic progression that requires urgent medical intervention (e.g., central nervous system metastasis, respiratory failure due to tumor compression, spinal cord compression).

<u>Duration of Response</u>

- <u>Duration of overall response</u>: The duration of overall response is measured from the time measurement criteria are met for CR or PR (whichever is first recorded) until the first date that recurrent or progressive disease is objectively documented (taking as reference for progressive disease the smallest measurements recorded since the treatment started).
- The duration of overall CR is measured from the time measurement criteria are first met for CR until the first date that progressive disease is objectively documented.
- <u>Duration of stable disease</u>: Stable disease is measured from the start of the treatment until the criteria for progression are met, taking as reference the smallest measurements recorded since the treatment started, including the baseline measurements.

<u>Progression-Free Survival</u>

PFS is defined as the duration of time from start of treatment to time of progression or death, whichever occurs first.

<u>Confirmation of Response and Response Review</u>

The main goal of confirmation of objective response is to avoid overestimating the response rate observed. In cases where confirmation of response is not feasible, it should be made clear when reporting the outcome of such studies that the responses are not confirmed.

To be assigned a status of PR or CR, changes in tumor measurements must be confirmed by repeat assessments that should be performed no less than 4 weeks after the criteria for response are first met. Longer intervals as determined by the study protocol may also be appropriate. In the case of SD, follow-up measurements must have met the SD criteria at least once after study entry at a minimum interval, not less than 6 weeks.

<u>Reporting of Results</u>

Only patients who received one full cycle of treatment and are re-evaluated will be assessed for response to treatment, even if there are major protocol treatment deviations or if they are ineligible. Each patient will be assigned one of the following categories: 1) complete response, 2) partial response, 3) stable disease, 4) progressive disease, 5) early death from malignant disease, 6) early death from toxicity, 7) early death because of other cause, or 9) unknown (not assessable, insufficient data).

Patients in response categories 4-8 should be considered as failing to respond to treatment (disease progression). Thus, an incorrect treatment schedule or drug administration does not result in exclusion from the analysis of the response rate. Precise definitions for categories 4-8 will be protocol specific.

All conclusions should be based on all enrolled patients.

Sub-analyses may then be performed on the basis of a subset of patients, excluding those for

whom major protocol deviations have been identified. However, these sub-analyses may not serve as the basis for drawing conclusions concerning treatment efficacy, and the reasons for excluding patients from the analysis should be clearly reported.

The 95% confidence intervals should be provided.

7.6 Toxicity Criteria

The following adverse event management guidelines are intended to ensure the safety of each patient while on the study. The descriptions and grading scales found in the revised NCI Common Terminology Criteria for Adverse Events (CTCAE) version 4.0 will be utilized for AE reporting. All appropriate treatment areas should have access to a copy of the CTCAE version 4.0. A copy of the CTCAE version 4.0 can be downloaded from the CTEP web site (http://ctep.cancer.gov/protocolDevelopment/electronic_applications/ctc.htm#ctc_40).

8 NIH REPORTING REQUIREMENTS/DATA AND SAFETY MONITORING PLAN

8.1 Definitions

Please refer to definitions provided in Policy 801: Reporting Research Events found **here**.

8.2 OHSRP Office of Compliance and Training / IRB Reporting

8.2.1 Expedited Reporting

Please refer to the reporting requirements in Policy 801: Reporting Research Events and Policy 802 Non-Compliance Human Subjects Research found **here**. Note: Only IND Safety Reports that meet the definition of an unanticipated problem will need to be reported per these policies.

8.2.2 IRB Requirements for PI Reporting at Continuing Review

Please refer to the reporting requirements in Policy 801: Reporting Research Events found **here**.

8.3 NCI Clinical Director Reporting

Problems expeditiously reported to the OHSRP in iRIS will also be reported to the NCI Clinical Director. A separate submission is not necessary as reports in iRIS will be available to the Clinical Director.

In addition to those reports, all deaths that occur within 30 days after receiving a research intervention should be reported via email to the Clinical Director unless they are due to progressive disease.

To report these deaths, please send an email describing the circumstances of the death to Dr. Dahut at NCICCRQA@mail.nih.gov within one business day of learning of the death.

8.4 NIH Required Data and Safety Monitoring Plan

8.4.1 Principal Investigator/Research Team

The clinical research team will meet on a regular basis when patients are being actively treated on the trial to discuss each patient. Decisions about dose level enrollment and dose escalation if applicable will be made based on the toxicity data from prior patients.

All data will be collected in a timely manner and reviewed by the PI or a lead associate investigator. Events meeting requirements for expedited reporting as described in Section **8.2.1** will be submitted within the appropriate timelines. See Section **7.1**for documentation requirements.

The PI will review adverse event and response data on each patient to ensure safety and data accuracy. The PI will personally conduct or supervise the investigation and provide appropriate delegation of responsibilities to other members of the research staff.

9 SPONSOR SAFETY REPORTING

9.1 Definitions

9.1.1 Adverse Event

Any untoward medical occurrence in a patient or clinical investigation subject administered a pharmaceutical product and which does not necessarily have a causal relationship with this treatment. An adverse event (AE) can therefore be any unfavorable and unintended sign (including an abnormal laboratory finding), symptom, or disease temporally associated with the use of a medicinal (investigational) product, whether or not related to the medicinal (investigational) product (ICH E6 (R2))

9.1.2 Serious Adverse Event (SAE)

An adverse event or suspected adverse reaction is considered serious if in the view of the investigator or the sponsor, it results in any of the following:

- Death,
- A life-threatening adverse event (see **9.1.3**)
- Inpatient hospitalization or prolongation of existing hospitalization

  - A hospitalization/admission that is pre-planned (i.e., elective or scheduled surgery arranged prior to the start of the study), a planned hospitalization for pre-existing condition, or a procedure required by the protocol, without a serious deterioration in health, is not considered a serious adverse event.
  - A hospitalization/admission that is solely driven by non-medical reasons (e.g., hospitalization for patient or subject convenience) is not considered a serious adverse event.
  - Emergency room visits or stays in observation units that do not result in admission to the hospital would not be considered a serious adverse event. The reason for seeking medical care should be evaluated for meeting one of the other serious criteria.
- Persistent or significant incapacity or substantial disruption of the ability to conduct normal life functions
- A congenital anomaly/birth defect.
- Important medical events that may not result in death, be life-threatening, or require hospitalization may be considered a serious adverse drug experience when, based upon appropriate medical judgment, they may jeopardize the patient or subject and may require medical or surgical intervention to prevent one of the outcomes listed in this definition.

9.1.3 Life-threatening

An adverse event or suspected adverse reaction is considered "life-threatening" if, in the view of either the investigator or sponsor, its occurrence places the patient or subject at immediate risk of death. It does not include an adverse event or suspected adverse reaction that, had it occurred in a more severe form, might have caused death. (21CFR312.32)

9.1.4 Severity

The severity of each Adverse Event will be assessed utilizing the CTCAE version 4.0.

9.1.5 Relationship to Study Product

All AEs will have their relationship to study product assessed using the terms: related or not related.

- Related – There is a reasonable possibility that the study product caused the adverse event. Reasonable possibility means that there is evidence to suggest a causal relationship between the study product and the adverse event.

- Not Related – There is not a reasonable possibility that the administration of the study product caused the event.

9.1.6 Adverse Events of Special Interest (AESI)

An AESI is one of scientific and medical interest specific to understanding of the investigational product and may require close monitoring and rapid communication by the investigator to AstraZeneca/Medimmune. An AESI may be serious or nonserious. The rapid reporting of AESIs allows ongoing analysis of these events in order to characterize and understand them in association with the use of this investigational product.

AESIs for Durvalumab include but are not limited to events with a potential inflammatory or immune-mediated mechanism and which may require more frequent monitoring and/or interventions such as steroids, immunosuppressants and/or hormone replacement therapy. These AESIs are being closely monitored in clinical studies with Durvalumab monotherapy and combination therapy. An immune-related adverse event (irAE) is defined as an adverse event that is associated with drug exposure and is consistent with an immune-mediated mechanism of action and where there is no clear alternate etiology. Serologic, immunologic, and histologic (biopsy) data, as appropriate, should be used to support an irAE diagnosis. Appropriate efforts should be made to rule out neoplastic, infectious, metabolic, toxin, or other etiologic causes of the irAE. See the Durvalumab investigator brochure for further information.

AESIs observed with Durvalumab include:

- Diarrhea/Colitis
- Pericarditis
- Myocarditis
- Myositis/Polymyositis
- Pneumonitis
- Sarcoidosis
- Vasculitis
- ALT/AST increases / hepatitis / hepatotoxicity
- Neuropathy / neuromuscular toxicity (i.e. events of meningitis, encephalitis, peripheral motor and sensory neuropathies, Guillain-Barré, and myasthenia gravis)
- Endocrinopathy (i.e. events of hypophysitis, adrenal insufficiency, and hyper- and hypothyroidism)
- Dermatitis and other events involving the skin (ex, scleroderma, vitiligo and pemphigoid)
- Nephritis
- Pancreatitis (or labs suggestive of pancreatitis - increased serum lipase , increased serum amylase)
- Uveitis and other events involving the eye (ex, keratitis and optic neuritis)
- Hematological (ex, hemolytic anemia and immune thrombocytopenic purpura)
- Rheumatological events (polymyalgia rheumatic and autoimmune arthritis)

<u>Pneumonitis</u>

- Adverse events of pneumonitis are of interest for AstraZeneca/Medimmune, as pneumonitis has been reported with anti-PD-1 MAbs (Topalian et al, NEJM 2012). Initial work-up should include high-resolution CT scan, ruling out infection, and pulse oximetry. Pulmonary consultation is highly recommended.

- Guidelines for the management of subjects with immune-mediated events including pneumonitis are outlined in Section **3.4.4.2.2**.

Hypersensitivity reactions

- Hypersensitivity reactions as well as infusion-related reactions have been reported with anti-PD-L1 and anti-PD-1 therapy (Topalian et al, 2012; Brahmer et al, 2012). As with the administration of any foreign protein and/or other biologic agents, reactions following the infusion of MAbs can be caused by various mechanisms, including acute anaphylactic (immunoglobulin E-mediated) and anaphylactoid reactions against the MAb, and serum sickness. Acute allergic reactions may occur, may be severe, and may result in death. Acute allergic reactions may include hypotension, dyspnea, cyanosis, respiratory failure, urticaria, pruritus, angioedema, hypotonia, arthralgia, bronchospasm, wheeze, cough, dizziness, fatigue, headache, hypertension, myalgia, vomiting and unresponsiveness.
- Guidelines for management of subjects with hypersensitivity (including anaphylactic reaction) and infusion-related reactions are outlined in Section **3.4.4.2.1**.

Hepatic function abnormalities (hepatotoxicity)

- Increased transaminases have been reported during treatment with anti-PD-L1/anti-PD-1 antibodies (Brahmer et al 2012). Inflammatory hepatitis has been reported in 3 % to 9 % of subjects treated with anti-CTLA-4 monoclonal antibodies (e.g., ipilimumab). The clinical manifestations of ipilimumab-treated subjects included general weakness, fatigue, nausea and/or mild fever and increased liver function tests such as AST, ALT, alkaline phosphatase, and/or total bilirubin.
- Hepatic function abnormality is defined as any increase in ALT or AST to greater than 3 × ULN and concurrent increase in total bilirubin to be greater than 2 × ULN. Concurrent findings are those that derive from a single blood draw or from separate blood draws taken within 8 days of each other. Follow-up investigations and inquiries will be initiated promptly to determine whether the findings are reproducible and/or whether there is objective evidence that clearly supports causation by a disease (e.g., cholelithiasis and bile duct obstruction with distended gallbladder) or an agent other than the investigational product. Guidelines for management of subjects with hepatic function abnormality are outlined in Section **3.4.4.2.2.**

9.1.7 Other events requiring reporting

9.1.7.1 Overdose

An overdose is defined as a subject receiving a dose of Durvalumab in excess of that specified in the Investigator's Brochure, unless otherwise specified in this protocol.

Any overdose of a study subject with Durvalumab, with or without associated AEs/SAEs, is required to be reported within 24 hours of knowledge of the event to the sponsor and AstraZeneca/MedImmune Patient Safety or designee using the designated Safety e-mailbox (see Section **9.3** for contact information). If the overdose results in an AE, the AE must also be

recorded as an AE. Overdose does not automatically make an AE serious, but if the consequences of the overdose are serious, for example death or hospitalization, the event is serious and must be recorded and reported as an SAE. There is currently no specific treatment in the event of an overdose of Durvalumab.

9.1.7.2 Hepatic function abnormality

Hepatic function abnormality (**Table 20**) in a study subject, with or without associated clinical manifestations, is required to be reported as "hepatic function abnormal" ***within 24 hours of knowledge of the event*** to the sponsor and AstraZeneca/MedImmune Patient Safety using the designated Safety e-mailbox (see Section **9.3** for contact information), unless a definitive underlying diagnosis for the abnormality (e.g., cholelithiasis or bile duct obstruction) that is unrelated to investigational product has been confirmed.

- If the definitive underlying diagnosis for the abnormality has been established and is unrelated to investigational product, the decision to continue dosing of the study subject will be based on the clinical judgment of the investigator.
- If no definitive underlying diagnosis for the abnormality is established, dosing of the study subject must be interrupted immediately. Follow-up investigations and inquiries must be initiated by the investigational site without delay.
- Each reported event of hepatic function abnormality will be followed by the investigator and evaluated by the sponsor and AstraZeneca/MedImmune.

9.2 Assessment of Safety Events

AE information collected will include event description, date of onset, assessment of severity and relationship to study product and alternate etiology (if not related to study product), date of resolution of the event, seriousness and outcome. The assessment of severity and relationship to the study product will be done only by those with the training and authority to make a diagnosis and listed on the Form FDA 1572 as the site principal investigator or sub-investigator. AEs occurring during the collection and reporting period will be documented appropriately regardless of relationship. AEs will be followed through resolution.

SAEs will be:

- Assessed for severity and relationship to study product and alternate etiology (if not related to study product) by a licensed study physician listed on the Form FDA 1572 as the site principal investigator or sub-investigator.
- Recorded on the appropriate SAE report form, the medical record and captured in the clinical database.
- Followed through resolution by a licensed study physician listed on the Form FDA 1572 as the site principal investigator or sub-investigator.

For timeframe of recording adverse events, please refer to section **7.1**. All serious adverse events recorded from the time of first investigational product administration must be reported to the sponsor.

9.3 Reporting of Serious Adverse Events

Any AE that meets a protocol-defined serious criteria, or meets the definition of Adverse Event of Special Interest that require expedited reporting must be submitted immediately (within 24 hours of awareness) to OSRO Safety using the CCR SAE report form.

All SAE reporting must include the elements described in **9.2**.

SAE reports will be submitted to the Center for Cancer Research (CCR) at: OSROSafety@mail.nih.gov and to the CCR PI and study coordinator. CCR SAE report form and instructions can be found at:
https://ccrod.cancer.gov/confluence/display/CCRCRO/Forms+and+Instructions

Following the assessment of the SAE by OSRO, other supporting documentation of the event may be requested by the OSRO Safety and should be provided as soon as possible.

9.4 Safety Reporting Criteria to the Pharmaceutical Collaborators

All events listed below must be reported in the defined timelines to CCRsafety@mail.nih.gov.

The CCR Office of Regulatory Affairs will send all reports to the manufacturer, as described below, for SAEs, SUSARs, AESIs, overdoses, and pregnancies.

If any pregnancy occurs in the course of the study, then the investigator should inform the appropriate AstraZeneca representatives within 1 day, ie, immediately, but **no later than 24 hours** of when he or she becomes aware of it.

The Investigator will notify AstraZeneca/MedImmune via email to: AEMailboxClinicalTrialTCS@astrazeneca.com in a written IND. Safety Report (MedWatch, Form 3500A) as soon as possible and no later than 3 business days for death or life threatening or 7 business days for all SAEs for which serious criteria apply. To the extent possible, any AE whose severity and attribution require expedited submission of the AE report to the FDA will be sent to AstraZeneca/MedImmune and company in advance of report submission to the FDA.

The Investigative site must also indicate, either in the SAE report or the fax cover page, the causality of events in relation to each of the study medications and if the SAE is related to disease progression, as determined by the principal investigator. Any event or hospitalization that is unequivocally due to progression of disease, as determined by the PI, will not be reported as an SAE to the FDA, however should be communicated to AstraZeneca/MedImmune.

A cover page should accompany the MedWatch form indicating the following:

- Durvalumab, olaparib, and cediranib Investigator Sponsored Study (ISS)
- The investigator IND number assigned by the FDA
- The investigator's name and address
- The trial name and AstraZeneca/MedImmune Reference number (ESR-14-10366)
- Causality

Follow-up information on SAEs must also be reported by the investigator to NCI and then

AstraZeneca/MedImmune within 3 business days for death or life threatening or 7 business days for all SAEs for which serious criteria apply after initial receipt of the information.

If follow-up indicates a change in the SAE from serious to fatal or life threatening, this information also needs to be communicated to NCI and AstraZeneca/MedImmune within 2 business days. If follow-up indicates a change from serious to non-serious, this information needs to be communicated to NCI and AstraZeneca/MedImmune within 7 business days.

All SAEs will be reported to AstraZeneca/MedImmune on a MedWatch form using the following guidelines:

- if there is more than one SAE listed on the MedWatch form, each should be bulleted or numbered
- causality for each SAE must be specified in the narrative section
- the date the site notified NCI and AstraZeneca/MedImmune of the SAE must be specified in the narrative section
- initial and follow-up MedWatch reports should be numbered either in the narrative section or in section G.7 of the MedWatch form

Once a MedWatch form is completed, it will be sent to AstraZeneca/MedImmune via email at: AEMailboxClinicalTrialTCS@astrazeneca.com

9.5 Reporting Pregnancy

All required pregnancy reports/follow-up to OSRO will be submitted to: OSROSafety@mail.nih.gov and to the CCR PI and study coordinator. Forms and instructions can be found here: https://ccrod.cancer.gov/confluence/display/CCRCRO/Forms+and+Instructions

9.5.1 Maternal exposure

If a patient becomes pregnant during the course of the study, the study treatment should be discontinued immediately, and the pregnancy reported to the Sponsor no later than 24 hours of when the Investigator becomes aware of it. The Investigator should notify the Sponsor no later than 24 hours of when the outcome of the pregnancy become known,

Pregnancy itself is not regarded as an SAE. However, congenital abnormalities or birth defects and spontaneous miscarriages that meet serious criteria (**9.1.2**) should be reported as SAEs.

The outcome of all pregnancies (spontaneous miscarriage, elective termination, ectopic pregnancy, normal birth, or congenital abnormality) should be followed up and documented.

9.5.2 Paternal exposure

Male patients should refrain from fathering a child or donating sperm during the study and for 3 months after the last dose of Durvalumab, cediranib, and/or olaparib.

Pregnancy of the patient’s partner is not considered to be an AE. However, the outcome of all pregnancies (spontaneous miscarriage, elective termination, ectopic pregnancy, normal birth, or congenital abnormality) occurring from the date of the first dose until 3 months after the last dose should, if possible, be followed up and documented.

9.6 REGULATORY REPORTING FOR STUDIES CONDUCTED UNDER CCR-SPONSORED IND

Following notification from the investigator, CCR, the IND sponsor, will report any suspected adverse reaction that is both serious and unexpected. CCR will report an AE as a suspected adverse reaction only if there is evidence to suggest a causal relationship between the study product and the adverse event. CCR will notify FDA and all participating investigators (i.e., all investigators to whom the sponsor is providing drug under its INDs or under any investigator's IND) in an IND safety report of potential serious risks from clinical trials or any other source, as soon as possible, in accordance to 21 CFR Part 312.32.

All serious events will be reported to the FDA at least annually in a summary format.

10 CLINICAL MONITORING PLAN

As a sponsor for clinical trials, FDA regulations require the CCR to maintain a monitoring program. The CCR's program allows for confirmation of: study data, specifically data that could affect the interpretation of primary and secondary study endpoints; adherence to the protocol, regulations, ICH E6, and SOPs; and human subjects protection. This is done through independent verification of study data with source documentation focusing on:

- Informed consent process
- Eligibility confirmation
- Drug administration and accountability
- Adverse events monitoring
- Response assessment.

The monitoring program also extends to multi-site research when the CCR is the coordinating center.

This trial will be monitored by personnel employed by a CCR contractor. Monitors are qualified by training and experience to monitor the progress of clinical trials. Personnel monitoring this study will not be affiliated in any way with the trial conduct.

11 STATISTICAL CONSIDERATIONS

11.1 PHASE I AND PHASE II OVARIAN CANCER COHORT 1 (DURVALUMAB+O AND DURVALUMAB+C)

The primary objectives of this study are to determine the recommended phase 2 dose (RP2D) of Durvalumab/olaparib (Durvalumab+O), Durvalumab/cediranib (Durvalumab+C) and Durvalumab/olaparib/cediranib (Durvalumab+O+C) in advanced tumors and to determine the preliminary clinical activity in recurrent ovarian cancer and other solid tumors. Secondary objectives are to determine the safety and toxicity, progression free survival (PFS), response rate, and the potential relationship between pretreatment tumor PD-L1 expression obtained from biopsies and clinical response in all patients enrolled in the phase II portion of the trial.

The trial begins as a phase I study with separate dose escalations planned to evaluate safety in patients receiving Durvalumab+O, Durvalumab+C and Durvalumab+O+C. For all phase I arms, using a standard 3+3 design, with 3 dose levels maximally explored in each arm (for example, patients could start on dose level 1, go to dose level -1 and then to dose level -2 or patients could

start on dose level 1, go to dose level 2 and then to dose level 3), a maximum of 18 patients for Durvalumab+O, a maximum of 36 for both Durvalumab+C daily schedule and Durvalumab+C intermittent schedule arms, and a maximum of 12 patients for Durvalumab+O+C would be required to be accrued in the phase I portion of the trial. An additional 3 slots will be allowed to account for inevaluable patients. Therefore, up to 69 total patients may be required for phase I altogether.

Following completion of the phase I portion of the trial for each arm, the phase II portion will be undertaken. It is expected that the overall fraction of patients who are likely to respond to single agent treatment would be approximately 15% based on the relevant literature. If a large majority of patients have platinum-resistant recurrent ovarian cancer, poorer prognosis would be expected. Following assignment to treatment, patients in each arm will be treated accordingly and their response determined. In order to ensure that the trial does not continue to enroll patients to either arm in the event that the response rates are substantially below a reasonable level, each arm will be conducted using a two-stage Simon optimal design[169] as follows.

Randomization procedures do not apply to the Phase I and Phase II portions of the study. The arms will be open concurrently and treatment assignment for each patient will be at the PI's discretion and/or based on prior treatment history. In each arm, the study will be conducted using an optimal two-stage phase II trial design, in order to rule out an unacceptably low 10% response rate (p0=0.10) in favor of a greatly improved overall response rate of 30% (p1=0.30). The trial will set alpha=0.10 (probability of accepting a poor treatment=0.10) and beta = 0.10 (probability of rejecting a good treatment=0.10).

For each of the Durvalumab+O or the Durvalumab+C arms, the first stage will initially enroll 12 evaluable patients to the appropriate arm, and if 0 to 1 of the 12 have a response, then no further patients will be accrued to that arm. If only one arm has 2 or more responses in the first 12 patients, only that arm will remain open for accrual of the remaining patients. If 2 or more of the first 12 patients have response, then accrual would continue on one or both arms until a total of 35 patients have enrolled on the appropriate arms. If there are 2 to 5 responses in 35 patients on an arm, this would be an uninterestingly low response rate. If there were 6 or more responses in 35 patients on an arm (17.1%), this may be sufficiently interesting to warrant further study in later trials. Under the null hypothesis (10% response rate), the probability of early termination of either arm is 65.9%.

PFS will also be estimated and compared between the two arms as a secondary endpoint, using Kaplan-Meier curves with a two-tailed log-rank test. The analyses will be considered exploratory and descriptive since the study does not have sufficient power to detect a large difference between two curves. This analysis will be done for all patients as well as within subsets of patients as determined by the adequacy of patient numbers in subsets. Response rates and safety and toxicity will also be evaluated in gBRCAm-based and platinum sensitivity-based subsets in the phase II portion of the trial if sufficient patients are available in appropriate categories.

If a death attributed to study treatment occurs on either arm during either the phase I or the phase II portion of the trial, accrual to that treatment arm will stop at that point, but accrual will continue to the other arm. In addition, during the phase II portion of the study, patients will be monitored for development of severe toxicity. If the cumulative fraction of patients with a DLT equals or exceeds 1/3 at any point on a given arm during phase II, no further patients will be

enrolled onto that arm as soon as this has been identified; patients will still be permitted to accrue to the other arm.

If accrual continues on one or both arms of the phase II portion of the trial until a total of 35 patients have enrolled on the appropriate arms, pre-treatment PD-L1 expression levels will have been obtained from all the phase II patients if possible. If there are 17 evaluable patients who respond and 17 who don't respond, then there would be 80% power to compare the pre-treatment PD-L1 levels between responders and non-responders with a 1.0 SD effect size, using a two-sided two-group t-test using a 0.05 alpha level. If there were 9 responders and 25 non-responders, there would be 70% power for the same calculation, or 80% or greater power to detect a larger effect size. The analyses will be done within each arm as well as for both arms together, with appropriate explanation of the results. For the other translational endpoints, each endpoint data analysis will provide 80% power to compare the changes between the two arms, from pre- to post-cycle 1 for a single endpoint, with a 1.0 SD effect size, using a two-sided two-group t-test using a 0.05 alpha level. These other translational studies are all being done with exploratory intent; thus, there is no formal correction for the multiple tests being evaluated, but that the results should be presented in the context of the number of tests performed.

11.2 PHASE II TRIPLE NEGATIVE BREAST CANCER (TNBC) COHORT 5 (DURVALUMAB+O ONLY)

The primary objective of this trial is to determine the adequacy of the clinical response rate in patients with triple negative breast cancer (TNBC) who receive treatment at the RP2D of Durvalumab+O.

In similar patients with TNBC who ordinarily would receive combination treatment, the clinical response rate may be estimated to be approximately 30-35% [121]. Preliminary, limited data suggest that patients with gBRCAm may have a response rate as high as 60-65%, while TNBC patients with BRCAwt, many of whom have been previously treated, may have response rates of approximately 15-20% overall. Because of the potential disparity in the response rates for these two subsets of patients with TNBC, each cohort will be enrolled separately using a design appropriate to each cohort.

For the TNBC patients with BRCAwt, estimated to be 65-80% of patients who may enroll, the goal would be to determine if using Durvalumab+O would rule out a 10% response rate and target a rate of 30%. For these patients, the trial will be conducted using a Simon Minimax two-stage phase II trial design[170] in order to rule out an unacceptably low PR+CR rate of 10% (p0=0.10) in favor of an improved response rate of 30% (p1=0.30). With alpha=0.10 (probability of accepting a poor treatment=0.10) and beta = 0.10 (probability of rejecting a good treatment=0.10), this first stage will enroll 16 evaluable patients, and if 0 to 1 of the 16 have a clinical response, then no further patients will be accrued. If 2 or more of the first 16 patients have a response, then accrual would continue until a total of 25 evaluable patients have been enrolled. As it may take several months to determine if a patient has experienced a response, a temporary pause in the accrual may be necessary to ensure that enrollment to the second stage is warranted. If there are 2 to 4 patients with a response out of 25 patients, this would be an uninterestingly low response rate. If there were 5 or more of 25 (20%) who experienced a

response, this would be sufficiently interesting to warrant further study in later trials. Under the null hypothesis (10% response rate), the probability of early termination is 51%.

For the TNBC patients with gBRCAm, estimated to be 20-35% of all patients who may enroll, the limited number of patients who may enroll during the time that the BRCAwt patients are accruing suggests that a small single stage design be used for this cohort of patients. There is only preliminary, limited data on the clinical response rate for these patients, so the objective will be to form an approximate estimate of their response rate to Durvalumab+O. A single cohort of 10 patients will be enrolled who have gBRCAm-related TNBC, and the response rate for them will be estimated separately from that of BRCAwt patients on this trial. There will be an early stopping rule implemented in this cohort: in view of the anticipated modestly high response rate for these patients, if 0 clinical responses are identified in the first 5 patients with gBRCAm then no further gBRCAm patients will be enrolled since the upper one-tailed 90% CI about 0/5 is 37%, which is substantially below the anticipated response rate for these patients and would justify enrolling no further patients once this can be determined.

The secondary objectives will each be evaluated using standard statistical methods, which will largely consist of reporting descriptive statistics and associated 95% confidence intervals. Time to event endpoints such as duration of response, progression free survival, and overall survival, will be determined using a Kaplan-Meier curve. Subset analyses of patients may be performed in a descriptive fashion after accrual has been completed. Any statistical testing performed will have results presented without formal adjustment for multiple comparisons.

11.3 PHASE II NON-SMALL CELL LUNG CANCER (NSCLC) COHORT 2 (DURVALUMAB+O AND DURVALUMAB+C)

NSCLC patients will be enrolled in both Durvalumab+C and Durvalumab+O arms. Randomization procedures do not apply to the Phase II NSCLC cohort. The arms will be open concurrently and treatment assignment for each patient will be at the PI's discretion and/or based on prior treatment history. The primary objective is to determine whether the combination of Durvalumab+C or Durvalumab+O is able to be associated with improved PFS in patients with NSCLC compared to that associated with an immune checkpoint inhibitor.

Data from a published trial of nivolumab indicated that patients with NSCLC experience a median PFS of 3.5 months, and another study determined a median of 3.7 months for those treated with pembrolizumab. The goal is to determine if use of Durvalumab+O or Durvalumab+C in NSCLC is able to be associated with an improved PFS from 3.5 months to 6.0 months. With 37 evaluable patients receiving Durvalumab+O and 37 receiving Durvalumab+C, randomly assigned to each arm without stratification, assuming accrual would take place over approximately 1 to 1.5 years, and assuming that there would be at least 6 months of additional potential follow-up after the last patient has received either therapy, there would be 80% power to determine whether there is a difference between a 3.5 month median PFS and an improved 6 month median PFS, with a one sided 0.10 alpha level test, using the method of Brookmeyer and Crowley (Brookmeyer R and Crowley, JJ. A confidence interval for the median survival time. *Biometrics,* 38, 29-41, 1982.). For each cohort, an early stopping rule will be implemented: if after 20 patients have been enrolled and potentially followed for 3 months, the median PFS is 3.5 months or less, that arm will no longer enroll any patients. In practice, Kaplan-Meier curves and

appropriate confidence intervals at selected time points will be provided to help interpret results relative to the expected results.

11.4 PHASE II SMALL CELL LUNG CANCER (SCLC) COHORT 3 (DURVALUMAB+O ONLY)

SCLC patients will only receive Durvalumab+O. For patients with SCLC, this trial will be conducted using an optimal two-stage phase II trial design (Simon R, Controlled Clinical Trials 10:1-10, 1989) in order to rule out an unacceptably low PR+CR rate of 15% (p0=0.15) in favor of an improved response rate of 35% (p1=0.35). With alpha=0.10 (probability of accepting a poor treatment=0.10) and beta = 0.10 (probability of rejecting a good treatment=0.10), this first stage will initially enroll 19 evaluable patients, and if 0 to 3 of the 19 have a response, then no further patients will be accrued in this cohort. If 4 or more of the first 19 patients have a response, then accrual would continue until a total of 33 patients have been enrolled. As it may take several weeks to determine if a patient has experienced a response, a temporary pause in the accrual may be necessary to ensure that enrollment to the second stage is warranted. If there are 4 to 7 responses in 33 patients, this would be an uninterestingly low response rate. If there were 8 or more responses in 33 patients (24.2%), this would be sufficiently interesting to warrant further study in later trials. Under the null hypothesis (15% response rate), the probability of early termination is 68%.

11.5 PHASE II METASTATIC CASTRATE-RESISTANCE PROSTATE CANCER (MCRPC) COHORT 4 (DURVALUMAB+O ONLY)

The primary objective of this trial is to establish in a small number of patients if the progression free survival (PFS) of patients treated with Durvalumab in combination with olaparib (Durvalumab+O) for mCRPC is sufficiently high to consider further development of this regimen in that patient population.

Once the optimal dose level of Durvalumab+O has been determined, patients with mCRPC will be enrolled in a pilot/phase II evaluation to determine if the use of Durvalumab+O can be associated with an improved PFS consistent with 70% PFS at 4 months compared to an estimated 50% PFS at 4 months. With 25 evaluable patients receiving Durvalumab+O and assuming accrual would take place over approximately 3 years, and that there would be at least 8 months of additional potential follow-up after the last patient has received therapy, there would be 80% power to determine whether there is a difference between a 50% 4 month PFS and an improved 70% 4 month PFS, with a one sided 0.10 alpha level test, using the method of Brookmeyer and Crowley (Brookmeyer R and Crowley, JJ. A confidence interval for the median survival time, *Biometrics,* 38, 29-41, 1982). In addition, an early stopping rule will be implemented: if after 12 patients have been enrolled and potentially followed for 6 months, the 4 month PFS is 50% or less, this mCRPC cohort will no longer enroll any patients. In practice, Kaplan-Meier curves and appropriate confidence intervals at selected time points will be provided to help interpret results relative to the expected results.

11.5.1 ***Expansion cohort*** *(effective with amendment M [version date 7/19/2017])*

The primary objective of this expansion cohort is to determine the clinical response rate to Durvalumab-O in patients with prostate cancer who do not have a DNA damage repair pathway mutation. At present, in patients who do not have a DNA damage repair pathway mutation, the objective response rate is approximately 20-25% with a checkpoint inhibitor alone. As such, the objective of this study will be to determine if the objective response rate may be raised to be 30% or higher in these patients.

Because determination of the presence of a DNA damage repair pathway mutation cannot be made until after treatment will have been underway or completed, the trial will plan to enroll 65 patients in this cohort based on the following assumptions. It is expected that approximately 30% of patients will have the DNA damage repair pathway mutation and 70% will not. Thus, out of 65 patients, it is anticipated that approximately 45 patients (69.2%) will not have the DNA damage repair pathway mutation, but it is possible that as few as 40 (or even less) may have this condition. For these patients without the DNA damage repair pathway mutation, it would be desirable if their response rate would exceed 20% and be consistent with 35% or greater. An exact binomial test with a 0.10 one-sided significance level test will have 84% power to detect a difference between a 20% response rate and a 35% response rage when there are 45 patients. Using that as a guideline, if there are 45 patients without the DNA damage repair pathway mutations who receive the combination treatment, 14 responses in 45 patients (31.1%) would be associated with a two-sided 90% exact confidence interval of 19.9-44.3%, and 13 responses in 45 (28.9%) would be associated with a two-sided 90% exact confidence interval of 18.0-42.0%. With 40 patients without the proper mutations, 13 responses in 40 (32.5%) is associated with a confidence interval of 20.4-46.6%, and 12 responses in 40 (30.0%) would have a confidence interval of 18.3-44.0%. The values for 41-44 patients have similar confidence intervals. Thus, in the expected range of 40-45 patients without mutations, a minimum of 12-13 responses, but preferably 14 or more depending on the number without a DNA damage repair pathway mutation, would be the lowest numbers of responses obtained which could be considered sufficiently interesting to allow the treatment to be evaluated further in these patients.

If among the first 12 treated patients with prostate cancer who do not have DNA damage repair pathway mutations, only 1 of 12 responds, then no further patients will be enrolled as soon as this can be determined, since the upper one-sided 90% CI bound on 1/12 = 28.8%, which would be below the intended goal of 30% or greater response rate in these patients.

In a published study of 16 patients with prostate cancer who have one of a set of specified mutations, 14 (87.5%) had experienced a clinical response when treated with olaparib alone. While it is unlikely that this could be improved upon, it would be of interest to explore if a similarly high response rate could be seen in patients treated at the NCI with Durvalumab and olaparib (Durvalumab-O). In the remaining patients, estimated to be 20-25 from among the 65 total patients to be enrolled, the fraction who respond to Durvalumab-O will be determined and reported along with 80% and 95% two-sided confidence intervals.

Patients who are included in the other mCRPC cohort who can have their DNA damage repair pathway mutation status determined may be included as part of this cohort. Thus, depending on how many of the 25 patients in that cohort may be included, as few as 40 additional evaluable patients may need to be enrolled for evaluation in this cohort.

11.6 Phase II colorectal cancer (CRC) Cohort 6 (C+Durvalumab only)

After establishment of a safe and tolerable dose for Durvalumab+C, a cohort of N=13 patients with CRC will be enrolled. Based on studies in a similar (refractory) patient population the expected median progression free survival (PFS) is approximately 1.9 months. The primary objective of this portion of the trial is to determine in a preliminary fashion whether the use of C+Durvalumab may be able to be associated with improved PFS in patients with irinotecan-refractory colon cancer compared to that associated with ISIS183750 and irinotecan.

Data from the NCI in patients with irinotecan-refractory CRC treated with ISIS183750 in combination with irinotecan indicated that the expected median PFS of a similar patient population was 1.9 months. The goal is to determine in a preliminary fashion if use of Durvalumab+C in such patients with irinotecan-refractory colon cancer may potentially be able to be associated with an improved PFS from 1.9 months to a level consistent with 5.0 months. With 13 evaluable patients receiving C+Durvalumab, assuming accrual would take place over approximately 9 to 12 months, and assuming that there would be at least 4 months of additional potential follow-up after the last patient has received therapy, there would be 80% power to determine whether there is a difference between a 1.9 month median PFS and an improved 5 month median PFS, with a one sided 0.10 alpha level test, using the method of Brookmeyer and Crowley (Brookmeyer R and Crowley, JJ. A confidence interval for the median survival time. Biometrics, 38, 29-41, 1982.). In practice, Kaplan-Meier curves and appropriate confidence intervals at selected time points will be provided to help interpret results relative to the expected results. In addition, preliminary estimates of overall survival and of the clinical response rate will also be determined, each reported as being preliminary, secondary results, with appropriate confidence intervals. Biopsies (mandatory pretreatment and optional post-treatment) will also be obtained to determine changes in the levels of correlative parameters. For a given measure, if there are at least 10 evaluable patients with determinations at the two time-points, there would be at least 80% power to detect a change equal to one SD of the change (effect size=1.0), using a paired t-test with a 0.05 two-sided significance level. In practice, unless the changes are normally distributed (p>0.05 by a Shapiro-Wilks test), a Wilcoxon signed rank test will be used. The results of the correlative studies will be reported without formal adjustment for multiple comparisons, but in the context of the number of such tests performed.

11.7 Phase II Durvalumab+O+C in patients with ovarian cancer

Effective with amendment M (version date 7/19/2017), patients with ovarian cancer will be enrolled to receive this combination. The primary objective of this cohort will be to estimate the overall response rate in patients with recurrent ovarian cancer.

Preliminary results from this trial in patients with ovarian cancer show 2 PRs in 10 patients treated with Durvalumab+O and 3 PRs in 7 patients treated with Durvalumab+C. It would be desirable if the overall response rate to Durvalumab+O+C would demonstrate at least the same overall results or better than the experience with Durvalumabplus either agent alone. Recognizing the early nature of the results to date, this arm of the trial will be conducted using an optimal two-stage phase II trial design in order to rule out an unacceptably low PR+CR rate of 20% (p0=020) in favor of an improved response rate of 40% (p1=0.40). With alpha=0.10 (probability of accepting a poor treatment=0.10) and beta = 0.10 (probability of rejecting a good

treatment=0.10), the first stage will initially enroll 17 evaluable patients, and if 0 to 3 of the 17 have a response, then no further patients will be accrued. As it may take several weeks to determine if a patient has experienced a response, a temporary pause in the accrual may be necessary to ensure that enrollment to the second stage is warranted. If 4 or more of the first 17 patients have a response, then accrual would continue until a total of 37 patients have enrolled. If there are 4 to 10 responses in 37 patients, this would be an uninterestingly low response rate. If there were 11 or more responses in 37 patients (29.7%), this may be sufficiently interesting to warrant further study in later trials. Under the null hypothesis (20% response rate), the probability of early termination is 54.9%. At the conclusion of accrual to this cohort, the patients will have their response rates reported overall, and a separate exploratory analysis according to whether the patients are platinum-sensitive or platinum-resistant may also be undertaken. In all cases, the actual response rates and the associated 95% confidence intervals will be reported.

11.8 Expected patient enrollment and accrual ceiling

For Phase I and Phase II OvCa cohort 1, it is expected that 24-36 patients per year may enroll onto this trial. Thus, approximately 3-3.5 years may be required in order to enroll up to 66 evaluable patients in the phase I portion and to enroll up to 107 evaluable patients in the phase II portion, allowing for a small number of inevaluable patients, the accrual ceiling should be set at 183 patients.

For Phase II TNBC patients cohort 5, it is expected that approximately 20-24 patients per year may enroll on this trial; thus it is anticipated that approximately one and half years may be required for accrual of up to 35 evaluable patients, approximately 25 patients with BRCAwt and approximately 10 patients with BRCAwt. In order to allow for a small number of non-evaluable patients, the accrual ceiling will be set at 38.

To conduct this Phase II pilot portion of the trial in all three lung cancer arms (cohorts 2 and 3), a total of up to 37+33=70 patients may be required. Based upon current accrual rates it is anticipated that all three cohorts can complete accrual in less than 2 years. In order to allow for a small number of inevaluable patients, the accrual ceiling for this portion of the trial will be set at 78.

For Phase II mCRPC cohort 4, it is expected that approximately 1-2 patients per month may enroll onto this cohort in the trial. Thus, it is expected that 3 - 5 years may be required to enroll up to 65 evaluable patients. To allow for a small number of inevaluable patients, the accrual ceiling for this cohort will be set at 70 patients.

For Phase II CRC cohort 6, in order to enroll 13 evaluable CRC patients, the accrual ceiling for this portion of the trial will be set at 15 patients; it is anticipated that all patients for this cohort will be accrued within one year.

In order to enroll patients of all six disease types (cohorts 1-6) and the four treatment arms (Durvalumab+O, Durvalumab+C, C+Durvalumab and Durvalumab+O+C) as described above for the Phase I and II portions of the trial, the overall accrual ceiling will be set at 384 patients.

Effective with Amendment O (version date 7/25/2018),

- Phase II Durvalumab+O Ovarian cancer cohort was closed due to completed enrollment.
- Phase II Durvalumab+O Small cell lung cancer cohort was closed due to completed enrollment.
- Phase II Durvalumab+C Non-small cell lung cancer cohort was closed due to slow accrual. No patients enrolled on this Durvalumab+C arm.
- Phase II Durvalumab+C CRC cohort was closed due to completed enrollment.

12 COLLABORATIVE AGREEMENTS

The study agents, Durvalumab, olaparib and cediranib will be provided under an umbrella CRADA (#2299) executed between the manufacturers (AstraZeneca and MedImmune) and the Women's Malignancies Branch, Center for Cancer Research, National Cancer Institute.

A Sample Transfer Agreement with the designated collaborating institution will be executed prior to the shipment of any samples from NCI to the collaborator(s) for the purpose of biomarker, correlative, and/or special studies (effective with the Phase II portion of the trial).

13 HUMAN SUBJECTS PROTECTIONS

13.1 Rationale For Subject Selection

Subjects from all racial and ethnic groups are eligible for this trial if they meet the eligibility criteria. Efforts will be made to extend the accrual to a representative population. If differences in outcome that correlate to racial or ethnic identity are noted, accrual may be expanded or additional studies may be performed to investigate those differences more fully.

13.2 Participation of Children

The age group for enrollment on this trial is 18 or more years of age. Because no dosing or adverse event data are currently available on the use of Durvalumab, cediranib or olaparib in participants < 18 years of age, children are excluded from this study.

13.3 Participation of Subjects Unable to Give Consent

Adults unable to give consent are excluded from enrolling in the protocol. However re-consent may be necessary and there is a possibility, though unlikely, that subjects could become decisionally impaired. For this reason and because there is a prospect of direct benefit from research participation (section **13.5**), all subjects ≥ age 18 will be offered the opportunity to fill in their wishes for research and care, and assign a substitute decision maker on the "NIH Advance Directive for Health Care and Medical Research Participation" form so that another person can make decisions about their medical care in the event that they become incapacitated or cognitively impaired during the course of the study.

Note: The PI or AI will contact the NIH Ability to Consent Assessment Team (ACAT) for evaluation to assess ongoing capacity of the subjects and to identify an LAR, as needed. Please see section **13.6.1** for consent procedure.

13.4 EVALUATION OF BENEFITS AND RISKS/DISCOMFORTS

13.4.1 Risks

13.4.1.1 Study Drug Risks

The primary risks to patients participating in this research study are associated with the toxicities of the study agents, as outlined in the Investigator Brochure and in the informed consent. Patients will be routinely monitored while on study in order to detect and treat any adverse events as quickly as possible.

- In brief, the most common adverse events (AEs) with cediranib included fatigue, diarrhea, nausea, vomiting, hoarseness, hand-foot syndrome, and hypertension.
- AEs considered to be associated with olaparib included anemia (mild to moderate), neutropenia (mild to moderate), and thrombocytopenia (mild to moderated, sometimes severe), stomatitis, nausea and vomiting (mild to moderate), and fatigue (mild to moderate).
- AEs associated with Durvalumab include fatigue, nausea, dyspnea, diarrhea, vomiting, pyrexia, myalgia, hypothyroidism, decreased appetite, dizziness, cough, pruritus, rash, abdominal pain, increased aspartate aminotransferase and alanine aminotransferase, arthralgia, asthenia, influenza-like illness, edema peripheral, headache, dry skin, and constipation; Grade 3 or higher AEs included increased aspartate aminotransferase, hypothyroidism, vomiting, fatigue, infusion-related reaction, troponin, dehydration, and arthralgia.

13.4.1.2 Specimen Collection Risks

Risks include those associated with specimen collection including pain, bleeding and the possibility of infection at the sampling site. All care will be taken to minimize risks that may be incurred by tumor sampling. However, there are procedure-related risks (such as bleeding, infection and visceral injury) that will be explained fully during informed consent. If patients suffer any physical injury as a result of the biopsy, immediate medical treatment is available at the NIH's Clinical Center in Bethesda, Maryland. Although no compensation is available, any injury will be fully evaluated and treated in keeping with the benefits or care to which patients are entitled under applicable regulations.

13.4.1.3 Privacy Risks

This includes the risk that data related to your BRCA status can be released to members of the public, insurers, employers, or law enforcement agencies.

As part of study efforts to provide confidentiality of subject information, this study will obtain a Certificate of Confidentiality, which helps to prevent forced disclosure of personally identifiable research information. The Certificate of Confidentiality allows investigators on this trial to refuse to disclose identifying information related to the research participants, should such disclosure have adverse consequences for subjects or damage their financial standing, employability, insurability or reputation. The informed consent includes the appropriate coverage and restrictions of the Certificate of Confidentiality.

13.4.1.4 Radiation Risks

The study will involve radiation from the following sources:

- Up to 3 CT scans for the collection of biopsies (Phase II participants only)
- Up to 8 CT scans per year for disease assessment for all participants except for the Ph II Cohort 4 (Prostate Cancer), which has up to 6 CT scans per year.
- Up to 6 technetium-99 bone scans for disease assessment (Ph II Cohort 4 Prostate Cancer only)

Subjects in the Phase I portion of the study may be exposed to approximately 8.8 rem. This amount is more than would be expected from everyday background radiation. Being exposed to excess radiation can increase the risk of cancer. The risk of getting cancer from the radiation exposure in the Phase I portion of this study is 0.9 out of 100 (0.9%) and of getting a fatal cancer is 0.4 out of 100 (0.4%).

Subjects in Phase II Cohort 4 may be exposed to approximately 11.34 rem. The risk of getting cancer from the radiation exposure in Phase II Cohort 4 is 1.1 out of 100 (1.1%) and of getting a fatal cancer is 0.6 out of 100 (0.6%).

Subjects in all other Phase II Cohorts (1, 2, 3, 5, and 6) may be exposed to approximately 11.2 rem. The risk of getting cancer from the radiation exposure in Phase II Cohort 4 is 1.1 out of 100 (1.1%) and of getting a fatal cancer is 0.6 out of 100 (0.6%).

13.4.1.5 Risks from Blood Draws: Side effects of blood draws include pain and bruising, lightheadedness, and rarely, fainting.

13.4.1.6 Risks of CT Scans: In addition to the radiation risks discussed above, risks associated with CT scans are allergic reaction to and kidney damage from the contrast dye, nausea, vomiting, and anxiety.

13.4.1.7 Risks of Technetium-99 Bone Scans: In addition to the radiation risks described above, risks associated with technetium-99 bone scans are pain, bruising, injection site infection, and allergic reaction to the contrast agent.

13.4.2 Benefits

The potential benefit to a patient on this study is a reduction in the bulk of their tumor and improvement in cancer lesions, which may or may not have favorable impact on symptoms and/or survival.

13.5 RISKS/BENEFITS ANALYSIS

Patients will receive clinical evaluation of their disease at the NCI Clinical Center. This protocol may or may not benefit an individual, but the results may help the investigators learn more about the disease and develop new treatments for patients with this disease.

Patients will be examined and evaluated prior to enrollment. All evaluation to monitor the treatment of patients will be recorded in the patient record. If patients suffer any physical injury as a result of participation in this study, immediate medical treatment is available at the Clinical Center, NCI, in Bethesda, Maryland.

Although no compensation is available, any injury will be evaluated and treated in keeping with the benefits or care to which patients are entitled under applicable regulations. In all publications and presentation resulting from this trial, patients' anonymity will be protected to the maximum extent possible. Authorized personnel from the NCI and Food and Drug Administration (FDA) or other regulatory authorities may have access to research files in order to verify that patients'' rights have been appropriately safeguarded. In addition, patient names will be given to the Central Registration Office to register and verify eligibility.

13.6 CONSENT PROCESS AND DOCUMENTATION

The informed consent document will be provided as a physical or electronic document to the participant or consent designee(s) (e.g., legally authorized representative [LAR] if participant is an adult unable to consent) for review prior to consenting. A designated study investigator will carefully explain the procedures and tests involved in this study, and the associated risks, discomforts and benefits. In order to minimize potential coercion, as much time as is needed to review the document will be given, including an opportunity to discuss it with friends, family members and/or other advisors, and to ask questions of any designated study investigator. A signed informed consent document will be obtained prior to entry onto the study.

The initial consent process as well as re-consent, when required, may take place in person or remotely (e.g., via telephone or other NIH approved remote platforms used in compliance with local policy, including HRPP Policy 303) per discretion of the designated study investigator and with the agreement of the participant/consent designee(s). Whether in person or remote, the privacy of the subject will be maintained. Consenting investigators (and participant/consent designee, when in person) will be located in a private area (e.g., clinic consult room). When consent is conducted remotely, the participant/consent designee will be informed of the private nature of the discussion and will be encouraged to relocate to a more private setting if needed.

Note: When required, witness signature will be obtained similarly as described for the investigator and participant as described below.

Consent will be documented with required signatures on the physical document (which includes the printout of an electronic document sent to participant) or on the electronic document. Signatures on electronic documents are described below. Note: FDA only regulates electronic signatures (i.e., an electronic timestamp is generated at the time of signature) in FDA regulated research.

Manual (non-electronic) signature on electronic document:

When a manual signature on an electronic document is used for the documentation of consent at the NIH Clinical Center, this study will use the Adobe platform (which is not 21 CFR Part 11 compliant) to obtain the required signatures.

During the consent process, participants and investigators will view individual copies of the approved consent document on screens at their respective locations.

Both the investigator and the subject will sign the document using a finger, stylus or mouse.

Electronic signature on electronic document:

When permitted by the NIH Clinical Center, an electronic signature may be obtained using the iMedConsent platform to obtain the required signatures once it is designated as 21 CFR Part 11 compliant.

During the consent process, participants and investigators will view individual copies of the approved consent document on screens at their respective locations.

The identity of the participant will be determined by a prompt which will require the provision of information from a form of government-issued identification prior to obtaining the signature. If participant does not have such identification available, security questions will be used to confirm identity.

Both the investigator and the subject will sign the document electronically per system prompts.

For the optional research biopsies in the protocol, the patient will consent at the time of the procedure. If the patient refuses the optional biopsy at that time, the refusal will be documented in the medical record and in the research record.

13.6.1 Consent Process for Adults Who Lack Capacity to Consent to Research Participation

For participants addressed in section **13.3**, an LAR will be identified consistent with Policy 403 and informed consent obtained from the LAR, as described in Section **13.6**.

13.6.2 Request for Waiver of Consent for Screening Activities

Prior to the subject signing the consent for this study pre-screening activities listed in section **2.7.1** may be performed.

We request a waiver of consent for these activities as they involve only minimal risk to the subjects. A waiver will not adversely affect the rights and welfare of the subjects given that the activities are only intended to determine suitability for screening for participation in research protocols. These activities could not practicably be carried out without the wavier as central recruiting services, utilized in the NIH Clinical Center, perform pre-screening activities for multiple studies and obtaining consent for each one is beyond their resources. The subjects will be provided with additional pertinent information after participation as they will be informed whether or not they are eligible to sign a consent for additional screening.

13.7 STUDY DOCUMENTATION

The investigator must prepare and maintain adequate and accurate case histories designed to record all observations and other data pertinent to the study for each research participant. This information enables the study to be fully documented and the study data to be subsequently verified.

13.8 RECORDS RETENTION

All study-related documents must be retained for the maximum period required by applicable federal regulations and guidelines or institutional policies.

14 REGULATORY AND OPERATIONAL CONSIDERATIONS

14.1 STUDY DISCONTINUATION AND CLOSURE

This study may be temporarily suspended or prematurely terminated if there is sufficient reasonable cause. Written notification, documenting the reason for study suspension or termination, will be provided by the suspending or terminating party to study participants, investigator, funding agency, the Investigational New Drug (IND) sponsor and regulatory authorities. If the study is prematurely terminated or suspended, the Principal Investigator (PI) will promptly inform study participants, the Institutional Review Board (IRB), and sponsor and will provide the reason(s) for the termination or suspension. Study participants will be contacted, as applicable, and be informed of changes to study visit schedule.

Circumstances that may warrant termination or suspension include, but are not limited to:

- Determination of unexpected, significant, or unacceptable risk to participants
- Demonstration of efficacy that would warrant stopping
- Insufficient compliance to protocol requirements
- Data that are not sufficiently complete and/or evaluable
- Determination that the primary endpoint has been met
- Determination of futility

Study may resume once concerns about safety, protocol compliance, and data quality are addressed, and satisfy the sponsor, IRB and as applicable, Food and Drug Administration (FDA).

14.2 QUALITY ASSURANCE AND QUALITY CONTROL

The clinical site will perform internal quality management of study conduct, data and biological specimen collection, documentation and completion. An individualized quality management plan will be developed to describe a site's quality management.

Quality control (QC) procedures will be implemented beginning with the data entry system and data QC checks that will be run on the database will be generated. Any missing data or data anomalies will be communicated to the site(s) for clarification/resolution.

Following written Standard Operating Procedures (SOPs), the monitors will verify that the clinical trial is conducted and data are generated and biological specimens are collected, documented (recorded), and reported in compliance with the protocol, International Conference on Harmonisation Good Clinical Practice (ICH GCP), and applicable regulatory requirements (e.g., Good Laboratory Practices (GLP), Good Manufacturing Practices (GMP)).

The investigational site will provide direct access to all trial related sites, source data/documents, and reports for the purpose of monitoring and auditing by the sponsor, and inspection by local and regulatory authorities.

14.3 CONFLICT OF INTEREST POLICY

The independence of this study from any actual or perceived influence, such as by the pharmaceutical industry, is critical. Therefore, any actual conflict of interest of persons who have a role in the design, conduct, analysis, publication, or any aspect of this trial will be disclosed and managed. Furthermore, persons who have a perceived conflict of interest will be required to have such conflicts managed in a way that is appropriate to their participation in the design and conduct of this trial. The study leadership in conjunction with the National Cancer Institute has established policies and procedures for all study group members to disclose all conflicts of interest and will establish a mechanism for the management of all reported dualities of interest.

14.4 CONFIDENTIALITY AND PRIVACY

Participant confidentiality and privacy is strictly held in trust by the participating investigators, their staff, and the sponsor(s). This confidentiality is extended to cover testing of biological samples and genetic tests in addition to the clinical information relating to participants. Therefore, the study protocol, documentation, data, and all other information generated will be held in strict confidence. No information concerning the study or the data will be released to any unauthorized third party without prior written approval of the sponsor.

All research activities will be conducted in as private a setting as possible.

The study monitor, other authorized representatives of the sponsor, representatives of the Institutional Review Board (IRB), and/or regulatory agencies may inspect all documents and records required to be maintained by the investigator, including but not limited to, medical records (office, clinic, or hospital) and pharmacy records for the participants in this study. The clinical study site will permit access to such records.

The study participant's contact information will be securely stored at the/each clinical site for internal use during the study. At the end of the study, all records will continue to be kept in a secure location for as long a period as dictated by the reviewing IRB, Institutional policies, or sponsor requirements.

Study participant research data, which is for purposes of statistical analysis and scientific reporting, will be stored at the NCI CCR. This will not include the participant's contact or identifying information. Rather, individual participants and their research data will be identified by a unique study identification number. The study data entry and study management systems used by the NCI CCR research staff will be secured and password protected. At the end of the study, all study databases will be archived at the NIH.

To further protect the privacy of study participants, a Certificate of Confidentiality has been issued by the National Institutes of Health (NIH). This certificate protects identifiable research information from forced disclosure. It allows the investigator and others who have access to research records to refuse to disclose identifying information on research participation in any civil, criminal, administrative, legislative, or other proceeding, whether at the federal, state, or local level. By protecting researchers and institutions from being compelled to disclose information that would identify research participants, Certificates of Confidentiality help achieve

the research objectives and promote participation in studies by helping assure confidentiality and privacy to participants.

15 PHARMACEUTICAL INFORMATION: DRUG FORMULATION AND ADMINISTRATION

15.1 DURVALUMAB

15.1.1 Source

Durvalumab will be supplied under a collaborative agreement with AstraZeneca, parent company of the agent manufacturer MedImmune, and the NCI.

15.1.2 Toxicities

15.1.2.1 Identified Toxicities

Detailed safety data are available from a Durvalumab monotherapy study, 10 mg/kg every 2 weeks cohort (NCT01693562, Durvalumab IB V8.0 2015). Of the 694 patients treated in this cohort, the majority of AEs were grades 1 to 2 and manageable with optimal medical treatment. AEs (all grades) related to Durvalumab in > 10 % of patients were fatigue, nausea, dyspnea, decreased appetite, cough, constipation, diarrhea and vomiting, pyrexia and back pain, and arthralgia. Treatment-related grade 3 AEs in 10 or more patients were dyspnea, pneumonia, sepsis, abdominal pain, pleural effusion, and dehydration. 25 patients (3.6%) had SAEs considered to be related to Durvalumab. Durvalumab-related SAEs in > 2 patients were colitis and pneumonitis (3 patients each). A majority of the treatment-related SAEs were grade 3 or higher in severity and recovered with or without sequelae.

One patient died due to pneumonia considered to be related to Durvalumab. Before this fatal event of pneumonia, this patient experienced treatment-related to grade 4 pneumonitis and grade 3 pneumonia. A total of 76 patients (11%) treated with 10 mg/kg q2 weeks permanently discontinued Durvalumab due to AEs; events that resulted in discontinuation of Durvalumab in > 2 subjects were general physical health deterioration (6 subjects), non-small cell lung cancer (5 subjects) and colitis (3 subjects).

As with any antibody, allergic reactions to dose administration are possible. Across multiple Durvalumab monotherapy trials, there have been few infusion related reactions related to Durvalumab, with an incidence of 0.8 % (10/1,265 patients). The typical onset can be within 30 minutes to 2 hours after the initiation of drug infusion, although symptoms may be delayed for up to 24 hours. The majority of reactions occur after the first or second exposure to the agent but between 10% and 30% occur during subsequent treatments. In the monotherapy study with Durvalumab 10 mg/kg administered every 2 weeks, 5 infusion reactions were reported as grade 2 and 3 events. In a combination study with Durvalumab 3 mg/kg every 2 weeks and dabrafenib or trametinib, one patient had 2 infusion reactions, reportedly grade 1 and 2. All events resolved, were nonserious, and were managed with standard clinical practice.

Durvalumab IB v16 Table 30 (for reference):

Table 31 Frequency of ADRs for Durvalumab Monotherapy From Pooled Data Across Multiple Tumour Types (N=3006)

SOC	PT/medical concept MedDRA (v21.1)	CIOMS frequency indicator [a]/ overall frequency all CTC grades	All CTCAE Grades n (%)	Grade 3 or 4 n (%)
Respiratory, thoracic, and mediastinal disorders	Cough/productive cough	Very common	646 (21.5)	11 (0.4)
	Pneumonitis [c]	Common	114 (3.8)	26 (0.9)
	Dysphonia	Common	93 (3.1)	2 (<0.1)
	ILD	Uncommon	18 (0.6)	4 (0.1)
Hepatobiliary disorders	ALT increased/AST increased [b, c]	Common	244 (8.1)	69 (2.3)
	Hepatitis [b, c]	Uncommon	25 (0.8)	12 (0.4)
Gastrointestinal disorders	Diarrhoea	Very common	491 (16.3)	19 (0.6)
	Abdominal pain [b]	Very common	383 (12.7)	53 (1.8)
	Colitis [b]	Uncommon	28 (0.9)	10 (0.3)
Endocrine disorders	Hypothyroidism [b]	Very common	305 (10.1)	5 (0.2)
	Hyperthyroidism [b]	Common	137 (4.6)	0
	Thyroiditis [b]	Uncommon	23 (0.8)	2 (<0.1)
	Adrenal insufficiency	Uncommon	18 (0.6)	3 (<0.1)
	Type 1 diabetes mellitus	Rare	1 (<0.1)	1 (<0.1)
	Hypophysitis/Hypopituitarism	Rare	2 (<0.1)	2 (<0.1)
	Diabetes insipidus	Rare	1 (<0.1)	1 (<0.1)
Renal and urinary disorders	Blood creatinine increased	Common	105 (3.5)	3 (<0.1)
	Dysuria	Common	39 (1.3)	0
	Nephritis [b]	Uncommon	9 (0.3)	2 (<0.1)
Skin and subcutaneous tissue disorders	Rash [b]	Very common	480 (16.0)	18 (0.6)
	Pruritus [b]	Very common	325 (10.8)	1 (<0.1)

Table 31 Frequency of ADRs for Durvalumab Monotherapy From Pooled Data Across Multiple Tumour Types (N=3006)

SOC	PT/medical concept MedDRA (v21.1)	CIOMS frequency indicator [a]/ overall frequency all CTC grades	All CTCAE Grades n (%)	Grade 3 or 4 n (%)
	Night sweats	Common	47 (1.6)	1 (<0.1)
	Dermatitis	Uncommon	22 (0.7)	2 (<0.1)
	Pemphigoid [b]	Rare	3 (<0.1)	0
Cardiac disorders	Myocarditis	Rare	1 (<0.1)	1 (<0.1)
General disorders and administration site conditions	Pyrexia	Very common	414 (13.8)	10 (0.3)
	Oedema peripheral [b]	Common	291 (9.7)	9 (0.3)
Infections and infestations	Upper respiratory tract infections [b]	Very common	407 (13.5)	6 (0.2)
	Pneumonia [b,c]	Common	269 (8.9)	106 (3.5)
	Oral candidiasis	Common	64 (2.1)	0
	Dental and oral soft tissue infections [b]	Common	50 (1.7)	1 (<0.1)
	Influenza	Common	47 (1.6)	2 (<0.1)
Musculoskeletal and connective tissue disorders	Myalgia	Common	178 (5.9)	2 (<0.1)
	Myositis	Uncommon	6 (0.2)	1 (<0.1)
	Polymyositis [d]	Not determined	-	-
Nervous system disorders	Myasthenia gravis [e]	Not determined	-	-
Injury, poisoning, and procedural complications	Infusion related reaction [b]	Common	49 (1.6)	5 (0.2)
Blood and lymphatic system disorders	Immune thrombocytopenia [c]	Rare	2 (<0.1)	1 (<0.1)

[a] The corresponding frequency category for each ADR is based on the CIOMS III convention and is defined as: (1) very common (≥1/10); (2) common (≥1/100 to <1/10); (3) uncommon (≥1/1000 to

<1/100); (4) rare (≥1/10000 to <1/1000); (5) very rare (<1/10000); not determined (cannot be estimated from available data).

[b] Denotes a medical concept. See Table 31 for individual PTs.

[c] Fatal events have been reported.

[d] Polymyositis (fatal) was observed in a patient treated with durvalumab from an ongoing AstraZeneca-sponsored clinical study outside of the pooled dataset.

[e] Reported frequency from AstraZeneca-sponsored clinical studies outside of the pooled dataset is rare, with no events at Grade >2.

ADR = adverse drug reaction; ALT = alanine aminotransferase; AST = aspartate aminotransferase; CIOMS = Council for International Organisations of Medical Sciences; CTC = Common Toxicity Criteria; CTCAE = Common Terminology Criteria for Adverse Events; DCO = data cut-off; ILD = interstitial lung disease; MedDRA = Medical Dictionary for Regulatory Activities; N = total number of patients; PT = preferred term; SOC = system organ class.

15.1.2.2 Potential Risks

Durvalumab, an anti-PD-L1 antibody, binds with high affinity and specificity to PD-L1 and blocks its binding to PD-1 and CD80, thus promoting antitumor immunity and tumor cell killing. The important potential risks, based on the mechanism of action of Durvalumab, as well as data from studies of relevant or similar therapies include: immune-mediated reactions such as enterocolitis, dermatitis, hepatotoxicity, endocrinopathy, pneumonitis, and neuropathy; see Section **9.1.6** for further definitions. In addition, it is important to note that overlapping toxicities are expected with each of the combinations being used in this study.

Development of serious infection is a theoretical risk based on findings from nonclinical safety studies.

As with the administration of any immunoglobulin, infusion reactions and acute IgE-mediated allergic reactions may occur, may be severe, and may result in death.

Although Durvalumab is a human monoclonal antibody, it is possible for humans to develop anti-drug antibodies (ADA). The occurrence of such ADA could result in immune complex disease (with manifestations such as arthralgias, serum-sickness, abdominal pain, back pain, and vasculitis) or altered Durvalumab levels or activity.

Other rare or less frequent AESIs and immune-mediated adverse events:

Events with an inflammatory or immune mediated mechanism could occur in nearly all organs. Potential risks with an immune-mediated etiology that are rare or less frequent include, but are not limited to, Guillain-Barre Syndrome, myasthenia gravis, pericarditis, sarcoidosis, uveitis, and other events involving the eye (eg, keratitis and optic neuritis), skin (eg, scleroderma, vitiligo and pemphigoid), hematological (eg, hemolytic anemia and immune thrombocytopenic purpura), rheumatological events (polymyalgia rheumatic and autoimmune arthritis), vasculitis, non-infectious meningitis and non-infectious encephalitis.

15.1.3 Formulation and preparation

Durvalumab is formulated at 50 mg/mL in 26 mM histidine/histidine-HCl, 275 mM trehalose dihydrate, 0.02% (weight/volume [w/v]) polysorbate 80, pH 6.0. The investigational product is

supplied as a vialed liquid solution in clear 10R glass vials closed with an elastomeric stopper and a flip-off cap overseal. Each vial contains 500 mg (nominal) of active investigational product at a concentration of 50 mg/mL (500 mg/vial). The solution will be diluted with 0.9% (w/v) saline for IV infusion.

Calculate the dose volume of Durvalumab and number of vials needed for the subject to achieve the accurate dose according to Appendix I.

Preparation of infusion bags

The preparation of infusion bags should be done under aseptic conditions by trained personnel; it should not be prepared on the ward.

An additional volume of 0.9% (w/v) saline equal to the calculated volume of Durvalumab to be added to the IV bag must be removed from the bag prior to addition of Durvalumab.

The calculated volume of Durvalumab is then added to the IV bag, and the bag is mixed by gentle inversion to ensure homogeneity of the dose in the bag.

Vials should be used for specific patients and should not be shared between patients.

15.1.4 Stability and Storage

Unopened vials of liquid Durvalumab drug product must be stored at 2°C to 8°C (36°F to 46°F) and must be used within the individually assigned expiration date on the label. Vials should be used for specific patients and should not be shared between patients. Total in-use storage time from needle puncture of Durvalumab vial to start of administration should not exceed 4 hours at room temperature or 24 hours at 2-8°C (36-46°F). If in-use storage time is greater than 4 hours, a new dose must be prepared from new vials. Infusion solutions must be allowed to equilibrate to room temperature prior to starting administration. Durvalumab does not contain preservatives and any unused portion must be discarded.

15.1.5 Administration procedures

Please see section **3.2.1**

15.1.6 Incompatibilities

No formal drug-drug interaction studies have been conducted with Durvalumab. There are no known clinically significant interactions of Durvalumab with other medicinal products.

15.2 OLAPARIB

15.2.1 Source

Olaparib is the first FDA-approved PARPi and will be provided to the NCI under a collaborative agreement with AstraZeneca and the NCI.

15.2.2 Toxicity

As of May 2014, approximately 2103 patients with ovarian, breast, pancreatic, gastric, and other solid tumors had been treated with olaparib as monotherapy or in combination with other agents.

The recommended monotherapy capsule dose is 400 mg twice-daily. Olaparib (capsule) monotherapy appears to be generally well tolerated across studies at doses up to and including the MTD of 400 mg twice-daily. In comparison, the recommended olaparib monotherapy tablet dose is 300 mg twice-daily, with similar tolerability profile to the 400 mg twice-daily capsule dose.

Preliminary data from Phase I dose escalation studies of olaparib in combination with various chemotherapy agents indicated an increase in bone marrow toxicity (anemia, neutropenia, thrombocytopenia) greater than expected if the agents had been administered alone.

Toxicities considered to be associated with administration of olaparib include hematological effects (anemia, neutropenia, lymphopenia, thrombocytopenia, mean corpuscular volume [MCV] elevation), decreased appetite, nausea and vomiting, diarrhea, dyspepsia, stomatitis, upper abdominal pain, dysgeusia, fatigue (including asthenia), increase in blood creatinine, headache and dizziness. These are discussed in further detail below:

Hematologic Disorders: Laboratory findings and/or clinical diagnoses of anemia (generally mild to moderate, grade 1 or 2), neutropenia (predominantly grade 1 or 2), and thrombocytopenia (generally grade 1 or 2) were observed. The incidence of anemia was 26 %, neutropenia was 1 %, and thrombocytopenia was 5 %. A few cases of myelodysplasia have been reported on studies including olaparib.

Gastrointestinal Disorders: The most common gastrointestinal AEs were nausea (56 of 98 patients, 57 %) and vomiting (44 of 98 patients, 45 %). Most were of mild to moderate intensity (grade 1 or 2), intermittent, and manageable on continued treatment. Abdominal pain (24 of 98 patients, 24.5 %), constipation (18 of 98 patients, 18.4 %), and diarrhea (25 of 98 patients, 25.5 %) were also observed.

MDS/AML have been reported in < 1 % of patients exposed to olaparib. The cases were typical of secondary MDS/therapy-related AML. The duration of therapy with olaparib in patients who developed secondary MDS/AML varied from < 6 months to > 2 years. All patients had potential contributing factors for the development of MDS/AML, having received extensive previous chemotherapy with platinum agents. Many had also received other DNA damaging agents.

General Disorders: The most common general AE reported was fatigue, observed in 52 patients (53 %). Most were intermittent and grade 1 or 2. Other AEs included pyrexia (10 patients, 10 %) and peripheral edema (16 patients, 16 %).

Nervous System Disorders: The most common nervous system AEs reported were headache (15 %), dizziness (11 %), and dysgeusia (13 %). Other reported AEs included somnolence, attention or mental impairment, cognitive disorder, dizziness, depression, and memory impairment.

Pneumonitis events have been reported in <1 % of patients receiving olaparib. The reports of pneumonitis had no consistent clinical pattern and were confounded by a number of predisposing factors (cancer and/or metastases in lungs, underlying pulmonary disease, smoking history, and/or previous chemotherapy and radiotherapy).

15.2.3 Formulation and preparation

Olaparib tablets are film-coated tablets and will be supplied in the 100 mg or 150 mg strength. Tablets are packaged in induction-sealed high-density polyethylene (HDPE) bottles with child-resistant closures and induction sealed membranes that provide tamper evidence. Each bottle contains 32 tablets with desiccant. Tablet core consists of olaparib, copovidone, colloidal silicon dioxide, mannitol and sodium stearyl fumarate. Tablet film-coating consists of hydroxypropyl methylcellulose (hypromellose), macrogol 400 (polyethylene glycol 400), titanium dioxide, iron oxide yellow and iron oxide black.

15.2.4 Stability and Storage

- Storage: Store the olaparib tablets below 30°C. Olaparib tablets may be repackaged by the Clinical Center Pharmacy Department and dispensed in light-resistant high-density polyethylene (HDPE) containers.
- Stability: Stability studies are ongoing.

15.2.5 Administration procedures

Please see section **3.2.2**.

15.2.6 Incompatibilities

Based on *in vitro* data and clinical exposure data, olaparib is considered unlikely to cause clinically significant drug interactions through inhibition or induction of CYP enzyme activity. *In vitro* data have shown that the principal enzyme responsible for oxidative metabolism of olaparib in human hepatic microsomes was CYP3A4, the major P450 enzyme responsible for drug metabolism in humans.

Consequently, to ensure patient safety, please follow the guidelines to avoid concurrent use of potent inhibitors and inducers of CYP3A4 listed in **Appendix B** for any patient receiving olaparib.

15.3 CEDIRANIB

15.3.1 Source

Cediranib is an investigational agent and will be provided to the NCI under a collaborative agreement with AstraZeneca and the NCI.

15.3.2 Toxicity

Overall, the common AEs (all grades, >20 % of 895 patients) seen with cediranib monotherapy include fatigue, diarrhea, and hypertension.

Cardiovascular Disorders: Hypertension is an expected AE with agents that inhibit VEGF signaling. In cediranib studies, increases in blood pressure have been observed and cases of hypertension have been reported, including grade 4 hypertension and end-organ damage related to hypertension, such as cerebrovascular events.

Left ventricular dysfunction is rare but has been observed in patients receiving cediranib with risk factors for left ventricular dysfunction (including previous or concomitant anthracycline treatment).

Gastrointestinal Disorders: Other less common AEs include abdominal pain, mucositis, vomiting, and rarely, gastrointestinal perforation.

Hematologic Disorders: A number of events of bleeding and hemorrhage have occurred, they were mostly mild and the most common type of bleeding was mucocutaneous (epistaxis).

Thrombocytopenia and neutropenia, predominantly grade 1 or 2, have been seen with cediranib monotherapy. In combination with chemotherapy, more frequent and severe neutropenia and thrombocytopenia have been observed.

Investigations: Proteinuria, a dose-related trend of increases from baseline in TSH levels, which may be associated with clinical hypothyroidism. Increases in transaminases, which are sometimes associated with increases in total bilirubin, have been seen.

Nervous System Disorders: Reversible posterior leukoencephalopathy syndrome (RPLS) has been observed in patients receiving cediranib in clinical studies (with more cases seen on cediranib than placebo/comparator).

15.3.3 Formulation and preparation

The drug product consists of a range film-coated tablets packed in HDPE bottles. The range is a series of beige film-coated tablets containing 15, or 20 mg cediranib free base. The 15 mg are 7 mm in diameter and the 20 mg are 8 mm in diameter.

The tablets contain cediranib maleate, mannitol, dibasic calcium phosphate anhydrous, sodium starch glycolate, microcrystalline cellulose and magnesium stearate with a film coat containing hypromellose 2910, polyethylene glycol 400, red iron oxide, yellow iron oxide, black iron oxide and titanium dioxide.

15.3.4 Stability and Storage

The tablets should be stored in the original pack until use. Store intact bottles at controlled room temperature (20-25 degrees Celsius, or 68-77 degrees Fahrenheit), and protect from light. Cediranib tablets may be repackaged by the Clinical Center Pharmacy Department and dispensed in light-resistant high-density polyethylene (HDPE) containers. For further information investigators should refer to the investigational product label.

Stability studies are ongoing.

15.3.5 Administration procedures

Please see section **3.2.3.**

15.3.6 Incompatibilities

No clinically relevant incompatibilities have been identified in studies performed for CYP450 interactions, anti-hypertensive medications or with other chemotherapy agents.

16 REFERENCES

1. Siegel R, Naishadham D, Jemal A. Cancer statistics, 2013. CA: a cancer journal for clinicians 2013;63:11-30.

2. Cannistra SA. Cancer of the ovary. N Engl J Med 2004;351:2519-29.

3. Tothill RW, Tinker AV, George J, et al. Novel molecular subtypes of serous and endometrioid ovarian cancer linked to clinical outcome. Clinical cancer research : an official journal of the American Association for Cancer Research 2008;14:5198-208.

4. Mellman I, Coukos G, Dranoff G. Cancer immunotherapy comes of age. Nature 2011;480:480-9.

5. Integrated genomic analyses of ovarian carcinoma. Nature 2011;474:609-15.

6. Zhang L, Conejo-Garcia JR, Katsaros D, et al. Intratumoral T cells, recurrence, and survival in epithelial ovarian cancer. N Engl J Med 2003;348:203-13.

7. Schreiber RD, Old LJ, Smyth MJ. Cancer immunoediting: integrating immunity's roles in cancer suppression and promotion. Science 2011;331:1565-70.

8. Hwang WT, Adams SF, Tahirovic E, Hagemann IS, Coukos G. Prognostic significance of tumor-infiltrating T cells in ovarian cancer: a meta-analysis. Gynecologic oncology 2012;124:192-8.

9. Leffers N, Gooden MJ, de Jong RA, et al. Prognostic significance of tumor-infiltrating T-lymphocytes in primary and metastatic lesions of advanced stage ovarian cancer. Cancer immunology, immunotherapy : CII 2009;58:449-59.

10. Facciabene A, Motz GT, Coukos G. T-regulatory cells: key players in tumor immune escape and angiogenesis. Cancer Res 2012;72:2162-71.

11. Wolf D, Wolf AM, Rumpold H, et al. The expression of the regulatory T cell-specific forkhead box transcription factor FoxP3 is associated with poor prognosis in ovarian cancer. Clinical cancer research : an official journal of the American Association for Cancer Research 2005;11:8326-31.

12. Curiel TJ, Coukos G, Zou L, et al. Specific recruitment of regulatory T cells in ovarian carcinoma fosters immune privilege and predicts reduced survival. Nat Med 2004;10:942-9.

13. Sato E, Olson SH, Ahn J, et al. Intraepithelial CD8+ tumor-infiltrating lymphocytes and a high CD8+/regulatory T cell ratio are associated with favorable prognosis in ovarian cancer. Proc Natl Acad Sci U S A 2005;102:18538-43.

14. Movahedi K, Guilliams M, Van den Bossche J, et al. Identification of discrete tumor-induced myeloid-derived suppressor cell subpopulations with distinct T cell-suppressive activity. Blood 2008;111:4233-44.

15. Toh B, Wang X, Keeble J, et al. Mesenchymal transition and dissemination of cancer cells is driven by myeloid-derived suppressor cells infiltrating the primary tumor. PLoS biology 2011;9:e1001162.

16. Yang R, Cai Z, Zhang Y, Yutzy WHt, Roby KF, Roden RB. CD80 in immune suppression by mouse ovarian carcinoma-associated Gr-1+CD11b+ myeloid cells. Cancer Res 2006;66:6807-15.

17. Keir ME, Butte MJ, Freeman GJ, Sharpe AH. PD-1 and its ligands in tolerance and immunity. Annual review of immunology 2008;26:677-704.

18. Keir ME, Francisco LM, Sharpe AH. PD-1 and its ligands in T-cell immunity. Current opinion in immunology 2007;19:309-14.

19. Dong H, Zhu G, Tamada K, Chen L. B7-H1, a third member of the B7 family, co-stimulates T-cell proliferation and interleukin-10 secretion. Nat Med 1999;5:1365-9.

20. Freeman GJ, Long AJ, Iwai Y, et al. Engagement of the PD-1 immunoinhibitory receptor by a novel B7 family member leads to negative regulation of lymphocyte activation. The Journal of experimental medicine 2000;192:1027-34.

21. Tseng SY, Otsuji M, Gorski K, et al. B7-DC, a new dendritic cell molecule with potent costimulatory properties for T cells. The Journal of experimental medicine 2001;193:839-46.

22. Latchman Y, Wood CR, Chernova T, et al. PD-L2 is a second ligand for PD-1 and inhibits T cell activation. Nature immunology 2001;2:261-8.

23. Sandhu SK, Schelman WR, Wilding G, et al. The poly(ADP-ribose) polymerase inhibitor niraparib (MK4827) in BRCA mutation carriers and patients with sporadic cancer: a phase 1 dose-escalation trial. The lancet oncology 2013;14:882-92.

24. Lin PY, Sun L, Thibodeaux SR, et al. B7-H1-dependent sex-related differences in tumor immunity and immunotherapy responses. Journal of immunology 2010;185:2747-53.

25. Maine CJ, Aziz NH, Chatterjee J, et al. Programmed death ligand-1 over-expression correlates with malignancy and contributes to immune regulation in ovarian cancer. Cancer immunology, immunotherapy : CII 2013.

26. Hamanishi J, Mandai M, Iwasaki M, et al. Programmed cell death 1 ligand 1 and tumor-infiltrating CD8+ T lymphocytes are prognostic factors of human ovarian cancer. Proc Natl Acad Sci U S A 2007;104:3360-5.

27. Brahmer JR, Tykodi SS, Chow LQ, et al. Safety and activity of anti-PD-L1 antibody in patients with advanced cancer. N Engl J Med 2012;366:2455-65.

28. Hamanishi J MM, Ikeda T, Minami M, Kawaguchi A, Matsumura N, Abiko K, Baba T, Yamaguchi K, Ueda A, Kanai M, Mori Y, Matsumoto S, Murayama T, Chikuma S, Morita S, Yokode M, Shimizu A, Honjo T, Konishi I. Efficacy and safety of anti-PD-1 antibody (Nivolumab: BMS-936558, ONO-4538) in patients with platinum-resistant ovarian cancer. J Clin Oncol 32:5s, 2014 (suppl; abstr 5511).

29. Le DT, Uram JN, Wang H, et al. PD-1 Blockade in Tumors with Mismatch-Repair Deficiency. The New England journal of medicine 2015.

30. Galluzzi L, Senovilla L, Zitvogel L, Kroemer G. The secret ally: immunostimulation by anticancer drugs. Nature reviews Drug discovery 2012;11:215-33.

31. Schoenfeld JD, Dranoff G. Anti-angiogenesis immunotherapy. Human vaccines 2011;7:976-81.

32. Kandalaft LE, Motz GT, Busch J, Coukos G. Angiogenesis and the tumor vasculature as antitumor immune modulators: the role of vascular endothelial growth factor and endothelin. Current topics in microbiology and immunology 2011;344:129-48.

33. Tartour E, Pere H, Maillere B, et al. Angiogenesis and immunity: a bidirectional link potentially relevant for the monitoring of antiangiogenic therapy and the development of novel therapeutic combination with immunotherapy. Cancer metastasis reviews 2011;30:83-95.

34. Sondak VK, Smalley KS, Kudchadkar R, Grippon S, Kirkpatrick P. Ipilimumab. Nature reviews Drug discovery 2011;10:411-2.

35. Ohm JE, Carbone DP. VEGF as a mediator of tumor-associated immunodeficiency. Immunologic research 2001;23:263-72.

36. Oyama T, Ran S, Ishida T, et al. Vascular endothelial growth factor affects dendritic cell maturation through the inhibition of nuclear factor-kappa B activation in hemopoietic progenitor cells. Journal of immunology 1998;160:1224-32.

37. Terme M, Pernot S, Marcheteau E, et al. VEGFA-VEGFR pathway blockade inhibits tumor-induced regulatory T-cell proliferation in colorectal cancer. Cancer Res 2013;73:539-49.

38. Gabrilovich DI, Ostrand-Rosenberg S, Bronte V. Coordinated regulation of myeloid cells by tumours. Nature reviews Immunology 2012;12:253-68.

39. Huang Y, Goel S, Duda DG, Fukumura D, Jain RK. Vascular normalization as an emerging strategy to enhance cancer immunotherapy. Cancer Res 2013;73:2943-8.

40. Terme M, Colussi O, Marcheteau E, Tanchot C, Tartour E, Taieb J. Modulation of immunity by antiangiogenic molecules in cancer. Clinical & developmental immunology 2012;2012:492920.

41. Amin A PE, Infante JR, Ernstoff MS. Nivolumab (anti-PD-1; BMS-936558, ONO-4538) in combination with sunitinib or pazopanib in patients (pts) with metastatic renal cell carcinoma (mRCC). J Clin Oncol 32:5s (suppl; abstr 5010) 2014.

42. Hodi FS, Lawrence D, Lezcano C, et al. Bevacizumab plus ipilimumab in patients with metastatic melanoma. Cancer immunology research 2014;2:632-42.

43. Perren TJ, Swart AM, Pfisterer J, et al. A phase 3 trial of bevacizumab in ovarian cancer. N Engl J Med 2011;365:2484-96.

44. Burger RA, Brady MF, Bookman MA, et al. Incorporation of bevacizumab in the primary treatment of ovarian cancer. N Engl J Med 2011;365:2473-83.

45. Pujade-Lauraine E, Hilpert F, Weber B, et al. Bevacizumab combined with chemotherapy for platinum-resistant recurrent ovarian cancer: The AURELIA open-label randomized phase III trial. Journal of clinical oncology : official journal of the American Society of Clinical Oncology 2014;32:1302-8.

46. Matulonis UA, Berlin S, Ivy P, et al. Cediranib, an oral inhibitor of vascular endothelial growth factor receptor kinases, is an active drug in recurrent epithelial ovarian, fallopian tube,

and peritoneal cancer. Journal of clinical oncology : official journal of the American Society of Clinical Oncology 2009;27:5601-6.

47. Ledermann JA PJ, Raja FA, et al. Randomised double-blind phase III trial of cediranib (AZD 2171) in relapsed platinum sensitive ovarian cancer: Results of the ICON6 trial. Presented at the 38th Congress of the European Society for Medical Oncology (ESMO), Abst LBA10 2013.

48. Kaye SB, Lubinski J, Matulonis U, et al. Phase II, open-label, randomized, multicenter study comparing the efficacy and safety of olaparib, a poly (ADP-ribose) polymerase inhibitor, and pegylated liposomal doxorubicin in patients with BRCA1 or BRCA2 mutations and recurrent ovarian cancer. Journal of clinical oncology : official journal of the American Society of Clinical Oncology 2012;30:372-9.

49. Wu CY, Yang LH, Yang HY, et al. Enhanced cancer radiotherapy through immunosuppressive stromal cell destruction in tumors. Clinical cancer research : an official journal of the American Association for Cancer Research 2014;20:644-57.

50. Gameiro SR, Ardiani A, Kwilas A, Hodge JW. Radiation-induced survival responses promote immunogenic modulation to enhance immunotherapy in combinatorial regimens. Oncoimmunology 2014;3:e28643.

51. Kroemer G, Galluzzi L, Kepp O, Zitvogel L. Immunogenic cell death in cancer therapy. Annual review of immunology 2013;31:51-72.

52. Snyder A, Makarov V, Merghoub T, et al. Genetic Basis for Clinical Response to CTLA-4 Blockade in Melanoma. N Engl J Med 2014.

53. Gajewski TF, Schreiber H, Fu YX. Innate and adaptive immune cells in the tumor microenvironment. Nature immunology 2013;14:1014-22.

54. O'Sullivan CC, Moon DH, Kohn EC, Lee JM. Beyond Breast and Ovarian Cancers: PARP Inhibitors for BRCA Mutation-Associated and BRCA-Like Solid Tumors. Frontiers in oncology 2014;4:42.

55. LEE JM LJ, Choyke PL, Elbuluk O. Biomarker correlates from the randomized phase 2 trial of the PARP inhibitor olaparib (O) with or without the antiangiogenic TKI cediranib (C) in recurrent platinum-sensitive ovarian cancer (NCT01116648). J Clin Oncol 32:5s (suppl; abstr 5535) 2014.

56. McAlpine JN, Porter H, Kobel M, et al. BRCA1 and BRCA2 mutations correlate with TP53 abnormalities and presence of immune cell infiltrates in ovarian high-grade serous carcinoma. Modern pathology : an official journal of the United States and Canadian Academy of Pathology, Inc 2012;25:740-50.

57. Lee JM GN, Lee MJ, Trepel J, Yu M, Kohn EC. Development of flow cytometric predictive biomarker assay for response to PARP inhibitor (PARPi) therapy in high-grade serous ovarian cancer (HGSOC). Gynecologic Oncology 2014;133.

58. Jonathan A. Ledermann PH, Charlie Gourley, Michael Friedlander, Ignace Vergote, Gordon J. S. Rustin, Clare L. Scott, Werner Meier, Ronnie Shapira-Frommer, Tamar Safra, Daniela Matei, Anitra Fielding, Euan Macpherson, Brian Dougherty, Juliane M. Jürgensmeier,

Maria Orr, Ursula Matulonis. Olaparib maintenance therapy in patients with platinum-sensitive relapsed serous ovarian cancer (SOC) and a BRCA mutation (BRCAm). Journal of clinical oncology : official journal of the American Society of Clinical Oncology 2013;31:suppl; abstr 5505.

59. Fong PC, Yap TA, Boss DS, et al. Poly(ADP)-ribose polymerase inhibition: frequent durable responses in BRCA carrier ovarian cancer correlating with platinum-free interval. Journal of clinical oncology : official journal of the American Society of Clinical Oncology 2010;28:2512-9.

60. Gelmon KA, Tischkowitz M, Mackay H, et al. Olaparib in patients with recurrent high-grade serous or poorly differentiated ovarian carcinoma or triple-negative breast cancer: a phase 2, multicentre, open-label, non-randomised study. The lancet oncology 2011;12:852-61.

61. Lee JM, Hays JL, Annunziata CM, et al. Phase I/Ib Study of Olaparib and Carboplatin in BRCA1 or BRCA2 Mutation-Associated Breast or Ovarian Cancer With Biomarker Analyses. Journal of the National Cancer Institute 2014;106.

62. Liu JF, Barry WT, Birrer M, et al. Combination cediranib and olaparib versus olaparib alone for women with recurrent platinum-sensitive ovarian cancer: a randomised phase 2 study. The lancet oncology 2014;15:1207-14.

63. Economopoulou M, Langer HF, Celeste A, et al. Histone H2AX is integral to hypoxia-driven neovascularization. Nat Med 2009;15:553-8.

64. Tentori L, Lacal PM, Muzi A, et al. Poly(ADP-ribose) polymerase (PARP) inhibition or PARP-1 gene deletion reduces angiogenesis. Eur J Cancer 2007;43:2124-33.

65. Kumareswaran R, Ludkovski O, Meng A, Sykes J, Pintilie M, Bristow RG. Chronic hypoxia compromises repair of DNA double-strand breaks to drive genetic instability. Journal of cell science 2012;125:189-99.

66. Adotevi O, Pere H, Ravel P, et al. A decrease of regulatory T cells correlates with overall survival after sunitinib-based antiangiogenic therapy in metastatic renal cancer patients. J Immunother 2010;33:991-8.

67. Finke JH, Rini B, Ireland J, et al. Sunitinib reverses type-1 immune suppression and decreases T-regulatory cells in renal cell carcinoma patients. Clinical cancer research : an official journal of the American Association for Cancer Research 2008;14:6674-82.

68. Desar IM, Jacobs JH, Hulsbergen-vandeKaa CA, et al. Sorafenib reduces the percentage of tumour infiltrating regulatory T cells in renal cell carcinoma patients. International journal of cancer Journal international du cancer 2011;129:507-12.

69. Xin H, Zhang C, Herrmann A, Du Y, Figlin R, Yu H. Sunitinib inhibition of Stat3 induces renal cell carcinoma tumor cell apoptosis and reduces immunosuppressive cells. Cancer Res 2009;69:2506-13.

70. Lesterhuis WJ, Punt CJ, Hato SV, et al. Platinum-based drugs disrupt STAT6-mediated suppression of immune responses against cancer in humans and mice. The Journal of clinical investigation 2011;121:3100-8.

71. SEER Cancer Statistics Factsheets: Lung and Bronchus Cancer. National Cancer Institute. Bethesda M, http://seer.cancer.gov/statfacts/html/lungb.html. Last accessed February 12, 2015. .

72. Blumenthal GM, Karuri SW, Zhang H, et al. Overall response rate, progression-free survival, and overall survival with targeted and standard therapies in advanced non-small-cell lung cancer: US Food and Drug Administration trial-level and patient-level analyses. Journal of clinical oncology : official journal of the American Society of Clinical Oncology 2015;33:1008-14.

73. Rajan A, Schrump DS. Precision Therapy for Lung Cancer: Tyrosine Kinase Inhibitors and Beyond. Semin Thorac Cardiovasc Surg 2015;27:36-48.

74. Anagnostou VK, Brahmer JR. Cancer immunotherapy: a future paradigm shift in the treatment of non-small cell lung cancer. Clinical cancer research : an official journal of the American Association for Cancer Research 2015;21:976-84.

75. Gettinger SN, Horn L, Gandhi L, et al. Overall Survival and Long-Term Safety of Nivolumab (Anti-Programmed Death 1 Antibody, BMS-936558, ONO-4538) in Patients With Previously Treated Advanced Non-Small-Cell Lung Cancer. Journal of clinical oncology : official journal of the American Society of Clinical Oncology 2015;33:2004-12.

76. Garon EB, Rizvi NA, Hui R, et al. Pembrolizumab for the treatment of non-small-cell lung cancer. N Engl J Med 2015;372:2018-28.

77. Taube JM, Klein A, Brahmer JR, et al. Association of PD-1, PD-1 ligands, and other features of the tumor immune microenvironment with response to anti-PD-1 therapy. Clinical cancer research : an official journal of the American Association for Cancer Research 2014;20:5064-74.

78. Champiat S, Ferte C, Lebel-Binay S, Eggermont A, Soria JC. Exomics and immunogenics: Bridging mutational load and immune checkpoints efficacy. Oncoimmunology 2014;3:e27817.

79. Birkelbach M, Ferraiolo N, Gheorghiu L, et al. Detection of impaired homologous recombination repair in NSCLC cells and tissues. Journal of thoracic oncology : official publication of the International Association for the Study of Lung Cancer 2013;8:279-86.

80. Spigel DR. PARP inhibitors in lung cancer. Journal of thoracic oncology : official publication of the International Association for the Study of Lung Cancer 2012;7:S392-3.

81. Liang W, Wu X, Hong S, et al. Multi-targeted antiangiogenic tyrosine kinase inhibitors in advanced non-small cell lung cancer: meta-analyses of 20 randomized controlled trials and subgroup analyses. PLoS One 2014;9:e109757.

82. Herbst RS, Sun Y, Eberhardt WE, et al. Vandetanib plus docetaxel versus docetaxel as second-line treatment for patients with advanced non-small-cell lung cancer (ZODIAC): a double-blind, randomised, phase 3 trial. The Lancet Oncology 2010;11:619-26.

83. van Meerbeeck JP, Fennell DA, De Ruysscher DK. Small-cell lung cancer. Lancet 2011;378:1741-55.

84. Gandhi L, Johnson BE. Paraneoplastic syndromes associated with small cell lung cancer. Journal of the National Comprehensive Cancer Network : JNCCN 2006;4:631-8.

85. Fischer JR, Darjes H, Lahm H, Schindel M, Drings P, Krammer PH. Constitutive secretion of bioactive transforming growth factor beta 1 by small cell lung cancer cell lines. Eur J Cancer 1994;30A:2125-9.

86. Singal DP, Ye M, Qiu X. Molecular basis for lack of expression of HLA class I antigens in human small-cell lung carcinoma cell lines. International journal of cancer Journal international du cancer 1996;68:629-36.

87. Schultheis AM, Scheel AH, Ozretic L, et al. PD-L1 expression in small cell neuroendocrine carcinomas. Eur J Cancer 2015;51:421-6.

88. Ishii H, Azuma K, Kawahara A, et al. Significance of programmed cell death-ligand 1 expression and its association with survival in patients with small cell lung cancer. Journal of thoracic oncology : official publication of the International Association for the Study of Lung Cancer 2015;10:426-30.

89. Ott PA et al. Pembrolizumab (MK-3475) in patients (pts) with extensive-stage small cell lung cancer (SCLC): Preliminary safety and efficacy results from KEYNOTE-028. J Clin Oncol 33, 2015 (suppl; abstr 7502). 2015.

90. Antonia SJ, et al. Phase I/II study of nivolumab with or without ipilimumab for treatment of recurrent small cell lung cancer (SCLC): CA209-032. J Clin Oncol 33, 2015 (suppl; abstr 7503). 2015.

91. Rizvi NA, Hellmann MD, Snyder A, et al. Cancer immunology. Mutational landscape determines sensitivity to PD-1 blockade in non-small cell lung cancer. Science 2015;348:124-8.

92. Byers LA, Wang J, Nilsson MB, et al. Proteomic profiling identifies dysregulated pathways in small cell lung cancer and novel therapeutic targets including PARP1. Cancer discovery 2012;2:798-811.

93. al WZe. Wainberg ZA, Rafii S, Ramanathan RK,Mina LA, Byers LA, Chugh R, et al. Safety and antitumor activity of the PARP inhibitor BMN673 in a phase 1 trial recruiting metastatic small-cell lung cancer (SCLC) and germline BRCA-mutation carrier cancer patients. J Clin Oncol 32:5s, 2014 (suppl; abstr 7522). . 2014.

94. Cardnell RJ, Feng Y, Diao LX, et al. Proteomic Markers of DNA Repair and PI3K Pathway Activation Predict Response to the PARP Inhibitor BMN 673 in Small Cell Lung Cancer. Clinical Cancer Research 2013;19:6322-8.

95. Siegel RL, Miller KD, Jemal A. Cancer statistics, 2015. CA: A Cancer Journal for Clinicians 2015;65:5-29.

96. Eisenberger MA, Blumenstein BA, Crawford ED, et al. Bilateral orchiectomy with or without flutamide for metastatic prostate cancer. N Engl J Med 1998;339:1036-42.

97. Scher HI, Halabi S, Tannock I, et al. Design and end points of clinical trials for patients with progressive prostate cancer and castrate levels of testosterone: recommendations of the Prostate Cancer Clinical Trials Working Group. J Clin Oncol 2008;26:1148-59.

98. Tannock IF, de Wit R, Berry WR, et al. Docetaxel plus prednisone or mitoxantrone plus prednisone for advanced prostate cancer. N Engl J Med 2004;351:1502-12.

99. Petrylak DP, Tangen CM, Hussain MH, et al. Docetaxel and estramustine compared with mitoxantrone and prednisone for advanced refractory prostate cancer. N Engl J Med 2004;351:1513-20.

100. de Bono JS, Logothetis CJ, Molina A, et al. Abiraterone and increased survival in metastatic prostate cancer. N Engl J Med 2011;364:1995-2005.

101. Ryan CJ, Smith MR, de Bono JS, et al. Abiraterone in metastatic prostate cancer without previous chemotherapy. N Engl J Med 2013;368:138-48.

102. Beer TM, Armstrong AJ, Rathkopf DE, et al. Enzalutamide in metastatic prostate cancer before chemotherapy. N Engl J Med 2014;371:424-33.

103. Scher HI, Fizazi K, Saad F, et al. Increased survival with enzalutamide in prostate cancer after chemotherapy. N Engl J Med 2012;367:1187-97.

104. Robinson D, Van Allen EM, Wu YM, et al. Integrative clinical genomics of advanced prostate cancer. Cell 2015;161:1215-28.

105. Schiewer MJ, Knudsen KE. Transcriptional Roles of PARP1 in Cancer. Molecular Cancer Research 2014;12:1069-80.

106. Feng Felix Y, de Bono Johann S, Rubin Mark A, Knudsen Karen E. Chromatin to Clinic: The Molecular Rationale for PARP1 Inhibitor Function. Molecular Cell 2015;58:925-34.

107. Gilardini Montani M, Prodosmo A, Stagni V, et al. ATM-depletion in breast cancer cells confers sensitivity to PARP inhibition. Journal of Experimental & Clinical Cancer Research 2013;32:95.

108. Guo R, Wang Y, Yan J, Yan H. Recurrence quantification analysis on pulse morphological changes in patients with coronary heart disease. J Tradit Chin Med 2012;32:571-7.

109. DNA repair defects and antitumor activity with PARP inhibition: TOPARP, a phase II trial of olaparib in metastatic castration resistant prostate cancer April 2015. at http://www.abstractsonline.com/Plan/ViewAbstract.aspx?sKey=d60a28c6-91a3-4a37-a419-d79f96801b30&cKey=f119b58a-3f6f-4c22-93ef-a25da5df64a0.)

110. AACR 2015: Subgroup of Men With Metastatic Prostate Cancer Respond to Olaparib Treatment The ASCO Post, 2015. at http://www.ascopost.com/ViewNews.aspx?nid=26597.)

111. Gulley J, Arlen P, Madan R, et al. Immunologic and prognostic factors associated with overall survival employing a poxviral-based PSA vaccine in metastatic castrate-resistant prostate cancer. Cancer Immunology, Immunotherapy 2010;59:663-74.

112. Kantoff PW, Higano CS, Shore ND, et al. Sipuleucel-T Immunotherapy for Castration-Resistant Prostate Cancer. New England Journal of Medicine 2010;363:411-22.

113. Barach YS, Lee JS, Zang X. T cell coinhibition in prostate cancer: new immune evasion pathways and emerging therapeutics. Trends in Molecular Medicine 2011;17:47-55.

114. Ebelt K, Babaryka G, Frankenberger B, et al. Prostate cancer lesions are surrounded by FOXP3+, PD-1+ and B7-H1+ lymphocyte clusters. European Journal of Cancer 2009;45:1664-72.

115. Bishop JL, Sio A, Angeles A, et al. PD-L1 is highly expressed in Enzalutamide resistant prostate cancer2014.

116. Sambucci M, Laudisi F, Novelli F, Bennici E, Rosado MM, Pioli C. Effects of PARP-1 Deficiency on Th1 and Th2 Cell Differentiation. The Scientific World Journal 2013;2013:8.

117. Rosado MM, Bennici E, Novelli F, Pioli C. Beyond DNA repair, the immunological role of PARP-1 and its siblings. Immunology 2013;139:428-37.

118. Nasta F, Laudisi F, Sambucci M, Rosado MM, Pioli C. Increased Foxp3+ Regulatory T Cells in Poly(ADP-Ribose) Polymerase-1 Deficiency. The Journal of Immunology 2010;184:3470-7.

119. Brenton JD, Carey LA, Ahmed AA, Caldas C. Molecular classification and molecular forecasting of breast cancer: Ready for clinical application? Journal of Clinical Oncology 2005;23:7350-60.

120. Bauer KR, Brown M, Cress RD, Parise CA, Caggiano V. Descriptive analysis of estrogen receptor (ER)negative, progesterone receptor (PR)-negative, and HER2-negative invasive breast cancer, the so-called triple-negative phenotype - A population-based study from the California Cancer Registry. Cancer 2007;109:1721-8.

121. Tutt A, Ellis P, Kilburn L, et al. The TNT trial: A randomized phase III trial of carboplatin (C) compared with docetaxel (D) for patients with metastatic or recurrent locally advanced triple negative or BRCA1/2 breast cancer (CRUK/07/012). Cancer Research 2015;75:2.

122. Nanda R, Chow LQ, Dees EC, et al. A phase Ib study of pembrolizumab (MK-3475) in patients with advanced triple-negative breast cancer. Cancer Research 2015;75.

123. Emens LA, Braiteh FS, Cassier P, et al. Inhibition of PD-L1 by MPDL3280A leads to clinical activity in patients with metastatic triple-negative breast cancer. Cancer Research 2015;75.

124. Patel SP, Kurzrock R. PD-L1 Expression as a Predictive Biomarker in Cancer Immunotherapy. Molecular Cancer Therapeutics 2015;14:847-56.

125. Domagala P, Jakubowska A, Jaworska-Bieniek K, et al. Prevalence of Germline Mutations in Genes Engaged in DNA Damage Repair by Homologous Recombination in Patients with Triple-Negative and Hereditary Non-Triple-Negative Breast Cancers. Plos One 2015;10.

126. Kiiski JI, Pelttari LM, Khan S, et al. Exome sequencing identifies FANCM as a susceptibility gene for triple-negative breast cancer. Proceedings of the National Academy of Sciences of the United States of America 2014;111:15172-7.

127. Wong-Brown MW, Avery-Kiejda KA, Bowden NA, Scott RJ. Low prevalence of germline PALB2 mutations in Australian triple-negative breast cancer. International Journal of Cancer 2014;134:301-5.

128. Pern F, Bogdanova N, Schurmann P, et al. Mutation Analysis of BRCA1, BRCA2, PALB2 and BRD7 in a Hospital-Based Series of German Patients with Triple-Negative Breast Cancer. Plos One 2012;7.

129. Watkins J, Weekes D, Shah V, et al. Genomic Complexity Profiling Reveals That HORMAD1 Overexpression Contributes to Homologous Recombination Deficiency in Triple-Negative Breast Cancers. Cancer Discovery 2015;5:488-505.

130. Zhu X, Shan L, Wang F, et al. Hypermethylation of BRCA1 gene: implication for prognostic biomarker and therapeutic target in sporadic primary triple-negative breast cancer. Breast Cancer Research and Treatment 2015;150:479-86.

131. Tan XH, Peng J, Fu YB, et al. miR-638 mediated regulation of BRCA1 affects DNA repair and sensitivity to UV and cisplatin in triple-negative breast cancer. Breast Cancer Research 2014;16.

132. Garcia AI, Buisson M, Bertrand P, et al. Down-regulation of BRCA1 expression by miR-146a and miR-146b-5p in triple negative sporadic breast cancers. Embo Molecular Medicine 2011;3:279-90.

133. Ribeiro E, Ganzinelli M, Andreis D, et al. Triple Negative Breast Cancers Have a Reduced Expression of DNA Repair Genes. Plos One 2013;8.

134. Fumagalli C, Pruneri G, Possanzini P, et al. Methylation of O-6-methylguanine-DNA methyltransferase (MGMT) promoter gene in triple-negative breast cancer patients. Breast Cancer Research and Treatment 2012;134:131-7.

135. McCabe N, Turner NC, Lord CJ, et al. Deficiency in the repair of DNA damage by homologous recombination and sensitivity to poly(ADP-ribose) polymerase inhibition. Cancer Research 2006;66:8109-15.

136. Fong PC, Boss DS, Yap TA, et al. Inhibition of Poly(ADP-Ribose) Polymerase in Tumors from BRCA Mutation Carriers. New England Journal of Medicine 2009;361:123-34.

137. Tutt A, Robson M, Garber JE, et al. Oral poly(ADP-ribose) polymerase inhibitor olaparib in patients with BRCA1 or BRCA2 mutations and advanced breast cancer: a proof-of-concept trial. Lancet 2010;376:235-44.

138. Dent RA, Lindeman GJ, Clemons M, et al. Phase I trial of the oral PARP inhibitor olaparib in combination with paclitaxel for first-or second-line treatment of patients with metastatic triple-negative breast cancer. Breast Cancer Research 2013;15.

139. Gelmon KA, Tischkowitz M, Mackay H, et al. Olaparib in patients with recurrent high-grade serous or poorly differentiated ovarian carcinoma or triple-negative breast cancer: a phase 2, multicentre, open-label, non-randomised study. Lancet Oncology 2011;12:852-61.

140. Lee J-M, Hays JL, Annunziata CM, et al. Phase I/Ib Study of Olaparib and Carboplatin in BRCA1 or BRCA2 Mutation-Associated Breast or Ovarian Cancer With Biomarker Analyses. Jnci-Journal of the National Cancer Institute 2014;106.

141. Mlecnik B, Tosolini M, Kirilovsky A, et al. Histopathologic-based prognostic factors of colorectal cancers are associated with the state of the local immune reaction. Journal of clinical oncology : official journal of the American Society of Clinical Oncology 2011;29:610-8.

142. Pages F, Kirilovsky A, Mlecnik B, et al. In situ cytotoxic and memory T cells predict outcome in patients with early-stage colorectal cancer. Journal of clinical oncology : official journal of the American Society of Clinical Oncology 2009;27:5944-51.

143. Gabrilovich D. Mechanisms and functional significance of tumour-induced dendritic-cell defects. Nat Rev Immunol 2004;4:941-52.

144. Gabrilovich DI, Chen HL, Girgis KR, et al. Production of vascular endothelial growth factor by human tumors inhibits the functional maturation of dendritic cells. Nature medicine 1996;2:1096-103.

145. Voron T, Colussi O, Marcheteau E, et al. VEGF-A modulates expression of inhibitory checkpoints on CD8+ T cells in tumors. The Journal of experimental medicine 2015;212:139-48.

146. Cunningham D, Wong RP, D'Haens G, et al. Cediranib with mFOLFOX6 vs bevacizumab with mFOLFOX6 in previously treated metastatic colorectal cancer. British journal of cancer 2013;108:493-502.

147. Grosso J, Horak CE, Inzunza D. Association of tumor PD-L1 expression and immune biomarkers with clinical activity in patients (pts) with advanced solid tumors treated with nivolumab (anti-PD-1; BMS-936558; ONO-4538). J Clin Oncol 31, 2013 (suppl; abstr 3016).

148. Rizvi NA GE, Patnaik A. Safety and clinical activity of MK-3475 as initial therapy in patients with advanced non-small cell lung cancer (NSCLC). J Clin Oncol 32:5s, 2014 (suppl; abstr 8007).

149. Zhang S, Shi R, Li C, Parivar K, Wang DD. Fixed dosing versus body size-based dosing of therapeutic peptides and proteins in adults. J Clin Pharmacol 2012;52:18-28.

150. Wang DD, Zhang S, Zhao H, Men AY, Parivar K. Fixed dosing versus body size-based dosing of monoclonal antibodies in adult clinical trials. J Clin Pharmacol 2009;49:1012-24.

151. Ng CM, Lum BL, Gimenez V, Kelsey S, Allison D. Rationale for fixed dosing of pertuzumab in cancer patients based on population pharmacokinetic analysis. Pharm Res 2006;23:1275-84.

152. Narwal R, Roskos LK, Robbie GJ. Population pharmacokinetics of sifalimumab, an investigational anti-interferon-alpha monoclonal antibody, in systemic lupus erythematosus. Clin Pharmacokinet 2013;52:1017-27.

153. George J, Alsop K, Etemadmoghadam D, et al. Nonequivalent gene expression and copy number alterations in high-grade serous ovarian cancers with BRCA1 and BRCA2 mutations. Clinical cancer research : an official journal of the American Association for Cancer Research 2013;19:3474-84.

154. Ascierto PA, Kalos M, Schaer DA, Callahan MK, Wolchok JD. Biomarkers for immunostimulatory monoclonal antibodies in combination strategies for melanoma and other

tumor types. Clinical cancer research : an official journal of the American Association for Cancer Research 2013;19:1009-20.

155. Suntharalingam G, Perry MR, Ward S, et al. Cytokine storm in a phase 1 trial of the anti-CD28 monoclonal antibody TGN1412. N Engl J Med 2006;355:1018-28.

156. Annunziata CM, Stavnes HT, Kleinberg L, et al. Nuclear factor kappaB transcription factors are coexpressed and convey a poor outcome in ovarian cancer. Cancer 2010;116:3276-84.

157. Hernandez L, Hsu SC, Davidson B, Birrer MJ, Kohn EC, Annunziata CM. Activation of NF-kappaB signaling by inhibitor of NF-kappaB kinase beta increases aggressiveness of ovarian cancer. Cancer Res 2010;70:4005-14.

158. Hsu S, Kim M, Hernandez L, et al. IKK-epsilon coordinates invasion and metastasis of ovarian cancer. Cancer Res 2012;72:5494-504.

159. Lee JM, Cimino-Mathews A, Peer CJ, et al. Safety and Clinical Activity of the Programmed Death-Ligand 1 Inhibitor Durvalumab in Combination With Poly (ADP-Ribose) Polymerase Inhibitor Olaparib or Vascular Endothelial Growth Factor Receptor 1-3 Inhibitor Cediranib in Women's Cancers: A Dose-Escalation, Phase I Study. Journal of clinical oncology : official journal of the American Society of Clinical Oncology 2017;35:2193-202.

160. Weber JS, Kahler KC, Hauschild A. Management of immune-related adverse events and kinetics of response with ipilimumab. Journal of clinical oncology : official journal of the American Society of Clinical Oncology 2012;30:2691-7.

161. Daud A HO, Ribas A, Hodi FS, Hwu WJ, Kefford R, Wolchok J, Hersey P, Weber JS, Joseph R, Gangadhar TC, Dronca RS, Patnaik A, Zarour H, Joshua AM, Gergich K, Wu D, Lunceford JK, Emancipator K, Dolled-Filhart M, Li N, Ebbinghaus S, Kang SP, Robert C Antitumor activity of the anti-PD-1 monoclonal antibody MK-3475 in melanoma(MEL): Correlation of tumor PD-L1 expression with outcome AACR (Abst CT104) 2014.

162. Snyder A, Makarov V, Merghoub T, et al. Genetic basis for clinical response to CTLA-4 blockade in melanoma. The New England journal of medicine 2014;371:2189-99.

163. van Rooij N, van Buuren MM, Philips D, et al. Tumor exome analysis reveals neoantigen-specific T-cell reactivity in an ipilimumab-responsive melanoma. Journal of clinical oncology : official journal of the American Society of Clinical Oncology 2013;31:e439-42.

164. Heemskerk B, Kvistborg P, Schumacher TN. The cancer antigenome. The EMBO journal 2013;32:194-203.

165. Mantia-Smaldone G, Ronner L, Blair A, et al. The immunomodulatory effects of pegylated liposomal doxorubicin are amplified in BRCA1- deficient ovarian tumors and can be exploited to improve treatment response in a mouse model. Gynecologic oncology 2014.

166. Pecot CV, Bischoff FZ, Mayer JA, et al. A novel platform for detection of CK+ and CK-CTCs. Cancer discovery 2011;1:580-6.

167. Scher HI, Halabi S, Tannock I, et al. Design and End Points of Clinical Trials for Patients With Progressive Prostate Cancer and Castrate Levels of Testosterone: Recommendations of the Prostate Cancer Clinical Trials Working Group. Journal of Clinical Oncology 2008;26:1148-59.

168. Eisenhauer EA, Therasse P, Bogaerts J, et al. New response evaluation criteria in solid tumours: Revised RECIST guideline (version 1.1). European Journal of Cancer 2009;45:228-47.

169. Simon R. Optimal two-stage designs for phase II clinical trials. Controlled clinical trials 1989;10:1-10.

170. Simon R. OPTIMAL 2-STAGE DESIGNS FOR PHASE-II CLINICAL-TRIALS. Controlled Clinical Trials 1989;10:1-10.

17 APPENDICES

17.1 APPENDIX A: PERFORMANCE STATUS CRITERIA

**ECOG Performance Status Scale**		**Karnofsky Performance Scale**	
Grade	Descriptions	Percent	Description
0	Normal activity. Fully active, able to carry on all pre-disease performance without restriction.	100	Normal, no complaints, no evidence of disease.
		90	Able to carry on normal activity; minor signs or symptoms of disease.
1	Symptoms, but ambulatory. Restricted in physically strenuous activity, but ambulatory and able to carry out work of a light or sedentary nature (e.g., light housework, office work).	80	Normal activity with effort; some signs or symptoms of disease.
		70	Cares for self, unable to carry on normal activity or to do active work.
2	In bed <50% of the time. Ambulatory and capable of all self-care, but unable to carry out any work activities. Up and about more than 50% of waking hours.	60	Requires occasional assistance, but is able to care for most of his/her needs.
		50	Requires considerable assistance and frequent medical care.
3	In bed >50% of the time. Capable of only limited self-care, confined to bed or chair more than 50% of waking hours.	40	Disabled, requires special care and assistance.
		30	Severely disabled, hospitalization indicated. Death not imminent.
4	100% bedridden. Completely disabled. Cannot carry on any self-care. Totally confined to bed or chair.	20	Very sick, hospitalization indicated. Death not imminent.
		10	Moribund, fatal processes progressing rapidly.
5	Dead.	0	Dead.

17.2 Appendix B: Inhibitors and Inducers of CYP3A4

CYP3A4 INDUCERS (PROHIBITED)

Armodafenil[1]	Modafinil[2]	Primidone[1]
Barbiturates[2]	Nafcillin[1]	Rifabutin
Bosentan[1]	Nevirapine	Rifampin
Carbamazepine	Oxcarbazepine	Rifapentine[1]
Dexamethasone[1]	Pentobarbital[1]	St. John's wort[2]
Efavirenz	Phenobarbital	Troglitazone[3]
Fosphenytoin[1]	Phenytoin	
Glucocorticoids[2] (see note)	Pioglitazone[2]	

Note: Topical steroids are permitted. Please contact overall PI if systemic steroids are clinically indicated while on trial.

1 Cited in Cytochrome P450 Enzymes: Substrates, Inhibitors, and Inducers. In: Lacy CF, Armstrong LL, Goldman MP, Lance LL, eds. Drug Information Handbook 20th ed. Hudson, OH; LexiComp Inc. 2011-2012: 1810-1818

2 Cited in Flockhart DA. Drug Interactions: Cytochrome P450 Drug Interaction Table. Indiana University School of Medicine (2007). http://medicine.iupui.edu/clinpharm/ddis/table.asp. Accessed Nov 2011.

3 Weak inhibitor per Lacy et al. May be used with caution.

Note: Drugs without a superscript are cited in both the Lacy and Flockhart references.

CYP3A4 INHIBITORS

**Strong Inhibitors** (prohibited)	**Moderate Inhibitors** (use with caution, avoid if possible)	**Weak Inhibitors** (use with caution, avoid if possible)
Amprenavir[1]	Amiodarone[1]	Chloramphenicol[2]
Atazanavir[1]	Aprepitant	Ciprofloxacin[2]
Clarithromycin	Cimetidine[1]	Diethyldithiocarbamate[2]
Conivaptan[1]	Clotrimazole[1]	Fluvoxamine[2]
Delavirdine[1]	Cyclosporine[1]	Gestodene[2]
Fosamprenavir[1]	Desipramine[1]	Mibefradil[2]
Fospropofol[1]	Doxycycline[1]	Mifepristone
Imatinib[1]	Efavirenz[1]	Norfluoxetine[2]
Indinavir	Erythromycin	Star fruit[2]
Isoniazid[1]	Fluconazole	Troleandomycin[2]
Itraconazole	Fosaprepitant[1]	

Ketoconazole	Grapefruit juice	
Miconazole[1]	Haloperidol[1]	
Nefazodone	Lidocaine[1]	
Nelfinavir	Metronidazole[1]	
Nicardipine[1]	Norfloxacin[1]	
Posaconazole[1]	Sertraline[1]	
Propofol[1]	Tetracycline[1]	
Quinidine[1]	Verapamil	
Ritonavir	Voriconazole[1]	
Saquinavir[2]		
Telithromycin		

1 Cited in Cytochrome P450 Enzymes: Substrates, Inhibitors, and Inducers. In: Lacy CF, Armstrong LL, Goldman MP, Lance LL, eds. Drug Information Handbook 20th ed. Hudson, OH; LexiComp Inc. 2011-2012: 1810-1818

2 Cited in Flockhart DA. Drug Interactions: Cytochrome P450 Drug Interaction Table. Indiana University School of Medicine (2007). http://medicine.iupui.edu/clinpharm/ddis/table.asp. Accessed Nov 2011.

Note: Drugs without a superscript are cited in both the Lacy and Flockhart references.

CYP3A4 Substrates (allowed – take note of possible interactions)

Alfentanil	Dexlansoprazole	Isradipine	Ranolazine
Alfuzosin	Dextromethorphan[2]	Itraconazole	Rifabutin
Alprazolam	Diazepam	Ixabepilone	Repaglinide
Ambrisentan	Dihydroergotamine	Ketamine	Risperidone[2]
Amiodarone	Diltiazem	Ketoconazole	Ritonavir
Amlodipine	Disopyramide	Lansoprazole	Salmeterol
Aprepitant	Docetaxel	Lapatinib	Saquinavir
Aripiprazole	Domperidone[2]	Lercanidipine[2]	Sibutramine
Armodafinil	Doxorubicin	Levonorgestrel	Sildenafil
Astemizole[2]	Eletriptan	Lidocaine	Simvastatin
Atazanavir	Efavirenz	Lovastatin[2]	Sirolimus
Atorvastatin	Eplerenone	Lopinavir	Solifenacin
Benzphetamine	Ergoloid mesylates	Maraviroc	Spiramycin
Bisoprolol	Ergonovine	Medroxyprogesterone	Sufentanil
Bortezomib	Ergotamine	Mefloquine	Sunitinib
Bosentan	Erlotinib	Mestranol	Tacrolimus

CYP3A4 Substrates (allowed – take note of possible interactions)

Bromazepam	Erythromycin	Methadone	Tadalafil
Bromocriptine	Escitalopram	Methylergonovine	Tamoxifen
Budesonide	Esomeprazole	Methylprednisolone	Tamsulosin
Buprenorphine	Estradiol	Miconazole	Temsirolimus
Buspirone	Estrogens, conjugated synthetic	Midazolam	Telithromycin
Busulfan	Estrogens, conjugated equine	Mirtazapine	Teniposide
Cafergot[2]	Estrogens, esterified	Modafinil	Terfenadine[2]
Caffeine[2]	Estropipate	Montelukast	Testosterone[2]
Carbamazepine	Eszopiclone	Nateglinide	Tetracycline
Cerivastatin[2]	Ethinyl estradiol	Nefazodone	Theophylline
Chlordiazepoxide	Ethosuximide	Nelfinavir	Tiagabine
Chloroquine	Etoposide	Nevirapine	Ticlopidine
Chlorpheniramine	Exemestane	Nicardipine	Tinidazole
Ciclesonide	Felbamate	Nifedipine	Tipranavir
Cilostazol	Felodipine	Nilotinib	Tolterodine
Cisapride	Fentanyl	Nimodipine	Toremifene
Citalopram	Finasteride[2]	Nitrendipine[2]	Tramadol
Clarithromycin	Flunisolide	Nisoldipine	Trazodone
Clobazam	Flurazepam	Norethindrone	Triazolam
Clonazepam	Flutamide	Norgestrel	Trimethoprim
Clorazepate	Fluticasone	Omeprazole	Trimipramine
Cocaine	Fosamprenavir	Ondansetron	Vardenafil
Codeine[2]	Fosaprepitant	Paclitaxel	Venlafaxine
Colchicine	Gefitinib	Paricalcitol	Verapamil
Conivaptan	Haloperidol	Pazopanib	Vinblastine
Cyclophosphamide	Hydrocortisone[2]	Pimozide	Vincristine
Cyclosporine	Ifosfamide	Primaquine	Vinorelbine
Dantrolene	Imatinib	Propranolol[2]	Zaleplon[2]
Dapsone	Indinavir	Progesterone	Ziprasidone[2]
Darifenacin	Irinotecan	Quazepam	Zolpidem
Darunavir		Quetiapine	Zonisamide
Dasatinib		Quinidine	Zopiclone

CYP3A4 Substrates (allowed – take note of possible interactions)

Delavirdine<br>Dexamethasone	Isosorbide dinitrate<br>Isosorbide mononitrate	Quinine<br>Rabeprazole	

* Note: CYP3A4 Substrate Table adapted from Cytochrome P450 Enzymes: Substrates, Inhibitors, and Inducers. In: Lacy CF, Armstrong LL, Goldman MP, Lance LL, eds. Drug Information Handbook 20th ed. Hudson, OH; LexiComp Inc. 2011-2012: 1810-1818

** Substrates with 2 superscript denote substrates cited in Flockhart DA. Drug Interactions: Cytochrome P450 Drug Interaction Table. Indiana University School of Medicine (2007). http://medicine.iupui.edu/clinpharm/ddis/table.asp. Accessed Nov 2011

17.3 APPENDIX C: ORAL MEDICATION DIARY

CEDIRANIB

Today's Date ________________________________ Cycle #__________

Patient Name_______________________________ Patient Study ID ___________________

(*initials acceptable for patient's name)*

Please bring your pill bottle and this form to your physician when you go for your next appointment.
This is required for study compliance.

INSTRUCTIONS TO THE PATIENT:

1. Complete one form for each cycle (28 days).
2. You will take ___ (number) ____ mg (dosage) tablet(s) each morning. You may take the tablet(s) one hour before or two hours after eating, as you prefer.
3. Record the date, the number of pills you took, and when you took them.
4. If you have any comments or notice any side effects, please record them in the Comments column.

Date	Day	# pills and when taken: cediranib			Comments	Date	Day	# pills and when taken: cediranib			Comments
		15 mg	20 mg	Time				15mg	20mg	Time	
	1						15				
	2						16				
	3						17				
	4						18				
	5						19				
	6						20				
	7						21				
	8						22				
	9						23				
	10						24				
	11						25				
	12						26				
	13						27				
	14						28				

Patient's Signature: ___________________________________ Date: _________________________

Study Team will complete this section:

1. Date patient started protocol treatment_____________________
2. Date patient was removed from study _____________________
3. Patient's planned daily dose______________________________
4. Total number of pills taken this month ______________________

Physician/Nurse's Signature ____

OLAPARIB

Today's Date ______________________________ Cycle #__________

Patient Name______________________________ (*initials acceptable for patient's name)*

Patient Study ID __________________

Please bring your pill bottle and this form to your physician when you go for your next appointment. ***This is required for study compliance.***

INSTRUCTIONS TO THE PATIENT:

1. Complete one form for each cycle (28 days).
2. You will take ___ tablets each day twice a day 12 hours apart. You may take the tablets with a light meal or snack.
3. Record the date, the number of tablets you took, and when you took them.
4. If you have any comments or notice any side effects, please record them in the Comments column.

Date	Day	# tablets and when taken: olaparib				Comments	Date	Day	# tablets and when taken: olaparib				Comments
		#	AM	#	PM				#	AM	#	PM	
	1							15					
	2							16					
	3							17					
	4							18					
	5							19					
	6							20					
	7							21					
	8							22					
	9							23					
	10							24					
	11							25					
	12							26					
	13							27					
	14							28					

Patient's Signature: ________________________________ Date: ____________________

Study Team will complete this section:

1. Date patient started protocol treatment____________________

2. Date patient was removed from study ______________________
3. Patient's planned daily dose______________________________
4. Total number of pills taken this month _____________________

Physician/Nurse's Signature
____

17.4 APPENDIX D: PATIENT'S BLOOD PRESSURE DIARY (DURVALUMAB+C OR DURVALUMAB+O+C ARM ONLY)

Today's Date ___________________________

Patient Name ___________________________ Patient Study ID _______________

Please bring this form to every clinic visit or appointment. ***This is required for study compliance.***

Instructions to the Patient

1. Your blood pressure readings have two numbers. The first number is the pressure in your blood vessels during a heart beat (systolic), and the second number is the pressure in the vessels when the heart rests in between beats (diastolic). These numbers are usually written with a slash in between them (for example, normal blood pressure is 120/80).
2. Record the date, then record your blood pressure twice each day using a home blood pressure monitor:
    - each morning while you are resting or reading (not while you are active: dressing, making breakfast, etc.)
    - each evening at bedtime or while you are relaxing during the evening
3. If you take your blood pressure at other times of the day, please record the numbers and time under "Other readings."
4. If your systolic pressure is greater than 140 or your diastolic blood pressure is greater than 90 twice in a row measured several hours apart, please contact your doctor's office at __________________ for instructions.

Date	AM readings	PM readings	Other readings (include time of day)	Date	AM readings	PM readings	Other readings (include time of day)
	/	/			/	/	
	/	/			/	/	
	/	/			/	/	
	/	/			/	/	
	/	/			/	/	
	/	/			/	/	
	/	/			/	/	
	/	/			/	/	
	/	/			/	/	
	/	/			/	/	
	/	/			/	/	
	/	/			/	/	
	/	/			/	/	

	/	/			/	/	

Patient's Signature: ___________________________________ Date: _________________

Study Team will complete this section
Date of visit: __________________________
Physician/Nurse's Signature: _________________________________ Date: _____________

17.5 APPENDIX E: PATIENT'S DIARRHEA DIARY (ALL ARMS)

Today's Date _____________________________

Patient Name _____________________________ Patient Study ID ________________

Please bring this form to every clinic visit or appointment. ***This is required for study compliance.***

Instructions to the Patient

- Record the date, then record the number of bowel movements you have at the end of each day.
- Please note if there is blood or mucus in the stool, or if you have any abdominal pain.
- If you have 4 or more episodes of diarrhea in one day, or notice blood, mucus, or pain, please contact your doctor's office at ______________________________ for instructions.

Date	Number of stools	Comments	Date	Number of stools	Comments

Patient's Signature: ______________________________________ Date: __________________

Study Team will complete this section

Date of visit: ___________________________

Physician/Nurse's Signature: ___________________________________ Date: _____________

17.6 APPENDIX F: SUGGESTED ANTI-HYPERTENSIVE MEDICATIONS, STARTING DOSES AND HEPATIC METABOLISM

**Agent**	**Initial Dose**	**Intermediate Dose**	**Maximum Dose**	**Hepatic Metabolism**
**Nifedipine XL***	30 mg po qd	60 mg po qd	90 mg po qd	CYP 3A4 substrate
**Amlodipine***	2.5 mg po qd	5 mg po qd	10 mg po qd<br>5 mg po qd<br>5 mg po qd	CYP 3A4 substrate
Felodipine	2.5 mg po qd	5 mg po qd	10 mg po qd	CYP 3A4 substrate + inhibitor
**Selective β blockers (BB):**				
**Agent**	**Initial Dose**	**Intermediate Dose**	**Maximum Dose**	**Hepatic Metabolism**
Metoprolol	25 mg po bid	50 mg po bid	100 mg po bid	CYP 2D6 substrate
**Atenolol***	25 mg po bid	50 mg po qd	100 mg po bid	No
Acebutolol	100 mg po bid	200mg-300 mg po bid	400 mg po bid	Yes (CYP450 unknown)
Bisoprolol	2.5 mg po qd	5-10 mg po bid	20 mg po qd	Yes (CYP450 unknown)
**Angiotensin Converting Enzyme Inhibitors (ACEIs):**				
**Agent**	**Initial Dose**	**Intermediate Dose**	**Maximum Dose**	**Hepatic Metabolism**
Captopril	12.5 po tid	25 mg po tid	50 mg po tid	CYP 2D6

				substrate
Enalapril	5 mg po qd	10-20 mg po qd	40 mg po qd	CYP 3A4 substrate
Ramipril	2.5 mg po qd	5 mg po qd	10 mg po qd	Yes (CYP450 unknown)
**Lisinopril***	5 mg po qd	10-20 mg po qd	40 mg po qd	No
Fosinopril	10 mg po qd	20 mg po qd	40 mg po qd	Yes (CYP450 unknown)
**Rarely used: Perindopril***	4mg po qd	none	8mg po qd	Yes but not CYP450
**Rarely used: Quinapril***	10mg po qd	20 mg po qd	40 mg po /qd	No
**Angiotensin II Receptor Blockers (ARB's):**				
**Agent**	**Initial Dose**	**Intermediate Dose**	**Maximum Dose**	**Hepatic Metabolism**
Losartan	25mg po qd	50 mg po qd	100 mg po qd	CYP 3A4 substrate
Candesartan	4mg po qd	8-16 mg po qd	32mg po qd	CYP 2C9 substrate
Irbesartan	75mg po qd	150 mg po qd	300 mg po qd	CYP 2C9 substrate
**Telmisartan***	40 mg po qd	none	80 mg po qd	Yes but not CYP450
**Valsartan***	80 mg po qd	none	160mg po qd	Yes but not CYP450
Alpha and beta blockers				

Agent	Initial Dose	Intermediate Dose	Maximum Dose	Hepatic Metabolism
Labetolol	100 mg po bid	200 mg po bid	400 mg po bid	CYP 2D6<br>substrate and inhibitor

*Agents in bold characters are suggested as optimal choices to avoid or minimize potential drug interactions with cediranib through CYP450.

17.7 APPENDIX G: LEE LABORATORY STANDARD OPERATING PROCEDURE-TISSUE CORE COLLECTION

Needle Biopsy–Cryopreservation in OCT

Principle:

Core needle biopsies are used to sample tissue from a specific, defined location. These biopsies may consist of normal, pre-malignant and malignant tissue due to the multi-level tissue sample that is obtained. This type of sample is ideal for studying the micro-tumor environment.
Rapid freezing of the sample is required to prevent degradation of the proteins or RNA. Optimal Cutting Temperature (OCT) compound is an alcohol polymer that is liquid at room temperature and a solid at–20 degrees C. This polymer is used to cryo-protect the tissue and provide a medium for cryo-sectioning.

Materials:

Cryomolds (Sakura Finetek Ca.t # 4728)
OCT (Sakura Finetek Cat. # 4583)
Dry ice
Ultra cold freezer (-700 to -800C)
Needle: 16 or 18 gauge
Permanent marker
Sterile forceps
Sterile Glass slides
Aluminum foil or 50ml Falcon tubes

Procedure:

1. Prepare all supplies prior to the biopsy procedure to avoid delay once the specimen has been obtained.
2. Label the handle and the front surface of the cryo mold with the sample or patient's identifying information.
3. Perform core needle biopsy.
4. Pick the core from the biopsy needle onto a sterile glass slide.
5. Fill cryomold about 1/3 full with OCT. Place the cryomold in dry ice to partially freeze the OCT. The OCT should be jelly-like, not completely frozen.

6. Carefully lift the core biopsy by both ends with sterile forceps.
Do not stretch the biopsy or it will break.
7. Lay the biopsy as straight as possible in the OCT. Once the sample touches the OCT, you cannot reposition it or the sample will break apart.
8. Quickly add OCT on top of the biopsy, completely covering the sample.
9. Ensure the sample is level and freeze immediately in dry ice.
10. Store wrapped in aluminum foil or in a 50ml Falcon tube at 70 degrees C.

Note: Do not lay the biopsy on frozen OCT and cover it with liquid OCT. The OCT will not fuse and will split into two sections when cutting the frozen tissue sections.

Frozen Section Slides:

1. Frozen sections for proteomic analysis should be cut at 5-8um on plain, uncoated glass microscope slides.
2. The tissue section should be placed as close as possible to the center of the slide. Do not place the frozen section at the end of the slide.
3. Two tissue sections from the same biopsy may be placed on the same glass slide if space permits.
4. Do not allow the tissue section to air on the slide. Freeze immediately on dry ice or at–
80 degrees C.

17.8 APPENDIX H: SOP FOR PRESERVING A NEEDLE BIOPSY TISSUE IN FORMALIN FOR PARAFFIN EMBEDDING

1. Prepare two or three Eppendorf tubes which contain 1 ml of 4% formalin and leave tubes at room temperature.

2. Quickly make your way to Interventional Radiology (IR) on the 1st floor.

   Ask one of the techs or nurses where your patient is and prepare to receive the sample.

3. Lay a paper towel next to your bucket.

4. Take out the forceps and rinse/dry them thoroughly before placing them on the paper towel.

5. Put on a mask, hair net and gloves.

6. When the radiologist is ready, take the slide with the sample on it while being **CAREFUL NOT TO TOUCH THE DOCTOR'S GLOVE.**

7. Use the forceps to gently pickup and place the sample in the Eppendorf tube.

8. Leave the Eppendorf tube at room temperature overnight.

9. Next day carefully remove 4% formalin from the Eppendorf tube and replace 1 ml of 70% ethanol. Leave the tube at room temperature.

10. Call AmericanHistolab (Tel: 301-330-1200) to pick up the sample.

11. Cost for paraffin embedding and H&E staining: $7.95/block. Catalogue Number: 1001

12. The sample labelling with a random code and barcoding should be same as OTC preserved samples collected at the same day (Appendix G).

17.9 APPENDIX I: METHOD FOR PREPARING PBMC SAMPLES FROM BLOOD FOR PHARMACODYNAMIC (PD) STUDIES

Using a BD Vacutainer CPT, draw the blood sample. Assure that the anticoagulant being used (sodium citrate or sodium heparin) is correct for the intended use of the specimen and that the tube size is correct for the blood draw volume required by the protocol.

Mix the sample by inverting the tube for 5-8 times and centrifuge the sample at 1500 g for 30min at 18-25°C, without break.

After centrifuge, remove two thirds of plasma and transfer whitish PBMC layer into the labeled 15-ml conical tube using a 3-ml Falcon transfer pipet.

Discard the Vacutainer CPT tube in biohazard waste container.

Add Plasma-Lyte A USP to the 15-ml tube to bring total volume to 14 ml, cap and mix by gentle inversion 5-8 times.

Centrifuge the sample at 430 g for 10 min at 18-25°C, without break.

Using a new transfer pipet, aspirate as much supernatant as possible without disturbing the cell pellet and discard the supernatant into biohazardous liquid waste.

Re-suspend the cell pellet in 2 ml of Plasma-Lyte A USP by gently flicking the bottom of the tube with the index finger and then gently pipet up and down a few times.

Immediately after resuspending the cell pellet, transfer 10 ul of sample into a cell count to count the number of cells.

The calculated volume required to make a suspension of 3 x 10^6 cells/ml.

Transfer 1 mL aliquots of the PBMC cell suspension into individual 1.5-ml cryovials.

Centrifuge the cryovials in Eppendorf centrifuge at 10000 rpm for 10 minutes at 4-10°C.

Remove and discard as much supernatant as possible without disturbing the cell pellet.

For viable cells storage, the cell pellet should be suspended in FBS with 10% DMSO at this stage and then stored at -80°C until use.

17.10 APPENDIX J: DURVALUMAB DOSE CALCULATIONS

Durvalumab Dosing

The Durvalumab dosing should be done depending on subject weight:

1. Cohort dose: X mg/kg

2. Subject weight: Y kg

3. Dose for subject: XY mg = X (mg/kg) × Y (kg)

4. Dose to be added into infusion bag:

Dose (mL) = XY mg / 50 (mg/mL)

where 50 mg/mL is Durvalumab nominal concentration

The corresponding volume of Durvalumab should be rounded to the nearest tenth mL (0.1 mL). Dose adjustments for each cycle only needed for greater than 10% change in weight.

5. The theoretical number of vials required for dose preparation is the next greatest whole number of vials from the following formula:

Number of vials = Dose (mL) / 10 (mL/vial)

Example:

1. Cohort dose: 10 mg/kg

2. Subject weight: 80 kg

3. Dose for subject: 800 mg = 10 (mg/kg) × 80 (kg)

4. Dose to be added into infusion bag:

Dose (mL) = 800 mg / 50 (mg/mL) = 16.0 mL

5. The theoretical number of vials required for dose preparation:

Number of vials = 16.0 (mL) / 10 (mL/vial) = 2 vials

17.11 APPENDIX K: DOSING MODIFICATION AND TOXICITY MANAGEMENT GUIDELINES (TMG) FOR IMMUNE-MEDIATED, INFUSION-RELATED, AND NON-IMMUNE–MEDIATED REACTIONS (DURVALUMAB MONOTHERAPY OR IN COMBINATION WITH OTHER PRODUCTS) VERSION 14 OCTOBER 2020 (CTCAE V5.0)

<table>
<tr><th colspan="2">General Considerations regarding Immune-Mediated Reactions</th></tr>
<tr><th>Dose Modifications</th><th>Toxicity Management</th></tr>
<tr><td>
Drug administration modifications of study drug/study regimen will be made to manage potential immune-related AEs based on severity of treatment-emergent toxicities graded per NCI CTCAE v5.0.
In addition to the criteria for permanent discontinuation of study drug/study regimen based on CTC grade/severity (table below), permanently discontinue study drug/study regimen for the following conditions:

Inability to reduce corticosteroid to a dose of ≤10 mg of prednisone per day (or equivalent) within 12 weeks of the start of the immune-mediated adverse event (imAE)
Grade 3 recurrence of a previously experienced treatment-related imAE following resumption of dosing

Grade 1 No dose modification
Grade 2 Hold study drug/study regimen dose until Grade 2 resolution toGrade ≤1.
If toxicity worsens, then treat as Grade 3 or Grade 4.
Study drug/study regimen can be resumed once event stabilizes to Grade ≤1 after completion of steroid taper.
Patients with endocrinopathies who may require prolonged or continued steroid replacement can be retreated with study drug/study regimen on the following conditions:

The event stabilizes and is controlled.
The patient is clinically stable as per Investigator or treating physician's clinical judgement.
Doses of prednisone are at ≤10 mg/day or equivalent.

Grade 3 Depending on the individual toxicity, study drug/study regimen may be permanently discontinued. Please refer to guidelines below.
</td><td>
It is recommended that management of immune-mediated adverse events (imAEs) follows the guidelines presented in this table:

It is possible that events with an inflammatory or immune mediated mechanism could occur in nearly all organs, some of them not noted specifically in these guidelines.
Whether specific immune-mediated events (and/or laboratory indicators of such events) are noted in these guidelines or not, patients should be thoroughly evaluated to rule out any alternative etiology (e.g., disease progression, concomitant medications, and infections) to a possible immune-mediated event. In the absence of a clear alternative etiology, all such events should be managed as if they were immune related. General recommendations follow.
Symptomatic and topical therapy should be considered for low-grade (Grade 1 or 2, unless otherwise specified) events.
For persistent (>3 to 5 days) low-grade (Grade 2) or severe (Grade ≥3) events, promptly start prednisone 1 to 2 mg/kg/day PO or IV equivalent.
Some events with high likelihood for morbidity and/or mortality – e.g., myocarditis, or other similar events even if they are not currently noted in the guidelines – should progress rapidly to high dose IV corticosteroids (methylprednisolone at 2 to 4 mg/kg/day) even if the event is Grade 2, and if clinical suspicion is high and/or there has been clinical confirmation. Consider, as necessary, discussing with the study physician, and promptly pursue specialist consultation.
If symptoms recur or worsen during corticosteroid tapering (28 days of taper), increase the corticosteroid dose (prednisone dose [e.g., up to 2 to 4 mg/kg/day PO or IV equivalent]) until stabilization or improvement of symptoms, then resume corticosteroid tapering at a slower rate (>28 days of taper).

</td></tr>
</table>

General Considerations regarding Immune-Mediated Reactions	
**Dose Modifications**	**Toxicity Management**
**Grade 4** Permanently discontinue study drug/study regimen.<br><br>Note: For asymptomatic amylase or lipase levels of >2X ULN, hold study drug/study regimen, and if complete work up shows no evidence of pancreatitis, study drug/study regimen may be continued or resumed.<br><br>Note: Study drug/study regimen should be permanently discontinued in Grade 3 events with high likelihood for morbidity and/or mortality – e.g., myocarditis, or other similar events even if they are not currently noted in the guidelines. Similarly, consider whether study drug/study regimen should be permanently discontinued in Grade 2 events with high likelihood for morbidity and/or mortality – e.g., myocarditis, or other similar events even if they are not currently noted in the guidelines – when they do not rapidly improve to Grade <1 upon treatment with systemic steroids and following full taper<br><br>Note: There are some exceptions to permanent discontinuation of study drug for Grade 4 events (i.e., hyperthyroidism, hypothyroidism, Type 1 diabetes mellitus).	– More potent immunosuppressives such as TNF inhibitors (e.g., infliximab; also refer to the individual sections of the imAEs for specific type of immunosuppressive) should be considered for events not responding to systemic steroids. Progression to use of more potent immunosuppressives should proceed more rapidly in events with high likelihood for morbidity and/or mortality – e.g., myocarditis, or other similar events even if they are not currently noted in the guidelines – when these events are not responding to systemic steroids.<br>– With long-term steroid and other immunosuppressive use, consider need for *Pneumocystis jirovecii* pneumonia (PJP, formerly known as *Pneumocystis carinii* pneumonia) prophylaxis, gastrointestinal protection, and glucose monitoring.<br>– Discontinuation of study drug/study regimen is not mandated for Grade 3/Grade 4 inflammatory reactions attributed to local tumor response (e.g., inflammatory reaction at sites of metastatic disease and lymph nodes). Continuation of study drug/study regimen in this situation should be based upon a benefit-risk analysis for that patient.

AE Adverse event; CTC Common Toxicity Criteria; CTCAE Common Terminology Criteria for Adverse Events; imAE immune-mediated adverse event; IV intravenous; NCI National Cancer Institute; PO By mouth.

Specific Immune-Mediated Reactions			
**Adverse Events**	**Severity Grade of the Event (NCI CTCAE version 5.0)**	**Dose Modifications**	**Toxicity Management**
**Pneumonitis/Interstitial Lung Disease (ILD)**	**Any Grade**	**General Guidance**	**For Any Grade:**<br>– Monitor patients for signs and symptoms of pneumonitis or ILD (new onset or worsening shortness of breath or cough). Patients should be evaluated with imaging and pulmonary function tests, including other diagnostic procedures as described below.<br>– Suspected pneumonitis should be confirmed with radiographic imaging and other infectious and disease-related aetiologies excluded, and managed as described below<br>– Initial work-up may include clinical evaluation, monitoring of oxygenation via pulse oximetry (resting and exertion), laboratory work-up, and high- resolution CT scan.<br>– Consider Pulmonary and Infectious Disease Consults
	**Grade 1** (asymptomatic, clinical or diagnostic observations only; intervention not indicated)	No dose modifications required. However, consider holding study drug/study regimen dose as clinically appropriate and during diagnostic work-up for other etiologies.	**For Grade 1 (radiographic changes only):**<br>– Monitor and closely follow up in 2 to 4 days for clinical symptoms, pulse oximetry (resting and exertion), and laboratory work-up and then as clinically indicated.<br>– Consider Pulmonary and Infectious Disease consults.
	**Grade 2** (symptomatic; medical intervention indicated; limiting	Hold study drug/study regimen dose until Grade 2 resolution to Grade ≤1.<br>• If toxicity worsens, then treat as Grade 3 or Grade 4.	**For Grade 2 (mild to moderate new symptoms):**<br>– Monitor symptoms daily and consider hospitalization.

Specific Immune-Mediated Reactions			
**Adverse Events**	**Severity Grade of the Event (NCI CTCAE version 5.0)**	**Dose Modifications**	**Toxicity Management**
	instrumental ADL)	• If toxicity improves to Grade ≤1, then the decision to reinitiate study drug/study regimen will be based upon treating physician's clinical judgment and after completion of steroid taper.	– Promptly start systemic steroids (e.g., prednisone 1 to 2 mg/kg/day PO or IV equivalent).<br>– Reimage as clinically indicated, consider Chest CT with contrast and repeat in 3-4 weeks.<br>– If no improvement within 2 to 3 days, additional workup should be considered and prompt treatment with IV methylprednisolone 2 to 4 mg/kg/day started<br>– If still no improvement within 2 to 3 days despite IV methylprednisolone at 2 to 4 mg/kg/day, promptly start immunosuppressive therapy such as TNF inhibitors (e.g., infliximab at 5 mg/kg IV once, may be repeated at 2 and 6 weeks after initial dose at the discretion of the treating provider). Caution: It is important to rule out sepsis and refer to infliximab label for general guidance before using infliximab.<br>– Once the patient is improving, gradually taper steroids over ≥28 days and consider prophylactic antibiotics, antifungals, or anti-PJP treatment (refer to current NCCN guidelines for treatment of cancer-related infections)[a]<br>– Consider Pulmonary and Infectious Disease consults.<br>– Consider, as necessary, discussing with study physician.
	**Grade 3 or 4**<br>(Grade 3: severe symptoms; limiting self-care	Permanently discontinue study drug/study regimen.	**For Grade 3 or 4 (severe or new symptoms, new/worsening hypoxia, life-threatening):**

**Specific Immune-Mediated Reactions**			
**Adverse Events**	**Severity Grade of the Event (NCI CTCAE version 5.0)**	**Dose Modifications**	**Toxicity Management**
	ADL; oxygen indicated)<br><br>(Grade 4: life-threatening respiratory compromise; urgent intervention indicated [e.g., tracheostomy or intubation])		– Promptly initiate empiric IV methylprednisolone 1 to 4 mg/kg/day or equivalent.<br>– Obtain Pulmonary and Infectious Disease consults; consider, as necessary, discussing with study physician.<br>– Hospitalize the patient.<br>– Supportive care (e.g., oxygen).<br>– If no improvement within 2 to 3 days, additional workup should be considered and prompt treatment with additional immunosuppressive therapy such as TNF inhibitors (e.g., infliximab at 5 mg/kg IV, may be repeated at 2 and 6 weeks after initial dose at the discretion of the treating provider) . Caution: rule out sepsis and refer to infliximab label for general guidance before using infliximab.<br>– Once the patient is improving, gradually taper steroids over ≥28 days and consider prophylactic antibiotics, antifungals, and, in particular, anti-PJP treatment (refer to current NCCN guidelines for treatment of cancer-related infections).[a]
**Diarrhea/Colitis**<br>**Large intestine perforation/Intestine perforation**	**Any Grade**	**General Guidance**	**For Any Grade:**<br>– Monitor for symptoms that may be related to diarrhea/enterocolitis (abdominal pain, cramping, or changes in bowel habits such as increased frequency over baseline or blood in stool) or related to bowel perforation

Specific Immune-Mediated Reactions			
**Adverse Events**	**Severity Grade of the Event (NCI CTCAE version 5.0)**	**Dose Modifications**	**Toxicity Management**
			(such as sepsis, peritoneal signs, and ileus).<br>- When symptoms or evaluation indicate a perforation is suspected, consult a surgeon experienced in abdominal surgery immediately without any delay.<br>- PERMANENTLY DISCONTINUE STUDY DRUG FOR ANY GRADE OF INTESTINAL PERFORATION<br>– Patients should be thoroughly evaluated to rule out any alternative etiology (e.g., disease progression, other medications, or infections), including testing for clostridium difficile toxin, etc.<br>– Steroids should be considered in the absence of clear alternative etiology, even for low-grade events, in order to prevent potential progression to higher grade event, including perforation.<br>– Use analgesics carefully; they can mask symptoms of perforation and peritonitis.
	**Grade 1**<br>(Diarrhea: stool frequency of <4 over baseline per day)<br>(Colitis: asymptomatic; clinical or diagnostic observations only; intervention not indicated)	No dose modifications.	**For Grade 1:**<br>– Monitor closely for worsening symptoms.<br>– Consider symptomatic treatment, including hydration, electrolyte replacement, dietary changes (e.g., American Dietetic Association colitis diet), loperamide and other supportive care measures.<br>– If symptoms persist consider checking lactoferrin; if positive treat as Grade 2

**Specific Immune-Mediated Reactions**			
**Adverse Events**	**Severity Grade of the Event (NCI CTCAE version 5.0)**	**Dose Modifications**	**Toxicity Management**
			below. If negative and no infection, continue Grade 1 management .
	**Grade 2**<br>(Diarrhea: stool frequency of 4 to 6 over baseline per day; limiting instrumental ADL)<br>(Colitis: abdominal pain; mucus or blood in stool)<br>(Perforation: invasive intervention not indicated)	Hold study drug/study regimen until resolution to Grade ≤1<br>• If toxicity worsens, then treat as Grade 3 or Grade 4.<br>• If toxicity improves to Grade ≤1, then study drug/study regimen can be resumed after completion of steroid taper.	**For Grade 2:**<br>– Consider symptomatic treatment, including hydration, electrolyte replacement, dietary changes (e.g., American Dietetic Association colitis diet), and loperamide and/or budesonide.<br>– Promptly start prednisone 1 to 2 mg/kg/day PO or IV equivalent.<br>– If event is not responsive within 2 to 3 days or worsens despite prednisone at 1 to 2 mg/kg/day PO or IV equivalent, GI consult should be obtained for consideration of further workup, such as imaging and/or colonoscopy, to confirm colitis and rule out perforation, and prompt treatment with IV methylprednisolone 2 to 4 mg/kg/day started.<br>– If still no improvement within 2 to 3 days despite 1 to 2 mg/kg IV methylprednisolone, promptly start immunosuppressives such as infliximab at 5 mg/kg IV, may be repeated at 2 and 6 weeks after initial dose at the discretion of the treating provider . **Caution:** it is important to rule out bowel perforation and refer to infliximab label for general guidance before using infliximab.<br>– Consider, as necessary, discussing with study physician if no resolution to Grade ≤1 in 3 to 4 days.<br>– Once the patient is improving, gradually taper steroids over ≥28 days

Specific Immune-Mediated Reactions			
**Adverse Events**	**Severity Grade of the Event (NCI CTCAE version 5.0)**	**Dose Modifications**	**Toxicity Management**
			and consider prophylactic antibiotics, antifungals, and anti-PJP treatment (refer to current NCCN guidelines for treatment of cancer-related infections).[a]
	**Grade 3 or 4**<br>(Grade 3 Diarrhea: stool frequency of ≥7 over baseline per day; limiting self care ADL;<br>Grade 4 Diarrhea: life threatening consequences)<br>(Grade 3 Colitis: severe abdominal pain, fever; ileus; peritoneal signs;<br>Grade 4 Colitis: life-threatening consequences, urgent intervention indicated)<br>(Grade 3 Perforation: invasive intervention indicated;<br>Grade 4 Perforation: life-threatening consequences; urgent intervention indicated)	**Grade 3**<br>- For patients treated with PD-1 or PDL-1 inhibitors, hold study drug/study regimen until resolution to Grade ≤1; study drug/study regimen can be resumed after completion of steroid taper. Permanently discontinue study drug/study regimen for Grade 3 if toxicity does not improve to Grade ≤1 within 14 days.<br>- Permanently discontinue study drug for 1) Grade 3 colitis in patients treated with CTLA-4 inhibitors or 2) Any grade large intestine perforation/Intestinal perforation in any patient treated with ICI.<br><br>**Grade 4**<br>Permanently discontinue study drug/study regimen.	**For Grade 3 or 4:**<br>– Promptly initiate empiric IV methylprednisolone 1 to 2 mg/kg/day or equivalent.<br>– Monitor stool frequency and volume and maintain hydration.<br>– Urgent GI consult and imaging and/or colonoscopy as appropriate.<br>– If still no improvement within 2 days, continue steroids and promptly add further immunosuppressant agents (e.g., infliximab at 5 mg/kg IV, may be repeated at 2 and 6 weeks after initial dose at the discretion of the treating provider)). **Caution**: Ensure GI consult to rule out bowel perforation and refer to infliximab label for general guidance before using infliximab. If perforation is suspected, consult a surgeon experienced in abdominal surgery immediately without any delay .<br>– Once the patient is improving, gradually taper steroids over ≥28 days and consider prophylactic antibiotics, antifungals, and anti-PJP treatment (refer to current NCCN guidelines for treatment of cancer-related infections).[a]

Specific Immune-Mediated Reactions			
**Adverse Events**	**Severity Grade of the Event (NCI CTCAE version 5.0)**	**Dose Modifications**	**Toxicity Management**

**Hepatitis (elevated LFTs)**<br>Infliximab should not be used for management of immune-related hepatitis.<br>**PLEASE SEE shaded area immediately below this section to find guidance for management of "Hepatitis (elevated LFTS)" in HCC patients**	**Any Grade**	**General Guidance**	**For Any Grade**<br>– Monitor and evaluate liver function test: AST, ALT, ALP, and TB.<br>– Evaluate for alternative etiologies (e.g., viral hepatitis, disease progression, concomitant medications).
	**Grade 1**<br>(AST or ALT >ULN and ≤3.0×ULN if baseline normal, 1.5-3.0×baseline if baseline abnormal; and/or TB >ULN and ≤1.5×ULN if baseline normal, >1.0-1.5×baseline if baseline abnormal)	• No dose modifications.<br>• If it worsens, then treat as Grade 2.	– Continue LFT monitoring per protocol.
	**Grade 2**<br>(AST or ALT >3.0×ULN and ≤5.0×ULN if baseline normal, >3-5×baseline if baseline abnormal; and/or TB >1.5×ULN and ≤3.0×ULN if baseline normal, >1.5-3.0×baseline if	• Permanently discontinue study drug/study regimen for any case meeting Hy's law criteria (AST and/or ALT >3 × ULN + bilirubin >2 × ULN without initial findings of cholestasis (i.e., elevated alkaline P04) and in the	**For Grade 2:**<br>– Regular and frequent checking of LFTs (e.g., every 1 to 2 days) until elevations of these are improving or resolved.<br>– If no resolution to ≤Grade 1 in 1 to 2 days, consider, as necessary, discussing with study physician.<br>– If event is persistent (>2 to 3 days) or worsens, promptly start prednisone 1 to 2 mg/kg/day PO or IV equivalent.<br>– If still no improvement within 3 to 5 days despite 1 to 2 mg/kg/day of prednisone PO

	baseline abnormal)	absence of any alternative cause.	or IV equivalent, consider additional work up and start prompt treatment with IV methylprednisolone 2 to 4 mg/kg/day.<br>– If still no improvement within 3 to 5 days despite 2 to 4 mg/kg/day of IV methylprednisolone, promptly start immunosuppressives (i.e., mycophenolate mofetil).[a] Discuss with study physician if mycophenolate mofetil is not available. **Infliximab should NOT be used.**<br>– Once the patient is improving, gradually taper steroids over ≥28 days and consider prophylactic antibiotics, antifungals, and anti-PJP treatment (refer to current NCCN guidelines for treatment of cancer-related infections).[a]
	**Grade 3**<br>(AST or ALT >5.0×ULN and ≤20×ULN if baseline normal, >5-20× baseline if baseline abnormal; and/or TB >3.0×ULN and ≤10.0×ULN if baseline normal, >3.0-10.0× baseline if baseline abnormal)<br><br>**Grade 4**<br>(AST or ALT >20×ULN if baseline normal, >20×baseline if baseline abnormal; and/or TB >10×ULN if baseline normal,	For elevations in transaminases ≤8×ULN, or elevations in TB ≤5×ULN:<br>• Hold study drug/study regimen dose until resolution to Grade≤1<br>• Resume study drug/study regimen if elevations downgrade to Grade≤1 within 14 days and after completion of steroid taper.<br>• Permanently discontinue study drug/study regimen if the elevations do not downgrade to Grade≤1 within 14 days.<br>• For elevations in transaminases >8×ULN or elevations in	**For Grade 3 or 4:**<br>– Promptly initiate empiric IV methylprednisolone at 1 to 2 mg/kg/day or equivalent.<br>– If still no improvement within 2 to 3 days despite 1 to 2 mg/kg/day methylprednisolone IV or equivalent, promptly start treatment with immunosuppressive therapy (i.e., mycophenolate mofetil 0.5 – 1 g every 12 hours then taper in consultation with hepatology consult). Discuss with study physician if mycophenolate is not available. **Infliximab should NOT be used.**<br>– Request Hepatology consult, and perform abdominal workup and imaging as appropriate.<br>– Once the patient is improving, gradually taper steroids over ≥28 days and consider prophylactic antibiotics, antifungals, and anti-PJP treatment (refer to current NCCN guidelines for treatment of cancer-related infections).

	>10.0×baseline if baseline abnormal)	bilirubin >5×ULN, permanently discontinue study drug/study regimen.<br>• Permanently discontinue study drug/study regimen for any case meeting Hy's law criteria (AST and/or ALT >3×ULN + bilirubin >2×ULN without initial findings of cholestasis [i.e., elevated alkaline P04] and in the absence of any alternative cause).	

**Hepatitis (elevated LFTs)**<br>Infliximab should not be used for management of immune-related hepatitis.<br>**THIS shaded area is guidance *only* for management of "Hepatitis (elevated LFTs)" in HCC patients**<br>See instructions at bottom of shaded area if transaminase rise is not isolated but (at any time) occurs in setting of either **increasing bilirubin or signs of DILI/liver decompensation**	**Any Elevations of AST, ALT, or TB as Described Below**	**General Guidance**	**For Any Elevations Described:**<br>– Monitor and evaluate liver function test: AST, ALT, ALP, and TB.<br>– Evaluate for alternative etiologies (e.g., viral hepatitis, disease progression, concomitant medications, worsening of liver cirrhosis [e.g., portal vein thrombosis]).<br>– For HBV+ patients: evaluate quantitative HBV viral load, quantitative HBsAg, or HBeAg<br>– For HCV+ patients: evaluate quantitative HCV viral load<br>– Consider consulting hepatologist/Infectious Disease specialist regarding change/implementation in/of antiviral medications for any patient with an elevated HBV viral load >2000 IU/ml<br>– Consider consulting hepatologist/Infectious Disease

			specialist regarding change/implementation in/of antiviral HCV medications if HCV viral load increased by ≥2-fold<br>– For HCV+ with HBcAB+: Evaluate for both HBV and HCV as above
	**Isolated AST or ALT >ULN and ≤5.0×ULN, whether normal or elevated at baseline**	• No dose modifications.<br>• If ALT/AST elevations represents significant worsening based on investigator assessment, then treat as described for elevations in the row below.<br>• For all transaminase elevations, see instructions at bottom of shaded area if transaminase rise is not isolated but (at any time) occurs in setting of either **increasing bilirubin or signs of DILI/liver decompensation**	
	**Isolated AST or ALT >5.0×ULN and ≤8.0×ULN, if normal at baseline**<br><br>**Isolated AST or ALT >2.0×baseline and ≤12.5×ULN, if elevated >ULN at baseline**	• Hold study drug/study regimen dose untilresolution to AST or ALT ≤5.0×ULN.<br>• If toxicity worsens, then treat as described for elevations in the rows below.<br>• If toxicity improves to AST or ALT ≤5.0×ULN, resume study drug/study regimen after completion of steroid taper	– Regular and frequent checking of LFTs (e.g., every 1 to 3 days) until elevations of these are improving or resolved.<br>– Recommend consult hepatologist; consider abdominal ultrasound, including Doppler assessment of liver perfusion.<br>– Consider, as necessary, discussing with study physician.<br>– If event is persistent (>2 to 3 days) or worsens, and investigator suspects toxicity to be immune-mediated AE, recommend to start prednisone 1 to 2 mg/kg/day PO or IV equivalent.<br>– If still no improvement within 2 to 3 days despite 1 to 2 mg/kg/day of

			prednisone PO or IV equivalent, consider additional workup and treatment with IV methylprednisolone 2 to 4 mg/kg/day.<br>– If still no improvement within 2 to 3 days despite 1 to 2 mg/kg/day of IV methylprednisolone, consider additional abdominal workup (including liver biopsy) and imaging (i.e., liver ultrasound), and consider starting immunosuppressives (i.e., mycophenolate mofetil 0.5 – 1 g every 12 hours then taper in consultation with hepatology consult). Discuss with study physician if mycophenolate mofetil is not available. **Infliximab should NOT be used.**
	**Isolated AST or ALT >8.0×ULN and ≤20.0×ULN, if normal at baseline**<br><br>**Isolated AST or ALT >12.5×ULN and ≤20.0×ULN, if elevated >ULN at baseline**	• Hold study drug/study regimen dose until resolution to AST or ALT ≤5.0×ULN<br>• Resume study drug/study regimen if elevations downgrade to AST or ALT ≤5.0×ULN within 14 days and after completion of steroid taper.<br>• Permanently discontinue study drug/study regimen if the elevations do not downgrade to AST or ALT ≤5.0×ULN within 14 days<br>• Permanently discontinue study drug/study regimen for any case meeting Hy's law criteria, in the absence of any alternative cause.	– Regular and frequent checking of LFTs (e.g., every 1-2 days) until elevations of these are improving or resolved.<br>– Consult hepatologist (unless investigator is hepatologist); obtain abdominal ultrasound, including Doppler assessment of liver perfusion; and consider liver biopsy.<br>– Consider, as necessary, discussing with study physician.<br>– If investigator suspects toxicity to be immune-mediated, promptly initiate empiric IV methylprednisolone at 1 to 4 mg/kg/day or equivalent.<br>– If no improvement within 3 to 5 days despite 1 to 4 mg/kg/day methylprednisolone IV or equivalent, obtain liver biopsy (if it has not been done already) and promptly start treatment with immunosuppressive therapy (mycophenolate mofetil). Discuss with study physician if mycophenolate is not available. **Infliximab should NOT be used.**<br>– Once the patient is improving, gradually taper steroids over ≥28 days

			and consider prophylactic antibiotics, antifungals, and anti-PCP treatment (refer to current NCCN guidelines for treatment of cancer-related infections
	**Isolated AST or ALT >20×ULN, whether normal or elevated at baseline**	Permanently discontinue study drug/study regimen	**Same as above (except would recommend obtaining liver biopsy early)**
	**If transaminase rise is not isolated but (at any time) occurs in setting of either increasing total/direct bilirubin (≥1.5×ULN, if normal at baseline; or 2×baseline, if >ULN at baseline) or signs of DILI/liver decompensation (e.g., fever, elevated INR):**<br>**Manage dosing for each level of transaminase rise as instructed for the next highest level of transaminase riseFor example, manage dosing for second level of transaminase rise (i.e., AST or ALT >5.0×ULN and ≤8.0×ULN, if normal at baseline, or AST or ALT >2.0×baseline and ≤12.5×ULN, if elevated >ULN at baseline) as instructed for the third level of transaminase rise (i.e., AST or ALT >8.0×ULN and ≤20.0×ULN, if normal at baseline, or AST or ALT >12.5×ULN and ≤20.0×ULN, if elevated >ULN at baseline)**<br>**For the third and fourth levels of transaminase rises, permanently discontinue study drug/study regimen**		
**Nephritis or renal dysfunction**<br>(elevated serum creatinine)	**Any Grade**	**General Guidance**	**For Any Grade:**<br>– Consult with nephrologist.<br>– Monitor for signs and symptoms that may be related to changes in renal function (e.g., routine urinalysis, elevated serum BUN and creatinine, decreased creatinine clearance, electrolyte imbalance, decrease in urine output, or proteinuria, recent IV contrast, medications, fluid status).<br>– Patients should be thoroughly evaluated to rule out any alternative etiology (e.g., disease progression or infections).<br>– Steroids should be considered in the absence of clear alternative etiology even for low-grade events (Grade 2), in order to prevent potential progression to higher grade event.

	**Grade 1**<br>(serum creatinine >ULN to 1.5×ULN)	No dose modifications.	**For Grade 1:**<br>– Monitor serum creatinine weekly and any accompanying symptoms.<br>• If creatinine returns to baseline, resume its regular monitoring per study protocol.<br>• If creatinine worsens, depending on the severity, treat as Grade 2, 3, or 4.<br>– Consider symptomatic treatment, including hydration, electrolyte replacement, and diuretics.<br>– If baseline serum creatinine is elevated above normal, and there is a rise to > 1 to 1.5 × baseline, consider following recommendations in this row.
	**Grade 2**<br>(serum creatinine >1.5 to 3.0×baseline; >1.5 to 3.0×ULN)	Hold study drug/study regimen until resolution to Grade ≤1 or baseline.<br>• If toxicity worsens, then treat as Grade 3 or 4.<br>• If toxicity improves to Grade ≤1 or baseline, then resume study drug/study regimen after completion of steroid taper.	**For Grade 2:**<br>– Consider symptomatic treatment, including hydration, electrolyte replacement, and diuretics.<br>– Carefully monitor serum creatinine every 2 to 3 days and as clinically warranted.<br>– Consult nephrologist and consider renal biopsy if clinically indicated.<br>– If event is persistent beyond 3 to 5 days or worsens, promptly start prednisone 1 to 2 mg/kg/day PO or IV equivalent.<br>– If event is not responsive within 3 to 5 days or worsens despite prednisone at 1 to 2 mg/kg/day PO or IV equivalent, consider additional workup .<br>– Once the patient is improving, gradually taper steroids over ≥28 days and consider prophylactic antibiotics, antifungals, and anti-PJP treatment (refer to current NCCN guidelines for treatment of cancer-related infections).[a]<br>– When event returns to baseline, resume study drug/study regimen and routine

			serum creatinine monitoring per study protocol.
	**Grade 3 or 4**<br>(Grade 3: serum creatinine >3.0×baseline; >3.0 to 6.0×ULN)<br><br>(Grade 4: serum creatinine >6.0×ULN)	Permanently discontinue study drug/study regimen.	**For Grade 3 or 4:**<br>– Carefully monitor serum creatinine on daily basis.<br>– Consult nephrologist and consider renal biopsy if clinically indicated.<br>– Promptly start prednisone 1 to 2 mg/kg/day PO or IV equivalent.<br>– If event is not responsive within 3 to 5 days or worsens despite prednisone at 1 to 2 mg/kg/day PO or IV equivalent, additional workup should be considered and prompt treatment with an immunosuppressive in consultation with a nephrologist .<br>– Once the patient is improving, gradually taper steroids over ≥28 days and consider prophylactic antibiotics, antifungals, and anti-PJP treatment (refer to current NCCN guidelines for treatment of cancer-related infections).[a]
**Rash or Dermatitis (including Pemphigoid)**	**Any Grade**<br>(refer to NCI CTCAE v 5.0 for definition of severity/grade depending on type of skin rash)	**General Guidance**	**For Any Grade:**<br>– Monitor for signs and symptoms of dermatitis (rash and pruritus).<br>– Hold study drug if Stevens-Johnson Syndrome (SJS), Toxic Epidermal Necrolysis (TEN), or other severe cutaneous adverse reaction (SCAR) is suspected<br>– Permanently discontinue study drugs if SJS, TEN or SCAR is confirmed.
	**Grade 1**	No dose modifications.	**For Grade 1:**<br>– Consider symptomatic treatment, including oral antipruritics (e.g., diphenhydramine or hydroxyzine) and topical therapy (e.g., emollient, lotion, or institutional standard).

	**Grade 2**	• For persistent (>1 week) Grade 2 events, hold scheduled study drug/study regimen until resolution to Grade ≤1 or baseline.<br>• If toxicity worsens, then treat as Grade 3.<br>• If toxicity improves to Grade ≤1 or baseline, then resume drug/study regimen after completion of steroid taper.	**For Grade 2:**<br>– Obtain Dermatology consult.<br>– Consider symptomatic treatment, including oral antipruritics (e.g., diphenhydramine or hydroxyzine) and topical therapy (e.g., urea cream).<br>– Consider moderate-strength topical steroid.<br>– If no improvement of rash/skin lesions occurs within 3 to 5 days or is worsening despite symptomatic treatment and/or use of moderate strength topical steroid, consider, as necessary, discussing with study physician and promptly start systemic steroids such as prednisone 1 to 2 mg/kg/day PO or IV equivalent. If > 30% body surface area is involved, consider initiation of systemic steroids promptly.<br>– Consider skin biopsy if the event is persistent for >1 week or recurs.
	**Grade 3 or 4**	**For Grade 3:**<br>Hold study drug/study regimen until resolution to Grade ≤1 or baseline.<br>If toxicity improves to Grade ≤1 or baseline, then resume drug/study regimen after completion of steroid taper.<br>If toxicity worsens, then treat as Grade 4.<br><br>**For Grade 4 (or life-threatening):**<br>Permanently discontinue study drug/study regimen.	**For Grade 3 or 4 (or life-threatening):**<br>– Consult Dermatology.<br>– Promptly initiate empiric IV methylprednisolone 1 to 2 mg/kg/day or equivalent.<br>– Consider hospitalization.<br>– Monitor extent of rash [Rule of Nines].<br>– Consider skin biopsy (preferably more than 1) as clinically feasible.<br>– Once the patient is improving, gradually taper steroids over ≥28 days and consider prophylactic antibiotics, antifungals, and anti-PJP treatment (refer to current NCCN guidelines for treatment of cancer-related infections).[a]<br>– Consider, as necessary, discussing with study physician.
**Endocrinopathy**<br>(e.g., hyperthyroidism, thyroiditis,	**Any Grade**<br>(depending on the type of	**General Guidance**	**For Any Grade:**<br>– Consider consulting an endocrinologist for endocrine events.

hypothyroidism, Type 1 diabetes mellitus, hypophysitis, hypopituitarism, and adrenal insufficiency; exocrine event of amylase/lipase increased also included in this section)	endocrinopathy, refer to NCI CTCAE v5.0 for defining the CTC grade/severity)		– Consider, as necessary, discussing with study physician.<br>– Monitor patients for signs and symptoms of endocrinopathies. Non-specific symptoms include headache, fatigue, behavior changes, changed mental status, vertigo, abdominal pain, unusual bowel habits, polydipsia, polyuria, hypotension, photophobia, visual field cuts and weakness.<br>– Patients should be thoroughly evaluated to rule out any alternative etiology (e.g., disease progression including brain metastases, or infections).<br>– Depending on the suspected endocrinopathy, monitor and evaluate thyroid function tests: TSH, free T3 and free T4 and other relevant endocrine and related labs (e.g., blood glucose and ketone levels, HgA1c).<br>– Investigators should ask subjects with endocrinopathies who may require prolonged or continued hormonal replacement, to consult their primary care physicians or endocrinologists about further monitoring and treatment after completion of the study.<br>– If a patient experiences an AE that is thought to be possibly of autoimmune nature (e.g., thyroiditis, pancreatitis, hypophysitis, or diabetes insipidus), the investigator should send a blood sample for appropriate autoimmune antibody testing.
	**Grade 1**	No dose modifications.	**For Grade 1 (including those with asymptomatic TSH elevation):**<br>– Monitor patient with appropriate endocrine function tests.<br>– For suspected hypophysitis/hypopituitarism, consider consultation of an endocrinologist to guide

			assessment of early-morning ACTH, cortisol, TSH and free T4; also consider gonadotropins, sex hormones, and prolactin levels, as well as cosyntropin stimulation test (though it may not be useful in diagnosing early secondary adrenal insufficiency).<br>– If TSH < 0.5 × LLN, or TSH >2 × ULN, or consistently out of range in 2 subsequent measurements, include free T4 at subsequent cycles as clinically indicated and consider consultation of an endocrinologist.
	**Grade 2**	For Grade 2 endocrinopathy other than hypothyroidism and Type 1 diabetes mellitus, hold study drug/study regimen dose until patient is clinically stable.<br>• If toxicity worsens, then treat as Grade 3 or Grade 4.<br>Study drug/study regimen can be resumed once event stabilizes and after completion of steroid taper.<br>Patients with endocrinopathies who may require prolonged or continued steroid replacement (e.g., adrenal insufficiency) can be retreated with study drug/study regimen on the following conditions:<br>1. The event stabilizes and is controlled.<br>2. The patient is clinically stable as per investigator or	**For Grade 2 (including those with symptomatic endocrinopathy):**<br>– Consult endocrinologist to guide evaluation of endocrine function and, as indicated by suspected endocrinopathy and as clinically indicated, consider pituitary scan.<br>– For all patients with abnormal endocrine work up, except those with isolated hypothyroidism or Type 1 DM, and as guided by an endocrinologist, consider short-term corticosteroids (e.g., 1 to 2 mg/kg/day methylprednisolone or IV equivalent) and prompt initiation of treatment with relevant hormone replacement (e.g., hydrocortisone, sex hormones).<br>– Isolated hypothyroidism may be treated with replacement therapy, without study drug/study regimen interruption, and without corticosteroids.<br>– Isolated Type 1 diabetes mellitus (DM) may be treated with appropriate diabetic therapy, without study drug/study regimen interruption, and without corticosteroids. Only hold study drug/study regimen in setting of hyperglycemia when diagnostic workup is positive for diabetic ketoacidosis.

		treating physician's clinical judgement.<br>3. Doses of prednisone are ≤10 mg/day or equivalent.	– Once patients on steroids are improving, gradually taper immunosuppressive steroids (as appropriate and with guidance of endocrinologist) over ≥28 days and consider prophylactic antibiotics, antifungals, and anti-PJP treatment (refer to current NCCN guidelines for treatment of cancer-related infections).[a]<br>– For patients with normal endocrine workup (laboratory assessment or MRI scans), repeat laboratory assessments/MRI as clinically indicated.
	**Grade 3 or 4**	For Grade 3 or 4 endocrinopathy other than hypothyroidism and Type 1 diabetes mellitus, hold study drug/study regimen dose until endocrinopathy symptom(s) are controlled.<br>Study drug/study regimen can be resumed once event stabilizes and after completion of steroid taper.<br>Patients with endocrinopathies who may require prolonged or continued steroid replacement (e.g., adrenal insufficiency) can be retreated with study drug/study regimen on the following conditions:<br>1. The event stabilizes and is controlled.<br>2. The patient is clinically stable as per investigator or treating physician's clinical judgement.	**For Grade 3 or 4:**<br>– Consult endocrinologist to guide evaluation of endocrine function and, as indicated by suspected endocrinopathy and as clinically indicated, consider pituitary scan. Hospitalization recommended.<br>– For all patients with abnormal endocrine work up, except those with isolated hypothyroidism or Type 1 DM, and as guided by an endocrinologist, promptly initiate empiric IV methylprednisolone 1 to 2 mg/kg/day or equivalent, as well as relevant hormone replacement (e.g., hydrocortisone, sex hormones).<br>– For adrenal crisis, severe dehydration, hypotension, or shock, immediately initiate IV corticosteroids with mineralocorticoid activity.<br>– Isolated hypothyroidism may be treated with replacement therapy, without study drug/study regimen interruption, and without corticosteroids.<br>– Isolated Type 1 diabetes mellitus may be treated with appropriate diabetic therapy, without study drug/study regimen interruption, and without corticosteroids. Only hold study drug/study regimen in setting of hyperglycemia when diagnostic workup is positive for diabetic ketoacidosis.

		3. Doses of prednisone are ≤10 mg/day or equivalent.	− Once patients on steroids are improving, gradually taper immunosuppressive steroids (as appropriate and with guidance of endocrinologist) over ≥28 days and consider prophylactic antibiotics, antifungals, and anti-PJP treatment (refer to current NCCN guidelines for treatment of cancer-related infections).
Amylase/Lipase increased		Dose Modifications (General Guidance): none<br>Dose Modifications (Grade 1): No dose modifications<br>Dose Modifications (Grade 2, 3 or 4):<br>In consultation with relevant pancreatic specialist consider continuing study drug/study regimen if no clinical/radiologic evidence of pancreatitis ± improvement in amylase/lipase.	For Any Grade<br>−For modest asymptomatic elevations in serum amylase and lipase, corticosteroid treatment is not indicated as long as there are no other signs or symptoms of pancreatic inflammation.<br>−If isolated elevation of enzymes without evidence of pancreatitis, continue immunotherapy.<br>−Assess for signs/symptoms of pancreatitis<br>−Consider appropriate diagnostic testing (e.g, abdominal CT with contrast, MRCP if clinical suspicion of pancreatitis and no radiologic evidence on CT)<br>−Consider other causes of elevated amylase/lipase<br>−If evidence of pancreatitis, manage according to pancreatitis recommendations
Acute Pancreatitis		Dose Modifications (General Guidance): none<br>Dose Modifications (Grade 1): No dose modifications<br>Dose Modifications (Grade 2): Hold study drug/study regimen dose until resolution to Grade ≤1. Consider resumption of study drug/study regimen if no clinical/radiologic evidence of pancreatitis ± improvement in amylase/lipase in	For Any Grade<br>-Consider gastroenterology referral<br>For Grade 1:<br>-IV hydration<br>-Manage per amylase/lipase increased (asymptomatic)<br>For Grade 2,3 or 4:<br>-Promptly start systemic steroids prednisone 1 to 2 mg/kg/day PO or IV equivalent<br>-IV hydration

		consultation with relevant pancreatic specialist<br>Dose Modification (Grade 3 or 4): Permanently discontinue study drug/study regimen	
**Neurotoxicity**<br>(to include but not be limited to limbic encephalitis and autonomic neuropathy, excluding Myasthenia Gravis and Guillain-Barre)	**Any Grade**<br>(depending on the type of neurotoxicity, refer to NCI CTCAE v5.0 for defining the CTC grade/severity)	**General Guidance**	**For Any Grade:**<br>– FOR TRANSVERSE MYELITIS, PERMANENTLY DISCONTINUE FOR ANY GRADE<br>– Patients should be evaluated to rule out any alternative etiology (e.g., disease progression, infections, metabolic syndromes, or medications).<br>– Monitor patient for general symptoms (headache, nausea, vertigo, behavior change, or weakness).<br>– Consider appropriate diagnostic testing (e.g., electromyogram and nerve conduction investigations).<br>– Perform symptomatic treatment with Neurology consult as appropriate.

	**Grade 1**	No dose modifications.	**For Grade 1:**<br>– See "Any Grade" recommendations above.<br>Treat mild signs/symptoms as Grade 1 (e.g. loss of deep tendon reflexes or paresthesia)
	**Grade 2**	For acute motor neuropathies or neurotoxicity, hold study drug/study regimen dose until resolution to Grade ≤1.<br>For sensory neuropathy/neuropathic pain, consider holding study drug/study regimen dose until resolution to Grade ≤1.<br>If toxicity worsens, then treat as Grade 3 or 4.<br>Permanently discontinue study drug/study regimen if Grade 2 imAE does not resolve to Grade ≤1 within 30 days .	**For Grade 2:**<br>– Consider, as necessary, discussing with the study physician.<br>– Obtain Neurology consult.<br>– Sensory neuropathy/neuropathic pain may be managed by appropriate medications (e.g., gabapentin or duloxetine).<br>– Promptly start systemic steroids prednisone 1 to 2 mg/kg/day PO or IV equivalent.<br>– If no improvement within 2 to 3 days despite 1 to 2 mg/kg/day prednisone PO or IV equivalent, consider additional workup and promptly treat with additional immunosuppressive therapy (e.g., IV IG other immunosuppressive depending on the specific imAE).
	**Grade 3 or 4**	**For Grade 3 or 4:**<br>Permanently discontinue study drug/study regimen.	**For Grade 3 or 4:**<br>– Consider, as necessary, discussing with study physician.<br>– Obtain Neurology consult.<br>– Consider hospitalization.<br>– Promptly initiate empiric IV methylprednisolone 1 to 2 mg/kg/day or equivalent.<br>– If no improvement within 2 to 3 days despite IV corticosteroids, consider additional workup and promptly treat with additional immunosuppressants (e.g., IV IG or other immunosuppressive depending on the specific imAE).<br>Once stable, gradually taper steroids over ≥28 days.
**Peripheral neuromotor syndromes**	**Any Grade**	**General Guidance**	**For Any Grade:**<br>– The prompt diagnosis of immune-mediated peripheral neuromotor syndromes is important, since certain patients may

(such as Guillain-Barre and myasthenia gravis)			unpredictably experience acute decompensations that can result in substantial morbidity or in the worst case, death. Special care should be taken for certain sentinel symptoms that may predict a more severe outcome, such as prominent dysphagia, rapidly progressive weakness, and signs of respiratory insufficiency or autonomic instability.<br>– Patients should be evaluated to rule out any alternative etiology (e.g., disease progression, infections, metabolic syndromes or medications). It should be noted that the diagnosis of immune-mediated peripheral neuromotor syndromes can be particularly challenging in patients with underlying cancer, due to the multiple potential confounding effects of cancer (and its treatments) throughout the neuraxis. Given the importance of prompt and accurate diagnosis, it is essential to have a low threshold to obtain a Neurology consult.<br>– Neurophysiologic diagnostic testing (e.g., electromyogram and nerve conduction investigations, and "repetitive stimulation" if myasthenia is suspected) are routinely indicated upon suspicion of such conditions and may be best facilitated by means of a Neurology consultation.<br>– It is important to consider that the use of steroids as the primary treatment of Guillain-Barre is not typically considered effective. Patients requiring treatment should be started with IV IG and followed by plasmapheresis if not responsive to IV IG.
	**Grade 1**<br>(Guillain-Barre [GB]: mild symptoms)<br>(Myasthenia gravis [MG]: asymptomatic or	No dose modifications.	**For Grade 1:**<br>– Consider, as necessary, discussing with the study physician.<br>– Care should be taken to monitor patients for sentinel symptoms of a potential decompensation as described above.

	mild symptoms; clinical or diagnostic observations only; intervention not indicated)		Obtain a Neurology consult.
	**Grade 2**<br>(GB: moderate symptoms; limiting instrumental ADL)<br>(MG: moderate; minimal, local or noninvasive intervention indicated; limiting age-appropriate instrumental ADL)	Hold study drug/study regimen dose until resolution to Grade ≤1.<br>Permanently discontinue study drug/study regimen if it does not resolve to Grade ≤1 within 30 days or if there are signs of respiratory insufficiency or autonomic instability.	**For Grade 2:**<br>– Consider, as necessary, discussing with the study physician.<br>– Care should be taken to monitor patients for sentinel symptoms of a potential decompensation as described above.<br>– Obtain a Neurology consult<br>– Sensory neuropathy/neuropathic pain may be managed by appropriate medications (e.g., gabapentin or duloxetine).<br>*MYASTHENIA GRAVIS:*<br>– Steroids may be successfully used to treat myasthenia gravis. It is important to consider that steroid therapy (especially with high doses) may result in transient worsening of myasthenia and should typically be administered in a monitored setting under supervision of a consulting neurologist.<br>o Patients unable to tolerate steroids may be candidates for treatment with plasmapheresis or IV IG. Such decisions are best made in consultation with a neurologist, taking into account the unique needs of each patient.<br>o If myasthenia gravis-like neurotoxicity is present, consider starting AChE inhibitor therapy in addition to steroids. Such therapy, if successful, can also serve to reinforce the diagnosis.<br>o Avoid medications that can worsen myasthenia gravis<br>*GUILLAIN-BARRE:*<br>o It is important to consider here that the use of steroids as the primary treatment

			of Guillain-Barre is not typically considered effective.<br>o Patients requiring treatment should be started with IV IG and followed by plasmapheresis if not responsive to IV IG.
	**Grade 3 or 4**<br>(Grade 3 GB: severe symptoms; limiting self care ADL;<br>Grade 4 GB: life-threatening consequences; urgent intervention indicated; intubation)<br>(Grade 3 MG: severe or medically significant but not immediately life-threatening; hospitalization or prolongation of existing hospitalization indicated; limiting self care ADL;<br>Grade 4 MG: life-threatening consequences; urgent intervention indicated)	**For Grade 3:**<br>Hold study drug/study regimen dose until resolution to Grade ≤1.<br>Permanently discontinue study drug/study regimen if Grade 3 imAE does not resolve to Grade ≤1 within 30 days or if there are signs of respiratory insufficiency or autonomic instability.<br><br>**For Grade 4:**<br>Permanently discontinue study drug/study regimen.	**For Grade 3 or 4 (severe or life-threatening events):**<br>– Consider, as necessary, discussing with study physician.<br>– Recommend hospitalization.<br>– Monitor symptoms and obtain Neurology consult.<br>*MYASTHENIA GRAVIS:*<br>– Steroids may be successfully used to treat myasthenia gravis. They should typically be administered in a monitored setting under supervision of a consulting neurologist.<br>– Patients unable to tolerate steroids may be candidates for treatment with plasmapheresis or IV IG.<br>– If myasthenia gravis-like neurotoxicity present, consider starting AChE inhibitor therapy in addition to steroids. Such therapy, if successful, can also serve to reinforce the diagnosis.<br>*GUILLAIN-BARRE:*<br>– It is important to consider here that the use of steroids as the primary treatment of Guillain-Barre is not typically considered effective.<br>– Patients requiring treatment should be started with IV IG and followed by plasmapheresis if not responsive to IV IG.
**Myocarditis**	**Any Grade**	**General Guidance**<br>Discontinue drug permanently if biopsy-proven immune-mediated myocarditis.	**For Any Grade:**<br>– The prompt diagnosis of immune-mediated myocarditis is important, particularly in

			patients with baseline cardiopulmonary disease and reduced cardiac function.<br>– Consider, as necessary, discussing with the study physician.<br>– Monitor patients for signs and symptoms of myocarditis (new onset or worsening chest pain, arrhythmia, shortness of breath, peripheral edema). As some symptoms can overlap with lung toxicities, simultaneously evaluate for and rule out pulmonary toxicity as well as other causes (e.g., pulmonary embolism, congestive heart failure, malignant pericardial effusion). A Cardiology consultation should be obtained early, with prompt assessment of whether and when to complete a cardiac biopsy, including any other diagnostic procedures.<br>– Initial work-up should include clinical evaluation, BNP, cardiac enzymes, ECG, echocardiogram (ECHO), monitoring of oxygenation via pulse oximetry (resting and exertion), and additional laboratory work-up as indicated. Spiral CT or cardiac MRI can complement ECHO to assess wall motion abnormalities when needed.<br>Patients should be thoroughly evaluated to rule out any alternative etiology (e.g., disease progression, other medications, or infections)

	**Grade 1**<br>(asymptomatic or mild symptoms*; clinical or diagnostic observations only; intervention not indicated)<br>*Treat myocarditis with mild symptoms as Grade 2.	No dose modifications required unless clinical suspicion is high, in which case hold study drug/study regimen dose during diagnostic work-up for other etiologies. If study drug/study regimen is held, resume after complete resolution to Grade 0.	**For Grade 1 (no definitive findings):**<br>- Monitor and closely follow up in 2 to 4 days for clinical symptoms, BNP, cardiac enzymes, ECG, ECHO, pulse oximetry (resting and exertion), and laboratory work-up as clinically indicated.<br>- Consider using steroids if clinical suspicion is high.

	**Grade 2, 3 or 4**<br>(Grade 2: Symptoms with moderate activity or exertion)<br>(Grade 3: Severe with symptoms at rest or with minimal activity or exertion; inter-vention indicated; new onset of symptoms*)<br>(Grade 4: Life-threatening conse-quences; urgent intervention indicated (e.g., continuous IV therapy or mechanical hemodynamic support))<br><br>* Consider "new onset of symptoms" as referring to patients with prior episode of myocarditis.	- If Grade 2 -- Hold study drug/study regimen dose until resolution to Grade 0. If toxicity rapidly improves to Grade 0, then the decision to reinitiate study drug/study regimen will be based upon treating physician's clinical judgment and after completion of steroid taper. If toxicity does not rapidly improve, permanently. discontinue study drug/study regimen.<br>If Grade 3-4, permanently discontinue study drug/study regimen.	**For Grade 2-4:**<br>– Monitor symptoms daily, hospitalize.<br>– Promptly start IV methylprednisolone 2 to 4 mg/kg/day or equivalent after Cardiology consultation has determined whether and when to complete diagnostic procedures including a cardiac biopsy.<br>– Supportive care (e.g., oxygen).<br>– If no improvement within 2 to 3 days despite IV methylprednisolone at 2 to 4 mg/kg/day, promptly start immunosuppressive therapy such as TNF inhibitors (e.g., infliximab at 5 mg/kg IV, may be repeated at 2 and 6 weeks after initial dose at the discretion of the treating provider). Caution: It is important to rule out sepsis and refer to infliximab label for general guidance before using infliximab. Infliximab is contraindicated for patients who have heart failure.<br>– Once the patient is improving, gradually taper steroids over ≥28 days and consider prophylactic antibiotics, antifungals, or anti-PJP treatment (refer to current NCCN guidelines for treatment of cancer-related infections).[a]
**Myositis/Polymyositis ("Poly/myositis")**	**Any Grade**	**General Guidance**	**For Any Grade:**<br>– Monitor patients for signs and symptoms of poly/myositis. Typically, muscle weakness/pain occurs in proximal muscles including upper arms, thighs, shoulders, hips, neck and back, but rarely affects the extremities including hands and fingers; also difficulty breathing and/or trouble swallowing can occur and progress rapidly. Increased general feelings of tiredness and fatigue may occur, and there can be new-onset falling, difficulty getting up from a fall, and

			trouble climbing stairs, standing up from a seated position, and/or reaching up.<br>– If poly/myositis is suspected, a Neurology consultation should be obtained early, with prompt guidance on diagnostic procedures. Myocarditis may co-occur with poly/myositis; refer to guidance under Myocarditis. Given breathing complications, refer to guidance under Pneumonitis/ILD.<br>Given possibility of an existent (but previously unknown) autoimmune disorder, consider Rheumatology consultation.<br>– Consider, as necessary, discussing with the study physician.<br>– Initial work-up should include clinical evaluation, creatine kinase, aldolase, LDH, BUN/creatinine, erythrocyte sedimentation rate or C-reactive protein level, urine myoglobin, and additional laboratory work-up as indicated, including a number of possible rheumatological/antibody tests (i.e., consider whether a rheumatologist consultation is indicated and could guide need for rheumatoid factor, antinuclear antibody, anti-smooth muscle, antisynthetase [such as anti-Jo-1], and/or signal-recognition particle antibodies). Confirmatory testing may include electromyography, nerve conduction studies, MRI of the muscles, and/or a muscle biopsy. Consider Barium
			– swallow for evaluation of dysphagia or dysphonia.<br>Patients should be thoroughly evaluated to rule out any alternative etiology (e.g., disease progression, other medications, or infections).
	**Grade 1**<br>(mild pain)	- No dose modifications.	**For Grade 1:**

			– Monitor and closely follow up in 2 to 4 days for clinical symptoms and initiate evaluation as clinically indicated.<br>– Consider Neurology consult.<br>– Consider, as necessary, discussing with the study physician.
	**Grade 2**<br>(moderate pain associated with weakness; pain limiting instrumental activities of daily living [ADLs])	Hold study drug/study regimen dose until resolution to Grade ≤1.<br>- Permanently discontinue study drug/study regimen if it does not resolve to Grade ≤1 within 30 days or if there are signs of respiratory insufficiency.	**For Grade 2:**<br>– Monitor symptoms daily and consider hospitalization.<br>– Obtain Neurology consult, and initiate evaluation.<br>– Consider, as necessary, discussing with the study physician.<br>– If clinical course is rapidly progressive (particularly if difficulty breathing and/or trouble swallowing), promptly start IV methylprednisolone 2 to 4 mg/kg/day systemic steroids along with receiving input from Neurology consultant<br>– If clinical course is *not* rapidly progressive, start systemic steroids (e.g., prednisone 1 to 2 mg/kg/day PO or IV equivalent); if no improvement within 2 to 3 days, continue additional work up and start treatment with IV methylprednisolone 2 to 4 mg/kg/day<br>– If after start of IV methylprednisolone at 2 to 4 mg/kg/day there is no improvement within 2 to 3 days, consider start of immunosuppressive therapy such as TNF inhibitors (e.g., infliximab at 5 mg/kg IV, may be repeated at 2 and 6 weeks after initial dose at the discretion of the treating provider). Caution: It is important to rule out sepsis and refer to infliximab label for general guidance before using infliximab.<br>– Once the patient is improving, gradually taper steroids over
			– ≥28 days and consider prophylactic antibiotics, antifungals, or anti-PJP treatment (refer to current NCCN

			guidelines for treatment of cancer-related infections).[a]
	**Grade 3 or 4**<br>(Grade 3: pain associated with severe weakness; limiting self-care ADLs<br><br>Grade 4: life-threatening consequences; urgent intervention indicated)	**For Grade 3:**<br>Hold study drug/study regimen dose until resolution to Grade ≤1.<br>Permanently discontinue study drug/study regimen if Grade 3 imAE does not resolve to Grade ≤1 within 30 days or if there are signs of respiratory insufficiency.<br><br>**For Grade 4:**<br>- Permanently discontinue study drug/study regimen.	**For Grade 3 or 4 (severe or life-threatening events):**<br>– Monitor symptoms closely; recommend hospitalization.<br>– Obtain Neurology consult, and complete full evaluation.<br>– Consider, as necessary, discussing with the study physician.<br>– Promptly start IV methylprednisolone 2 to 4 mg/kg/day systemic steroids along with receiving input from Neurology consultant.<br>– If after start of IV methylprednisolone at 2 to 4 mg/kg/day there is no improvement within 2 to 3 days, consider start of immunosuppressive therapy such as TNF inhibitors (e.g., infliximab at 5 mg/kg IV, may be repeated at 2 and 6 weeks after initial dose at the discretion of the treating provider). Caution: It is important to rule out sepsis and refer to infliximab label for general guidance before using infliximab.<br>– Consider whether patient may require IV IG, plasmapheresis.<br>– Once the patient is improving, gradually taper steroids over ≥28 days and consider prophylactic antibiotics, antifungals, or anti-PJP treatment (refer to current NCCN guidelines for treatment of cancer-related infections).[a]
Other Immune Mediated Reactions		Dose Modifications (Any Grade): *Note it is possible that events with an inflammatory or immune mediated mechanism could occur in nearly all organs, some of them are not noted specifically in these guidelines<br>Dose Modifications (Grade 1): No dose modifications<br>Dose Modifications (Grade 2): Hold study drug/study regimen until	Any Grade<br>- Thorough evaluation to rule out any alternative etiology (e.g. disease progression, concomitant medications and infections)<br>- Consultation with relevant specialist<br>- Treat accordingly, as per institutional standard<br>Grade 1<br>- Monitor as clinically indicated<br>Grade 2,3,4<br>- Treat accordingly as per institutional standard, appropriate clinical practice guidelines, and other society guidelines (e.g., NCCN, ESMO)

		resolution to ≤Grade 1 or baseline. If toxicity worsens, then treat as Grade 3 or Grade 4. Study drug/study regimen can be resumed once event stabilizes to Grade ≤1 after completion of steroid taper. Consider whether study drug/study regimen should be permanently discontinued in Grade 2 events with high likelihood for morbidity and/or mortality when they do not rapidly improve to Grade <1 upon treatment with systemic steroids and following full taper<br>Dose Modification (Grade 3): Hold study drug/study regimen<br>Dose Modification (Grade 4): Discontinue study drug/study regimen	

[a]ASCO Educational Book 2015 "Managing Immune Checkpoint Blocking Antibody Side Effects" by Michael Postow MD.

[b]FDA Liver Guidance Document 2009 Guidance for Industry: Drug Induced Liver Injury – Premarketing Clinical Evaluation.

AChE Acetylcholine esterase; ADL Activities of daily living; AE Adverse event; ALP Alkaline phosphatase test; ALT Alanine aminotransferase; AST Aspartate aminotransferase; BUN Blood urea nitrogen; CT Computed tomography; CTCAE Common Terminology Criteria for Adverse Events; ILD Interstitial lung disease; imAE immune-mediated adverse event; IG Immunoglobulin; IV Intravenous; GI Gastrointestinal; LFT Liver function tests; LLN Lower limit of normal; MRI Magnetic resonance imaging; NCI National Cancer Institute; NCCN National Comprehensive Cancer Network; PJP *Pneumocystis jirovecii* pneumonia (formerly known as *Pneumocystis carinii* pneumonia); PO By mouth; T3 Triiodothyronine; T4 Thyroxine; TB Total bilirubin; TNF Tumor necrosis factor; TSH Thyroid-stimulating hormone; ULN Upper limit of normal.

Infusion-Related Reactions		
**Severity Grade of the Event (NCI CTCAE version 5.0)**	**Dose Modifications**	**Toxicity Management**
**Any Grade**	General Guidance	**For Any Grade:**<br>– Manage per institutional standard at the discretion of investigator.<br>– Monitor patients for signs and symptoms of infusion-related reactions (e.g., fever and/or shaking chills, flushing and/or itching, alterations in heart rate and blood pressure, dyspnea or chest discomfort, or skin rashes) and anaphylaxis (e.g., generalized urticaria, angioedema, wheezing, hypotension, or tachycardia).
**Grade 1 or 2**	**For Grade 1:**<br>The infusion rate of study drug/study regimen may be decreased by 50% or temporarily interrupted until resolution of the event.<br><br>**For Grade 2:**<br>The infusion rate of study drug/study regimen may be decreased 50% or temporarily interrupted until resolution of the event.<br>Subsequent infusions may be given at 50% of the initial infusion rate.	**For Grade 1 or 2:**<br>– Acetaminophen and/or antihistamines may be administered per institutional standard at the discretion of the investigator.<br>– Consider premedication per institutional standard prior to subsequent doses.<br>– Steroids should not be used for routine premedication of Grade ≤2 infusion reactions.
**Grade 3 or 4**	**For Grade 3 or 4:**<br>Permanently discontinue study drug/study regimen.	**For Grade 3 or 4:**<br>– Manage severe infusion-related reactions per institutional standards (e.g., IM epinephrine, followed by IV diphenhydramine and famotidine, and IV glucocorticoid).

CTCAE Common Terminology Criteria for Adverse Events; IM intramuscular; IV intravenous; NCI National Cancer Institute.

Non–Immune-Mediated Reactions		
**Severity Grade of the Event (NCI CTCAE version 5.0)**	**Dose Modifications**	**Toxicity Management**
**Any Grade**	Note: Dose modifications are not required for AEs not deemed to be related to study treatment (i.e., events due to underlying disease) or for laboratory abnormalities not deemed to be clinically significant.	Treat accordingly, as per institutional standard.
**Grade 1**	No dose modifications.	Treat accordingly, as per institutional standard.
**Grade 2**	Hold study drug/study regimen until resolution to ≤Grade 1 or baseline.	Treat accordingly, as per institutional standard.
**Grade 3**	Hold study drug/study regimen until resolution to ≤Grade 1 or baseline.<br>For AEs that downgrade to ≤Grade 2 within 7 days or resolve to ≤Grade 1 or baseline within 14 days, resume study drug/study regimen administration. Otherwise, discontinue study drug/study regimen.	Treat accordingly, as per institutional standard.
**Grade 4**	Discontinue study drug/study regimen (Note: For Grade 4 labs, decision to discontinue should be based on accompanying clinical signs/symptoms, the Investigator's clinical judgment, and consultation with the Sponsor.).	Treat accordingly, as per institutional standard.

Note: As applicable, for early phase studies, the following sentence may be added: "Any event greater than or equal to Grade 2, please discuss with Study Physician."

AE Adverse event; CTCAE Common Terminology Criteria for Adverse Events; NCI National Cancer Institute.
