# Supplementary material for: Durvalumab and cediranib with and without olaparib in recurrent ovarian cancer: a phase II proof-of-concept study
Source: Nat Commun. 2026 Mar 16;17:4160. doi: 10.1038/s41467-026-70785-6 (PMC13153414; doi:10.1038/s41467-026-70785-6)
Supplement: Supplementary file 2 — Description of Additional Supplementary Files [file 41467_2026_70785_MOESM2_ESM.pdf]

Description of Additional Supplementary Files

Supplementary Data 1. Baseline characteristic.

Supplementary Data 2. Treatment related adverse events.

Supplementary Data 3. GSEA analysis of NCB vs CB in D+O+C arm.

Supplementary Data 4. GSEA analysis of NCB vs CB in D+C arm.

Supplementary Data 5. Differential gene expression analysis of pretreatment samples of NCB vs CB in D+O+C arm.

Supplementary Data 6. RNAseq of pretreatment samples of NCB vs CB in D+C arm.

Supplementary Data 7. GSEA analysis of NCB vs exceptional responders in D+O+C arm.

Supplementary Data 8. GSEA analysis of NCB vs exceptional responders in D+C arm.

Supplementary Data 9. Differential gene expression analysis of pretreatment samples of NCB vs exceptional responders in D+O+C arm.

Supplementary Data 10. STRING analysis of upregulated differential expressed genes in exceptional responders in D+O+C.

Supplementary Data 11. Differential gene expression analysis of pretreatment samples of NCB vs exceptional responders in D+C arm.

Supplementary Data 12. STRING analysis of upregulated differential expressed genes in exceptional responders in D+C.

Supplementary Data 13. GSEA analysis of D+O+C post-treatment vs pre-treatment in CB (n=9) and NCB (n=2).

Supplementary Data 14. Differential gene expression analysis of posttreatment versus pretreatment CB samples in D+O+C arm (n=9 pairs).

Supplementary Data 15. Differential gene expression analysis of posttreatment versus pretreatment NCB samples in D+O+C arm (n=2 pairs).

Supplementary Data 16. GSEA analysis of D+C post-treatment vs pre-treatment in CB (n=6) and NCB (n=3).

Supplementary Data 17. Differential gene expression analysis of posttreatment versus pretreatment CB samples in D+C arm (n=6 pairs).

Supplementary Data 18. Differential gene expression analysis of posttreatment versus pretreatment NCB samples in D+C arm (n=3 pairs).

Supplementary Data 19. Upregulated pathways in NCB in D+O+C and D+C arms.

Supplementary Data 20. Overlapping morphogenesis- and microtubule-related genes for NCB signature.

Supplementary Data 21. Predictive performance of translational signatures in platinum-resistant subgroups.

Supplementary Data 22. Antibodies used in this study.

Supplementary Data 23. Immune phenotypes and functional markers used for flow cytometric analysis.

Supplementary Data 24. Sequences of siRNAs used in this study.
